# Implementing a community-based shared care breast cancer survivorship model in Singapore: a qualitative study among primary care practitioners

**DOI:** 10.1186/s12875-022-01673-3

**Published:** 2022-04-08

**Authors:** Yu Ke, Rose Wai Yee Fok, Yoke Lim Soong, Kiley Wei-Jen Loh, Mohamad Farid, Lian Leng Low, Joanne Hui Min Quah, Farhad Fakhrudin Vasanwala, Sher Guan Low, Ling Ling Soh, Ngiap-Chuan Tan, Alexandre Chan

**Affiliations:** 1 Department of Pharmacy, National University of Singapore, Singapore, Singapore; 2 Division of Medical Oncology, National Cancer Centre Singapore, Singapore, Singapore; 3 Department of Radiation Oncology, National Cancer Centre Singapore, Singapore, Singapore; 4 Singapore General Hospital Singapore (Family Medicine and Continuing Care), Duke-NUS Medical School Singapore, Singapore, Singapore; 5 SingHealth Polyclinics Singapore, Duke-NUS Medical School Singapore, Singapore, Singapore; 6 Institute of Mental Health, Singapore, Singapore; 7 Post-Acute and Continuing Care, Sengkang Community Hospital Singapore, Singapore, Singapore; 8 National Cancer Centre Singapore (Department of Pharmacy), Singapore, Singapore; 9 Department of Clinical Pharmacy Practice, School of Pharmacy & Pharmaceutical Sciences, University of California Irvine, 101 Theory, Suite 100, Mail Code: 3958, Irvine, CA 92697 USA

**Keywords:** Cancer survivor, Breast cancer, Primary care, Continuity of care, Delivery of health care

## Abstract

**Background:**

The adaptability of existing recommendations on shared care implementation to Asian settings is unknown. This qualitative study aims to elicit public- and private-sectors primary care practitioners’ (PCPs) perspectives on the sustainable implementation of a shared care model among breast cancer survivors in Singapore.

**Methods:**

Purposive sampling was employed to engage 70 PCPs from SingHealth Polyclinics, National University Polyclinics, National Healthcare Group Polyclinics, and private practice. Eleven focus groups and six in-depth interviews were conducted between June to November 2018. All sessions were audio-recorded and transcribed verbatim. Guided by the RE-AIM framework, we performed deductive thematic analysis in QSR NVivo 12.

**Results:**

PCPs identified low-risk breast cancer survivors who demonstrated clear acceptability of PCPs’ involvement in follow-up as suitable candidates for shared care. Engagement with institution stakeholders as early adopters is crucial with adequate support through PCP training, return pathways to oncologists, and survivorship care plans as communication tools. Implementation considerations differed across practices. Selection of participating PCPs could consider seniority and interest for public and private practice, respectively. Proposed adoption incentives included increased renumeration for private PCPs and work recognition for public PCPs. Public PCPs further proposed integrating shared care elements to their existing family medicine clinics.

**Conclusions:**

PCPs perceived shared care favorably as it echoed principles of primary care to provide holistic and well-coordinated care. Contextual factors should be considered when adapting implementation recommendations to Asian settings like Singapore. With limited competitive pressure, the government is then pivotal in empowering primary care participation in survivorship shared care delivery.

**Supplementary Information:**

The online version contains supplementary material available at 10.1186/s12875-022-01673-3.

## Background

Breast cancer is the most commonly diagnosed cancer among females worldwide, accounting for 29.1% of new cases in Singapore from 2011 to 2015 [1, 2]. With improvements in screening, diagnosis, and treatment, the number of cancer survivors continues to rise. Singapore, a high-resource country in Asia, currently adopts an oncologist-centric model where cancer is mainly managed in specialist settings with a focus on surveillance [3]. However, breast cancer survivors continue to experience a range of physical, emotional, and social issues in the survivorship phase [4–7]. Specifically, Singapore-based studies have shown that the majority of breast cancer survivors reported at least one unmet care need following treatment, especially in health information and psychological needs, with inadequate health literacy to identify symptoms of psychosocial distress for management [8–10]. Consequently, local practitioners have raised concerns over the sustainability of managing these diverse long-term cancer survivorship issues beyond surveillance in specialist settings [3, 11]. The involvement of primary care practitioners (PCPs) in survivorship care delivery is then appealing as PCPs are well-positioned to address the highlighted care needs in managing psychological concerns, promoting self-management, and advocating preventive health behavior [12–14]. Additionally, PCPs play a crucial role in comorbidity management and identifying early signs of recurrence given 10% annual cancer recurrence rate in the first 5 years [15]. Primary care in Singapore is available through public polyclinics and private general practitioner clinics [16]. Among alternative care models with primary care involvement, the shared care model involving the joint provision of care by oncologists and PCPs demonstrated comparable effectiveness as oncologist-centric model and with higher survivor satisfaction [17–19].

Existing literature on shared care implementation recommendations is predominantly discussed in Western health care systems [20–22]. These recommendations covered care processes, health care professionals training, patient education, and supportive policies. However, the cross-system applicability of these recommendations to Singapore is unclear given differences in health care financing systems and primary health care practice characteristics [23–26]. Specifically, primary care experts in Singapore rated primary care delivery less favorably than those in Western countries like Australia, Canada, and United Kingdom [26]. Furthermore, patient-related barriers specific to Asian breast cancer survivors, such as fear of unplanned hospitalization or receiving inappropriate treatments from primary care providers, were reported [11]. Thus, engaging perspectives of PCPs practicing in Singapore is necessary to contextualize potential implementation strategies, maximizing the envisioned shared care model’s compatibility with the primary landscape in Singapore.

Previously, a qualitative study conducted in Singapore revealed PCPs’ desire and motivation to participate in breast cancer survivorship care [13]. However, the sampled frame of private PCPs alone was not sufficiently extensive to include the perspectives of public PCPs. Divergent views could potentially stem from their differences in financing and education structures. First, private PCPs operate on a fee-for-service model whereas public PCPs deliver subsidized care in government-funded polyclinics. Second, as compared to structured family medicine residency and in-house training programs that are available for public PCPs, engagement with private PCPs in training programs are ad-hoc in nature. Furthermore, the lack of engagement with key opinion leaders holding decisional power over the adoption of new care programs in primary care institutions precluded a thorough discussion of health system issues [27]. This qualitative study then aims to elicit perspectives from a comprehensive range of public and private PCPs, as well as key opinion leaders on the sustainable implementation of a shared care model among breast cancer survivors in Singapore.

## Methods

This study was part of a larger qualitative study that adopted a phenomenological approach to examine PCPs’ perspectives of a breast cancer shared care model in Singapore. By analyzing focus groups discussions (FGDs) and in-depth interviews (IDIs), a previous study has reported on the envisioned roles of PCPs in a shared care landscape [28]. This study complemented the discussion by focusing on the implementation aspects – proposals to design and evaluate a prospective shared care model for breast cancer survivors in Singapore. Written informed consent was obtained from all study participants. This study was approved by the SingHealth Central Institutional Review Board (CIRB 201711-00029).

### Study sample selection

We employed purposive sampling to identify PCPs of different age, qualifications, experience, practice settings, and practice locations to provide a comprehensive and diverse range of perspectives [29]. Key opinion leaders from clinical services experienced in shared care programs in other disease states and residency and college programs were invited to participate in in-depth interviews. We included PCPs who were actively practicing in family medicine with adequate exposure and work experience (defined as three or more years post-graduation). PCPs practicing in non-community areas such as emergency departments and acute care settings were excluded since the provision of survivorship care is usually not the primary goal for cancer survivors as compared to general practice. From June 2018 to November 2018, eligible PCPs from SingHealth Polyclinics (SHP), National University Polyclinics, National Healthcare Group Polyclinics, and private practice were invited to participate in the study via e-mails, followed by confirmation via telephone calls.

### Data collection

Eleven focus groups with PCPs and six in-depth interviews with key opinion leaders were conducted in English at private meeting rooms in National Cancer Centre Singapore and SHP. Each focus group consisted of three to eight participants and lasted for 30 to 80 min. Before each session, participants completed an anonymized survey, obtaining information on their demographics, medical practice, and previous encounters with cancer survivors. Moderator(s) facilitated the sessions using an interview guide developed and pilot tested by the study team (Table 1). A note-taker was present to record non-verbal cues. The main moderator (R.W.Y.F.) is a family physician who encountered some participants during training programs organized by the College of Family Physicians Singapore. The co-moderator and note-taker (A.C. and Y.K.) are health services research pharmacists with no professional relationships with the participants before the sessions and were not involved with recruitment. The moderator(s) began each session with an introduction of the proposed shared care model for breast cancer survivors described by the American Society of Clinical Oncology (Additional file 1) before posing questions [30]. We reimbursed each participant approximately 22 USD to cover transport costs and time. Focus groups and interviews continued until data saturation was achieved where no new themes emerged from additional sessions [31].

**Table 1:** Facilitator guide used in focus group discussions and in-depth interviews

Section	Questions
Background survey on current practice	Can you share with us some of your experience(s) with cancer survivors?
Discuss the perceived barriers of the proposed shared care model	What are some of the barrier(s) that you can foresee with this shared care model – patient related, physician related, and health care system related?
Gather feedback on the Survivorship Care Plan (SCP) to facilitate communications planning	What information should be included in the SCP?
Explore some of the motivations for participation in the shared care model	What are some of your motivation(s) to participate in this shared care model?
Relationship with stakeholders	Who do you think are or should be stakeholders in this shared care model, and possible barrier(s) that affect communication and seamless coordination and transition of care?
Community resources	Who are the community resources available and whom we can engage/ refer for effective shared care?

### Data analysis

Information on participants’ demographics and medical practice were summarized using descriptive statistics. All focus group discussions and in-depth interviews were audio-recorded and transcribed verbatim. We performed deductive thematic analysis [32] in QSR NVivo 12 based on the RE-AIM (reach, effectiveness, adoption, implementation, and maintenance) conceptual framework [33–36]. This framework focuses on dimensions related to the design, dissemination, and implementation of health-related interventions and have demonstrated to be useful across cultures, settings, and health conditions [35]. Three coders (Y.K., G.Y.L.W., and D.Z.W.N.) first familiarized themselves with the transcripts before coding the data independently to generate preliminary themes based on recurring patterns and concepts. These three coders met regularly to revise the thematic structure and to resolve any discrepancies. We repeated the coding processes iteratively, interspersed with the ongoing focus groups or interviews conducted.

Throughout the data analysis, all investigators deliberately and continually engage in critical self-evaluation to examine one’s positionality when reviewing the participants’ accounts. This reflexive process helps to identify potential personal biases that may have influenced data interpretation. We employed strategies including maintaining a clear audit trail of all coding, field notes and reflexive notes [37]. Also, member checking was performed whereby participants were invited to corroborate with the summary of our study findings.

## Results

### Study participants

Among 80 approached eligible participants, three did not respond, and seven did not participate due to scheduling difficulties. Table 2 summarizes the characteristics of the 70 PCPs recruited. The majority were female (51.4%), Chinese (84.3%), aged between 30 and 39 years old (58.6%). The majority had 5 to 15 years of practice experience (68.6%). Most participants practiced in public settings (78.6%) distributed across Singapore geographically. Most participants were managing a monthly patient load > 600 (64.3%) with an average of 5 to 10 min per consultation (62.9%). Also, the majority reviewed < 10 cancer survivors monthly (62.9%) and spent < 20% of total consultation time caring for cancer-related issues (95.7%).

**Table 2:** Participants’ demographics and practice characteristics (*N* = 70)

Characteristic	N (%)
**Demographic**	
Gender	
Male	34 (48.6%)
Ethnicity	
Chinese	59 (84.3%)
Indian	7 (10.0%)
Others	4 (5.7%)
Practice experience (years)	
3–4	4 (5.7%)
5–10	30 (42.9%)
11–15	18 (25.7%)
16–20	5 (7.1%)
> 20	13 (18.6%)
Age (years)	
20–29	5 (7.1%)
30–39	41 (58.6%)
40–49	14 (20.0%)
50–59	10 (14.3%)
**Practice setting**	
Current practice setting	
Polyclinic	55 (78.6%)
Private general practitioner	15 (21.4%)
Practice area	
North	10 (14.3%)
South	15 (21.4%)
East	11 (15.7%)
West	10 (14.3%)
Central	24 (34.3%)
Types of medical records	
Paper records	5 (7.1%)
Partial/ in transition	5 (7.1%)
Full electronic records	60 (85.7%)
**Current experience with patients**	
Average number of patients seen monthly	
< 300	7 (10.0%)
300–400	6 (8.6%)
401–500	3 (4.3%)
501–600	9 (12.9%)
> 600	45 (64.3%)
Average amount of time spent with each patient (minutes)	
< 5	1 (1.4%)
5–10	44 (62.9%)
11–15	21 (30.0%)
16–20	3 (4.3%)
> 20	1 (1.4%)
Average number of cancer survivors seen monthly	
< 5	23 (32.9%)
5–10	21 (30.0%)
11–15	15 (21.4%)
16–20	2 (2.9%)
> 20	9 (12.9%)
Time spent caring for cancer survivors care on cancer-related issues (% of total consultation time spent in practice)	
< 20	67 (95.7%)
20–50	3 (4.3%)

### Themes

Five major themes related the RE-AIM framework were identified and the corresponding subthemes were discussed below. Additional quotes encapsulating the themes discussed are available in Additional file 2.

#### Reach: characterization and effective engagement with target population

Implementation of shared care necessitates a clear characterization of the target group of cancer survivors who are likely to benefit from the new model. PCPs collectively conceptualized ‘low-risk survivors’ as ideal candidates based on clinical features. Survivors should have a stable disease in remission, good survival prospects, and a low risk of recurrence. Some PCPs suggested using 5 years post-diagnosis as a guide to gauge appropriateness. These considerations stemmed from underlying concerns over the extent of additional specialized skills required from PCPs. Additionally, PCPs cited favorable survivor factors such as a high level of disease awareness and comorbidities presentation.

After a clear characterization of the target group, PCPs ascribed oncologists with the pivotal role of educating survivors to address misconceptions on primary care and to avoid a sense of abandonment or appointment defaulting behavior.

*“…patient has been living with this [oncologist-centric] model …; the general understanding is that if you have cancer, you see the specialist.” – IDI#2, public*

*“[Oncologist should provide] reassuring parts for the patients’ level, so that they know it is just going forward, you are not just lagging all behind. So, it’s all to enhance their care…, it’s a lifelong journey.” – FGD#13, private*

Complementing oncologists’ involvement, PCPs articulated other engagement strategies by appealing to survivors’ practical concerns over cost and ease of accessibility. However, private PCPs acknowledged that their higher consultation cost would require financial relievers to enhance acceptability.

*“…because of the cost that may be involved to investigate or to have some therapy … it may be, from the fiscal point of view, much more advantageous for the patient to go back to the hospital.” – FGD#14, private*

Some PCPs remained uncertain over the geographical accessibility benefit of shared care given Singapore’s small size. Nevertheless, they recognized the greater ease of scheduling appointments in the primary care setting than in specialist clinics.

#### Empowering primary care to deliver effective survivorship care

Compliance with evidence-based survivorship care guidelines is crucial to ensure the quality and effectiveness of care. The need for cancer-specific training to equip PCPs with the associated knowledge and skills was a recurring subtheme that resonated with the majority. The training should address PCPs’ knowledge gaps, confidence, and provide increased exposure to managing breast cancer survivors. While PCPs generally agreed that training should begin with a subgroup before disseminating to the larger group, public and private PCPs hold divergent views over the selection criteria for PCPs to receive training. Private PCPs perceived interest as a key factor, whereas public PCPs suggested for senior PCPs in family medicine clinics as suitable candidates due to their longer consultation time slots. PCPs further anticipated challenges related to manpower redistribution to accommodate training needs. They also cautioned about the exclusive responsibility for cancer care associated only with PCPs who completed training.

*“I’m very worried, because with the course, a lot of doctors will say, ‘I never attend the course, I never do.’” – FGD#10, private*

Care coordination and communication between care providers is pertinent to co-manage survivors effectively under shared care. To facilitate this process, PCPs advocated for workflows and protocols to specify ‘red flags’ that trigger timely referral back to the tertiary setting, when necessary, preferably liaising with a direct contactable person in the tertiary system. Furthermore, they suggested for standardized care pathways delineating the systematic management of cancer-related complications. Moreover, some PCPs were concerned about the rigidity of such protocols and reiterated the importance of having clear communication with oncologists to cater to patient-specific issues.

*“…a protocol will not answer all questions for any of the patients, … even though with protocol, it’s very rigid. We don't really have the communication with the oncologists to make sure that… the patient is safe.” – FGD#61, public*

#### Adoption: understanding organizational culture to introduce changes

As adoption of shared care requires institutional level buy-in, one PCP alluded to the concept of ‘early adopters’ to describe the institutional stakeholders who are willing to trial and refine the proposed shared care model on a smaller scale to demonstrate its preliminary value.

*“This [early adopter] group is probably the most motivated group. …when this is more established, it can be ironed out, the workflow [and] the operational processes.” – FGD#13, private*

Key opinion leaders holding decisional power then provided comprehensive views on the routine decision making process over proposed changes at the institutional level. For public institutions, regular clinical governance meetings were held to consider new proposals. Thus, proposed strategies to engage early adopters were built upon the need for a convincing idea pitch, achieved by framing the value of the model into clear mission statements with detailed, precise role differentiation from oncologists, and a clear specification on the scope of disease coverage.

*“It’s a good idea to make it an idea that is yielding value, that requires a lot of talking by stakeholders, and commitment.” – IDI#5, public*

In addition to a clear justification of the proposed model’s value, key opinion leaders highlighted that supportive infrastructure should expand to provide subsidized rates to cancer-related tests or drugs ordered in primary settings. Some participants raised possible incentives to institutions through financial reimbursements or providing public recognition for their commitment to survivorship care.

*“From the private setting, I think in terms of the actual money. For the public setting, in terms of the recognition of [service to] that particular patient, a more complex patient that requires a bit more care.” – FGD#40, public*

#### Implementation: resources required to support shared care delivery

PCPs highlighted implementation resources in three core areas, revolving around the central goal to facilitate smooth care coordination between tertiary and primary settings. First, all participants affirmed the value of a survivorship care plan to document each survivor’s progress through shared care and to encourage patient ownership. Furthermore, the information presented in the plan should be concise to highlight active issues for management. While some private PCPs preferred care plans to be printed on paper so that patients could bring them to consultations, the public PCPs generally supported an electronic format for ease of retrieval and update. Second, enablers of information transfer across settings should be explored. For instance, PCPs could leverage technology platforms to safeguard health records maintenance, construct dynamic care templates, and incorporate intuitive workflow prompts or reminders based on patient-specific data. Lastly, PCPs echoed the need to consolidate existing supportive care services into networks to increase awareness among PCPs, thereby facilitating care referrals to other ancillary partners.

#### Maintenance: promoting sustainable adoption of shared care

The instrumental role that the government play in promoting sustainable adoption of shared care is a key subtheme discussed by PCPs. Specifically, the government’s commitment to reshaping survivors’ mindset and health care financing principles were highlighted. PCPs explained that the government is influential in disseminating a national objective to improve the status of family-based medicine among survivors and shape positive perceptions towards care in the community during survivorship. Additionally, they suggested that government subsidiary schemes coverage should adapt to each survivor’s comorbidities burden (including cancer) using a risk-stratification approach, tailoring funding to the care required proportionately.

*“In Singapore, the concept of having a family doctor is still fairly weak. So, many patients actually do jump around and … the risk of defaulting is very high.” – IDI#2, public*

Key opinion leaders further proposed assimilating elements of shared care into the existing clinic structure within public institutions to boost sustainability. This integration would omit tremendous logistical efforts required to create new designated clinics and address some PCPs’ concerns over an increasing trend of disease-specific clinics within primary care, as these arrangements over-segment each survivor’s diseases instead of holistically managing them.

*“Once the condition is stabilized and well-managed, it can continue on in a family physician clinic where they manage not just the condition, but in the context of the other diseases. Otherwise, there will be multiple specialized clinics and it’s not sustainable.” – IDI#1, public*

## Discussion

This qualitative study elicited a comprehensive range of perspectives on shared care implementation among breast cancer survivors in Singapore from the potential participating PCP pool. By engaging with key opinion leaders in public health care, we incorporated insights on the health system and policy to augment individual accounts. The RE-AIM dimensions shaped the overall thematic structure [38]. Shared care implementation was discussed considering different tiers of the health system – identifying ideal survivor characteristics at the individual level, encouraging uptake by primary care institutions, and advocating for the government’s influence to shape the national cancer survivorship care landscape.

At the survivor level, we identified two crucial steps in the selection of suitable breast cancer survivors for shared care. First, oncologists should ascertain that survivors are at ‘low risk’ for cancer recurrence. This selection criterion complements the risk stratification approach explored in the literature which utilized clinical features to guide decisions on the level of primary care involvement [39, 40]. Second, PCPs ascribed oncologists to have an immense influence on survivors’ perceptions of survivorship care. This result echoed oncologists as pivotal in engaging survivors in shared decision-making, guiding survivors to make informed decisions considering their personal health care preferences [41, 42]. Furthermore, as PCPs perceived higher cancer-related knowledge as a favorable characteristic, decision aids are possible tools that could address the uncertainty and ambiguity associated with shared care.

Adequate care coordination and communication between oncologists and PCPs are essential for effective shared care implementation. Traditionally, Asian oncology practitioners have reported infrequent and poor communication with non-oncology health care providers [11] Thus, insufficient communication and a lack of understanding of PCPs’ care capacity are significant barriers in coordinating follow-up care across practice settings. Our results shed light on two potential proposals to overcome these barriers. First, convenient and standardized communication channels through tools like survivorship care plans and information sharing systems could allay communication concerns. Second, training proposed by PCPs alluded to the involvement of oncologists as partners in training delivery. This opportunity for interaction between care providers not only contributes to a positive implementation climate for shared care [43], but it may also potentially boost oncologists’ confidence in PCPs for cancer-related problems management.

At the primary care institution level, the motivation for a change in care model likely involves targeted engagement with early adopters, a concept featured in our results. Consistent with the diffusion of innovation model, early adopters are driven by a clear vision of the shared care model’s value and are committed to trying new workflows [44]. Notably, the concept of shared care echoed with the value of primary care in four key areas described by Starfield et al.: sustaining close contact with survivors, providing holistic care, building continuity in patient-physician relationships, and coordinating with other specialties [45, 46]. Furthermore, the desirability of primary care’s involvement in shared care is exemplified by their valuable leadership in managing and preventing diseases proactively, as well as addressing unresolved and persistent psychological or social issues [47, 48]. This study was a valuable opportunity to engage leaders of public institutions who are prospective early adopters.

Our results highlighted a potential divergence in private and public primary care institutions’ readiness to adopt shared care. This divergence stemmed from underlying differences in organizational structure and infrastructural support [49]. First, our results suggested a greater ease of PCPs selection from the public than private institutions. Public PCPs presented a more systematic approach where they considered seniority and experience in family medicine. These standardized criteria could be applied readily across public institutions. In contrast, private PCPs operate as independent clinic groups without a centralized governance, explaining their proposal of employing interest in cancer survivorship as the selection factor. Second, training on the usage of survivorship care tools for care coordination resonated with our participants and were increasingly explored in the literature [39, 50]. However, private PCPs face significant barriers as they lacked access to electronic medical records from cancer centers, precluding effective care coordination and communication with oncologists in shared care [51]. Nevertheless, the formation of primary care networks to consolidate private practices by geographical locations serves as a promising initiative to improve the readiness of private practice [52]. These networks that currently map ancillary services to private patients could potentially expand to build collaborative partnerships with cancer centers.

A stark difference in the sustainable implementation of shared care exists between Singapore and the Western world by sources of pressure for a change in care models [53]. Foremost, Singapore’s small land area attenuated competitive pressure between institutions in the same health sector. This size limitation greatly hinders efforts by cancer centers and their community partners to differentiate their design of the shared care model. In contrast, Western countries such as Canada can leverage their distinct regional cancer centers to trial innovative follow-up care models with varying roles and degrees of PCP participation [20]. Additionally, external pressure originating from government policies or professional guidelines is weaker in the Singapore’s health care system that is historically focused on cancer treatment [3]. In contrast, government bodies in United Kingdom and United States both endorsed clear initiatives to address survivorship follow-up care models [54, 55]. Acknowledging the limited competitive pressure in Singapore, our participants reasonably urged for the government to play a more active role in influencing perceptions of primary care through general education and financing policies, highlighting the need for greater external pressure to induce practice change. These results echoed governmental efforts such as the ‘beyond hospital to community’ strategy announced by the Singapore’s Ministry of Health in 2017, potentially expanding the concept of community-based care to cancer survivorship [56]. Besides institutional support, national guidance and funding are exceptionally crucial to bring the shared care model to fruition.

The utility of findings from this study is three-fold. First, insights gathered on shared care implementation can inform the design of a pilot trial. Guided by the RE-AIM framework, the pilot should evaluate the acceptability of shared care among breast cancer survivors and assess the feasibility of care coordination across settings. Second, our results underscored the value and potential for PCPs to partake in survivorship care provision. PCPs’ perspectives are crucial in establishing a compelling case to attract buy-in from primary care institutions to devote manpower and resources to the shared care model. Lastly, the emphasis on empowering breast cancer survivors in their decision-making process justifies subsequent efforts in developing decision aids to facilitate each survivor’s autonomous decision over the adoption of shared care.

There are some limitations in our study. We were unable to engage key opinion leaders from major private primary care groups in Singapore to contrast their perspectives with the health system-related issues raised in this study. Also, our sampling method may have attracted PCPs with a pre-existing interest to participate in cancer survivorship care. Consequently, the range of strategies obtained for engaging the general PCPs pool may be compromised potentially.

## Conclusion

Overall, PCPs perceived shared care favorably as it echoed principles of primary care to provide holistic and well-coordinated care. Contextual factors should be considered when adapting shared care implementation recommendations from Western to Asian settings. With limited competitive pressure to encourage institutions to adopt innovative care models, the Singapore government is pivotal in empowering primary care participation in survivorship care delivery sustainably through national directives and financial support. Future work on piloting shared care should carefully collect relevant data of interest to enhance governmental support, reinforcing the external pressure for change.

## Supplementary Information

**Additional file 1.** Figure depicting the proposed shared care model for breast cancer survivors that was explained to participants.

**Additional file 2.** Table of themes, subthemes, and corresponding supplementary quotes from study participants.

**Additional file 3.** A compressed folder containing the raw data transcripts and demographics data collection form.

## Data Availability

All data generated or analyzed during this study are included in this published article (and its supplementary information files).

## Abbreviations

FGD: Focus groups discussion
IDI: In-depth interview
PCP: Primary care practitioner
RE-AIM: Reach, effectiveness, adoption, implementation, and maintenance
SHP: SingHealth Polyclinics
