# Supplementary material for: Implementing a community-based shared care breast cancer survivorship model in Singapore: a qualitative study among primary care practitioners
Source: BMC Prim Care. 2022 Apr 8;23:73. doi: 10.1186/s12875-022-01673-3 (PMC8991467; doi:10.1186/s12875-022-01673-3)
Supplement: Supplementary file 1 — Additional file 1. Figure depicting the proposed shared care model for breast cancer survivors that was explained to participants. [file 12875_2022_1673_MOESM1_ESM.pdf]

Proposed Model of Care

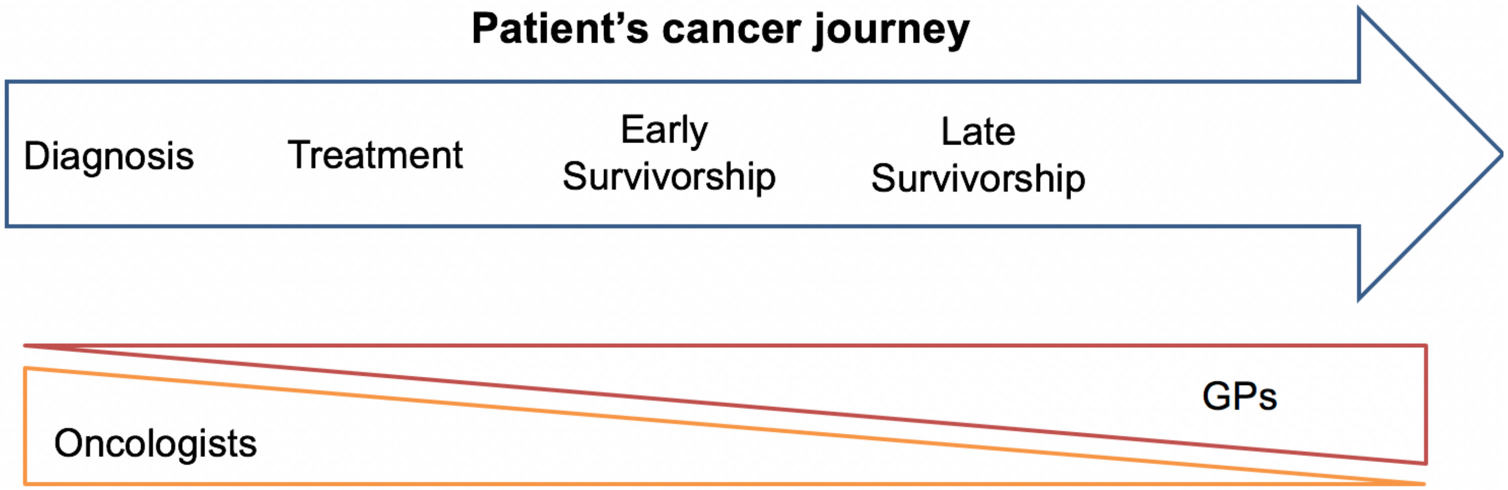

Roles of oncologists

- Cancer diagnosis and treatment
- Management of supportive and survivorship health issues
- Surveillance for recurrence
- Detection of secondary cancers

Roles of GPs

- Management of supportive and survivorship health issues
- Health promotion and prevention of recurrent and other cancers
- Management of chronic diseases
