# Supplementary material for: Implementing a community-based shared care breast cancer survivorship model in Singapore: a qualitative study among primary care practitioners
Source: BMC Prim Care. 2022 Apr 8;23:73. doi: 10.1186/s12875-022-01673-3 (PMC8991467; doi:10.1186/s12875-022-01673-3)
Supplement: Supplementary file 2 — Additional file 2. Table of themes, subthemes, and corresponding supplementary quotes from study participants. [file 12875_2022_1673_MOESM2_ESM.docx]

**Supplementary information file 2**: Themes, subthemes, and corresponding supplementary quotes from study participants.

| **Theme** | **Subtheme** | **Quotes** | **Participant** |
| --- | --- | --- | --- |
| Characterization and effective engagement to **reach** target breast cancer survivors for shared care model” | Survivor characteristics that are suitable for shared care model | “… different patients have different awareness of their illness… patients may not be so aware of their conditions, what sort of treatment, what sort of chemotherapy or hormonal therapy or radiation therapy that they went through. So, the ***patient factors***, their ***own awareness of their own disease*** may not be there…” | FGD#26, public |
|  |  | “I think shared care will be ideal for patients who, especially when they have ***multiple… conditions that the primary (care) physicians can actually handle***.” | FGD#45, public |
|  |  | “For the ***really stabilized patients*** who have been treated and have ***lower risk of recurrence***, all they need is really to have a fixed schedule (to) do this … every five years, ten years, (then) we can certainly do that, so long as we can sort out the financial part for it.” | IDI#2, public |
|  |  | “… there are [a] sub-selected group of patients that are really well and they have maybe ***an early-stage cancer***, they have ***survived for a good five years*** before they come over and see us. I think family physicians are in a good position to co-manage together with the oncologists, but these are really for a ***well-defined subgroup of patients and it cannot be for patients who have multiple oncological issues together with multiple psychosocial issues that require tertiary care***, and which requires multiple disease (referrals) back to the hospital.” | FGD#63, public |
|  | Oncologists should be involved in engaging survivors to avoid sense of abandonment | “***We need to let our oncologists-colleagues do the so-called patient selection***. I think initially, we probably got to start with something more straightforward, maybe five years, whatever participants’ age is.” | FGD#13, private |
|  |  | “Right now, even for patients who are referred to primary care, they are only referred after a long period of time after their cancer has been in remission for many years. So, when patients are referred to primary care, I would imagine that if we have this shared care model where patients are told that they are going to be referred to this primary care provider, ***they might feel very abandoned, like “Is there nobody that is going to take care of my cancer anymore?***”, or they may actually see it as a go-ahead, like, ‘Oh! My cancer is well enough that I don’t really need to see any specialist.’ …***because of this perception, they may be more prone to defaulting***.” | FGD#64, public |
|  |  | “Some patients still need to continue with specialist follow-up, because they are ***worried, especially if it’s cancer***, right? So, I think a lot of it would be also (on) the ***patient education***, and the ***specialist must sing the (same tune)*** that, you know, ‘You will be seen at the Poly(clinic), but if anything were to require escalation, you can have an easy access, a ready access out.’” | IDI#6, public |
|  | Strategies to engage cancer survivors through practical considerations | “As in, when the patient comes, … I think when they pay to see a doctor, it should be ***pegged to the same price as you pay to see a specialist***, otherwise if they pay more, there’s no incentive for them to see the GP (General Practitioner) – they would rather go back to NCC (National Cancer Centre). Maybe just a small difference is okay, but if the difference is relatively big, definitely they will still go back to the specialist centre. So, it really ***depends on how much … the private sector charges***.” | FGD#5, private |
|  |  | “Cheaper… so, the difference is still there. So, that means, instead of going to see the oncologist to do their mammogram or to do whatever, it’s the same. ***Firstly, to reduce visits, and also, it’s cheaper (in) the community, so the value is there***.” | IDI#1, public |
|  |  | “Although we tell patients that ‘you can actually see us and let the specialist handle your main situation’, … we also ***don't want them to travel all over the place***… [which] might actually lead to compliance issues.” | FGD#41, public |
|  |  | “It’s actually the patient’s perception, as well as (the fact that there is) ***no great difference in accessibility between the GPs (General Practitioners) practice versus the oncologist’s practice***, which makes many of them prefer the oncologist, which is, to be frank, a lot less crowded, and waiting time is not as long sometimes as compared to polyclinic waiting time.” | FGD#2, public |
|  |  | “It’s a good plan because primary care clinic, polyclinic and General Practitioners are ***easily accessible*** for the patient and they are within the (region), for example, this resident is around the area to come in and see (us in) the clinic, so it’s ***easy to make that appointment***.” | FGD#17, public |
| Empowering primary care practitioners to deliver **effective** cancer survivorship care to breast cancer survivors | Training required to equip primary care practitioners with knowledge and skills to deliver evidence-based care | “… because the more we do, I think the more experienced we’ll get, so we’ll be a bit more comfortable, and I think it’s also about what the team-let can offer. ***If we keep seeing the same things again and again, then our comfort level gets easier***.” | IDI#4, public |
|  |  | “The whole day (of clinic seeing cancer survivors) will be run by a GP (General Practitioner), ***with the support from the specialist for a few session***(s), then we can ***test-run*** in (the) polyclinic. Then, we can schedule for another ***formal training session*** maybe three months down the road for that kind of practice again, so that they can ***even out the technique and the knowledge***.” | FGD#36, public |
|  |  | “We can reassure the patient as a help to the oncologist as well. And also, ***this teaches us not to be too jittery about what we see post-chemotherapy or ... cancer survivor patients*** as well. So, (we learn) ***not to brush away those side effects***, and chemotherapy side effects that you see or don't see it here before.” | FGD#19, public |
|  | Selecting suitable primary care practitioners to train and participate in shared care | “Probably, we start a bit slower, because currently, family physicians run by MMed (Masters in Medicine) first. I think GDFM (graduate Diploma of Family Medicine) runs last, so maybe ***you can go for those (to) run family physician clinics***… So, we start off with the family physician clinics first.” | IDI#1, public |
|  |  | “…the family physician clinic is for ***complex, chronic patients*** focusing on diabetics. So, if we can actually put the (cancer) patients in that clinic, then it’s actually ***doable with the time (15-20 minutes) that’s accorded***.” | IDI#2, public |
|  |  | “…maybe it might be better to focus on ***educating all primary care physicians about late cancer survivorship***, what to look out for as they grow older, because these are the patients that we are really going to see.” | FGD#64, public |
|  |  | “I think only selected GPs (General Practitioners) – not only selected patients but selected GPs (General Practitioners) - … whom the surgeons can trust to discharge these patients to. So, I don't think it can be a run-of-the-mill GP (General Practitioner). ***Maybe certain regions, certain groups of GPs (General Practitioners) are trained, with a special interest and who are willing to take up this program***.” | FGD#5, private |
|  | Anticipated challenges to accommodate for training needs | “The training part, it certainly would have taken off time too, so more for the ***institution point of view that I’m losing doctors***, still under training, right? And they will get ***backfield*** or something like that.” | IDI#2, public |
|  |  | “Or some will say, ‘I trained in MMed (Master in Medicine in Family Medicine). I train (in) so many things, still cannot handle this sort of things. ***Must attend another course***.’ You know, and then the patient will say, “***Doctor, did you attend this course***?’” | FGD#10, private |
|  | Workflows to facilitate are coordination/ communication between care providers for shared care delivery | “We also need to work out the return path, so that when patients come out, ***there must be quite a seamless arrangement for patients to go back to the hospital if something happens***. And the patients get the confidence. We may need to have something like that, so that when they go to primary care based on all these red flags, they can be sent back to the centre quite seamlessly.” | IDI#2, public |
|  |  | “And the third bit is the referring-back… (there are) two (dimensions): one is sometimes you just ***need to call just for some advice***, a phone number or just email, just a quick thing like, ‘This patient has this thing. Should I worry about it or just do a few things first?’, that kind of thing. And the third one is the ***referral-back, when you think it’s something that is a bit ‘fierce’ (serious) already, and we need to send the patient back***.” | FGD#10, private |
|  |  | “I would suggest that it may be better to have a more ***standardized pathway*** to guide us more on what to do, because we are, in primary care, we are not quite trained in cancer-related (interventions), so we may not be able to know how to.” | FGD#21, public |
|  |  | “It can be at two level(s): one is through a ***backend information-checking***, which means somebody the primary care provider can link up (with), ‘got patient referred here that time and somebody should know.’ …Then, (for) ***what criteria should we refer back and the process (to refer)***.” | IDI#1, public |
| Understanding organizational culture to introduce changes, encouraging **adoption** of the shared care model by institutions | Understanding the routine decision-making process over proposed changes at the institution level | “Usually at first screen, if I pick up some big barrier, I have to raise it up… Usually, if it looks quite doable, then usually what will happen is that… ***I will need to clear that with my clinical governance*** also, so we have our clinic governance meetings every month, where all these new program are being cleared. So, ***once clinically, everyone agrees***, usually the management and all the clinical directors will sit in a meeting and (say) ‘Okay, fine, I think we can do this.’ And ***then, we will look at the operational aspects***.” | IDI#2, public |
|  |  | “(For) example, (there is) no clarity of directive (when) it doesn’t ***come with a clear statement or a few statement(s); not clearly described (to) stakeholders (on) whether it is participation from hospitalists or primary care physicians (and) the roles might not be clear***; the scope of the diseases, for example, (in) psychiatry, ‘mild’ means ‘how mild’?” | IDI#5, public |
|  |  | “So, whether it’s like this example you mentioned, follow-up of breast cancer patient survivors, looking out for either episodic needs which may or may not be related to breast cancer, or aspects of the need that is related to the breast cancer and its treatment complication(s) and so on. ***It’s a good idea to make it an idea that is yielding value, that requires a lot of talking by stakeholders, and commitmen***t.” | IDI#5, public |
|  | Infrastructural resources and incentives to support adoption | “In terms of support resources, so to care for these patients, ***if we need certain scans, or labs and all that to be done, it has to be put in***. So, currently, the lab catalogue that we have, the radiology catalogue that we have may have certain limitations, so you are not be able to do the necessary tests. …We’re not funded for all those tests, so when we do order these tests nowadays, it’s very expensive. So, it’s not sensible for patients to actually have these tests in the community. ***So, we must look at the funding also, to make sure that these tests become affordable when we do it in the polyclinic***.” | IDI#2, public |
|  |  | “The GP (General Practitioner) has to be ***remunerated*** accordingly from whichever source for spending more time and effort, and also for undergoing the training to be able to handle all these things... the ***remuneration part is definitely important… especially in the private sector***.” | FGD#5, private |
|  |  | “There’s some model already used in certain countries. The model is called ‘***Pay For C’, ‘Pay for Coordination’***. So, to do this, can I propose a two-framework of “Pay For C”: the first ‘Pay For C’ is to pay for competency, that means, (to) pay for competency training funding for the family physicians to know how to look after the cancer survivor(s), and that will help partially solve some of our medical-legal concern(s).” | FGD#9, private |
|  |  | “It could be ***on par with chronic disease management***, like for every diabetic patient or hypertensive patient, ***the government gives hundred dollars per patien***t. Cancer care is similar to chronic disease management.” | FGD#30, private |
| Implementation resources to support shard care delivery across care providers | Constructing a comprehensive and concise survivorship care plan as a tool to facilitate care coordination | “I think it’s ***useful*** still to have, even though there may not be any recommendations on them, and we also know that sometimes we may not be able to do anything about it. If anything, it’s more so that ***we know what patients come back to us with, so that if they do develop new symptoms, we know whether anyone has heard of that before, and perhaps we can reassure patients*** that “Oh, (that’s) whatever you have.”, and we are not too concerned, as compared to something which may happen, new, and it wasn’t present on the discharge summary.” | FGD#40, public |
|  |  | “But it’s good to have it on the form here, so that the ***patients know that there are some of these things that they can actually discuss with the family physician***; because if it’s not there, they probably don’t think about it.” | FGD#10, private |
|  |  | “Maybe sometimes else they could add on (is) ***red flags***, maybe something ***to list what are the urgent things for which we need to refer back or call the oncologist (for)*** – that could be on another section.” | FGD#16, private |
|  |  | “I would be happy if it’s going to be ***summarized*** in the form of the stage of the disease, the specific treatment that is given to the patient, and what symptoms and the side effects the patient had, (like), so far, what are the side effects, the primary complaints and the secondary complaints, or the primary issues and the secondary issues, ***which of the issues we, as a primary care physician, have to address, and so far, which are the issues that have been settled and the patient is coping well***.” | FGD#31, public |
|  |  | “I find that the ***relationship works a bit better if there’s a physical piece of paper*** that is moving between me and the patient.” | FGD#10, private |
|  |  | “It has to be something that ***we can see upfront*** and seeing that “Oh! These are the changes that have been made already.”. ***On paper form, it might be very difficult***, especially every time a patient comes in, there’s something that’s updated, then they give you another new form and then you have to feedback.” | FGD#59, public |
|  | Explore enablers of information transfer across systems | “So, I think this ***flow of information enabled through IT*** (information technology) would be also very helpful for us, so maybe I would say that, if shared care (is to happen), once in a while, you (can) ***get a status (update) of what the specialist is doing for follow-up***, and maybe the ***specialist can also check on how have the patients been in terms of what they are doing during our usual routine follow-up at the primary care in the polyclinic*** (then) I think that would be useful.” | IDI#6, public |
|  |  | “When you ***facilitate the co-management by having templates***, by having ***information that is easily accessible*** between the two of them, then co-management becomes a lot more easier.” | FGD#16, private |
|  |  | “They’re thinking of ***using prompts to highlight to the doctor what are the due test(s) to be done***, for example, colonoscopy or tumor markers or BMD (bone mineral density test), so this will decrease the need for the doctor to remember what to put.” | FGD#59, public |
|  | Consolidate existing supportive care services to facilitate referrals made within shared care | “I think, the social worker, the financer bit; it could be ***one-stop and link up with AIC*** (Agency for Integrate Care), ***the whole conglomerate*** and things (like that), that will be useful. And of course, psychologists, psychiatrist - somehow, sometimes they may not be active, but somehow ***they must be in the network***.” | FGD#13, private |
|  |  | “I feel that we should ***rope in the ancillary partnership members***, such as the dietician, psychologist, social worker and physiotherapist – I think they all play a part and it’s all part of this cancer survivorship.” | FGD#62, private |
|  |  | “Or maybe even those with ***social plus medical elements to be under our case managemen***t, because we then address not just the medical problem, but if there are social issues compounding the medical care, ***we can then touch base with social agencies outside of the clinic*** to kind of sort those things out.” | IDI#6, public |
| Maintenance: promoting sustainable adoption of shared care model | Governmental support is important to promote sustainability of shared care through general education and subsidiary schemes | “…able to have ***some form of guidance that can be instituted as a national objective***, so that you are really able to transfer substantial part of that care for patients who (are) cancer survivors, or even during the time when they are actually going through their cancer treatment, so (it’s whether) the ***primary care community*** ***is scaled up enough and have enough community assets to help to manage these patients***.” | FGD#14, private |
|  |  | “For a pure cancer patient with one diagnosis, if, let’s say, he was able to tap on CHAS (Community Health Assist Scheme) for that, I mean, that ***budget to me, may be or may not be enough***. So, if the patient has cancer, diabetes, hypertension, hyperlipidemia and all that, the same CHAS is going to cover all these, then obviously it’s not going to be enough. So, ***maybe there’s a system that we also need to look at, rather than giving the same kind of budget to all chronic patients. The budget needs to be adjusted to the number of diagnosis that they have.***” | FGD#40, public |
|  |  | “… the current funding, in a way, does not really recognize the theory. So, I think MOH (Ministry of Health) is also looking at how to ***risk-stratify the patients***, to say that ***certain type of patients would need a certain type of care, therefore (to) be funded differently***.” | IDI#6, public |
|  |  | “If I were (to) refer all these patients that I see right now with lymphedema back to the physiotherapist, some of the hospital physiotherapists… are willing to see and they are happy to see ***but the administrative-system-related issue is (that the) backend top-up subsidy*** for these patients where this patient is paying $20 out of the $100 cost, the $80 bill will go back to the polyclinic, and ***we have to pay the $80 bill for the patients, but not the oncologists***, (then) our boss will come back to us, ‘Now, $80, count on my account or count on your account?’” | FGD#60, public |
|  | Reshaping survivors’ mindset to maximize their compliance in the long term | “…***patients’ lack of trust or thinking that the specialist centre is always better***. So, that may be one of the main barriers that we will need to go manage, because or else, the patients wouldn’t want to come anyway. And in the end, they will just come back to us to ask for referral letter back to NCC (National Cancer Centre).” | FGD#51, public |
|  |  | “Things like ***patients’ attitudes towards physicians***, trying to change that would take time.” | FGD#35, public |
|  |  | “I think with time, ***patients have also come to realize that the polyclinic doctors, or rather, primary care doctors are not just ‘cough and cold doctors’***. They are doing much, much more. And so, that mindset will change” | IDI#6, public |
|  | Sustainability could be achieved by integrating elements of the shared care model into existing clinic structure | “Rather, ***we should have (the) ability to risk-stratify patients***. (For) patients who need more care, maybe we can spend more time. (For) patients (who) don't need so many things, (then they get) maybe a shorter consultation time. Or ***maybe we can even get our nurse(s) or APN (Advanced Practitioner Nurse) to see some of these patients***, but when patients need the full medical review, then we allocate the time accordingly, then that will be very good, because then ***we tier the resource according to the care needs of the patient***.” | IDI#6, public |
|  |  | “***We just have to really start discussing and look at the care path***, and so that we get an appreciation (of) ***can I actually see this as a normal patient under the family physician clinic. If I can do that, then no problem***, because the family physician clinic is, itself, in a way, sustainable, even with the fifteen, twenty minutes intervention, no problem. It’s a bit higher (subvention) for patients registered to our general clinic. It’s a bit higher, because this are for complex, chronic patients focusing on diabetics… then it’s actually doable with the time that’s accorded.” | IDI#2, public |
|  |  | “And the ***institution must have ownership of the relationship***, like, ‘Yah, this is my partner. Must take of him for me. And this is my patient. I must take care of him. So, together, we take care of him properly together.’ Then, your shared care model will not perish because it’s built on ownership; it’s built on relationship; it’s built on ***responsibilities between the partners***.” | FGD#14, private |

Abbreviations: FGD, focus group discussion; IDI, in-depth interview
