# Supplementary material for: Implementing a community-based shared care breast cancer survivorship model in Singapore: a qualitative study among primary care practitioners
Source: BMC Prim Care. 2022 Apr 8;23:73. doi: 10.1186/s12875-022-01673-3 (PMC8991467; doi:10.1186/s12875-022-01673-3)
Supplement: Supplementary file 3 — Additional file 3. A compressed folder containing the raw data transcripts and demographics data collection form. [file 12875_2022_1673_MOESM3_ESM.zip › Supplementary Information File 3/FGD (07.16.2018).pdf]

<u>Transcript for Focus Group Interview 16th July 2018</u>

Key:

Moderator / Interviewer:	M1, M2
Respondent:	Participant A (A)
	Participant B (B)
	Participant C (C)
	Participant D (D)
	Participant E (E)
	Participant F (F)
	Participant G (G)
( ):	Paraphrases, additions to or rectification of grammar, vocabulary and/or truncated sentences.
[ ]:	Non-verbal, e.g. *[xx laughs] [pause]*
…:	Removal of false starts, repetitive or ungrammatical long phrases
CAPITAL LETTER:	When there is a louder emphasis or stressing on a particular word or phrase

M1	*[Participants take some time to settle down; 0:00 – 0:40min]* We will just start now. We'll just continue. Thank you everyone for coming to our focus group. *[Someone interjects, "How about the late ones?"]*
Likely M2	I think he's coming soon.
M1	It's okay. As an introduction, we'll go on to introduce yourself as Dr A to G. And then, we will discuss the first topic on background survey on your current practice. So, the question is, "Can you share with us some of your experience with cancer survivors?" Can we start from Dr G.
G	Okay, I'm G. I've had some patients who are cancer survivors, and they are coming here for follow-up on their chronic conditions and for screening as well. So, there are some (but) no (other) opinions about it actually.
M1	Thank you, G. And F?
F	I'm F. I haven't had that many patients who've been discharged from onco(logy) and then follow up with us, but a few. Again, it's just for monitoring of their chronic conditions and also to continue with the usual cancer surveillance.
M1	Thank you.
E	I'm E. I haven't received any referrals from NCC (National Cancer Centre) also. I usually see this group of patients for other reasons, and they usually wouldn't bring it up, perhaps because they feel that it's not the (right) place. Maybe they feel they

	have been adequately cared for already in NCC (National Cancer Centre). I see them for other reasons.
M1	Okay. C?
C	I'm C. I have shared similar experiences with G, F and E, but sometimes we have patients who have underlying cancer which presents with certain symptoms that may flag up to us as possible recurrence metastasis. So, there are some situations in the past when I do refer patients to external doctors, specialists in the hospitals, or ask them to get earlier appointments to NCC (National Cancer Centre), so that they can do a more thorough check to confirm whether it's related to an underlying cancer.
M1	Thank you. B?
B	I'm B. So, from my experience, what I see is that, normally, I would look after patients for their other diseases, like the chronic diseases, and they'll still need to control their diabetes and high blood pressure. But there seems to be a separation of the management because the oncologist will look after the oncology side, and I will look after the other side, and there's not much communication between the two of us. So, sometimes I actually have to look at the case notes to see what is happening on the hospital's side.
M1	Thank you. A?
A	Hi, I'm A. So, I mean, majority of the long-surviving cancer survivors are the breast cancer survivors who are generally in quite good health, the ones that I generally see. Actually, so far my experience is that they seem even more on top of their chronic diseases than the general population, because… they know that they have gone through a cancer… they are more interested in their health, I feel. So, actually they don't seem to be that poorly-controlled in their chronic diseases, that I've experienced anyway. (With regards to) management, I guess the ones we see are usually the ones that are discharged, cured from their breast cancer. They are quite well. I rarely see the ones that are still going to chemo(therapy) or radiotherapy. There ARE occasional ones, but not often.
M1	Okay, thank you. I think we have D. Can you share with us what (is) your experience with cancer survivors? Do you see a lot of them here, and in what context do you see them?
D	Is it general cancer survivors, or is it in particular, breast cancer survivors?
M1	Breast cancer survivors.
D	I think, occasionally. I don't remember seeing a lot. There *[trails off]*. Sorry, what is the question again?

M1	So, do they usually see you for the cancer problem, or do they see you for chronic conditions?
D	Oh I see. I think they usually see me for, like, either general, acute complains or their chronic conditions – not really for the breast (cancer) conditions.
M1	Okay, thank you. Let's us now go on to the second theme to discuss the perceived barriers of the proposed shared care model. So, the question is, "So, what are some of the barriers that you can foresee with this shared care model? You can discuss them in terms of patient-related, physician-related and healthcare-related system.". We open to the floor?
B	I think that – I'm B – so I think the difficulty that we may have is sometimes to understand the management that is going on, in terms of the treatment that is going on for the breast cancer survivors, because certainly, we are NOT very familiar with some of the drugs used, medications used, as well as the side effects that we are supposed to look out for. Another barrier I will be concerned about is the communication between the physician as well as the specialist. So, ideally, it'll be good to have an easy way to ask questions, because as we know, nowadays, it's hard to get in touch with some of the hospital colleagues. And by going through the general line, it takes a while and it may not get through as well. So, (we should have) some ready means of communication between the primary care provider, as well as the hospital specialist.
M1	How about other doctors? Do you all agree?
A	I'm A. So, I do agree I'll be worried my personal lack of knowledge in, especially in the medications used, the chemotherapy drugs used, and the side effects. But I think it's good that nowadays the information is all online, like the notes from NCC (National Cancer Centre) are uploaded online. Secondly, if there is more information on what to look out for, that is documented, then maybe when we see, we will have a better idea. Actually, I just remember a patient that I saw on chemotherapy. I think she was presented to me mainly for some infection, I can't remember what. Basically, she was in neutropenia and she was on chemo(therapy), so I think that's like occasionally some of the people that you may see.
M1	So, actually you do have a *[trails off]*. So, Dr A, do I understand that you do have patients who are actually on chemotherapy, who see you for their acute condition?
A	Rarely.
M1	Rarely so?
A	Yah. So, sometimes it can be confusing because, I mean, they are neutropenic, and they are having some infection which may be just a simple URTI (Upper Respiratory Tract Infection), but you know, for us, it's a bit worrying… because he's walking around in the community *[laughs]* but they are having neutropenia, so that part is

	a bit (like) I'm not sure how far to treat it. Do we just give him antibiotics? Do we just treat him like a cold? So, that's the part that is confusing.
G	I think it's also the communication between the oncologist as well. So, I'm Dr A. *[M1 interjects, "Dr G."]* Oh, G! Sorry about it. So, again it's the communication bit. if there's actually an open line or somebody we can actually contact, that will help a lot.
M1	So, if it's an open line, does that mean it must be specific to the patient, or it can be a general line?
G	Specific to the patients' condition, I mean, like say, for example, breast cancer or things like that. I think other barriers that I can think of (are) possibly the patient's perception on separating between the malignancy and also the chronic conditions as well, because sometimes they don't tell us about the fact that they actually are undergoing treatment or have undergone treatment. So, that's something about the patients' ideas on their condition.
M1	So, what do the other doctors feel? Because the patients generally have already accepted the two different management(s) from oncology and primary care, so what do you all think about the patient?
Likely F	You are still talking about barriers?
M1	Yah, barriers. It can be patient-related or healthcare-related.
F	I'm F. I do agree that I think our personal knowledge about cancer treatments, follow-up plans, side effects and things to look out for (et cetera), that's lacking, at least in my personal view. The other thing is that, the few patients who have been discharged from my care have brought a paper referral, so I try as much as I can to transcribe it into the "C-Doc" *[Reference to computer programme for recording clinical notes]*, but I think that, maybe going forward, if this plan was – I don't know – in the system as a follow-up or something, like for example, do the colonoscopy every five years or for breast, maybe mammogram every two years of something, if that was in the (National) Electronic Health Record, that would be great, because sometimes paper can be lost.
M1	Yah, I agree.
E	I think – Dr E – I think that plan must also be communicated and shared with the patient. They must be agreeable that they come here; the oncologist needs to make the patient AWARE of what their plan is, otherwise they come here and then they have different sets of expectations, which WE then have to deal with. Or they'll say that, "Oh! The specialist doesn't want to see me anymore.". You know, that kind of feeling.
M1	Yeah, that is true. So, do you think we need to seek the consent of the patient?

E	I think a verbal consent should be sufficient. And then, they should also have some form of folder that you GIVE THEM, and they know what to look out for, what lifelong screening should they have (et cetera) – something that they feel that they own and they can… take care *[laughs lightly]* of themselves better, take ownership of their own care.
M1	So, do I hear that the patient should have the ownership and that we are facilitating the process?
E	Yah, I think that will be a good thing.
B	B. So, one other barrier perhaps will be the investigations that we have to give, if we have to do any investigations, because at the polyclinic, we don't have the full range of lab services that is available in the hospital, so some are relatively basic ones, like sometimes calcium and magnesium (tests), sometimes we don't do it. We can't do it in the polyclinic, and also not able to be replaced (by other tests), for example, like magnesium. I'm just saying it as an example. So sometimes, if from the hospital's point of view, they may think that they have all these facilities, and in actual fact, they don't, so that will make it a bit more complicated for us.
M1	So, the hospital may not be aware.
B	Yah, that's right.
Unidentified female, possibly E	*[inaudible; 13:01min – 13:05min]*. They are quite expensive, because they are not covered.
M1	So, the cost is an issue as well.
Unidentified female, possibly E	Yah, *[inaudible; 13:11 – 13:13min]* actually.
Unidentified female, possibly E	Subsequently, the cost to patient for primary care is cheaper, right? So -
M1	*[Crosstalks]* – yah, I think that the main reason is that we think it's cheaper, so the patients would be best be managed at the primary care.
Unidentified female, possibly E	And the price of the mammogram, will that be under the screening or *[trails off]*?
M1	I guess in terms of pricing, it will be more like diagnostic, because you already have an underlying condition.

C	I'm C. I think one of the barriers that we may face in the polyclinic when we carry out this shared care programme is whether we have the resource, in terms of time and manpower. And because right now at the moment, we are already managing their chronic medical conditions, various aspects of their life *[laughs]*, so in order to give them more holistic care (and) also to cover the cancer aspect, we may actually need more time. And if they are seen in the general clinic, we may NOT be able to actually manage them very holistically. So, if we were to carry out this shared care model, then I think we may have to go into those second-tier clinics, the prevention clinics and so on, special clinics.
B	I'm B. So, I agree with C. The thing is that it's not just one department that is coming to us for this, you know? It's EVERY department! *[M1 interjects, "Yah, every department wants to decant."]* Yah, every department wants to decant, and we are trying to face, we are trying to manage all of these departments that are coming to work with us. And each of us, each department has got their own protocol, has their own way of thinking, their own way of management, and then we have to also try to fit in with them. And so, without any additional resources from our part, it just gets tighter and tighter.
A	I'm A. So, the other thing about the resources is that, so we screen on our mammogram and ultrasound, and even NOW, when I try to order an ultrasound for the patient in the polyclinic, I could only get (a slot for) next year January. *[M1 interjects, "Too long. A few months."]* Yah. So, with additional patients, it's going to get longer and longer in the meantime.
D	Sorry, can I clarify, this shared care for cancer survivors, it's which (stage), (for instance, are you referring to patients who) are cured, I mean, they are those with no residual disease, they have been followed up for a few years by NCC (National Cancer Centre) or breast clinic, and then, they have hit their five-year survival and they are okay, and then, they are then discharged *[laughs and trails off]*. I'm just trying to understand what is the model… WHEN they will come to polyclinic?
M1	Okay, sure, thank you, D. So, our proposed model is that we are not quite certain when is the patient confident or the polyclinic is confident of doing this shared care; it could be after active treatment, say, six months to one year, which I think most patients may not be ready. So, we're thinking of a timeline of, like, five years where they have already gone through multiple screenings (and) they know what to do, what to expect, and they may need a yearly mammogram. And then, they need the other aspects of care, that means, the comorbidities, like osteoporosis (et cetera). So, that means, basically, they are cancer-free, and the reason why we chose breast cancer is because it's a big population and because nowadays, (with) the hormonal therapy, we are going (on) to ten years. So, they do encounter quite a lot of side effects, which may not really be, you know, managed well in the institutional setting.

D	Oh, okay. Well, okay, my concern is, for patients-related factors or barriers, different patients have different awareness of their illness. I think it was brought up by E or F – one of them - that patients may not be so aware of their conditions, what sort of treatment, what sort of chemotherapy or hormonal therapy or radiation therapy that they went through. So, the patient factors, their own awareness of their own disease may not be there, and if they are not aware, and there is no *[trails off].* If they are hold up somewhere else, I mean (for) NCC (National Cancer Centre), at least we can see it's an obvious system. So, that may be quite difficult. The other thing is their expectations, the patients' expectations. In a tertiary centre, they are given a lot more time. They may even have a breast care nurse that they have a phone number to, and they can contact if they have any concerns regarding their prosthesis not fitting well, they can't find the – I don't know – the bra something wrong, because they have to wear a special bra on the prosthesis, right *[laughs]*, if they didn't do a *[trails off]*. Yah, so (for) all these things, our nurses are not prepared to do. We cannot field these questions. So, these are *[trails off]*. So, they will lose that assurance, I guess. And when they come here, it's SO fast, everything is so fast, they may not *[trails off]*. I mean, the expectations (have) to be there that it is not going to be the same kind of care. It's that everything in the polyclinic is done really, really quickly to manage all these. So, we may need some sort of teaching, in terms of the nursing aspects as well, or just in case we have questions like these. And then, (for) the physician-related factors, I don't know about the rest, but I'm not so familiar (with) the chemotherapy drugs and their side effects. So, I think we'll need education on that. We'll also need detailed memos of any side effects of the medications that may, like, (for example), peripheral neuropathy is quite common. Sometimes we just see peripheral neuropathy *[trails off]*. I mean, they complain of numbness and we do EVRYTHING, a battery of tests from B12 folate, thyroid function *[laughs]*, check (for) diabetes, then after that we realize, "Is this after your chemotherapy?". And they are like *[laughs]*, "Oh yah! Now that you mentioned it, it did!". So, it's much easier if we actually have a detailed memo that says that this patient has all these things. How long is the peripheral neuropathy supposed to (last)? Is it expected to last, (for) any complications, I mean. This is quite common, like peripheral neuropathy is one of the more common ones. Any complications that happened, any side effects that happened, how long are they going to experience it for, that we ALSO would know, because I don't know. And (for) healthcare-system-related (factors), they have mentioned it - resources. We have not enough time to really go through all these things, and I think in a general clinic, I'm not sure if the doctors would also have enough knowledge to THINK ABOUT all these questions. I mean, if you can THINK of these questions, at least you can google these questions. But if you can't even, like, you don't even *[trails off]*. Like, yah, you are training, I mean, the knowledge is not even there, you may not even think of all these possible questions that may come up, you see. So, I think there are barriers on every level *[laughs lightly].*
M1	Thank you for the sharing. That brings us to the third topic. Let us refer to this leaflet that you have. This is our proposed survivorship care plan. It's actually taken from

	a template by the American Society of Clinical Oncology. So, we want to gather some feedback about whether this paper care plan is useful, and what are the information that are missing, that you would like to have? I guess the first part talks about the cancer treatment. (Are) there too many details? Do we need all the information to appreciate what the patient has gone through, in terms of surgery, radiotherapy, chemotherapy? Do we need to know the drugs that they are on?
C	*[Pause; 21:42 – 21:46min]* I'm C. I just want to clarify: so (for) this care plan, the patient would have to bring along for every single visit to the doctor?
M1	Yeah, that is one issue, because I guess the care plan is given once -
C	*[Crosstalks]* – because they may not bring along with them *[laughs lightly]* for every single visit. *[M1 agrees, "Right."]* We do have a number of times when patients don't come with discharge summary. So, I mean ideally, like what F says, all these care plan(s) should be in our NEHR (National Electronic Health Record), so that everybody else can have a look and it should be quite, very simple, to at least give some brief guidelines on what to do and so on.
M1	So, I guess one proposal is to include it in the "C-Doc" *[Reference to computer programme for recording clinical notes]*, since we share the same computer system -
C	*[Crosstalks]* – I think the NEHR ((National Electronic Health Record) are actually coming up care plans, but it's just that they are taking quite a long time to actually come up with a new design I attended one of the requirement gathering session. They did mention that they are going to put (in) care plans, but I'm not very sure whether, I mean, every single department's care plan will be submitted.
M1	As you said, (for) the care plans, you want it to be simple. So, you know, and we also want it to be comprehensive. So *[laughs lightly]*, which are the areas that are useful?
C	*[Pauses and ponders; 23:04 – 23:12min]*, probably – I'm C – I just want to maybe highlight to the other physicians that are also seeing the patient, what are the pertinent things to look out for in this patient, what is the plan for this patient, you know, like, within the next few months. I think that will be quite relevant. The schedule for clinical visits, I think we can actually see all the patients' clinical visits with various doctors in NEHR ((National Electronic Health Record) already, so maybe that's not really needed. Basically, it's just a summary of what care the patient has been given, and what's the plan for this patient within the next few months.
M1	Yes, thank you. B?
B	I'm B. So, just looking at the care plan, I think some of it is quite useful, for example, the possible side effects of the various chemotherapy medications – that's the part we are certainly not familiar with. So, I think that's useful. So, I think when you say

	what will be useful for us is that what's relevant for our side. So, not too much info(rmation) but what is relevant for our setting. That will do.
F	So, just to follow up – sorry I'm F – yup, just to follow up on this: it's good to know all these, probably the more information, the better, because I don't know much, BUT maybe a follow-up as to what to DO if the patient HAS these side effects; what do WE need to do? There has to be a plan as well.
B	Maybe a contact number somewhere will be good as well.
Unidentified female	Those will be here, right?
B	Oh okay, yah, yah.
M1	Yah, but I guess the other thing is like if you want to include emails, whether the doctor physician is comfortable to give it to the patient. So, the amount of contact information also, we'll also need to have a look into it.
Likely C	So, the other thing is that this care plan is supposed to be dynamic, right? So, after every visit *[M1 laughs and interjects, "Yah! How do we update it?"]* with the oncologist, the things will be updated, right? So, they will be issued, like (after) every visit back from the oncologist, they will get a new care plan. Am I right? *[laughs]*
Unidentified female	Assuming that these are patients that are very stable already, I don't think you need to see them -
M1	*[Crosstalks]* – yah, it's like once a year.
Likely C	*[Resumes]* – once a year? Because sometimes we need to know what is going to be ordered for the patient. And if we were to order tests, we don't want to actually, like, order a similar test.
Unidentified female	If you are talking about test, are you talking about -
Likely C	*[Crosstalks]* – actually, it's quite easy if it's automated. If you've ordered the test, we can actually go into the system and see; probably just need to highlight.
M1	But if you look at the appointments, will the investigations be seen as well? *[A few participants reply, "Ordered investigations, yes."]* Yah, ordered investigations.
Likely C	I mean, what I'm afraid is like *[laughs]* if we give the patient various hard copies of this, they might bring the outdated version *[laughs; M1 replies, "Yah, that's true."]* You give them the wrong information!

M1	But I guess for the breast cancer patient, usually we don't do blood test. So, the evidence-based (plan) is just the mammogram. So, it's only if there's any persistent symptom, then we will do the full battery of tests.
Unidentified female, possibly C	I think ultimately, the patient needs to have some kind of ownership. They need to know what their condition is, and what is the supposed kind of treatment, what is the plan for them, because we are helping to facilitate care (and) we cannot be totally taking over everything.
B	I guess some of the tests, perhaps the more specialized ones, you COULD time it with the visit to the hospital and do it together at that time. For example -
Unidentified female	*[Crosstalks]* – MRI (Magnetic Resonance Imaging) breast.
B	*[Resumes]* - MRI (Magnetic Resonance Imaging) breast or maybe a bone mineral density (test), because we don't have that as well.
M1	I guess one group of patient(s) is for them who are on aromatase inhibitors *[hormonal therapy for breast cancer],* we do a yearly memo to have a bone mineral density (test). And if they are on Denosumab *[medication for osteoporosis]*, usually we require test(s) which are done six-monthly. So, do you think that these will be useful information to be communicated, so that the test (doesn't) need to be repeated?
B	Yes, but if it's six-monthly, because we don't have the BMD (bone mineral density), that has to be done in the hospital.
M1	So, in terms of the sharing of care, if the patient is being looked after for osteoporosis, do you think the primary care or the polyclinic doctors have the duty of care to look at the BMD (bone mineral density), or they can assume that it's really looked after by the oncologist?
C	I think the answer to that is whether we have the time *[laughs]; a few other participants laugh in agreement too]*, because if we have more time *[laughs]*, we'll definitely look at it and act on it. But if we don't have time, then I guess, I think most of us would assume that the oncologist will handle all these things.
Unidentified female	Yah, again, it goes back to communication. *[A few other participants laugh and agree, "Yah! Communication!"]*
F	I'm F. I think as long as I notice they have a follow up with NCC (National Cancer Centre), I probably wouldn't look at ANYTHING NCC (National Cancer Centre)-related. *[laughs; a few others laugh along]*.
C	*[Crosstalks]* – unless it's serious, yah, something abnormal, then I will try to look. Otherwise, I will just ask them to get early appointment or let the oncologist know (at) the next visit.

F	Yes, I agree.
M1	So, thank you, C and F. So, do I understand that if the patient has already got an oncology appointment, so we will assume that they are being managed that way. If there's any additional value-add that the doctor needs to know, it must be communicated by a letter and -
C	*[Crosstalks]* – yah, to tell us we have to monitor. And THEN, we will do it. *[laughs; everyone laughs lightly too]*.
M1	But would the polyclinic doctors assume the responsibility of the health promotion, in terms of smoking cessation, vaccination (et cetera)? Is that reasonable?
C	I think generally we would do that IF we have time. *[laughs and everyone laughs along]*
Likely F	So, you see the common thread now? *[everyone laughs]*
Unidentified female, possibly E	Because ultimately, I mean, if they have other comorbidities, I will engage it as prevention. But if there is anything related to NCC (National Cancer Centre), I will put a clear demarcation, you know, because usually they won't just have breast cancer; it will be plus other things.
M1	Thank you. B?
B	I was going to say that because this is not something that we'll see very frequently, so it will never be second nature, in terms of management, to us. It's not like diabetes where we have to handle day in day out, and we know everything that is to be done right at the top of our head. So, (for) this one, we may actually miss things because it's not something that we do on a daily basis. So, I think a good guide or something will help us manage these patients.
M1	Thank you. So, in the interest of time, we will go on to the fourth topic about explor(ing) some of the motivations for the participation in the shared care model. So, the question is, "What are some of the motivations to participate in this shared care model?" Or should there be a motivation or it's not necessary?
C	I mean, I'm C. It's more for altruistic reason *[laughs lightly]* that you want to provide more holistic care for your regular patients, then you may want to, like, look into every single aspects of the patient's life, psychosocial and so on and so forth. Otherwise, I can't really think of any other reason *[everyone laughs]*, when you are already like short (of time and resources).
M1	Yeah, because one of the thing(s) we hope primary care can do is to look into the psychosocial component, which tertiary care may not be in a good position to do, because they also don't have time *[laughs and everyone laughs along]*. So, would it be reasonable? Would it be unrealistic? *[laughs]*

Likely F	I think it'll be unrealistic. Sorry! *[laughs]*
Likely C	Actually, I think some of the psychosocial aspects can further be explored by, like, psychologists or even nurses who are trained, because -
M1	*[Crosstalks]* – so, it's back in the tertiary centre?
Likely C	*[Resumes]* – yah, because I think WE mainly manage the medical problems, and then we also look into what are the possible psychosocial issues, But if let's say we need to, like, ask people, then we really need to go into in-depth interview. I'm not sure if we have the time to do that.
M1	So, again, it's (about) the resources.
F	I'm F. I think the motivations, like C mentioned, it's a good idea on paper *[M1 laughs lightly]*, as in, a noble idea, but I think if we don't really take a really good, hard look at the resources and time, it's very, very difficult to put into play.
E	E here. I mean, you are just talking to us doctors here, but (for) the healthcare-system-related (aspects), the management, they need to, they should be one of stakeholders. We can only do so much in the five minutes, in a five-minute consultation with the patient, regardless of how noble or altruistic we want to be.
M1	Okay. That brings us to the next topic about stakeholders. So, maybe I can ask the senior doctors in the management here: … is cancer survivorship a priority, like how you do special programmes for dementia care. How do you *[trails off].* What makes you select *[trails off again].* How do you select which condition to be a priority in primary care?
B	I'm B. So, first of all, I think there must be impact. And these are the conditions that need resources, additional time and expertise to manage. Of course, we wouldn't get common conditions to be handled by a special clinic, because we really need to use the higher-qualified doctors and these are all precious resources to us. We have got to use it in a good way, as in, a meaningful way.
M1	So, (for) cancer care, would it be one of the conditions that you would consider?
B	I would think so. I mean, I think there is impact if we look into cancer care.
M1	Because if it's like one in three who is going to have cancer, and if there ARE going to be long(-term) survivors, we have got the patients who will actually have cancer.
B	Again, it boils back down to the resources. I think we all mentioned this again and again. So, each time we take out a doctor to run a special clinic, … the rest of the doctors have to handle his clinical load, so we also don't want that to happen. So, we don't want to pinch from one place, and then put it in another place, and then have the rest of the clinic suffer a bit more. So, again, resources have to be there.

M1	Any views from the doctors? Who else do you think should be the stakeholders that we can rope in to look after this group of cancer survivors?
Likely C	The community? Because I think there's a lot of resources in the community that we can tap on? *[M1 probes, "Can you give some examples?"]* Now, there's a lot of talk about engaging the community, to help the community nurses. I mean, if you are talking about palliative care. In this case, maybe not, because we are looking at the survivors. Right? *[M1 clarifies, "Yes, these are the well patients."]* Yah, (so some examples are like) Singapore Cancer Society and all these different organizations. They probably are able to offer some form of help. So, we can explore those areas.
Unidentified female	General practitioners?
M1	General Practitioners? Is it because they have more time? *[laughs lightly]*
F	F. You mentioned (about) psychosocial. I think we can tap on to the community psychologists as well, (who are) specifically concerned about psychosocial issues for these patients.
B	Can these patients also *[trails off]*. I mean, (I'm) just exploring other areas patients can be managed as well. So, can it be managed by nurses instead of doctors, that is more stable?
M1	Actually, this is one model, because like for example, (in) big countries, they actually don't stay with the oncologists, like Canada, they are looked after by the GPs (General Practitioners). And they are looked after by Advanced Practice Nurse(s). So, does the polyclinic foresee having Advanced Practice Nurse(s) to look after certain conditions?
B	We actually have Advanced Practice Nurse(s), but at the moment, because they are quite limited, so (there is only) one for each clinic. And they are actually trained to see where the greatest need lies, which is chronic care. So, they are trained for chronic care, to look after diabetes, hypertension and these.
Unidentified female, possibly D	Why not APNs (Advanced Practice Nurses) from NCC (National Cancer Centre)? *[M1 clarifies, "Sorry?"]* APNs (Advanced Practice Nurses) from NCC (National Cancer Centre).
Unidentified female	They are not trained. It's more like chronic care.
M1	Err, all right. I guess it'll stressful for them as well.
D	I'm D. I do think that, because right now, the communication between tertiary care and primary care is not as IDEAL as we would like it to be. So, perhaps, as in, because if let's say… I think if having the APN (Advanced Practice Nurse) in NCC (National Cancer Centre) is better, because let's say, if really there's any problem that comes

	up, at least they can communicate with greater ease to the breast surgeon or to the oncologist. They are in the same hospital; there is a better *[trails off]*. I mean, it's easier to communicate, whereas here, we don't have time *[laughs]* to make phone calls until the end of the day. So, I think because of current climate, I think it's probably (better), if let's say, the concern is for psychosocial management and osteoporosis, and it's like (for) specific things, then perhaps APNs (Advanced Practice Nurses) in hospitals can be trained to help with the tertiary load, because we're not yet in a climate where we can communicate very easily with the specialist in-charge. But yah, that's it.
M1	Okay, does any doctor *[trails off]*. Okay, so how about community resources? Any other community resources or even support groups? (Do) the polyclinic doctors also refer to them, Singapore Cancer Society for rehabilitation? You've come across opportunities to refer?
Unidentified female	What sort of rehab(ilitation) are you talking about?
M1	Cancer rehab(ilitation).
Unidentified female	Like, what sort of rehab(ilitation)?
M1	I think it's basically general rehab(iltation) but knowing that there are limitations, like Singapore Cancer Society, they have a centre with the gym facilities and all the instructors, I think *[laughs lightly]*. I think they rented an area in JEM *[reference to a shopping centre in Jurong East, Singapore]*, so they actually have a nominal fee of about, maybe less-than-hundred-a-year membership fee.
Unidentified female	It's like a gym membership?
M1	Yes! It's like a gym membership, that's right! And then, maybe they have support groups, they have talks, they have counselling programmes.
Unidentified female	I wasn't aware. Is it specific for breast cancer or for all cancers?
M1	It's for all cancer types.
Unidentified female	I think most of us are not aware of all that, that there are resources available for cancer survivors, for cancer patients.
M1	Okay, thank you. We've come to the end of our focus group. Are there any last perspective that anyone would like to share? Okay! We thank you all for your time. We can stop recording.
	[Audio recording ends at 39:49min]
