# Supplementary material for: Implementing a community-based shared care breast cancer survivorship model in Singapore: a qualitative study among primary care practitioners
Source: BMC Prim Care. 2022 Apr 8;23:73. doi: 10.1186/s12875-022-01673-3 (PMC8991467; doi:10.1186/s12875-022-01673-3)
Supplement: Supplementary file 3 — Additional file 3. A compressed folder containing the raw data transcripts and demographics data collection form. [file 12875_2022_1673_MOESM3_ESM.zip › Supplementary Information File 3/FGD (06.23.2018).pdf]

Transcript for Focus Group Interview 23rd June 2018

Key:

Moderator / Interviewer:	M1, M2
Respondent:	Participant A (A)<br>Participant B (B)<br>Participant C (C)<br>Participant D (D)<br>Participant E (E)
( ):	Paraphrases, additions to or rectification of grammar, vocabulary and/or truncated sentences.
[ ]:	Non-verbal, e.g. *[xx laughs] [pause]*
…:	Removal of false starts, repetitive or ungrammatical long phrases
CAPITAL LETTER:	When there is a louder emphasis or stressing on a particular word or phrase

M1	We'll have some introduction. Thank you for coming today. So, I will moderate the first part and leave *[colleague's name]* to moderate the second part. So, just (here are) some ground rules: we'll appreciate if one person speak at a time; that before you speak, please identify yourself as Dr A; and when we refer to (one another), we try to avoid names; and this will be confidential (and) all information will be deidentified. There is no right or wrong; just give us your opinions. Okay, so, when you go around with the introduction(s)… to talk about where you are practising, you don't have to specify your name, and you will also leave all your phones on silent mode. So, the recorder is on, we can start. So, thank you for coming to our focus group discussion today on community cancer survivorship. So, today, our first theme is that of a background survey on current practice, so I will… first let each participant introduce yourself, and maybe you can share with us some of your experience with cancer survivors. So, we'll start with A.
M2	Just put it on the table. No need to talk into it. Leave it on the table, can.
A	I'm A. I'm a primary care physician who work in primary care clinics. I also help out in home care, home-ventilated patients sometimes, and some RTC (Residential Treatment Centre) nursing home(s). My experience with their cancer survivor(s) (is) mostly opportunistic - they see me for either acute or chronic conditions, and sometimes, by the way, enquire about their overall health. Dedicated programmes are rare and far in-betweens.
M1	How about B?
B	Yes, I'm practising as a family physician in a typical HDB (Housing Block Development) estate. And I just do what normal GPs (General Practitioners) do – I see patients in the clinic, as well as in their homes, and occasionally in the hospitals. And with cancer survivors, there are a few categories - maybe one (category) is those who are in remission, but they come and see me for other things, like cough

	and colds, or management of their chronic disease(s. Then, there are some who actually have a relapse already, and they do come to see me once in a while for pain and all the other stuff. And there are those who are dying, that means I've got to see them in their home and then, let them - *[M1 interjects, "Palliative?"]*. It's hard to say whether it's really palliative, because most of the time, it's that they will call me to see before they die, so there's a time where I see (them) after they die, to sign the death cer(tificate). But usually before that happens, they want to engage me first to build up a therapeutic relationship first, so I will see them at that point. Then, at that point, we will decide whether or not they wish to die at home or in the hospital, but this is actually very close-to-death situation, rather than those whose survival is actually longer. So, we are actually talking about three to four weeks before they die, and that's the time where we'll actually get to see them.
M1	So, I understand that at least you get to see the whole spectrum of their survivorship journey?
B	If they happen to be my patient(s), then it's part of my responsibility, continuity of care (for) whatever issues they come (and) present with, then I just deal with it.
M1	C?
C	Hello, I'm C. I'm a GP (General Practitioner) in a typical HDB (Housing Block Development) estate. Same thing like most GPs (General Practitioners), I see mainly opportunistic cases for cough, flu, diarrhoea (and) things like that. Once, I think there are two or three patients who have chronic disease(s) as well, a bit of high blood (pressure), (high) cholesterol, diabetes, so we see that as well. And then, the oncologists and the oncology team will take care of the other issues related to the cancer, but usually we defer to their opinion. I also have a diploma in Palliative Medicine, but currently I'm not actively using it.
M1	Can I understand, firstly, whether you communicate with the oncologist?
C	(With regards to the) communication, (it's) usually not so close – actually we use letters mainly, should there be a need to write a memo or update to the oncologist. So, usually it'll be a memo update - Not frequently done, not frequent.
M1	D?
D	Yah, I'm D. I practise in a HDB (Housing Block Development) clinic (for) twenty-five years. I did a Family Medicine vocational training twenty over years ago, so there I was in medical wards, surgical, obstetrics (and) gynaecology, where I saw - and Paediatrics as well where I saw - childhood leukaemias. I did ENT (Ear, Nose, Throat) also, so we had NPC (nasopharyngeal carcinoma) cases. In fact, I helped to run NPC (nasopharyngeal carcinoma) (clinics). And then, I did National Skin Centre, where I saw some skin cancers. So, carrying the experience into General Practice, over twenty years ago, I guess there was not much collaboration, and like most of our colleagues here, I tend to see them for continuing care, and sometimes for chronic

	illnesses. What I notice is that over the last twenty years, there's more and more surviving cancers, especially breast cancers and all that, which I don't know whether is it due to early pick-up, so we've got an increase (in) cases. So, coming here for this session, I've been thinking about the cases I had, then actually it struck me that I have quite a lot – almost up to ten (cases), but five (cases) see me regularly. And we developed quite a close relationship, because they liked the rapport, and like B said, there IS a therapeutic relationship and it's quite special. They will share with you their journey. They'll share with you their ups and downs (and) the multiple surgeries that they have to go for, the multiple chemotherapy, and quite a few might even ask us for our opinions. So, of course, like C, we defer to the oncology physicians or the surgeons and we reserve our opinion, but it'll be good if we can have a more common understanding of what treatments they are going for, what are the opinions that they have. I mean, we don't want to replace the specialists. We are generalists, and our skillsets (are) more attuned for primary care, but the trust that (patients) have in us is so great that they REALLY want OUR opinion. And I'm forced to open the internet and google for the drug name and all that. And then, sometimes if (I've) got time and all that, I might even try to get more papers to read. But at the same time when we are doing this, we have an anxiety that are we overstepping our bounds? Are we acting in the patients' best interests when we say what we say, given the fact that we're not trained especially in this kind of area? And the patient, like B said, can be in the early stages, can be in the relapse stages, can be in the terminal stages. So, but we're always the ones to provide comfort and care. We are not the ones to provide cure. So, I guess (for) the palliative role, like C, I would love also to attend a course in Palliative (Medicine) and be certified for that, because I can see that THERE IS a role for us to play, but we want to be more confident. I want to be more confident when I'm advising the patient. And a lot of the psychological burdens actually come to us, because they say that (when) they come to all these specialized centres, everything is talking about biomedical (aspects only). If the surgeons talk to her, it's all about what is good. In fact, sometimes, they just tell the patient, "You got to go for this chemo(therapy). You HAVE to go for this chemo(therapy)." And then, she wants me to refer for a second opinion – a THIRD opinion even - and then, after that, we come back with THREE DIFFERENT opinions, and then, she comes and see me and then says, "Which one should I take?" You know? And then, she TRUSTS ME more than the other three, but she sees that I'm neutral. So, here, there is a role for us to play, but my anxiety is (that) "Am I doing the patient a service? A disservice? What is my role?". So, the psychological burden actually is quite big. And of course, if you want to talk about the rate of depression, all of them are depressed at some stage. You know? Who wouldn't be, you know, if you've got cancer? So, I can see that there is a role for us to play, especially for psychological care. But again, (it's about) funding and all that.
M1	How about E?
E	I'm E. I am a family physician working in a (heartland). Like many of my colleagues, I suppose, for survivors, patients in a survivorship kind of journey, I probably see

	them in a few cases: first one would probably be the diagnostic cases – sometimes we pick them up, we screen them and we refer to our colleagues, as in, institutions, to get confirmation of all the modalities, so that will probably be the portion that we will encounter. Second part will probably come as an adhoc basis, whereby patients come to us for cancer therapeutic-related issues, like, so when their chief physicians are not available, be it in the institutions or in the private sectors, that's where we come in to fill the gaps. And so that's where I find that I do play a part, a small part, in that. The third one will be (for) all the comorbidities (whereby) they have already been with us before, like (for) all the metabolic syndromes. So, these are the part(s) that, of course, when they come back, either voluntarily or as part of… some other conditions that (are) well-handled by our colleagues in their institutions. Of course, the other last-but-not-least portion would be when you see them at the last part of their journey. So, I think these are probably the few clinical cases that we will probably see in the survivor journey.
M1	Let's us go on to the second theme. The second part is to discuss the perceived barriers of the proposed shared care model. So, the question is, "What are some of the barriers that you can foresee with this shared care model? You can discuss in terms for patient-related, physician-related and healthcare-system-related (aspects)." May I invite anyone to share?
D	*[Pause: 11:39 -11:47min]* I can think of one. I'm D. I'm D. I can think of one: I think the patient(s), being faced with such a life-threatening disease, they may be wondering whether are we qualified sufficiently; are we sufficiently-trained; is the primary care physician sufficiently-trained to participate in the shared-care model. So, not ALL will be open to the idea. It's similar to the National Heart Centre DOT (Delivering On Target) shared care programme. Some invariably will want to go back to (National) Heart Centre. So, some would not trust (us). So, they must have some basis for believing that it's going to be, (firstly), safe, in terms of patients' safety.
M1	Any other opinions?
B	I am B. I think it's back to the same. I won't talk about barriers, but I think that at the end of the day, it's actually a doctor-patient relationship. And part of it means that we have to be responsible for the patient, so when the patient come(s) and see me, I'm responsible for whatever the patient presents with. And we have also this *[trails off]*. It's a double-edged sword. We are very accessible, so when we are very accessible, things can just come out of the blue when we just got to deal with whatever situation that happens, and it could be, like you mentioned previously, it could just be the adhoc kind of call, but sometimes it could really be an emergency, it's something that's serious that requires a referral back to (the) hospital. And of course, like what D mentioned, the keyword is still "trust". Everything is about "trust". Now, if patient had been with me, then the relationship is there. And she subsequently (has) cancer, goes somewhere and comes back to me, then that relationship is easier because I've been managing her. But if the patient should come out of the blue with a referral from somewhere, then it's a bit odd. I find it a

	bit uncomfortable, unless the patient is staying where I'm practising, and I see her children and all that, then that may actually ease the thing. So, at the end (of the day), it's whether the patient trusts me and all that, but the scary thing - whatever scary thing, whatever you call it, it's not really a barrier thing but – (is about) the accountability. Like, how much am I responsible for? But if the patient trusts me, as in, it's my patient, I just carry on and do whatever is necessary, but there's always this *[trails off].* How do I put it? If I manage my patients, I would always say that if it's a cancer-related kind of problem, your primary physician is still *[pauses briefly and laughs lightly; 14:28 – 14:31min]* that oncologist or whoever it is, or the surgeon or who is looking after (the patient), because I know, "Oh, you follow up at SGH (Singapore General Hospital) for this. You are seeing the chemotherapy for this, this, this. Are you still seeing them? If there's anything, you can bring up the problem with them." And if I think that it's something urgent that the doctor needs to know, then I just write down a memo, and then tell the patient to get an early appointment to see the doctor. BUT the stand (has) always been (that) the primary doctor is still the one that is really managing the thing, and I'm just here to support you. And that's how I would deal with the patient in the relationship, that means even though I'm your GP (General Practitioner) looking after everything, but when it comes to things related to cancer, I have a tendency to say that, "You know, issues like these (are) still best settled by the oncologist. I can settle whatever I can at this point, but (for) anything (else), you know, you should go back to your oncologist.". It's this *[trails off].* I won't say it's a fear but there are medical-legal issues involved in this because (it's about) who has the primary responsibility for the patient. But I think I would put this across to the patient, and I think the patient understands that if it's really cancer-related, your primary doctor will still be the oncologist or whoever, the surgeon or whatever, but I will manage you at this point of time, and then we will see how it goes. But it's how we deal with the patients and how we establish and make clear this clear relationship with them, and then things are a bit clear(er), because if they think that I'm the one looking after EVERYTHING, then there's a potential difficulty there. But I always, at the onset, I will tell them that this relationship won't be LIKE THAT. And they are quite okay with it most of the time. And that's how I see it. So, the barrier is trust. If the patient trusts me fully, I also get a bit worried. You know? So, it's how I temper the (expectations); certain things you can trust me to deal with it, but (for) certain things, I'm really not that knowledgeable or whatever, still. But once I establish that relationship with patients, then things are usually quite okay.
A	I'm A. I just want to clarify, for this discussion, if you are talking about cancer survival, STRICTLY, then are we *[trails off]*. There (are) a few definitions, because I can sense that in my interaction with some of my specialist colleague(s), whether it's radiation oncologists, palliative care surgeons, or our fellow GPs (General Practitioners), and even our patients, there's anxiety level at these three stakeholders – patients, specialists and family physicians. But if we are talking about, strictly, cancer survival, that means they are no longer on any active treatment, am I right (that) that's one definition, (then) I think, someone say five-

	year mark; some say three-year mark; some say where the prospect of the primary cancer return is very low, so IF that's the case, I think that can help lower anxiety for these three stakeholder(s) for a shared care (model), If I'm right on that. The second thing that I can see (, that will) enable us to come together to help each other, for the family physicians to help the oncologists manage the various biopsychosocial issues, and including comorbid(ities). Now, (for) diabetes guidelines, it's no longer in our 90s where it's just two choices of drugs, (but) now it's a matrix of… four by six choices, (which) is as good as *[laughs lightly]* some oncological therapeutic choices. … So, how can the family physician help the oncology team (in these areas of chronic disease management)? And how can the oncologist team help the family physician do long-term second CA (cancer) prevention follow-up? Just now D mentioned something about funding. There's some model already used in certain countries. The model is called "Pay For C", "Pay for Coordination". So, to do this, can I propose a two-framework of "Pay For C": the first "Pay For C" is to pay for competency, that means, (to) pay for competency training funding for the family physicians to know how to look after the cancer survivor(s), and that will help partially solve some of our medical-legal concern(s). And some of these competency training for – I'm sorry for the specialists, so - they will have to recognize when they have to consult a family physician to manage a comorbid(ty), because we've seen cases where the hBA1c (Haemoglobin A1c or glycerated haemoglobin; used for measuring control of diabetes) was (only) done every two, three years *[laughs lightly]*, and the same medication (is) unchanged and the risk is sky-rocketing! And "Pay for Coordination" means (that in order) to make up all stakeholders together, right, including the patients, we need a strong administrative support. And there are some papers to show that all these programme(s) actually save the nation health dollars, which I think the funder will be VERY HAPPY to look at. So, I think someone must overcome this inertia to overcome some of these barriers.
M1	Any other opinion?
E	I'm E. You would like to ride (on) the different parts of the patients', doctors', nurses' system? So, you've got to start with patients factors. So, I think, like (what) A has elucidated, the definition is very important IN THAT SENSE, so that we have some element of scope, because I think there's also this thing called "a(n) education at different levels". So, can we move from a disease-centred to a survivor, patient-centred (mindset), just like (for) any other conditions? So, patients must understand *[trails off].* I mean, understandably, (naturally when we are) old… we are diagnosed with a catastrophic condition, but we are quite fortunate, in the sense that we have very dedicated (staff), we have very advanced knowledge, (we have) very capable colleagues in the institutions, thus we have very good result(s), (but) they must understand (that,) going forward, maybe we need to focus on wellness, rather than the condition itself. So, (in) life, there are more thing(s) we need to look after, rather than just the tumour condition. Of course, with that, we have the cancer-related condition that we need to look after, the secondary condition, the secondary

	cancer, so to speak, because it's related to the therapeutic options. Take one chunk (out) that we need to cover up for all the comorbidities, just like any other (person), once they have (gone) passed a particular age. So, these are thing(s) that are educational, that at the patient level, they need to understand what they are going through. And second(ly), is of course to give them the assurance. Some of these patients, who have been very well taken care of, they also have built rapport with their oncologists, their surgeons, whoever is in the (other) institutions, so we should ENHANCE that. That shouldn't be replaced. The family physicians, if we were to come in, with the patients, it's JUST another form of relationship. So, it doesn't mean that they're going to be cut off, straight off. I think all our colleagues, we all agree on that. BUT there need(s) to be a bit of that transitional period. So, maybe a hotline, or something, I think, most (established institutions)… have coordinator(s), nurse manager(s), they have very dedicated clinic manager(s) to (help) have that continuum. So, I think we should keep that. And if we should have a phone number, not only for the patient, but also all those who are in the shared care programme, for all healthcare providers, healthcare alliances (et cetera), (they all) SHOULD BE on that little platform, that communication-wise, there SHOULD be a continuum, all the way until that day *[trails off].* It depends on what kind of masses (patients have). If you talk about colorectal (cancer), if you talk about that eighty-odd percent, probably the recurrence is (in) the first three years. Anything more than that, they are quite safe. But of course, for breast (cancer), it's different. It could be ten, twenty years back and she is still around. So, but I suppose that is nitty-gritty. We NEED to let our oncologists-colleagues do the so-called patient selection. I think initially we probably got to start with something more straightforward, maybe five years, whatever participants' age is (et cetera) – I mean, we are okay with that. But I suppose if you want to build up far… you need some brain-cracking and coming together. All right. So, these are things, the educational parts, the reassuring parts for the patients' level, so that they know it is just going forward, (that) you are not just lagging all behind. So, it's all (done) to ENHANCE their care, going forward. So, it's a lifelong journey. It's not JUST THAT TUMOUR per se, correct? Once they are, so-called, "has been managed", we move on. As far as the physicians (are) concerned, I think we probably don't see many of some of these cases as frequent(ly). They are not our "bread and butter (cases)". (At an) adhoc basis, we probably need to have some gap-filling portion, which need(s) to leverage on our oncologists, our surgeons, to see tonnes (of cancer survivors) over the years. Like some therapeutic-related or some platins-associated neuropathies, we don't see so much (of these cases). We know that Neuroxetin *[reference to a drug used for treatment of neuropathic pains and other conditions]* may be one (issue we commonly see), but we do need some of the reassurance, update(s), so for (that aspect), we probably need that patch of update to reassure our physician(s) that this part (is what) we can handle. But I think the rest, probably we are quite all right with the comorbidities. We don't have to burden our oncologists to manage the hypertension, (as a) by-the-way. So, I suppose that part is important. So, I think our (primary care) physicians also need be mindful where (are the) areas we asre going to cover, to be very clear, where is our ground and which is our strength, which is

	the ground that which we need a constant, so-called, update (on). So, like C has elucidated, that this course can be forever evolving, like it can be parked under CME (Continuing Medical Education) as an update, so that's another thing that we probably can think through. As far as the system (is) concerned, I think B and A have also already elucidate(d), so I probably will talk about some other thing. So, (like) funding, I think for that (aspect), probably, we all… understand about it. With money, a lot of thing(s), we can be looked (after). Of course, with the scarce resources, it's always a challenge, but I think we can manage it (in) small stages. So, (it's) whether we want to have a quick fix, or we want to have a long-term solution. If you want to have a long-term solution, I think policy makers need to come into play, because this is going to be a national thing, and it's going to be across clusters. So, if you want to pluck some low-lying fruits, it's available. There are some schemes already available, if they can just tap on, extend (to), for example, for the CDMP (Chronic Diseases Management Programme), chronic disease(s). If you can extend that, because this is common. So, we can help the patient who is really in need, in terms of financial status. The other portion about systems-related (issues) will be the financial, so-called, subsidy status. At each juncture, because of the funding models, patients need to stay in a particular institution because of the funding. We haven't come to a stage whereby funding go(es) with the patients. So, that is something that I'm not sure (if), hopefully, you know, we can go a bit higher up to the institution (and) will not be BURENED by a lot (other) good idea(s), which is already on the ground, but policy is not catching up on what we wanted to do. And of course, the other thing would be the right-siting. It's not too difficult for the institutions to right-site, but how hard (do) your colleagues, so-called, (have the) bandwidth to do it, because they are just as busy. So, do we have coordinators to work hard (enough) to right-site in the right place? What do I mean (by this)? If you want to decant to the right, so-called, institution easily, I think it's (a) no-brainer – polyclinic. Because the institution funding is there. The whole *[trails off]*. It is wealth. I mean, with due respect, our colleagues in the polyclinics… are doing (a) good job, BUT they may not be the regular physician(s) of the respective patients. But the patients HAVE to go to a particular institution because of, you know what? Because of funding, because of the system's structures. I even have patients who need to (be)… on survivorship… so-called, in the earlier phase of the survivorship programme, (which is) not such an established (thing) in earlier days and years. For colonoscopy, you need be (seen) three years to five years for surveillance. Is there a need to go back to the same institution to be seen by the doctors (just) because (patients need) to have that colonoscopy done under subsidised rates? So, this is a waste of resources. So, can we open up services, allied healthcare (services too), the whole fraternity, for the patients' sake? So, I think this could be another journey, another milestone, that we may need to conquer. So, there are some systems-related issues, which is very (pertinent) to OUR system. Right? I mean, we can learn a lot of lesson(s) from the overseas. It's something that is useful (and) I think we should learn, but if not, we probably need a hybrid – something (suitable) for our nation, our system and how we function. Can we pull (it) off? Because we are not talking about (a) revolution. Everybody is scared about that. I think I'm

	talking more about evolution rather than a revolution. Of course, it depends on what timeframe that we are given and what sort of resource(s) there are. And of course, do we… spend (enough time) to find who is the physician? Because sometimes you ask the patient, "Eh, you know who is the GP General Practitioner) that looked after all these?", you ask her, she will bring you that, but (she doesn't know what is) the name, sometimes (even for) the block (location), she just can't tell you. So, can you give the patient the time to go back and find out, then you rise up (to it)? And if the patient is covered by this physician, and if the physician is not within a particular cluster, can you just invite, just speak to the physician, "Are you comfortable looking after this patient?". If he is, (invite him to) come and attend our course. Go through the course, you are ready to go! Do we go hard enough to find the right physician to the right-site, or are we just going the easier way? You know? So, these are (issues) at the multiple level(s) that we might need to think a bit harder (about) and think a bit further (about), think a bit higher (about), and maybe to (implement). Thank you.
M1	Maybe we go on to -
D	*[Crosstalks]* – can I just add one more thing? I'm D. This is not for every primary care physician, so some people just want to do the usual General Practice and leave all these to the specialists and NOT get involved. They want to go home and switch off the phone and not have to attend to any calls and not have any PSYCHOLOGICAL burden. So, it's not for everybody. Some people are comfortable with the way things are. I mean, I'm talking about primary care physicians. So, I think it has to be a selected group who would want to take on the extra – I would say, it's a – burden of taking care of the cancer survivors.
C	I'm C. I think one of the main barrier(s) is communication. So, if there is shared care, then there must be good communication, so either you have a common platform to record certain key findings or electronic sharing of data - I'm not sure, so, one of those methods - to have good communication. It's a form of delegation, I think. So, the workflow, I mean, the participants must be clear what they are in-charge of. So, maybe sometimes I understand that there are protocols in the hospitals, so maybe for the more common symptoms, perhaps the patients can carry this file around, and then we can update, maybe not everything (but just) a few key findings, so (as) to keep the specialists in the loop. (Regarding) resources, I agree with E (that) it's quite important as well. And whether or not we need special drugs that they need to go back to (the) hospital (for) and things like that.
M1	Okay, so we'll just move on to the next topic: we want to gather some feedback on this survivorship care plan to facilitate communications planning. So, this is the template on the ASCO (American Society of Clinical Oncology) for the plan, which is given to the patient to actually bring back to the primary care physician. So, I'd like you to go through to see which are the areas that are useful, not so useful, or which areas that can be improved on? *[pause; 31:49 – 31:53min]*

A	Okay, I notice in the form - I'm A - triple-negative (breast cancer) is ruled out.
M1	So, in this, we are mainly talking about low-risk breast cancer survivors, so the triple-negative (breast cancer) tend to be the younger patients, tend to be related more to the BRCA hereditary type, so usually we'll like to keep them in the institution. So, it's mainly the estrogen and the hormonal-positive (patients) that TEND to be long-term survivors, but they also have the chance of (developing) long-term comorbidities as well.
A	So, risk management of these (patients are) at institution sites?
M1	Yes.
B	I'm B. I've problems reading all these things *[laughs and everyone laughs]*. But what I hope to see in one of these things that the patients bring to me is three components: (firstly), what is the routine; (secondly), what is the action for anticipated problems, and (thirdly), is this referring-back issue. Now, for the routine (cases), I just need the diagnosis and maybe the staging and all that, because in case maybe I need to sign death cert(tificate), I know what to sign them up for. Second one is (the) prognosis. Then, I'll probably need to know, at what level or what degree of alertness I need to (have) for this particular patient. And then, what is the familial risk, if they have a cancer that can actually (pose) some risks to the children or whatever, you highlight to me, then as a family physician, it's my job to make sure that the rest of the family members get screened and all that. But I suppose you all do the same thing in the cancer centres, but just in case, you know, I can always get them back to screen because "Your mother, your father have got these things. You know, it's probably good for you to go for screening.". So, the second thing is the action for anticipated problems - it's like the common things that they will complain of - whatever, I don't know what, bone loss or whatever – and then, roughly what to do for them, (for instance), reassurance, or maybe we can suggest some form of management, so that we know roughly what to do. And the third bit is the referring-back. (With regards to) referring, (there's) two (dimensions to it): one is sometimes you just need to call just for some advice, you know, a phone number or just email, you know, just a quick thing like, "This patient has this thing. Should I worry about it or just do a few things first?", that kind of thing. And the third one is the referral-back, when you think it's something that is a bit "fierce" (serious) already, and we need to send the patient back, then we'll send the patient back. So, these are probably the components that I hope to see in the form that the patient brings to me. And I think, probably, most of it, you have it here (already), I think. I mean, I see things like the "email" and the "telephone number", you know, the "diagnosis" and (et cetera). And then, the "side effects" is there. Maybe something (else) we can say to help us talk with patients, like say, for this one, "hot flushes" *[everyone laughs]*, or maybe you can prescribe patient something, or something like that, so that you know roughly what to do. And oh yah, even the

	"family" thing is here. Oh yah, okay. And this one is the thing you tell the patient when to see the GP (General Practitioner), so that's pretty useful.
M1	So, could I say that in this critical page, most of these issues can be managed by primary care physicians?
B	Which particular issue? This "bone density" one ah?
M1	Do they need some guidelines (on) how to manage, or can the primary physicians manage on their own?
B	There are a few things - because if it's written down, then it's easier for us to tell the patients what to do. If you leave it there *[laughs]*, then they say, "Why you order extra things?" "But you write down there, you know. You have to worry about this, you know. Your family physician can recommend a bone marrow density (test).", or whatever, then it's easier for me to refer (them) back and say, "No, I think it's better for you to get it done, rather than leave it up to us to (decide)."
M1	I think this is a very big part of Oncology, that there's no standard recommendations, right?
Unidentified male, possibly A	I think it's very challenging to separate functioning and insurance, because in our local context, it'll be tough (for) not just the community to manage, (but) EVEN at the (level of the) institution (as well).
B	(I'm) B. But it's good to have it on the form here, so that the patients know that there are some of these things that they can actually discuss with the family physician; because if it's not there, they probably don't think about it, but if it's there, they may want to say, "Yah, yah! I think this is something that I will have to talk to the GP (General Practitioner) about.". So, it's good to put it there. At least they know that these are some of the things that they can discuss with us; because without it, they may think that, "Aiyah I think it's all right." *[laughs lightly]*, you know? Yes.
E	Yes, I think - (I'm) E – I think for many of our GPs (General Practitioners), for us, we are quite pragmatic. I mean, we run a very busy clinic. So, it's very comprehensive, but maybe at the functional level, we probably will suggest if we can have a feedback… that maybe the first part is more (about) the practicalities, like what B was talking about, the ESSENTIAL things (seen at) first glance on one page. Then, the second and third part(s) maybe come BEFORE the important information, where we will browse through subsequently. So, I suppose this can be *[trails off]*. This information set to Number Ten is spread to the respective family physician who is participating in the shared care a bit earlier, so that if they have time, they can browse through, if they wish to. Because even if it's not (the case), let's say, (patients) come in late on a particular day, the physician can browse through the MOST IMPORTANT part, so this is the primary concern. All right? Then, subsequently, if (we) need any information, we can just go back (to it). Of course,

	now we do this, and subsequently, hopefully in NEHR (National Electronic Health Record). But I suppose this is all important information, which will comes in useful when we manage the patients (in the) extended parts, like (how) A (is) talking about when you want to know in detail, like some of the relative(s') habit(s), the daughter is growing up (et cetera), so all these question(s) will pop up. So, we hope to have a bit more information, so that when we give the right counselling, we are aligned with what is given. So, if we put the ticks on "genetic counselling" if you think that that has been done, I think most (patients) would not be missed. But just in case. If you think it has already been done, we just probably can check with (the) patient what has been discussed. If it is not (done), we can just bounce (this patient) back to the centre, whether it's "BRCA (Gene) 1, 2", whatever. So, these are things that would be useful, but maybe not so immediate in the first place, unless things come along. So, I suppose the checklist is quite useful for us to browse through, just in case we miss out (on) some of the parts. Of course, the rest will be (that) it's always feasible except, again, whether the patients can do it in the community (or the) private setting under a private rate, or it's (done in the) institution. So, of course that's a different ball game. Being able to do and whether, (with) patients' financial status, we will be able to perform (are) different set(s) of issues (and) challenges.
M2	Can I ask B, *[laughs lightly]*, if we have an electronic version of this and you take any of content as a drop-down of all the options that we can choose, action-able plans, a guide, will that helpful? That means if you think that if the patient has "financial advice", "difficulty", then drop-down, like (who to) call, what's the number, or who's the link, if it's the case that it can give us an electronic roadmap that paper format CANNOT make up with, what's the "hotline", what's the different "options", like, let's say, (with) "memory loss" and things like that?
B	This one, it may be discussed later on, but if you take my (earlier) point of view, at the end of the day, it's still (about) this relationship. I find that the relationship works a bit better if there's a physical piece of paper that is moving between me and the patient. Something electronic is *[trails off]*. I don't know! It doesn't build the relationship, so I rather have a piece of paper, that the patient brings the piece of paper, I do a few things, I pass it back, I think THAT builds that relationship and that trust, rather than, "I don't trust the computer" and the patient don't trust what I'm typing, you know? So, it's always nicer to have a piece of paper that moves, and it's the relationship that I find very important in most of these. So, it still has to be paper. It HAS TO BE paper. I rather have paper. I have VERY BIG reservations regarding electronic forms, to me, TO ME, because at the end of the day, the best person to coordinate care is ALWAYS the patient. And the patient must hold it and he's responsible for this. Then, that's where all the relationship(-building) happens already, because moving, touching and all that (et cetera), I think it should work better than things on electronic (record). That's my personal view.
M1	Okay, thank you -

E	*[Crosstalks]* – okay, I think I can understand where B is coming from, because electronic is always good to have, because the kind of advantage (it has), I think nobody would doubt… it. But of course, (regarding the matter of) taking the ownership and the responsibility, we haven't give(n) patient(s) the time for them to move around. So, sometimes we really need to involve them to take on the ownership. For us, we are (in a) supporting role. But of course, I mean, there are different group(s) of patients – some of them, they just let doctors do the job; some (patients) are not (like this and; some (of them) really want to participate, so these are the (patients) we want to encourage. And I think as the years go by, they are more educated, they are more informed, they will probably be (like) that, but we want them to take the ownership. So, I think of course this comes in different forms and different kinds of comfort level(s). But of course, don't just do away with papers, just because IT (Information Technology) is THE THING now. Of course, we want to save more trees, but it's just like palliative care, it also comes in folders, right? It also comes in folders also. So, any physician who is participating would have a common doctor, and the whole family, the whole healthcare (team), the chief carrier (et cetera)… have it, so they have access to it rather than (use the) computer (only). Of course, with the computer, at the end of the day, if they have a WHOLE platform (set up), everybody is (IT)-savvy, then maybe that day will come, but maybe (for now, it's) "akan dating" *[Malay; translated to mean "will come" or "yet to come"]*.
M2	So, just now you mentioned about more potable subsidies, so if it DOES one day happen in Singapore, do you think the ownership will go up?
E	Ownership for patients?
M2	When the subsidy becomes potable, they actually hold the *[trails off]*?
E	Why not? They should be responsible for their own health(s).
M2	So, if there's subsid(ies) with patients, cross the three clusters, across the private and public -
E	*[crosstalks]* – so, patient(s) would have the choice; they make the choice! So, it'll be efficient, effective healthcare, so patient(s) will decide.
M1	We'll just move on to the next topic: we'll explore some of the motivations for participating in the shared care model. And the question is, "What are some of the motivations to participate in this shared care model?". *[pause; 42:23 – 43:28min]*
M2	So, I just want to thank all of you for coming again. I think D mentioned earlier that this is NOT something that all practitioners would want to train on, so I suppose the fact that you guys are here implies that there's something above and beyond what you do on a daily basis, and perhaps you can share with us what are some of these things that has brought you here to spend your Saturday afternoon (doing this) in the first place? Free lunch? *[everyone laughs]*

D	I'm D. I would like to see the patient through the journey, so I would like to be able to take care of the patient until the end of his or her life. And also, (the) current model now is that once the patient has cancer or what *[trails off].* There's one concept that I like – it's called "capture and no release". You see (in) a lot of videos, I watch a lot of "youtube" (whereby) people catch fish in the "long kang" *[Hokkien; translated to mean "drains" or "canals"]* even in Singapore, and then they will measure it, and then after that, take a picture and then throw it back into the water. But our hospital system, at the moment - (and) it's not just (for) cancer care (but also) care (for) the heart, the kidney, you name it - all the specialist departments will capture all the patients and never release! It may not be their fault. (It could be due to) the funding, you know, or whatever; could be funding, could be patients' preference, could be trust issues and all that. So, the hospital(s) capture the patients but never release (them). So, no wonder you guys are bloating, you guys are *[trails off]*. (We read in) newspapers (that patients) complain of waiting time and all that, and we are, down here, you know, waiting for patients to see and end up doing coughs and colds, because there's no release back to us. So, it's a little bit like salmon, you know, swim to the area, spawn and then die there and they never come back until the next season *[someone laughs]*. Yah, we're waiting for all the foreign workers to come and give birth and they come and see you. All the Singaporeans know where to get cheap and good vaccination and all that. So, basically, I think I want to see the patient through this journey, otherwise you get disruption of care, and you don't see the patient, and the next thing (is that) they call you for terminal care. Maybe you take care of the patient for two weeks, and then patient pass(es) away. The last time they call you is to sign a death cert(ificate). I find that to be highly unsatisfying, you know, to be missing in the caring of the patient. But a lot of things, as mentioned in this very wonderful and complicated and very user-unfriendly *[laughs]* form is that *[everyone laughs]* it's (supposed to be) DOABLE by us. *[laughs]]* It's like I look at this list and I want to fall off my chair. It needs to be improved. I mean, it's all there, it's all there. So, I mean, you look at all these things, "memory", "parenting" (et cetera), this is what the patients come and talk to us (about) all the time, you know, "fatigue", "emotional, mental health", "anxiety", "depression" (et cetera), they all talk to us (about these) all the time, and "side effects", most of the time they will also come and tell you. So, I've got one patient, a young man with lymphoma that was treated here, he has cardiomyopathy, he looks very fit, and every time he is a bit "chuan" *[Hokkien; translated to mean "breathless"]* only, then he get(s) scared and comes to see me. He'll be frightened; he'll be shocked; he's a high-powered civil servant actually. So, I think this "capture and no release" thing is quite bad. So, I think it should be reversed. So, if the GPs (General Practitioners) keep shying away from slightly more burdensome care, then we are the only ones left to (be) blamed. But if you want to see the patient through the journey, and the patient(s), those who trust you, will ALWAYS walk to your clinic and ALWAYS talk to you, you know, whether it's (for) their cardiomyopathy or their peripheral neuropathy or their fear of the second cancer. Now they are all very aware, very intelligent. So, (if) you (are) sitting down there and you keep shying away, you will have trouble with all the patients who have trust in you, on the

	doctor-patient relationship, the therapeutic relationship. They will keep coming to you! And then, here you are, feeling totally incompetent and all that. I think that's not the way to end my career.
M2	So, (it) sounds like a long-term continuity of care from the start, all the way to the end, is something that you value in your care?
D	*[Crosstalks]* – yah, yah, yah. And I would even want to do the palliative part, for those who have already chosen us. It's not for everyone; maybe I can take care of five, maximum ten, fifteen of such patients. I cannot have like fifty to a hundred (patients), I think I'll end up becoming a subsidiary of the National Cancer Centre *[laughs lightly]*, and this is not the intention. The intention is to take care of those who already are your patient(s), like what B said, you see. It's the relationship that you already HAVE with them, you know. And there's so much transparency, there's so much trust, they even have my handphone and all that, you know.
M2	Are there any other input from other participants?
A	So, actually, even though C and D have attended the graduate diploma in palliative care, we find that there's very little opportunities for us to practise what we are being trained and taught to (do). The system is just lacking. I think our motivation for coming here, other than the free lunch that is offered by F *[jokes and laughs]*, is that out of the six to seven thousand family physicians in Singapore, there's about hundred of us who are fellows. We have very high Maslow self-actualization needs, all the way up there *[someone laughs lightly]*, so all the participants here are unique. Hundred divided by six thousand, is zero point what percent ah? But I think if you have one hundred family physicians fellows (who are) willing to help you, I think that's really a big step forward already, meaning (that), those here will be, on one hand, continuously be medical trainers, medical teachers, on the other hand, we are also medical learners. So, we are in a unique position to help, but we know that the thin (line) for us is training others and getting trained at the same time, it is *[trails off].* We're willing to sacrifice a bit, I think, BUT not at a tremendous loss. Just now, we mentioned about the need for communications, the need for competency training, the need for coordination - all these, one thing it means is that there is a time-cost to it. So, in a way, we are doing remote multi-disciplinary, interdisciplinary, TRANS-disciplinary coordination for the patients. Patients have to own some of it, but there's certain part(s) of it that (are) beyond the patients, (therefore) WE, as family physicians, specialists and colleagues, have to own part of it. And that will require funding, and I totally like the idea of some potabilty to that funding, like what E mentioned, but I don't foresee it coming tomorrow - maybe not even in (year) 2019 *[laughs]*.
M2	So, it sounds like YOUR motivations is then that of a slightly higher calling, BEYOND the fraternity and duties of a family physician?
A	And there's a lot of satisfaction in *[trails off]*. Just now C said, you have to choose the *[trails off].* C or B (said), choose the right family physician, right? From my

	interaction with specialists, it is that you also have to choose the few specialists that (are) interested, and once the match is there, it's a bit like successful thinking, the satisfaction is there, the professional satisfaction is really there. You get to learn from (one another), and you get to help (one another) and it's a good feeling. But how to find that match? Unfortunately, as family physicians, we tend to be the ladies-in-waiting – we are waiting for the knight to come to us and say, "we want to dance." So, we are waiting for an initiative to start SOMEWHERE.
E	I'm E. So, I think we want to come here to participate because we hope to make a difference. And we hope to also have the opportunity to be able to give our perspective, because, I mean, it's not the fault of our colleagues in the institutions – I mean, they do a good job and they try to do that - but sometimes we do see things from a different angle, we work in a different institution and different clinical setting and a different context. So, hopefully, we hear from (one another), and as C has elucidated, (to open up) the communication channel. I don't think there would be a perfect model, but it's good to evolve and we tweak as situation change(s) – not every day is the same. So, I think we can bear with an IMPERFECT system to start off with, but it's just a matter of time how can we jolt to make the system better as we go along. We MUST BE better as the days go by, because that's the only way that we can save…(and) stretch our government to the max(imum). Second thing is that (we shouldn't) be disheartened – in any system, in any project, there will ALWAYS be early adopters, any system, any project, projects, any system, because there are many people who have so many concerns (but) they may not voice out. This group is probably the most motivated group. So, don't be disheartened. So, sometime(s) I think when this is more established, it can be ironed out, the workflow, the operational processes (are) ironed out, people will come. I mean, it has been proven but it will take time. So, whoever is getting the funding, be a bit patient. I mean, if you want to change the technical direction, it takes time to move. It's not like a car, but I think we will see (it), we WILL be there. I mean, if there's a will, there is a way.
C	I agree with D and E as well. So, I'm C. So, I think this shared care programme is a vehicle to allow us to continue to take care of the patient, so that we can participate in their journey while they are being treated for cancer, which nowadays is regarded more or less as (just) another disease. Actually, now that you think of it, (for) most of the symptoms, most of us probably can handle. So, I don't see how it can be so onerous. Of course, for cases that are very difficult, we can have direct access back to the specialist. So, yes.
M2	So, what I'm hearing is that there is room for some complementing of a locus of expertise between primary care as well as the specialists?
C	I think it will also benefit the specialists because then they can concentrate on their expertise, perhaps… what they are specialized at treating the cancer and higher-level work, in that regard.

A	I think they are doing a very good job. I think all these common cancers (are) becoming similar to HIV (Human Immunodeficiency Virus) *[C agrees, "Another disease."].* It's like another chronic disease. And most of the survivors actually don't die from the cancer anymore. They die from the bread-and-butter metabolic diseases, which (are) our forte. I think there is a real complement, as you mentioned. And when they DO decant patients to us, we hope that there's some tidying up - they decant to us but the HbA1C *[Haemoglobin A1c or glycerated haemoglobin; used for measuring control of diabetes]* is not done, last cholesterol (test done) is thirty years ago, and then (they are on) the same med(icine)s, so I think this tidying up can start at institutions if you have family physicians WITHIN your institutions to stabilize the patient chronic comorbid(ities) before they decant. Your potential pool of family physicians can do the decant too - maybe not even just the fellows, but (for) the non-fellows, we can decant too. That means, not only the cancer itself is being very well taken care of, but the chronic (disease) is ALSO very well taken care of, then the barrier will be even lower. But the funding should be the same.
M2	I'm going to move on to the next question -
B	*[Crosstalks]* – can I answer for this (question)? I'm B. I still think the best doctor is the doctor in the community. So, (whoever) the patient choses to be his family physician, that will be the best doctor. And I'm quite wary about these courses and all that, because once you organize a course, then those who never attend the course will definitely say, "I just excuse myself.". But if we just take it as part and parcel of being a family physician to care for whatever problem(s) the patient face(s), the patient just comes with the letter and then, that's it. And the family physician will be quite happy, like, "I know, I know, I know what problem (you have). You bring me this form. This one, I'll look out for (it).". And then, if the family physician is uncomfortable, then all he needs to do is to have some number that he can call, to speak with a doctor. (It) doesn't have to the oncologist in-charge of the patient, but just some doctor to reassure the patient that, "No, we are starting this thing. Don't worry too much. It's just a form to inform you of patient's condition. And if the patient complains of these things, you can always call us too. And then, we can probably help you with it.". And so, what I'm wary about this course, because the problem with this course is people may not come. But if you just take it as part and parcel of taking care, you know, "This is the patient's comorbid(ties). You've got this, you've got that. I probably will look after this.". And then, you carry on with life. And of course, there are many different level(s) of training in the GPs (General Practitioners) in the community, but I'm QUITE SURE if someone has a Masters… I think they will quite comfortable, because they are so trained that they should be able to deal with it. Those GSM1 (Graduate School of Medicine Year 1), some are actually quite mature and they can actually just go ahead and handle, but some are a bit more apprehensive. And then, for those who are a bit more apprehensive, (we) just give them a little call, just to reassure them. Because (for) most Masters (holders), they just take a look at this, they probably know what this

	is all about, and you just manage the patients from there. So, I just want to put (across) this point that the best doctor is still the doctor in the community, and if the doctor is uncomfortable, then it is the patient's responsibility to find another doctor that's comfortable doing this thing with them within the polyclinic or neighbouring (clinic), or if too bad, (in a location that is) far, far away. It can't be helped. Or their sons' doctors or their daughters' doctors or in their sons' place, they will find somebody, and then (they) just need to carry this (form), and then *[trails off].* I'm not saying that there's no need for course(s), but I'm very worried, because with the course, a lot of doctors will say, "I never attend the course. I never do.". Or some will say, "Wah! I trained in MMed (Master in Medicine in Family Medicine). I train (in) so many things, still cannot handle this sort of things. Must attend another course.". You know, and then the patient will say, "Doctor, did you attend this course?". And then, they say, "No.". (Then patient may ask,) "Then, (you) can handle this or not?". *[a few others laugh]* Then, how to answer this sort of question? *[laughs]* So, because I've been talking to the other doctors, so this thing about courses is a bit *[trails off].* Just take it as part of your doctor's job, and… the GP's (General Practitioner's) job, and then, if you are MMed-trained ((trained in Master of Medicine in Family Medicine), then they'll probably look at this (and think), "This is my job. I know what to do already. If I'm not sure, just give me a number to call, just to talk to a doctor. And then, just reassure a bit, what is this all about, and then, it is good enough.". So, this is another good point about this.
M2	Let's move on to the next one, which is (about) relationships with the stakeholders. So, what will you think are or SHOULD BE the stakeholders that are involved in the shared care model that we are talking about? Maybe you just answer that part first, "Who are the stakeholders that we think should be involved?". Obviously, primary care.
C	Some days, we talk about multidisciplinary team – I'm C – so I would presume that the stakeholders would include the GP (General Practitioner) in the community nearest to the patient, (and) also the paramedical, allied health staff, nurses, in case of wound care or dressing issues, physiotherapists maybe, occupational health (therapists), speech therapists (et cetera), the standard MDT (multidisciplinary team) in hospitals is what I would expect. I mean, in a way, we can view the GP (General Practitioner) clinic out there as just a consultation room outside, so it's a team. It's just happens so that their consultation room is not in the hospital. So, but that would mean that the allied health team will have to be quite mobile. Perhaps even THEY can link up with the local resources and maybe the nearby centres. I think, like NTUC (National Trades Union Congress), they (have) got some gym thing, a lot of things. So, of course the hospital doctors would be in this team as well. I was *[trails off]*. Of course, then the next question is, who is the leader of the team? So, it could either be egalitarian or maybe the specialists could lead in the acute phase of the cancer or something like that, and once the cancer has remitted, then the

	leader is (the) GP (General Practitioner) or something like that. depending on the stage of (the) disease.
M2	Are there other stakeholders that anyone else can think of?
E	I agree with C. But of course, probably we can establish to a bit more. And if there's anything to add on, it would be, like, we've already covered healthcare alliances. I think, never don't forget the patients, and even the caregiver(s). I think some went down and they can also be tapped (on), because we must also see things from their perspective. I think that also cannot be under-estimated. Of course, the healthcare team, I think, it's a humongous team, I think C already elucidated. So, of course, I think, the social worker, the financer bit; it could be one-stop and link up with GIC (Agency for Integrate Care), the whole conglomerate and things (like that), that will be useful. And of course, psychologists, psychiatrist - somehow, sometimes they may not be active, but somehow they MUST BE in the network, and of course the coordinator, the clinic manager, all (of them). So, everybody contribute(s) a bit, it will be a bit more manageable, because if we want to scale up, we need their capacity, we need their capabilities. So, I think if you ask me *[laughs]*, I think even policymakers should also have some hearing about it. They don't have to come down but they must at least be within reach, so, these are the so-called micro level and macro level. So, I think, basically, quite a bit (of them can be involved). Of course, probably the phone calls can be an extended part of that thing.
C	I was wondering about the role of case managers, which could be nurse clinicians, because sometimes doctors… can be quite busy, so then, the nurse clinicians will be monitoring where the patient is being sited. I wonder if that is possible.
A	So, I'm A. I totally agree with C. So, I have Rheumatological care, I have COPD (Chronic Obstructive Pulmonary Disease) ICP, "Integrated Care Plan". And if we talk to some of our private practice fraternity who visited, a lot of this shared care start with a bang and fizzle out with time *[laughs lightly; C replies, "True."]*. The key thing is a good case manager. *[C agrees, "Yes."]* or a care coordinator, ideally with clinical background – actually, (on) that end, (they can be) senior nurses. Hopefully, all the nurses fulfil these needs, and with some basic training and exposure to mental health, psychotherapy and social work. … And usually, these case manager(s) will be in their own institutions, but the CHALLENGE is for the institution to ensure MINIMAL handover. The moment the turnover occur(s), the link is broken, so that is the tough part, because they require someone who is trained, committed and willing to be mobile. It's quite difficult. It's quite difficult. So, case managers are important. That's the compelling truth.
C	It's true, you know. Some of these programmes, they fizzle out after a while, so it's very hard to keep the fire going.
A	They will be in our clinic and they'll hold the patients' hand, or AT LEAST, for the first visit, and then, (for) regular visit(s) after that. It's actually quite tough for them. So, they should be remunerated well, but that also (depends on) whether the

	funding *[trails off]*. The funding that I witnessed is usually time-based, that means *[trails off].* But you mentioned (about) the continuity of care, right? So, the funding has to be continuous (also). Most funding are located by institution because of policies and are TIME-limited, but patient usually survive BEYOND that funding. So, what happened? It goes back to the institution. And you find that it's even harder to manage, because there's a time lapse, blind spots, blackhole(s) of three, four years, and then you come back and we restart. That even more expensive then, the time-limited funding.
M2	All right. So, (for) the second part of this question, what are the potential barriers that may affect communication? So, (this refers to) communication between all the different stakeholders, so what do you foresee are the barriers that can affect communication and similar concerns?
B	I'm B. And I always say that the only stakeholder is actually the patient *[everyone laughs]*. And the patient is the first person to coordinate his own care *[everyone laughs again]*, so all you need is the patient to bring around this piece of paper wherever he goes. And then, we communicate on the same piece of paper, rather than I give you a memo, then you file here, file there, and don't know where it is. It's just a file, I can write a few things, then you write a few things, then at least there's some continuity in that document; because if I write you a memo, I can't remember what happened to you already. And you know, you probably file the memo somewhere and then everything is all forgotten. But if everything is written on some docket or something, it's all there. Everywhere he goes, he just brings this docket everywhere and the communication is all there. Whatever I want to write, I just write down. You know, if I don't enough time, I don't have to write. If there's something I need to tell somebody, I just write it down. Of course, the electronic record is still the best, but I STILL think the paper is important – it's like a form of handing over, I hand you back, you hand to me, rather than, electronically, there's no -
Unidentified male	*[Crosstalks]* – it's like a symbol.
B	*[Resumes]* – ah yah! A symbol! Like you hand to me, I hand to you. That's important!
M2	It's some form of documentation that help, the communication goes on?
B	Yah, and then we can communicate based on that, so all the information is documented. But of course, if you want to make us type again or make us write, also can, if you want to do it that way. But I still think that you still cannot rely totally on digital. You need that symbolic thing.
C	I'm C and I kind of agree with B. I was told that in India, they bring their case notes wherever they go. Of course, the question is whether our locals will misplace their

	file. I don't think it's uncommon to hear of people misplacing their health booklets, so *[laughs and trails off].*
Possibly A	The probability of them losing it is higher than losing their handphone. They treat their handphone more *[trails off]* -
Unidentified male, possibly B or C	So, maybe a handphone app? *[laughs]*
M2	So, what are the barriers you can think off, that hinder communication, between, for example, specialists and GPs (General Practitioners), patient (et cetera)?
Unidentified male, possibly B	I think the word is "busyness", because we can be quite busy that we forget to communicate. I think that's why probably the care coordinator might actually be quite important.
E	E. Maybe a few things, one step *[trails off]*. Time, obviously it's time; then, the other thing is platform. Everybody is on a different platform, so if we can have options, that will be good. How can all these options be linked together, come back to the same group, administration, it's another devil to crack. And of course, the other one is of course the *[trails off]*.
A	Maybe, in the business world, there's this thing called the "ommi channel", "o-m-m-i" *[a multichannel approach to sales that provides customers with a seamless shopping experience, whether they're shopping online from a desktop or mobile device, by telephone, or in a brick-and-mortar store].* That means, one touch point, then straightaway, you can access multiple platform(s). And it's cost-savings for them, so (there's) nothing wrong with the medical institution(s) taking the "ommi channel" approach, and that means, in this case, it can become a form of a real person, a case manager. So, I find that a good case manager has some clinical experience with one of the institutions. It remind(s) me of my ward rounds, so as a clerk, I mouth off what are the action point(s) to treat for, the case manager take(s) note and follow(s) up with the SOC institution (Specialist Outpatient Clinic) UNTIL the case manager resign(s) and change(s) *[laughs and everyone laughs too]*. Then, that thing is lost again. But THAT hinges on one person. I don't know whether there's a better system, a better platform for this continuity of care, continuity of communication and there's no hiccups; because I think it's really impossible for the patient and the caregiver. Unless the caregiver is a fellow physician, to understand some of these complex choices and all the medical lingo and the *[trails off]*. So, I guess it's a co-ownership on patients, caregiver(s) and physicians, and really to oil it all - either it's a good platform or a good case manager.
Unidentified male,	Actually, we almost forgot (about) the primary care network. I'm not sure how that would fit in at the moment, but there is something.

possibly C or B	
A	It's SEVERELY under-funded.
Unidentified male, possibly C or B	Yeah, I think so.
A	SEVERLY under-funded.
B	I am B. I got this idea, that what someone was mentioning: it's actually something that it is outside of the medical world, something that is private between health professionals only – because when I log into patient's case notes electronically, I can actually enter this (page that) is only seen by health professionals, and it shouldn't be part of the case notes and shouldn't be held medical-legal(ly) (accountable), whatever we see in there. So, if I want to talk to whoever I want to talk to, I just type and then we just type. So, if I need something, I just go in there and refer, but it's not part of the case notes, and it shouldn't be used to "chou chou" *[colloquial slang; translated to mean "disturb", "bother"]* us, medical-legally. It's just opinions of health providers looking at each other's "what's going on, what's going on, what's going on". Once it gets put into (the) case notes, the whole thing gets very, very scary. If only we have a platform that is separate from everything. It's like a phone call, except that this one is actually a recording of our phone call. And then, that would actually help sort of tidy up everybody. We don't type or write, because medially, legally (there will be) a lot of issues, but if we have a separate platform, and since we have no time to talk, we just type, type, type and then we just read, read, read. And it's easy that I can just click and I can go in there and look. But this is only for health professionals only, and it shouldn't be used for or against (anyone) in the event of any medical negligence. So, that's the little thing you mentioned about the platform, that would be quite useful.
E	E…. So, time, (having a) platform, and the other one is simplification, make things simple. If (it's) too complicated, I think everybody would be bogged down. So, I think that's another thing, if you can. If we can do the best that we can. And to respond to C, I think PCN (Primary Care Network) is something (in its) infancy that is coming up. I think, like A has elucidated, it's under-funded. It's not easy - but of course everyone has their own reason - but it can be a double-edged sword too. Why? If PCN (Primary Care Network) thing, it can end up as a clustering thing. So, it can move very fast, but it can also hamper, you know, if the patient is not in my cluster. So, you've got to try it in the way that (is) in the patient's interest, so across, borders. So, we are a NATION; we are not (segregated) cluster by cluster, so can we utilize, maximize, again… our nation dollars.
M2	So, it sounds like artificial borders (when) referring patient for care?

E	I mean, you want to *[trails off].* That's why we were saying, if you want to go FAST, you want to go alone, so, maybe, (with) just your own group, small team. If you want to go FAR, we have to go as a group.
Likely A	So, I think, (for) E, what he mention(s) is very correct. From what I know about from some senior management in an institution, twenty to thirty percent of our chronic patients cross clusters, and YET, there isn't a trans-cluster structure for the operation, at that level. And MOH (Ministry of Health) is somewhere, but come to the institution, it's nowhere. Then, the second thing would be *[trails off]*. I lost my train of thought. *[laughs]*
M2	Okay, never mind, let's move on to the last question, which is (about) the community resources. So, who are the community resources available and who can we engage and involve for effective shared care? So, this ties in a little bit to the stakeholders question that we have earlier. So, some of you have briefly mentioned some of it. Are there any other community resources that you thought of?
Unidentified male, possibly B or C	Recently, somehow I managed to attend this FSC thing, "Family Service Centre", one of their event(s). It's led by one of our primary (care) physicians. So, I thought I was quite impressed, because they have case managers I think, and they also deal with all kinds of family issues, and they also have a mental health wing that goes into the community, house visits and all that. So, I think they are quite present in the community, literally. So, maybe they could be one resource.
Unidentified male, possibly A or M2	Are they funded by MSF (Ministry of Social and Family Development), MOH (Ministry of Health) or charity organization?
Unidentified female, possibly B or C	Err, that one, I'm not sure, but it's an FSC. (Family Service Centre).
Unidentified male, possibly A or M2	So, there's case management demand?
Unidentified female, possibly B or C	They've got a fair staffing level… and they are in their exact locality in the community. So, maybe they could be someone you could tap on, because they can do mainly more than just mental health, and social problems of course, but that would mean that they need a lot of resources – much, much, more.
A	So, (in) Singapore Cancer Society, there's a lot of charity doctors and a lot of experienced, dedicated volunteers, And the volunteers that I can think of is, why not we start a virtuous cycle (of) cancer survivors helping cancer survivors? We talk

	about case management right, I don't know whether can cancer survivors be trained, to be accredited to train, to support or to be volunteer coordinator(s)? They have journeyed through it, so (they have received) the same message through us, so you are nurse and I'm sure you'll understand but if you are an ex-cancer-survivor or a fellow cancer survivor, and you are now helping me to coordinate, and with cancer care improving, I'm sure your cancer survivor numbers are increasing, so (with) all these, there's definitely a pool of potential volunteers and potential future employees to be trained to be care coordinators.
E	Actually – I'm E – so, I think in fact, in the line of the survivorship horizon, in fact, there is also this component called "returning back" too, right? So, we can keep that in mind, so that would be in line with A (talking) about if we get the survivor as his own career and (to) give back. Because (in) healthcare, we need those (looking for) second career change. So, this will be a good source because they went through it and they walk the talk, so (it's) more convincing for them to come in and then, to be part of this coordination part, because they lived through it. So, that's one part. The other thing is also I think there's a rehabilitation centre – I'm not sure whether you all heard about (it) – there's (currently) only ONE, so if we have MORE coming up that's free, FOC (free of charge) for them to mingle themselves -
Unidentified male, possibly A	*[Crosstalks]* – the one in Jurong ah?
E	Yah, Jurong!
Unidentified male, possibly A	*[Crosstalks]* – charity dollar(s), so I think they are more of a charitable (group).
E	Yes, they are. So, we just need to tap (on them). They are already there, but we just hope that they are not overwhelmed. If they have a lot of satellite (centres) around the whole country, then there's another better one *[trails off]*.
Unidentified male, possibly M2	So, you mentioned (about) returning to work. Are there resources in the community that can help patients, in terms of looking for a jobs and all that?
E	That is not an issue, depending on where you need them. If you need them to do that part, I would want to train them, give them the first cut, if you want to be in healthcare industry. Then, WE should grab them rather than refer them (elsewhere), otherwise it's the Singapore national career thing. You can engage with all the respective medias. It's not an issue, but (it's about) whether we want them first, because THAT is what we want. This is the additional value that come with them.

A	Maybe who can help you is social enterprise and unions. I think E is absolutely correct. For occasional needs, (this) can actually make it work.
M2	All right, thank you so much for this session. I’ll stop the recording now.
	[Audio recording stops at 1:18:27min]
