# Supplementary material for: Implementing a community-based shared care breast cancer survivorship model in Singapore: a qualitative study among primary care practitioners
Source: BMC Prim Care. 2022 Apr 8;23:73. doi: 10.1186/s12875-022-01673-3 (PMC8991467; doi:10.1186/s12875-022-01673-3)
Supplement: Supplementary file 3 — Additional file 3. A compressed folder containing the raw data transcripts and demographics data collection form. [file 12875_2022_1673_MOESM3_ESM.zip › Supplementary Information File 3/IDI (09.19.2018).pdf]

Transcript for IDI 19th September 2018

Key:

Moderator / Interviewer:	M1
Respondent:	A
( ):	Paraphrases, additions to or rectification of grammar, vocabulary and/or truncated sentences.
[ ]:	Non-verbal, e.g. *[xx laughs] [pause]*
…:	Removal of false starts, repetitive or ungrammatical long phrases
CAPITAL LETTER:	When there is a louder emphasis or stressing on a particular word or phrase

M1	*[takes time to settle down; 0:00 – 0:16min]* Hello, good afternoon. Thank you for attending today's focus group discussion. May I invite you to share on your background about your current practice about your experience with cancer survivors?
A	Yes, my name is A. My name is A and I'm from (a polyclinic) *[omitted for reasons of confidentiality]* and I'm a family physician there. So, some of the cancer survivors that we see are usually patients with, like, resolved from nasopharyngeal (cancers) NPCs, breast cancer, colorectal cancers (et cetera), those even with like stoma bags. And a lot of things they come for are like follow-up from their chronic illnesses. The others sometimes… come in for their, like, simple cough and flu or diarrhoea. Sometimes, they come in for fever and they get very scared, especially those who've had, like kind of like, had haematological malignancies, they get very scared. Then, those who are in some form of chemotherapy also come through to us when they have fever. So, we do see a large selection of cancer survivors, but they come in usually not for their cancer stuff, but usually for the other things like the other chronic illnesses or the other acute issues.
M1	Thank you. So, from what I can hear, you do see a significant number of cancer survivors in your practice, but they may not come primarily for their cancer, but for other comorbidities.
A	Correct.
M1	I see. So, we were discussing about this proposed shared care model. So, what do you foresee to be the barriers of this shared care model, and if you can help us with some proposed solutions, we can discuss them in terms of patient, physician or healthcare related barriers?
A	I think doing shared care will be an ideal where we actually know who to contact. So, sometimes we get these patients, and we really are not sure what happened to them and whether it is a high-risk cancer or it's going to highly recur, so we're not sure about… what kind of prognosis they actually have. And I think sometimes what

	we really worry about is (that) if you want us to follow (up on) that part, what do we have to do? So, because we don't have that much experience, and especially in oncology, (it's) something that's always developing. There might be new things that we're not really sure (about), we may not be really up to date, so learning more about it would be quite useful, like what type of treatment there are. And I think… sometimes when patients come into the polyclinic, they just come (as) part of the general pool, and if they come in with the general pool, you don't know whether they've been seen by someone who's a senior doctor or is it a fresh MO (Medical Officer), so then you have a great variety of what kind of services you are going to get, what kind of consultations you are going to get. So, I think, (for) this kind of cases, you have to be very careful, like where you are going to send them too. In polyclinics, especially in (our polyclinic) *[omitted for reasons of confidentiality]*, we do have team-let. We just started it. So, (with) something like that, if you are told to go to a particular team, which at least there is a FP (family physician) there to know what's happening and at least has the experience and knowledge to guide through the patient while they are in polyclinic.
M1	That's a very interesting sharing. We've heard about the team-let model. So, is this team-let model only for (the) management of complex diseases or is it for everybody?
A	So, I think we are still trying to establish what each team-let wants to do… What our team is kind of trying to achieve at the moment is trying to get a pool of various chronic patients in from the diabetics, the hypertensive and basically your thyroid patients, and basically, it could be very simple (cases) like every six months, they're someone (whom) you need to see… quite regularly, like your COPD (Chronic Obstructive Pulmonary Disease) patients, so those who have been discharged, or those who have, like, heart failures and are trying to manage, titrate their medications, so there is a great variety in terms of complexity. So, we have two doctors and a nurse and basically a care coordinator, so among ourselves, we see a variety of patients, so it's not just complex cases.
M1	So, does that mean that (for) this team-let, there is a lot communication between the family physicians and the other team members?
A	Yah, so basically the way we're set up in (our polyclinic) *[omitted for reasons of confidentiality]* is such that all our rooms are interconnected. … So, basically in my team, there are… three doctors, one care nurse and one care coordinator, and the case nurse manager… is in the middle and the two doctors are at the side, so we kind of like, in between, just talk among ourselves, and if there are any cases that we're not really sure about, we just go over (them). So, (it's) kind of like, sometimes, because I'm the senior doctor there in the team, sometimes a lot of them come to me, come to my room to ask questions on certain things. So, there's going to be communication definitely.
M1	So, it sounds like it is a good model in which we can even manage cancer survivors?

A	Yeah, I think, so the other good thing we have is that we have a care coordinator, so basically the care coordinator… kind of tracks patients, especially if they defaulted or they missed their appointments, and she can also become a point of reference to (whom) someone needs to talk to. It's kind of like a communications point. So, if, say, a family member needs something, (he or she) can just go and get in touch with the coordinator to get to see the doctors. And also, for stuff like referrals, she kind of like follows (up on) them, so (she does) that bit.
M1	So, that's very good. So, can I ask, how long is the consultation when they are in clinics?
A	The patients? So, it really depends on the complexity. So, … let's say if it's a very complex case, I've gone (for) as long as thirty minutes, thirty-five minutes, and I've gone (for) as short as like five minutes. So, some of them just come in for their cough and flu things, but those who are really complex case(s), those who have been discharged or they've got some caregiver issues, problems with titrations (et cetera), those kinds of things take much more time, but we have the nurses sometimes come in to help out also, (so) sometimes (it) can be quite long.
M1	Because from our understanding from previous focus group, they shared with us that they have general clinics, and then they have family physician clinics and specialized clinics. So, how does this team-let come in?
A	So, the team-let in (our polyclinic) *[omitted for reasons of confidentiality]*, how it works for (us) is that we just concentrate on this bunch of patients. So, we've just started off, so we only have three thousand five hundred patients. So, we're still dividing (the patient load amongst) ourselves. So, if we want to be, we CAN be part of this kind of communications with… shared management of oncology patients. Right now, we're thinking even about *[trails off]*. If we do, because the more we do, I think the more experienced we'll get, so we'll be a bit more comfortable, and I think it's also about what the team-let can offer. If we keep seeing the same things again and again, then our comfort level gets easier.
M1	Yah, that is in resonance (with) many of the other groups who shared, that the more you do, the better you become. So, do you foresee that cancer care is something which can be managed in community?
A	I think I was quite *[trails off]*. When I was reading through your stuff about the survivorship, especially about the psychological aspect of it, I think we would be quite helpful, in the sense of trying to get engaged, especially with, like, the psychological part, how they are getting back to the community, how they are getting back to their old style of life and whether they need help. And I think it's very difficult sometimes where I can imagine it's very difficult to get appointments to go back to the oncologist and I think it's much… easier for them, at this point anyway, to get appointments back to see their FP (family physician) doctors, so we would be able to help in that side.

M1	Because in terms of the oncology centre, they feel that family physicians are really the specialists in terms of psychosocial, as well as in health promotion. However, there were some groups of them (who) shared that, because of the limited time, especially in the general clinic in which they are limited to five to seven (minutes) per patient, they really cannot address the psychosocial (issues). So, can you share, from your own setting, whether this is something that family physicians can do?
A	So, if there (are) issues with the psychosocial part, … we can never tell which patients has got psychosocial issues, so usually in a team-let, how it works, as opposed to in the general clinic, is we just generally ask "How are you doing? What's happening in your life? Is there anything that we can do that is beyond just the medical help?", and I think that's when you create the relationship (and) it comes out easier. Maybe in (the) general clinic, it's difficult to ask those kinds of questions, because you don't have that rapport and you don't have that relationship, and which is understandable. And time is one issue, but I think it's more of like developing the rapport and being comfortable to talk about things that usually might be very difficult for patients to talk about. So, I know I've got a few patients whom I never knew… went through colorectal surgery, and they never really talked about their stoma (bag) until much later on, (on) how difficult it is for them to handle it. And they see private doctors, oncologists and surgeons, but they FEEL like they want to come to me to ask for MY opinions. I mean, I can do (this) up to a certain level, but I really don't know what surgery or what oncologist has *[trails off]*. I don't want to step on the wrong toes, because you don't know what information has been given to them and you don't want to step out of line. So, I think you are right in the sense that time is an issue, but I think more is (about) having the rapport, and I think having, in a team-let or in an FP (family physician) clinic, where they keep seeing the same doctor and they KNOW the oncologist has kind of, like, said, "Okay, you can see this doctor in this place.", I think it may help with the rapport and communication.
M1	That is very encouraging. So, do you think that if there is some communication between the oncologist and the family physician in this team-let, would there be, like you feel more comfortable even going on to a little bit of the oncology part of it?
A	So, I think what we need to know is like it's not that it's particular to the oncologist or anything, but (that) we just need a contact person and it even could be the oncologist nurse or (care) coordinator - someone we can (get) help (from) to ask to get questions (answered), you know, emails and stuff, that would be easy for us to communicate. So, even if, especially the oncologist wants us to monitor for something or for a particular patient, or to do some certain blood tests or something that they particularly need, I think, for us, it'll be easier to handle it for them, because it just saves time and it also gets us more involved with the patient care and that makes, again, the relationship better.

M1	Yes, yah, that's why we realize that in terms of the oncology patient(s), we usually see them only once a year, so… we don't really know what happened to them in between, whereas (for) the family physicians, I'm sure you'll see them more often *[A interjects, "Yah."]* and I think they really treasure the advice from you. Would you say so? Would they come to you for opinions?
A	Yeah. I think a lot of the times, they do come to us, and unfortunately we sometimes say, "Oh, we really don't know and I think it's best that you contact your doctor, your oncologist or your surgeon as soon as possible.", because unless that it's very obvious that there's some growth, like really (a) growth or something wrong that you can see when you examine them, something (et cetera), BUT sometimes you are not really sure of certain things, you know, like -
M1	*[Crosstalks]* – I guess it's all right. I mean, everybody ha(s) their own level of expertise and their own limitations *[A agrees, "Yeah."]*. Would you think that the family physicians can influence behaviour, because we still have many patients coming back, you know, having weight problems, smoking, having stressful lifestyle(s), and we know that these conditions actually lower their resistance and they may be actually more prone to relapse of cancers?
A	Okay, I mean, we do have, kind of like , a counselling service (for) them , and we do have smoking cessation, but in terms of behaviour, because we are definitely more trained in terms of psychological issues, and some of it has to do with motivation, and some of it is due to, like, changed behaviour, because it happens and chronic illnesses also, because we do have to change THEIR behaviours. So, I think we might be, could give some help of course, and if we can't, and if some of the other clinics do have psychologists, and if it is within our vicinity, within our reach, then I think it works well. So, yeah, I think we'll be okay with that actually.
M1	Okay, that's good to hear. So, if you do need the services of allied health, like physiotherapist, occupational therapist or even psychologist, would you have your own support within your system?
A	So, in our clinic, we do very physio(therapists); we have OTs (occupational therapists); we don't have speech therapists, so I know some of them have gone for radiation, those (experience) difficult(y) (in) especially, like, swallowing; we have dietician(s); we have social worker(s); we're just fine but we currently don't have a psychologist. We are part of NHG (National Healthcare Group), but we haven't got a psychologist yet, but we can refer to the psychologist in Ang Moh Kio or Toa Payoh *[reference to geographical locations in Singapore]*. So, we have everything, but maybe we don't have speech therapists.
M1	So, it looks like your system is really well set-up and you have a lot of support. So, in terms of the physician(s) themselves, do you think that the training and education (are) adequate at this point of time to manage survivors?

A	The problem is I don't know what are the problems we're going to face, what are the issues that we need to look out for, so, like, you have your DM (diabetes mellitus) patients, you know exactly what kind of complications you are looking for, you know what blood tests you want to do, and so you kind of like have an idea what you want to do. With like *[trails off].* I was reading (up on) colorectal (cancer), you need to see them once a year, something like that, and then, you need to know the (surveillance) level, like for colonoscopy, you probably need to know what kind of findings (there might be)… , to know how often you need to do the colonoscopy, so these are a little bit more technical in terms of each cancer, so that might be a little bit more difficult for us to grasp.
M1	So, if the patient come(s) to you with a care plan, which we are supposed to give…to the patient maybe at the end of the treatment as a form of communication, do you think this document would be useful? Which are the areas that have too much detail(s), too much technical detail(s) or any areas that you think can be elaborated on?
A	So, I like the treatment summary, what have been completed, what's ongoing and what's the side effects, so I think that's pretty helpful. It IS a lot to read *[M1 laughs and interjects, "Yes."]*, you are right. There is a lot to read, … and I'm not sure how this is going to be incorporated into our computer systems, because it's a lot of clicking.
M1	Right. Do you have any paper record or everything is electronic?
A	Everything (is) electronic, but I have a care manager who kind of *[trails off]*. So, the nurse kind of like keeps notes of more complex cases and she files them. So, when I have a complex case, (when the) patient comes along, she takes (the notes) out, so she knows what's happening, so she gives it to other doctors too.
M1	That sounds very good. What about if you turn to the other page? We think that this realm is really in the area whereby we can really provide more comprehensive care for the survivor(s), but if the survivor comes to you with these particular issues, like parenting or insurance or school or work, do you think that this is within the realm of a family physician (and) would you want them to tell you such things, or it's not really within your scope?
A	Err, I'm not sure about the insurance part, but I suppose, I think the rest seems okay, like in terms of financial counselling, we do have financial counsellors, so that's not an issue. We have psychologists, so (for) sexual functioning, that's okay, we can handle that.
M1	You can handle sexual function? Would you like to share, because some of (the doctors we interviewed earlier) actually said that (they) don't want to talk about it at all?

A	Oh! So, it's a little *[trails off]*. You just kind of like, I mean *[trails off]*. To be fair, I've only asked men about it. There (are) not many women that I've asked about it, but (for) men, yeah I've asked, usually whether there's any issues with erections and how's their sex life. That's not an issue; that's not difficult for me. For women, I haven't really asked… because I don't see, like, *[trails off]*. To be fair, I haven't thought about it.
M1	So, we have one particular theme of the focus group, whereby they were saying, like, breast cancer is a very personal area and usually to manage the patient, you do have to do a breast examination. So, would male family physicians be in a more difficult position to handle such survivors?
A	Not really. I mean, I've *[trails off].* Patients have come in saying that they feel there's a lump there and I've examined them, and I don't think I've an issue with it. I don't think there should be any problems with it. Thinking back on the sexual functioning, my care manager, she's a woman *[M1 clarifies, "She's a lady? Okay, that's good."]*. Yeah, so I mean, I think that might be easier if she had the conversation with her. I'm not really sure what medical treatment there are for sexual problems with women, other than *[trails off]*. I mean, I'm really thinking out loud, maybe like dryness might be an issue; maybe arousal - those are the only things that I can think of.
M1	I guess a lot of it is really the thinking, the mental part, the anxiety?
A	Yeah, possibly, because the only reason why I talk about this to men is because I've a pill I can give them *[M1 interjects, "Yah, so -"]*, so for women -
M1	*[Crosstalks]* - especially if they have breast cancer, we cannot use the hormone therapy, so a lot of them will be very hesitant to use, so most of it is really medication, it's really non-pharmacological.
A	Yeah, so maybe that's why I don't ask them, because I don't think… I can offer much, but in terms of maybe, coming (in to help) in terms of their anxiety, those things, maybe I could talk about those things. But, yeah, I haven't really thought about it. But insurance is the one thing that I'm not so sure (about), because I'm not so sure what they want about it. BUT if they want us to get a medical report, if they want us to write a medical report, something like that, then I suppose if we have information and they are okay for us to type for them, then - *[M1 interjects, "It's all right?"]*. I think it should be fine. We'll just say that we're part of shared care plan and patient had this. And we've got all the information, so I don't think there would be any difficulty.
M1	That's very good to hear. So, how about in terms of training and education, do you think (that) family physicians need a structured one-year course, a graduate diploma in oncology to equip themselves, or is it too much of such information?

A	Well, I remember in my residency, I did some bits and parts of palliative care. I think oncology should be part of it, a little bit? And I did *[trails off]*. You see, the thing is, I was in the UK (United Kingdom), so I was doing houseman-ship, we had to cover the oncology wards, and when I was here, I did haematology, so we just -
M1	*[Crosstalks]* – so, how was the experience?
A	So, (in) oncology, so we kind of, like, you know with the syringe drivers and all that, I know how to do the prescription, and then when I go to oncology, we know, kind of like, all those side effects, what kind of illnesses, what kind of things we're looking out for, but it's more in an acute setting, so I think those are my, kind of like, experiences with oncology and haematological malignancies *[laughs lightly]*… so, doing on-call in SGH (Singapore General Hospital) Haematology *[laughs]* was not very fun, but *[trails off]*.
M1	Yah, I think because in the inpatient setting, they are usually quite sick, so maybe it's best left to the internist to do. *[A agrees, "Yes."]* But are you quite comfortable with oncology patients if they are well, they are stable?
A	I'm okay. The only thing that I usually get a bit taken aback (by) is the medications. I'm like, sometimes I don't know what the medicines are and what are the side effects; because sometimes the patients ask me, "Is this a side effect of the medicine?", I'm like, I have to check and say, "Yah.", but I'm not sure whether that's a very common thing or just a checklist side effect. (And) you don't know whether it really is *[laughs]* or it's just in their mind or something. It's just the medicine – I'm not very comfortable (with it) sometimes, what medicine(s) they are on, but Tamoxifen is very common already.
M1	So, it looks like the family physician(s) actually have the capacity to manage, except for, like, new drugs, but again, it's not really within the expertise to know about all the medications as well?
A	Yah, I think the barrier is, like, you know, (when) the patients come in, they always say, "You know, I'm a survivor." or "You know, I've got cancer.", so they kind of make that… something that's very prominent, something that is very important to them, and they always have (at) the back of their minds, "Is this cancer?", and they are anxious, very anxious, especially when they come to primary care. And they are just like, "Can you help me refer? Do I need to go A&E (Accident & Emergency)? Do I need to do this? Do I need to do that?". So, a lot of the times, the barriers are going to be (the) building (of) the trust between the patient and the (family physician), and whether the patient wants to come to the polyclinic *[laughs]*, whether the patient WANTS to come to see the family physician. I think that would be part of the barrier that you might have to face. I think a lot of it would probably have to be building trust from … the oncologist's side, from the polyclinic's side, to tell them that (they) are still in safe hands.

M1	So, I guess if we can show to the patient that we are sharing care, and then, you know, there is a transfer of care, perhaps the patient may be more confident?
A	Yeah, I think that would help them with their confidence. You see, because sometimes, we could like tell (them) stuff, and we can say, like, “We can email.” – sometimes I suggest to them that, “We can email your surgeon or email your oncologist.” – but if we DON’T HAVE that relationship with the oncologist *[laughs]*, we can’t promise them that they may reply to us *[laughs]*!
M1	Yeah, that’s true. So, you do need a care coordinator.
A	Yeah. You just need someone to say, “Hi, this patient has come. They have some issues.”, because when I was in the UK (United Kingdom), sometimes when… a cancer patient… come(s)… to GP (General Practitioner) care, we’re not really sure, and we’ll just call up the oncologist … or we send an email, but usually it’s a call.
M1	Do they respond when you call them?
A	Yah.
M1	They do?
A	They respond.
M1	You mean they are free to pick up the call? *[laughs]*
A	No, no, sorry! Because they’ve always been on-call. There’s an oncologist on-call, so you can call them and say, like, “Hey, I’ve got this patient. I’ve got this, this, this, problem. Do you want me to send them now, or do you want me to kind of like fit them into your clinic early, or *[trails off]*.”, and they’ll just say, “Whatever.”.
M1	So, that’s very useful -
A	*[Crosstalks]* - yah, it’s quite useful, because especially like certain things like, if you know for a FACT that this patient has got back pain and… they’ve got some kind of like brain tumour and bad headaches but it’s not TOO bad, you are not really so sure whether to send (the patient) to A&E (Accident & Emergency), but they probably need a scan, so (with regards to) those kinds of thing, maybe you can just call somebody. It’s just (for) the grey areas that you are not so sure. So, sometimes we just call the oncologist on-call, because in the UK ((United Kingdom), not everywhere has a tertiary centre. So, basically, like, I was working in a very rural area, … so sometimes if you have to send them off, they probably have to go very far (and) it’s like about seventy, eighty miles away, so you have to really make that judgement, and sometimes you have to call them (and ask), “Okay, send them in.” or “No, it’s fine. We’ll just see them a week or two later.”, but that’s probably because of access to care. Here, definitely it’s easier -
M1	*[Crosstalks]* – yeah, here, you just walk next doors, right?

A	Yeah, next doors, that’s true. But what it is like, sometimes, it’s just, like, if you know that they need something and you are not so sure *[laughs lightly]*, so you just need that extra help sometimes.
M1	Yes, thank you. That’s very good. Is there any other proposal(s) that you have to make this shared care work?
A	Well, I think the shared care could work if *[trails off]*. I mean, obviously we have the doctors and I think there should be nurses involved and the care coordinators. I mean, I don't know how it’s (like on) the oncology side, how they are going to transfer (the cases), but whoever is helping with the transfer, I think it should be taken up by… one doctor, one person and (he) say(s), “Okay, we’ll take this patient. And then, we will then send him to the particular FP (family physician) or the particular doctor team.”, and then, they carry on. So, we just have ONE contact point in each place, so that it’s just very smooth talking, it’s very easy, and then, if they have like an email address or… any contact, we can just email and then they can respond to us within forty-eight hours. I think that should be fine, forty-eight (hours), two days, three days. Yah, I think that’s fine.
M1	*[laughs]* Yeah, and you can always call back the patient?
A	Yeah, for us, that is not an issue.
M1	Yeah, that is the benefit of shared care -
A	*[Crosstalks]* – yeah, we can always say, like, “You know, we need to be *[trails off]*.”. The only other thing is sometimes there’s certain blood test, which you may need but we may not (have), even stuff like Vitamin D, it’s not subsidized.
M1	*[laughs lightly]* Non-standard drug? Non-standard test?
A	Yeah, exactly. So, in NHG (National Healthcare Group), it’s subsidized, so there’s a variety there. So, maybe sometimes you want to be allowed to use, say, (for example), “Okay, you need to do a blood test for Vitamin D. Is it okay that you do the blood test? You want to pay extra or do you want to go all the way to the oncology centre *[laughs]* just to do the blood test and come back?”. I mean, these are, like, the kind of small stuff -
M1	*[Crosstalks]* - yah, I guess the technical part, I mean, we can work out what is best for the patient -
A	*[Crosstalks]* – yeah, you know stuff like (the) PSA (prostate-specific antigen blood test), right?
M1	Yeah, it’s too expensive.
A	Yeah, it’s expensive. If they want us to, like, say, okay, “You want us to monitor, I think you all just send us.”, “Can you please just monitor the PSA (prostate-specific

	antigen blood test) levels?", I don't mind *[laughs]*, but it is expensive. So, you have to tell the patients that and if the patients agree, then it's okay. And you know, there are also some thyroid (function tests) that we don't do? So, a lot of times, it's what (are) resources that we have, so we just need to MATCH it, and if you are going to do this, if you are in oncology, if the oncolog(ist) (can do it), we should be able to do the same thing in the polyclinic. Right? If that's the minimum that we should do.
M1	That's good. So, who else do you think should be stakeholders in this shared care and do you know of community resources?
A	So, in terms of the stakeholders, I was predominantly thinking… the stakeholders should probably be the doctors from the primary care team, and also from the oncologists' point of view, and I think a care coordinator, a care nurse. Whether the nurse should be in the community or is it someone in the hospital, I'm not really sure yet. I'm sure there will be like cancer advocacy groups, support groups. I'm sure they probably have a say in what they want and whether they are okay with this. I think if they are part of the stakeholders, then they can kind of like say, "Yes, we want.". It's easier for them to communicate and also to let them buy in to this idea that this is helpful, and this is going to make it better for you guys (and) it's going to give a better model, a better care.
M1	So, from your knowledge, we just want to know how active the community resource providers are, so would you have an idea who *[laughs]*, because most groups don't know?
A	I really don't know. In terms of cancers, I really don't know. We've just started a support group for diabetics in our clinic. We just started it in our clinic.
M1	Okay. So, what do they do?
A	To be fair, we just started last week *[laughs lightly]*, but basically, they've got a bunch of… about six to - I'm not sure, I think - twelve patients? I'm not sure. They come in with various levels of control, and they just ask the nurse there and they just kind of like facilitate, talking about the experience and learning about… diabetes, what diabetes is about and how it affects them, and they just get to talk about it. Yeah, it was done in English. It's apparently a thing that they learn from outside in the western (countries) - I think it's in England or America - so it's that programme they're just trying to do. It's kind of, like, to help with the psychological aspects also, so I don't know whether if you want to *[laughs]* test still, maybe you can have a support group within the community, polyclinics -
M1	*[Crosstalks]* – yeah, I guess they have their own, in the polyclinic setting, they may have different kinds of needs.
A	I think that could be good, if you want to (conduct them). I think our nurse went for a training programme – she went for a couple of days, and then she just started it. She's from our team, so, yah.

M1	It's very interesting.
A	*[laughs]* Yes, I mean, we just started it. I think it's more like her interest.
M1	Yeah, because we're just thinking of HOW to make this shared care work, how to bring it across, because there (are) so many cancer survivors now.
A	I DO think it HAS to be, like, … cancer survivors have to come together within our polyclinic. You know, I'm sure there are loads there. There are LOADS. We see them, but whether they know (one) (an)other or not, that's a completely different thing *[laughs lightly]*, but if they meet up and if they kind of, like, talk (about) what they need… from this polyclinic, then we can think about (it), "Okay, these are their needs that they want from this polyclinic.", so that they don't need to struggle to the oncology. THEN, we could really do something about it. But (conducting) support groups in polyclinics, that definitely makes more sense.
M1	Yah, that's a new idea. We haven't really thought about that before.
A	I mean, we're trying this with the diabetics, so we don't know. We'll see how it goes.
M1	That's very interesting. Maybe to summarize, would you like share, is there a strong motivation for you to want to look after the cancer aspects of the survivors, especially if it's your own patients?
A	I think that will be great. I think, of course, for every patient that comes along, we want to cover as much as we've done, and if the cancer is part of (what) is affecting their biopsychosocial (well being), then we should know how to help them, because a lot of times, we say, "Oh, okay, talk to your oncologist.".
M1	*[laughs]* Do you think that's satisfactory?
A	It's not satisfactory, because you don't know what's happening, and you feel kind of, like, the system doesn't help you. You know? It's like -
M1	*[Crosstalks]* - but do you feel a bit relived that you don't have to look after that aspect of the cancer?
A	But if it really is, if that aspect of the care is not there, it's like there's really nothing else to it, right? Then, it's like, I mean, the common things like, "Doctor, should I go for this treatment? Doctor, should I go for the surgery?", I mean, like really? *[laughs]*. It's like, "What would your oncologist say?", "I think that's okay.", "I think if the oncologist says this, then what are you afraid of?", and then, "You should discuss these things with your oncologist.". I mean, that's all we can really, really say, because you are *[trails off]*. I know, you know, you are out of your boundary in what you are saying, BUT IF… we're talking about cancer survivors, like later on (after their treatment), … some of the things they always ask, the things that they always worry about is, "Is this going to come back again? Am I okay?", so (their

	concern is) the same thing with ALL patients. You know, like, your stroke patients, they always come with “Oh, I’ve got some numbness. Am I having a stroke again?”.
M1	*[laughs]* it’s like another grey area, right?
A	“Have I got a stroke? Am I getting a stroke again?”. “No, you are not getting a stroke.”.
M1	But you ARE confident to say that, right, like, “You’re not getting a stroke.”.
A	Yeah.
M1	Like, for example, we have many cancer survivors that come back to the oncology centre and they say, “Doctor, I have knee pain. I have finger pain. Am I having cancer?”. I mean, the oncologists really don't know what to say, like, “Can you go and see your polyclinic doctor?”. So, it’s that kind of -
A	*[Crosstalks]* – yeah, so, like, (to) a lot of them, I’ll just say, “No, it’s probably your joints.”.
M1	So, you are confident to say that, right, I mean, it’s something that you are confident with -
A	*[Crosstalks]* – I mean, if it’s kind of like very obvious, I’ll just say it.
M1	It’s like osteoarthriitis of the hands?
A	Yeah, I’ll just say, “No, it’s not. It’s nothing to do with the cancer.”, but grey areas are the ones, especially with treatment, like “Is this medicine giving me this? Is this chemo(therapy) giving me this problem?”, and then, you are, like, “I don't know.”.
M1	Yeah, I guess that’s the one that is left best to the oncologist to answer the question.
A	Yeah. I mean, in terms of (the relevant points) for this interview, I think those post(-treatment) survivors, I think a lot of times, we get patients who get recurrence, and then, they will ask questions like, “Should I go ahead (with treatment)? I’m sixty-five (years old).”, or “I’m seventy (years old). Do you think I should do this?” *[laughs]*. And then, you are, like, you are thinking, “I don't know! *[laughs]* I really don't know! You should really speak to your oncologist about it.”, because it’s always, like, you can only give (advice) up to a certain level. Yeah.
M1	Okay. So, thank you so much for your input today. We really value it.
	[Audio recording ends at 39:28min]
