# Supplementary material for: Implementing a community-based shared care breast cancer survivorship model in Singapore: a qualitative study among primary care practitioners
Source: BMC Prim Care. 2022 Apr 8;23:73. doi: 10.1186/s12875-022-01673-3 (PMC8991467; doi:10.1186/s12875-022-01673-3)
Supplement: Supplementary file 3 — Additional file 3. A compressed folder containing the raw data transcripts and demographics data collection form. [file 12875_2022_1673_MOESM3_ESM.zip › Supplementary Information File 3/FGD (06.02.2018).pdf]

Transcript for Focus Group Interview 2nd June 2018

Key:

Moderator / Interviewer:	M1, M2
Respondent:	Participant A (A)<br>Participant B (B)<br>Participant C (C)<br>Participant D (D)<br>Participant E (E)<br>Participant F (F)<br>Participant G (G)<br>Participant H (H)
( ):	Paraphrases, additions to or rectification of grammar, vocabulary and/or truncated sentences.
[ ]:	Non-verbal, e.g. *[xx laughs] [pause]*
…:	Removal of false starts, repetitive or ungrammatical long phrases
CAPITAL LETTER:	When there is a louder emphasis or stressing on a particular word or phrase

M1	*[Time is taken to settle down; 0:00 – 0:06min]* We'll go down to introduce. *[M2 replies, "Start."]* Can start.
H	I'm H. I work as a private GP (General Practitioner) at *[location stated; omitted for reasons of confidentiality]*.
F	I'm F. I'm working as a family physician in Singhealth polyclinic.
D	I'm D. I'm working as a resident physician in Singhealth polyclinic.
C	I'm C. I'm (a) resident physician in Singhealth polyclinic.
B	I'm B. I'm also a resident physician at Singhealth polyclinic.
A	I'm A. I'm a doctor at NHG (National Healthcare Group) polyclinic.
E	I'm E. I'm a private GP in the north.
G	I'm G. I'm a private GP in the *[inaudible; 0:58min]*.
M1	Thank you for coming today. So, first, we will start (on) the background survey on your current practice. So, can you share with us some of your experience with cancer survivors? *[pause; 1:11 – 1:18min]*
B	Hi, I'm B. We… see cancer survivors mainly for their comorbid condition(s) when they come for chronic disease follow-up. The other time we see breast cancer survivors would be when they come for complications, such as lymphedema, but do not have a regular, scheduled follow-up in the next few weeks.

M1	Thank you, B. Does anyone else have other experience?
G	I'm G. I think, most of them, in my practice, I think (when) patients come to me, it's like in their post-operations or for post-cancer survival (where) they come for just screening mammogram(s).
M1	Anybody else?
C	So, I'm C. So, mainly, I see breast cancer survivors who are on follow-up for chronic conditions. Maybe I will share some of my difficulties when I deal with such patients. So, common questions they may ask me (are), sometimes they will ask what the follow-up plan for them is. So, either they may have defaulted their oncologist follow-up, or they may have been discharged and they are unsure of how routinely they should go back for screenings, and sometimes I find it a bit lacking on my knowledge to advise them accordingly. Secondly, also, I may not have sufficient data… for, let's say, breast cancer patients in general, which stage of the disease they are at. And then, that would affect how strict (the treatment is) towards, let's say, hBA1C (Haemoglobin A1c or glycerated haemoglobin; used for measuring control of diabetes), the diabetic control (and) the lipid control that I would like to enforce upon the patients. *[pause; 3:08 – 3:14min]*
M1	Anyone else? *[pause; 3:17 – 3:24min]* Okay, if not, let us go on to the second topic: "Discuss the perceived barriers to the proposed shared care model.". So, what are the barriers that you foresee with this shared care model? You can discuss them in three areas: first is patient-related, second is physician-related, and lastly is healthcare-system-related.
A	I'm A. So, in terms of patient-related barriers, maybe they would feel like they prefer to see a specialist or their oncologist for various reasons – maybe they feel that the specialist has better knowledge about breast cancer and how to follow up with them, and that the GP (General Practitioner) maybe not have enough knowledge to do so, or maybe because they have the rapport with the oncologists who have been on follow-up with them for a few years. For physician-related (barriers), maybe in terms of the amount of knowledge that we have? So, like, what C mentioned just now, in terms of how strict their control has to be with regards to their chronic conditions, how often do they have to follow up with their mammograms and things like that. But, I mean, if (it's) the shared care model, if the plan that the oncologist shares with us includes quite a lot of details as to how we can follow up with these aspects, I think that would really help us. Healthcare system issues? Time might be an issue as well, because I think in Singapore and in polyclinic settings, we have quite a short amount of time that we can spend with patients, and in general clinics, only five to ten minutes or less. So, on top of dealing with their current medical list, as well as their chronic (conditions), having to also follow up with other things with regards to the post-cancer care might take up a bit more time, so that might be a barrier as well.

E	I'm E. I agree partially with A has mentioned, because, firstly, in terms of patient-related (barriers), (it's) exactly like what she said, (that) there's still quite a lot of perception that the specialist knows best, and often times, I think the specialist probably has spent a lot of time with the patient and is in close rapport too. So, often times, the patient trusts the specialist more. And also, similarly, for physician-related (barriers), also, sometimes there is also a lack of, first and foremost… of confidence and knowledge in terms of dealing with these types of patients. In terms of healthcare-(system)-related (barriers), I think I'd like to add that – a few things – firstly, especially in private (practice), a lot of times, we may not have access to the notes of the specialists, therefore we do not really know the thought process and what are the things that have been done for the patient… especially in practices where it's still pen-and-paper, rather than computer-entered. Also, another thing, I mean, that is just a nature of Singapore is that it's very small *[laughs lightly]*, so it's still convenient, as in, the patient still can get to, for example, NCC (National Cancer Centre) quite easily. It's not like (you have to) take a plane or take a long train ride just to get to NCC (National Cancer Centre), for example. So, in a sense, everything is still very confluent to each other, so they don't mind making the distance once every few months to go see the specialist. Also, I think even in the government sector, sometimes you… still are unable to see the doctor's outpatient notes itself, I mean, you can only see the NEHR (National Electronic Health Record), so I think sometimes (it's) hard to understand… exactly… what the plans are. And also, another thing is that if you, sometimes… for example, if you have any questions or any doubts regarding the patient's history, you are not able to contact the surgeon directly, unless there's really sort of like, let's say, this breast surgeon has contacts with a few GPs (General Practitioners) and they can just contact the surgeon directly. So, sometimes we will have a lack of that, especially if the doctor is lost and needs to seek help from the specialist, but because there is (no means of) contacting the specialist, so there may be some problems.
M2	So, earlier on, it was mentioned that there might be a need for visibility to the clinical notes, especially on the oncology's side, so that you feel better informed and equipped and empowered to treat the patients. I was thinking, if the notes are available to the GPs (General Practitioners) and family physicians, is it all pros and no cons? *[pause; 8:49 – 8:56min]* Or will opening the notes solve everything? Or do you prefer the information to be, perhaps, more summarized, more crystallized and more focused for yourself?
E	Certainly – I'm E – so, certainly, of course definitely opening up the notes will definitely have higher risks of (breaching) confidentiality et cetera. Sometimes, despite reading the notes, you may not understand *[laughs lightly]* what the specialist is writing as well, (but) of course, I think, to some extent, it helps, but definitely, as what was suggested, a crystallized, summarized, more concise version will definitely help. But I think, also, this is going back to the first part (on) "physician-related (barriers)", I think that ultimately the doctor must have *[trails off]*. It definitely has much, much better advantage if the doctor has the experience,

	like, doing a posting (in), say, breast unit for example, even like, say, a three-month posting for example, the experience will definitely be invaluable when it comes to managing such (conditions) in this shared care programme thing. So, but yah, to answer the question, certainly, it'll be good if there will be good, concise information. But will there be risks? Definitely increased risks of patient confidentiality being leaked.
H	I'm H. I'm adding on to the (physician-related barriers), that in relation to chronic care, there is also a psychosocial factor that affect, say, they've never done a posting or much experience in preventive care, they might not feel confident to manage things like depression, loss of identity as a woman after mastectomy. So, definitely, as a private GP (General Practitioner) without easy access to counsellors, to social workers, it may be daunting to take on this role.
F	I'm F. Actually, I agree with H. Sometimes, I also feel (that for) a lot of cancers, colon cancers especially (in) elderly, they don't want to come; sometimes they don't want … to go to specialists.; they want to come to polyclinic. Sometimes, we don't have the facilities, like (in) specialist centres, to investigate them. And also (for) some psychological issues underlying, and let's say, healthcare, we don't have much to explore without full psychological details of the elderly patients. Maybe they don't have any caregiver or they might have some financial issues, so why (don't they) want to see specialist, they don't want to go to the hospital? Is it because of financial issues or some other reasons? So, (for) these kinds of challenges, we also face in the primary care setting. And then, we know that maybe their cancer is getting worse, the condition, but still, they sometimes… don't prefer to see the specialist (and) they want to come to polyclinic to get treatment. So, sometimes, because of the lack of time and the lack of facilities, we cannot explore details of the patients.
M1	May I know what you mean by "lack of facilities"?
For	Like, suppos(ing), in colon cancers, I referred patients (who were) diagnosed, say, ten years ago, they are coming to polyclinic for other reasons, for other chronic medical conditions. Suppos(ing) their condition deteriorate(s), like they have some bleeding, weight loss, we sometimes have to refer back to colorectal to investigate for them, but a lot of times, sometimes the elderly, they don't want to go; they want to come and do all their investigations in the polyclinic for colonoscopy and all, so we have to explain to them, "We don't have colonoscopy facilit(ies) in primary care.". Sometimes, it's difficult to convince them that we don't have these facilities, like we don't have like the specialist centres - these kinds of things, we sometimes face.
C	I'm C. So… one of the barriers that I feel that we may encounter is that I think quite a bit of counselling has to be done for the patient before they are… given this care model to bring to their GP (General Practitioner). We have to let them *[trails off].* Because they may feel confused as to, like, "Why am I given this piece of paper?

	Why?", because generally (speaking), for now, a lot of them don't have the faith that the GP (General Practitioner) can actually co-manage the other aspects of the breast-cancer-related issues. So, they will go to the GP (General Practitioner) and they may find (that), "Eh, I don't know why I'm here, but I'm just here to bring a letter (to you).", So, you have to… give them the confidence. Unless it's for other related problems, like lymphedema, psychosocial problems, depression, then they can actually seek treatment from the GP (General Practitioner), so as to let them know the reason why they can follow up with the GP (General Practitioner) in the meantime, as they can space out the longer appointment dates with the oncologist.
M1	So, are you saying that… when the oncologist gives this particular piece of letter to the patient, they should actually educate the patient on how they can use this piece of information?
C	Yes, I would think so, and then, to also let them know what's the rationale behind this care plan, so that they don't feel confused or they don't feel lost when they present this piece of paper to the GP (General Practitioner). And also, so that you'll give them a sense of, like, understanding of (the arrangement), and they can also prepare, let's say, when they go to the GP (General Practitioner), what are the things they might want to ask (and) what are the advice (they want) from the GP (General Practitioner).
B	I'm B. I just have something to add on to what have been said previously. Regarding patient-related barrier, I think E has actually stated clearly that – and also C – that it's actually the patient's perception, as well as (the fact that there is) no great difference in accessibility between the GPs (General Practitioners) practice versus the oncologist's practice, which makes many of them prefer the oncologist, which is, to be frank, a lot less crowded, and waiting time is not as long sometimes as compared to polyclinic waiting time. And with regards to physician-related factors, I would say the greater barrier for that of primary care would be that of physician's time. There are many things we can do (and) many things we can learn to do, but actually at polyclinic right now, we have 7.5 minutes per patient. And if this same patient is seeing me for diabetes management, hypertension management, cholesterol management and maybe frailty issues, and you add on cancer plan, it's never, ever going to fit, and it may not change in the near future, from what we know of the MOH (Ministry of Health) primary care plan right now. So, in addition to the lack of time, there is also a lack of availability of medication, lack of availability of BMD (bone mass density test) and lack of availability of ACCESS to nursing support, physiotherapy support, occupational therapy support, for things like lymphedema, which is a common problem in breast cancer survivors(s), and also (the) lack of availability to psychological, psychotherapy support. So, all these are actually better accessed at NCC (National Cancer Centre), which is why many patients, (cancer) survivors actually may find that it is beneficial for them to seek care at the tertiary centre rather than (at) primary care. However, I would say that if NCC (National Cancer Centre) makes available their nursing support, or their physiotherapy, occupational therapy services, as well as psycho-counselling

	services to general practitioners, it's a POSSIBILITY that it THEN can be translated down. Maybe we are able to do, like, a pilot programme with certain family physician clinic which has more time resources, location, as well as the access to prescription. So, I would say, in summary, it's the lack of ancillary staff support, the lack of time, the availability of med(icine) and also of investigations, such as BMD (bone mass density test) and mammography services.
M1	Thank you. Okay, we shall go on to the next (part) talking about the survivorship care plan. So, what are some of the information which you think are useful, and what else is missing and should be included?
M2	So, earlier on, we talked about how some information is not available for you to continue care or to have a good clinical picture, so this will be a good chance to tell us what you'll like to see in the survivor care.
B	I'm B. I would say that currently, at least in the public healthcare system, a lot of information is available on the NEHR (National Electronic Health Record), as well as the Singhealth settings available in *[inaudible; 17:52min]*, and actually most of the things in page one (of the form) is found, but a good summary such as this would be useful. I would like to (point out that) cancer type and histological subtype may not be very applicable for us. Everything (else) in the first page, I would say, would be useful. Probably even radiation (treatment) would be useful, I think, if you are looking at side effects of radiation-related risks. In the second page, I'm looking at the persistent symptoms or the side effects after completion of treatment. I would say that if some of the things are meant to be managed at the primary care level, this box is insufficient. "Fatigue" is a VERY generalized symptom. "Numbness" and "pain" are non-specific. I would say it would be a bit clearer if maybe you (have) a bigger box (for) free text and where the physician takes the time to fill (it) in. "Menopausal symptoms", again, would be rather *[trails off]*. There are A LOT OF "menopausal symptoms", and (having) no (text) box will require the general physician to go through the ENTIRE LIST of menopausal symptoms with the patient. Similarly, "psychosocial / depression", if it is present, I would say you may need a larger space to fill in some details, such as, treatment, current stage and current follow-up. Other side effects or things I may want to access would be things like, I'm not sure if ECOG status (Eastern Cooperative Oncology Status) would be somewhere in here – that would be useful – and… any current community or current nursing support, at some point in this page, would be useful, like what other community resources is the patient touching on OTHER than the primary healthcare (provider) or the cancer survivor (resources) or the cancer follow-up. I think that's about it.
M2	Can I just point out that this cancer care plan is filled up by the oncologist and given to the family physician? *[M2 interjects, "For the patient to bring forward or it can be faxed over or emailed over."]* So, it's actually not meant for family physicians to fill up. So, it's actually information FROM tertiary to primary care.

M1	Yah, I think what you say is useful, what the information lack(s), so you think that there are some areas that would need more details especially on the certain non-specific symptoms and psychosocial depression?
B	Yah, and also, it would be useful it you could include *[trails off]*. Because in most other countries, the primary physician is already the enrolled or empanelled care plan, and it's easily available, the social history, family support (et cetera) - everything is established. Whereas in Singapore, you realize that there are a number of patients who are diagnosed with primary cancer, (after) post-cancer treatment, (they) do not have a PRIMARY care physician AS YET. It would be useful to have a handover of things like pertinent social history as well, which I do not see anywhere in this page. Okay, for example, "Patient is on MSW (Medical Social Worker), currently stays in a rental HDB flat.", this may be useful information. "Patient is currently touching on escort services, Meals on Wheels." *[Reference to a social service that delivers free-of-charge meals to the needy)* may be useful information. Things like "Patient is on follow-up with NCC (National Cancer Centre)." may be useful information. Oher pertinent things would be what has been communicated to this patient, what are the specific concerns with regards to breast cancer. (For) this, I don't see a space there. In primary care, it's quite useful if you know what (are) the patient's ideas, concerns, expectations, and what (they have) been reassured by, and if there are any kind of particular concern about the cancer that the patient has, so that we do not inadvertently tell them something contradictory to what the breast surgeon or what the oncologist has already told them. That would help a bit as well. For example, patient is worried about "How often do I need to do my mammogram?", "What is my five-year survival risk?" (et cetera), some of these may be a little bit more iffy.
D	I'm D. So, at polyclinics, we see patients who are currently on chemotherapy or the radiotherapy for breast cancer and attending their chronic follow-ups. So, then we STUCK in some places, (such as) whether we can continue as per normal, or whether the patient might develop interactions with the current medication received from the oncologist. I would suggest this SHOULD include the past medical history of the patient, with the current medication and the possible interaction with the ongoing treatment, and if the patient needs to be prescribed later (on) with the Tamoxifin *[medication for preventing the relapse of hormone-receptor-positive breast cancer]*, and the possible interaction with the patient's current medication - this would be helpful.
M1	Anyone else? *[pause; 23:00 – 23:07min]* Okay.
B	I would also *[trails off]*. Sorry, (I) just (want to) mention, on the third page, there are MANY symptoms listed down here, that if the patients were to come back to me asking me questions about "insurance", "parenting", "sexual functioning", "fertility", "sunscreen use" and all that, I may not be able to answer these things. And I would say that, (for) what is listed there, if you have concerns in these areas, please speak with your doctor or nurses, and they come and see me, and I'll be like,

	"Huh?" *[tone of uncertainty; implies that she is not able to answer the questions]*. So, I'm not sure how to manage this, because you are, KIND OF, in a way, generating questions that MAY NOT be sufficiently addressed.
H	I'm H. If the patient is still undergoing chemotherapy especially, it would be good to know how many cycles there are, because sometimes we do encounter patients who are still undergoing chemotherapy with fever, things like neutropenia and sepsis may be a concern, so I think this information would be quite useful.
C	I'm C. Sorry, can I clarify, under the follow-up care plan for "coordinating provider", does it refer to the oncologist or the family physician?
M1	For the primary care provider here.
C	Sorry, under the pull-out "care plan", the "coordinating provider", does it refer to the appointment to see *[trails off]*. The "schedule for clinical visit", is it with the family physician or is it with the oncologist?
M1	So, this shared care (model) we are proposing is an alternating visit, so it's mainly to let the other physicians know when the next schedule is to come back, either to see the cancer centre or the primary care physician.
C	I see, okay. Sorry, part of my question was that I felt that it was important to me to know when's the patient's next visit back to the oncologist. And then, I don't know whether it might be helpful if there's a section here that we can actually write back to the oncologist about some concerns about patients that we may not be able to fully address and to seek their opinion.
M1	So, if that is so, which is the useful way to communicate, if you do have concerns that you would like to share?
C	I think it depends on this: because on the first page, there's the email and the contact number of the oncologist, if it's urgent, I guess we can call them directly. If it's non-urgent, I think we can "memo" them.
M1	So, in this care plan, do you think it's good to put down who is the primary care physician?
B	It depends on whether there is a primary care physician. If you go to a polyclinic setting, it may be rotation and if you go to a GP (General Practitioner) setting, it depends on what (the) practice (is). So, it would be useful to have one, (but) in our current healthcare system, we may not (need one).
M1	For those in the GP (General Practitioner) healthcare setting, you think it would be useful?
C	I'm C. One other thing is, does that mean that the patient holding this might have all the doctors' contact numbers and email?

M1	I'm afraid that is an issue too. But I think in this current cancer centre, they do provide emails, most of them.
A	I'm A. So, I think it's actually good, I mean, it's IDEAL if the patient has ONE primary care physician to follow up with. I think that is not only ideal for cancer patients' follow-up, but it's ideal for any patient in Singapore, but it's difficult to manage in the polyclinic setting. When it comes to private GP (General Practitioner), it may be easier because they go to one private GP (General Practitioner). But I DO know that in the polyclinic settings, they have family physician clinics which are run by senior doctors where they actually have one-to-one contact with patients. In NHG (National Healthcare Group) polyclinics, they are actually doing this team-up system where it's not just one doctor, but maybe two to three doctors to see the SAME POOL of patients. So, the whole idea is that you follow up with (the same group of patients), you see a few familiar patients, and then, you follow up with them. For this care team, we have nurses as well, we have case coordinators as well, so maybe that will be better for the patients because to them, it's a bit, like, it's maybe also like a smoother process in NCC (National Cancer Centre) where they not only see the oncologists, but they see the nurse, they see the social worker and everything as well. So, maybe in the new care model where, like the NHG (National Healthcare Group) is planning to come up with, … it'll be more feasible for them.
B	I'm B. I'd like to follow up on that: I agree that the team-up model is gaining ground in most of the polyclinics in Singapore, since it (has) been piloted two years in a couple of polyclinics. I would like to highlight a possible model, where you decant SELECTED… more suitable patients to a shared care model, by intent to, like, shared breast clinic, (just) like how we have shared psychology clinic. So, like the psychiatrist comes down to run a shared care clinic with the family physician (for) maybe about one or two days a week, and to see a pool of patients who are referred internally. Similarly, we do have patients that we pick up for cognitive impairment or dementia, who come back on a particular day of the week, and we have a neurologist who comes down to run the clinic with us in a kind of shared plan model, and this is how we gain experience, as well as (have) patients gain some confidence in the system. This is by appointment only, and… I would also suggest (this for) selected patients, selected by the oncologist, to help to start this model, you know, start this as a pilot, rather than giving the patient a sheet of paper and say "Please find a family physician of your choice.", who may or may not be ready to accept this (kind of) care (arrangements).
M1	So, seems like it's not suitable for the polyclinic but the GP (General Practitioner), where there is only one doctor?
B	So, can approach certain GPs (General Practitioners) who may be more willing to take up this role and provide support.
M1	How about the ladies? I'm sure breast cancer survivors will want to see female primary care physicians?

H	I’m H. I think we have such a pattern. As a private GP (General Practitioner), I’ll be more confident. At least, I would have the contact point to ask, if I have queries, and also, (for) information, because a lot of times, not a lot of things are available NEHR (National Electronic Health Record). We only have access to the discharge summary and we don't quite know what’s the clinical plan. So, from a private GP (General Practitioner)’s perspective, yes, there’s a time when it becomes a factor, but if the patient is comfortable to be encouraged for compliance and follow-up, and better management, then I’m willing to do it, if there is such a document available.
M1	You think that you are willing to do it. Do you think it will help to enhance your practice? Will it benefit the patient?
H	I think it would definitely benefit the sick patients, especially if the proximity of the house is nearer to where the GP clinic is, compared to NCC (National Cancer Centre). And we also free up slots in NCC (National Cancer Centre) for patients who need it more, but I agree with B that it should be certain selected patients, because patients still prefer a complexity of care, and it’ll be better if certain selected patients, who are more ready, are referred for a transition, rather than (for) ALL breast cancer survivors.
M1	Okay, thank you. So, our next topic that we go on to is “relationship with stakeholders”. So, who do you think are or should be *[trails off]*. Okay, we’ll go on to the motivations first: what do you think are some of the motivations to participate in this shared care model?
M2	So, earlier on, we stated some barriers. So, if you can just foresee… all these barriers that impede your care of breast cancer survivors, then perhaps some of these barriers, if you have some proposed solutions, it may become motivations, so that’s how we can think of it as well.
B	… I’m B. I think one of the big motivation(s) would be (that) I have patient(s) who are currently on follow-up with me, whom I would be confident to claim I’m the primary care physician, and this patient happens to get breast cancer, I’m more than happy to keep continuing follow-up after the primary management, surgical management has been done. This will be an ideal, so you are basically providing this patient with continuous and holistic care, and you are actually bridging the gap between tertiary care and (the) transition back to primary care. And I would already know the patient better on the social background and what is expected, and I think this will be a motivation for me for continuing the care of a patient that I’m comfortable with. Of course, the other motivation, I suppose, would be in terms of public service, you know that there are certain patients who do not require tertiary service and if you can transit them back to the community side, it will actually benefit them. One of the barriers, I would say, the community support of cancer survivors – anything in general, (such as) nursing support in the community, the physiotherapy, OT (occupational therapy), psychotherapy (et cetera) – is not very

	established as of yet. It needs some building-up of infrastructure before we say that we are confident to help them out in this sense. So, I suppose if we have the availability of services, it would be easier. But I think (with regards to) motivation, it would be right-siting, in a sense.
M2	Earlier, it was stated that some of the barriers are (the) lack of access to investigations. I was thinking just whether there (are) some proposed solutions for that, that might motivate you to take this on? So, you are saying that you can't really get access to CT scans from your GP clinic, so do you have some way around it? *[pause; 34:11 – 34:19min]*
E	I think, for this shared care programme to work for GPs (General Practitioners), there must be a few factors that happen. Firstly, the GP (General Practitioner) must have a certain interest in dealing with such things in the first place. Secondly, the truth is, practically speaking, … financially, it must make sense. So, the GP (General Practitioner) has to be remunerated accordingly from whichever source for spending more time and effort, and also for undergoing the training to be able to handle all these things, you know, to learn how to handle all these things. So, the remuneration part is definitely important - there's no denial, especially in the private sector. So, and but, apart from that, like I said, the GP (General Practitioner) must have personal interest in doing such things. Also, in terms of, let's say, like what M2 has mentioned about CT scans, I think when it comes to *[trails off]*. I think if the GP (General Practitioner) feels that this patient needs more investigations to such an extent *[trails off].* I'm sorry, I'm E by the way… What I'm trying to say is that there must be a very seamless re-referral back to the specialist, and for the specialist to make the decision, whether you need to do all these further investigations or not. At the same time, there must also be mutual trust between the specialist and the GP (General Practitioner), so I think only selected GPs (General Practitioners) – not only selected patients but selected GPs (General Practitioners) - … whom the surgeons can trust to discharge these patients to. So, I don't think it can be a run-of-the-mill GP (General Practitioner). Maybe certain regions, certain groups of GPs (General Practitioners) are trained, with a special interest and who are willing to take up this programme.
M1	Would you like to propose any form of remuneration? Who should the remuneration come from? Is it just from the patient or should it be from other people?
E	Ultimately, as in, I think, it probably has to come from the government sector side, because I don't think the patient would be willing to foot *[trails off]*. As in, when the patient comes, … I think when they pay to see a doctor, it should be pegged to the same price as you pay to see a specialist, otherwise if they pay more, there's no incentive for them to see the GP (General Practitioner) – they would rather go back to NCC (National Cancer Centre). Maybe just a small difference is okay, but if the difference is relatively big, definitely they will still go back to the specialist centre. So, it really depends on how much, I guess, the private sector charges, so the

	difference would be picked up by someone, and it'll most likely will be from the government sector side especially.
H	I'm H. I want to add (on) to (what) E (said), also as to (how) to make financial sense to the patient. I think for chronic conditions, like hypertension, diabetes, we don't know if our patients can claim from Medisave. Personally, working in *[location of clinic; omitted for reasons of confidentiality]*, I have access to DRP (diabetic retinal photography) at *[location of clinic; omitted for reasons of confidentiality]*, but in terms of, let's say, for breast cancer survivors, if we were to order BMD (bone mineral density test), it's not covered, and it costs hundreds to the patients. it just doesn't make sense, and the patient would then prefer to go back to the specialist that they are seeing at NCC (National Cancer Centre), since there's access to a social worker, access *to [inaudible; 37:51min]*, and it's just a lot cheaper.
A	Hi, I'm A. So, (I'm) just sharing a bit of experience because I studied overseas in the UK (United Kingdom). So, over there, it's a bit (more) focused on primary healthcare, and also GPs (General Practitioners). They actually have this reward system – it's more like a points system. So, if your clinic is able to support, let's say, breast cancer screening - I mean, they apply it to chronic conditions like hypertension and things like that as well – so if let's say a percent of your patients, who have hypertension, can achieve their blood pressure goals, (then) you'll get a certain number of points. And then, at the end of every quarter of the year, they actually accumulate the points and they actually give you money for your practice to use. It doesn't really go to the primary care physicians per se, but it goes towards the practice to ENHANCE the services that they can offer to the patients. So, maybe that is something you can consider here as well. I agree with what B says (about) … targeting certain practices in Singapore in certain regions – maybe dividing Singapore in different regions like north, south, east, west and central – and having maybe two to three (practices) per region where the GPs (General Practitioners) themselves have special interest in breast cancer, and then… where they can maybe go for short courses organized by NCC (National Cancer Centre), teaching them about the important things to look out for when continuing care for these patients. I mean, you can't get all the GPs (General Practitioners) to do a three-month posting with NCC (National Cancer Centre), so maybe short calls will do - I don't know - maybe (for) one or two weeks. And then, in terms of communication between the GPs (General Practitioners) and NCC (National Cancer Centre), I guess that would be easier to facilitate as well, because we don't get SO MANY GPs General Practitioners) contacting one oncologist; you get maybe those few GPs, so I guess that's easier that way as well. And In terms of motivation for the patients, I guess you have patients who insist on seeing their oncologists, but like what F says, they are patients who REFUSE to see their oncologist! So, … I think that's the whole basis for primary care GPs (General Practitioners) in the first place, if there's easy accessibility for the patients, so there are patients who PREFER to go to the GP (General Practitioner), then if the GPs (General Practitioners) are equipped, then I think this a win-win situation for both sides.

M1	So, in the interest of time, I'll just merge the last sections about stakeholders: "Who do you think are or SHOULD BE stakeholders in this shared care model, and what are the possible barriers that may affect communication and seamless coordination and transition of care? At the same time, you can also discuss about the community resources. Who are the community resources available, and who we can engage and refer for effective shared care?". *[pause; 40:55 – 41:10min]*
B	I'm B. So, to me, the most often neglected but very important resource will be the private care physicians, because, in every model of shared care, the planning tends to happen at the tertiary centre and in the government, MOH (Ministry of Health), and it tends to only roll out only in the last phase, and if you ask the person you are decanting down to, why do they think about it, you'll find out that, "Oh! We have given you all the heart centre patients to decant to you, (and) we just give them a memo to send to you." but, you know, there is actually no shared plan. We are just right-siting them. So, I suppose it's good to engage the end users and the patients especially, see what the patients' point of view are and how their reception is. My suspicion is that only a certain (percentage), if you are lucky, fifty percent of the patients will be willing for decanting, and you'll need to get their buy-in before you push them into the shared care model. If not, all that you'll get (is that) they'll just end up run(ning) back to NCC (National Cancer Centre) on their own. It's good to engage their primary care physicians earlier. At some point, I would say it will be difficult to fit it into normal general clinic at polyclinic. You may need to create, engage stakeholders, like polyclinic directors, manpower people, (and) if you have time and space to find a shared care clinic slot, like a morning a week just to run this kind of cancer clinic. I don't think it will ever work in our general clinic setting, given your 7.5 minutes per patient, including hypertension, diabetes and what else you have. So, I think it's important to engage early, so that appropriate things can be done before you roll out the plan, which is what this focus group is about – it is to move. And regarding seamless communication and transition of care, I think that our electronic record(s) in Singapore is not bad, but the government has been saying for quite a while that electronic shared records for GPs (General Practitioners) will happen. I don't think it's seamless yet, in any sense and form, and the reimbursement and setting up of electronic shared records for the private setting is having issues *[laughs lightly]* that I know of. So, if you are going to roll out (for) the GPs (General Practitioners), …. you have to boost up the electronic interface. That's it.
M1	*[pause; 43:48 – 43:59min]* Anyone else have any input regarding the community care resources, besides primary care physicians? Who else -
The	*[Crosstalks]* - I think – I'm E by the way – probably one very important role is having breast nurses, which already existed I'm sure, so I think the breast nurse, the coordinating nurse, case manager will be probably crucial in this whole thing as well, if there is one more third person. Probably, patient (will have) a closer relationship with the breast nurse as well, such that if any problems may arise, they go to the breast nurses first, and the breast nurses may be there for the patient to

	bring the patient to the GP (General Practitioner) or specialist. I think, the GP (General Practitioner) first, because the GP is easier to access, especially there is a pool of them et cetera, so I think it would be crucial, essential, in this shared care programme. So, having a coordinating person is actually essential.
B	I agree with E. I think having a coordinator with SOME kind of background - a nurse would be ideal – can actually also be, like, the interface between the GP (General Practitioner) and the oncologist. She can also answer any initial answers that the patient has, she can assist to maybe book the appointments on both side(s), and also, if you are a sufficiently-trained senior nurse who is oncology-trained, you can even get your nurse to start training your GPs (General Practitioners) on the common questions, the common handling and things like that. And importantly, the nurse supposedly would have more time, so you can even start recruiting patients from the phase (of) post-diagnosis while the nurse is seeing them, and the same nurse will gives care, will be seeing them in NCC (National Cancer Centre), who then helps to transit them into general practice.
M2	Can I just clarify, for these breast care nurses, (it) sounds like you are thinking and talking about them (as being from) tertiary centres, right? Not as a community resource?
B	So, what happens is that you offer them a different kind of package to not only work in primary care setting, but also work as what we call "transitional care nurses", like how we have those care coordinators in the community. Most of the time, they recruit them from within the hospital, and then, you get them to set up and helm a pilot community shared programme. And I would say (to deploy) nurses (for this role) because the doctors-resource is even more stretched than them.
M2	Okay.
M1	Anyone else?
M2	Earlier on, there was also mention about (the) lack of counsellors, psychologists (et cetera) to help with the psycho-emotional-social parts where your practice is concerned. I mean, will this tie in with community resources?
E	I'm E. I would casually lump them under "allied health", "allied health community help", so this would definitely be very helpful. But having said that, sometimes having too many (people helping) also gets confusing. I mean, good to have them available, but ultimately, I feel having one experienced breast care nurse will do the trick, because she can also double up as a little bit of psychosocial support et cetera, so if it gets to complex, then refer to the doctor or refer to the appropriate allied health specialist.
B	(To) further (add on) to this, I would also say community resources would also be – pardon my lack of personal knowledge in this but – in most diseases, you have a kind of a society for something, you know, like society for diabetes, society for

	Parkinson's Disease and all that, (so) I'm sure there is a society for breast cancer people out there. Get their partnership and buy-in, so that when they have support group, they can also talk about, "Eh, I see this GP (General Practitioner). His management of me, very good what! So, you can see him also.". I mean, there's a lot of those fellow patients *[trails off]*. You know, (in) Singapore, it's always like, "I tell you it's good, so you go.", so I think that may be useful. That's community support, especially among people who have been through the same experience, it's totally different from the medical perspective, and I think that will help A LOT with the patient buy-in. So, in the same sense of that, it MAKES sense to have a pilot of a smaller group of people, and then… ENSURE that they have a good experience, and then, get these pilot people to go and sell it to the rest of them.
M1	That's a very good point. Thank you. Anyone else have any last words? Okay, in that case, thank you very much for coming, and we hope that we'll be able to use the information to better support patients. Thank you.
	[Audio recording ends at 48:59min]
