# Supplementary material for: Implementing a community-based shared care breast cancer survivorship model in Singapore: a qualitative study among primary care practitioners
Source: BMC Prim Care. 2022 Apr 8;23:73. doi: 10.1186/s12875-022-01673-3 (PMC8991467; doi:10.1186/s12875-022-01673-3)
Supplement: Supplementary file 3 — Additional file 3. A compressed folder containing the raw data transcripts and demographics data collection form. [file 12875_2022_1673_MOESM3_ESM.zip › Supplementary Information File 3/FGD (07.10.2018).pdf]

Transcript for Focus Group Interview 10th July 2018

Key:

Moderator / Interviewer:	M1, M2
Respondent:	Participant A (A)<br>Participant B (B)<br>Participant C (C)<br>Participant D (D)<br>Participant E (E)<br>Participant F (F)
( ):	Paraphrases, additions to or rectification of grammar, vocabulary and/or truncated sentences.
[ ]:	Non-verbal, e.g. *[xx laughs] [pause]*
…:	Removal of false starts, repetitive or ungrammatical long phrases
CAPITAL LETTER:	When there is a louder emphasis or stressing on a particular word or phrase

M1	Okay, just before I go on to the background, just some ground rules: so, we use alphabets, so don't use names. When you are addressing yourselves, say, "I'm Dr C referring. I agree with Dr A.". And then, one person speaks at a time.
Unidentified female	You mean, try like this?
M2	I'll lay the rules, don't worry. *[M2 replies, "Okay, you can do."]* I'm going to turn this on.
	[Moderators take some time to set up; 0:28– 1:14min]
M2	Okay! Hi, everyone! Thanks for coming! Allow me to introduce myself. My name is M2. I'm an associate professor at *[place of institution; omitted for reasons of confidentiality]*. I'm actually a pharmacist, but my research has always been health services research, as well as looking into how to optimize the survivorship as a supportive care in our cancer patients. So, I have a joint appointment at NCC (National Cancer Centre) as well, and that's how I work with M1. At NCC (National Cancer Centre), we've been trying to do a lot of work on how to MOVE some of our care of patients out to the community. I'm sure you all know about the fact that there are a lot of cancer survivors nowadays, because we have SO MANY breast cancer survivors, colorectal (cancer), prostate (cancer), that we're trying to figure out what's the best way to optimize the care for these people. So, (for) today's focus group, primarily, we're trying to figure out whether a community shared care model is possible or not. Through a qualitative focus group sort of discussion, we're hoping to get open-ended responses from all of you, because you have filled up a very simple survey, but if we do a QUANTITATIVE type of survey, not a lot of facts will come out, and usually in conversations only, it would be generated when we start talking about the views and perspectives. So, M1 has

	eluded that what we're going to do is that I'll ask some thematic questions and then anybody can start off and just talk. Try not to identify yourself, other than the fact that you are Dr A, B, C, D, E or F. And then, you know, because, again, we want it to be anonymised. The recordings will only be processed by our research assistant, so people who are NOT HERE today will be listening to the tape, so you will not be identified. So, feel free to express your views; feel free to keep everything, whatever that you want to say, within four walls here. And because you have consented also, that whatever research findings that we are going to publish, we're not going to have your names on it, so again, you'll get anonymised. If you feel uncomfortable, just let us know.
M1	So, I guess I'll just go through the slides just as background information? *[M2 replies, "Sure, can."]* So, this is our current model of survivorship care, which we think is in Singapore, so we think that generally, it's quite fragmented, in which cancer survivors mainly see primary care physicians for comorbidities and health promotion, and the main thing with the tertiary centres is at referral and diagnosis; and because of this limited collaboration, it has resulted in unmet needs. So, this is our proposed shared care, in which we hope that in the patient cancer journey from diagnosis to treatment, to early and late survivorship, that it'll be a collaboration between the oncologists and the primary care physicians. So, but the role of the oncologists at the diagnosis is a major one, in the sense that they manage the treatment and the management and surveillance. We hope that along the cancer journey, primary care physicians will play a bigger role, especially in the area(s) of health promotion and prevention of diseases. Okay, so (for) the last slide, it's just from one of the articles which we shared (on) why we need community survivorship, and John Anaway *[requires verification; 4:20min]* – this is from Australia - … noted that there's a higher risk of chronic diseases in breast cancer patients, and because they often have a poorer compliance with comorbidities of diabetes and cardiovascular disease(s), mainly because they are very preoccupied with their cancer recurrence. And he also noted that uncontrolled chronic diseases also limit treatment options, and many of the chemo(therapy) drugs also have their own side effects, especially that of peripheral neuropathy. And he noted that cancer recurrences happened in between hospital appointments especially in primary care. And lastly, it's that oncologists are mainly trained in cancer diagnosis and treatment, and they really may not be the ideal people to provide health promotion and diabetes prevention. So, we'll go on to the current practice.
M2	So, what we are going to talk about today will be based on the interview guide. The very first question, really, is just to get a very quick overview from each of you about what your experience with cancer survivors (is) right now, IF you have seen any in your practice. We can keep it very short just so that everyone can talk about it. Maybe I'll start with you? *[Unidentified female says something inaudible; 5:31min]* Yes, we start recording already. So, can you just share, like, do you commonly see cancer survivors in your practice?

C	Erhm, we do, but usually - *[M2 interjects, "You are Dr C, right?"]*. Yah, C. Must, must?
M2	Yeah, it'll be easier for the transcriber.
C	Okay, I'm C. Yes, we do see cancer patients in our clinic, but usually they come for other health problems (and) not the cancer problems, like upper respiratory tract symptoms or diarrhoea, that kind of thing.
M2	And when do you see them? Are they receiving chemotherapy or is it out of their treatment already?
C	Usually, it's out of their treatment. Most of them who receive chemotherapy, I think they know that if they feel unwell, they will call the department in oncology, to ask them what to do, and while they are having chemo(therapy), neutropenic sepsis, and things that they are very concerned about. We did manage, we did see one guy who came here having fever for a few days, one month also after chemo(therapy), and he was quite sick. And initially, we do not know what he was here for, he said, "Because I have sore throat and fever for about one week, ten days, and I've just had chemotherapy about three weeks ago.".
M2	So, on average, how many survivors do you tend to see, let's say, on a weekly basis?
C	I myself normally see one per week?
M2	Okay. How about you, B? Thank you.
B	So, I see some cancer survivors are actually here for their chronic disease management. With respects to the malignancy that they have in the past, I just make sure they are on follow-up or they are discharged. Of course, if it's for a patient who is actively undergoing chemotherapy, like C mentioned, I'll have to make sure if they are presenting with a fever, or other kind of things like sepsis. But also, with regards to cancer survivorship, for these cancer survivors, for instance, for breast cancer, if they have had breast cancer, I ask them are they going from regular mammograms.
M2	On average, how many do you see a week?
B	So, I don't really keep track, because *[laughs]* if it's malignancy, we are not actively managing the cancer per se, but it's a chronic disease patient, I would say, among the chronic disease patients, I see about at least ten, fifteen (of them) per month?
M2	Okay, okay. A?
A	Yah, I experience similar experiences as C and B. Usually, it's for chronic disease management that I would be attending to the patients, so if patient has chronic medical problems along with malignancy, which has already been treated. Of

	course, I do experience patients who come for acute problems as well, where they have history of cancer in the past. And I think we do have (a) few patients who are breast survivors and who are like on Tamoxifin and all, and follow-up with the hospital. So, on an acute case presentation, I think I would be more looking out for red flags to see whether it's like related to the malignancy itself. And for secondary (cancer) prevention, of course we do give preventive care also for this patient, like we do monitor for other cancers as well, for example, (in) breast cancer survivors, we'll still be monitoring for other cancers, like perhaps cervical cancer and all these as well. But I guess if you ask me the question how many do I see, I guess it'll be around five a month, maybe? *[M2 interjects, "So, not very many?"]* Not very frequent.
M2	So, D, F and G – we have four here, right - do you all agree with what they are saying? Is there anything that you want to add? Or you see similar numbers as well?
Unidentified female	Yah, similar.
E	If you want to listen (to) something different, then I may be able to clarify some big difference from the others. I'm E. For me, I probably see more (patients) than what the rest of colleague(s) (see), because when I open the patient profile, I just open - it's just my habit to open everything - so I tend to pick up a lot of them before the patients come in, knowing that they have whatever cancer, whether they are on chemo(therapy) or whether they are already treated, when was their last CT (scan) would be all in my mind already. So, one part (that) I agree with them is that most of them are within chronic disease follow-up and the whole thing (is) not related to cancer. There are also some of them (who) are coming for problem just to see cold, flu, like, similar to non-cancer patients. But I do encounter (a) few case(s) that (are) related to the cancer, for example, like last week, I just saw one lady currently under chemo(therapy) for breast cancer, and then, she has a lot of eating problem(s). Then, they want to get medication and that is totally unrelated to cancer. Then, there are also cases I see, that is due to the complications of the cancer, for example, if the cancer *[trails off]*. Although the patient did not actively tell me that (he is) coming in for this problem, but it's just that when he (came) in, I look at him, the whole body become very yellow, then I go and actively ask and all that.
M2	So, you'll deal with some complications. So, everyone has SOME experiences with some survivors. The question is, most of the time, you deal with physical problems, do you guys deal with psychosocial problems of the survivors as well, meaning, (they) fear about cancer recurrences, they worry about how to deal with issues at home because they were sick. You ever encounter those kinds of problems? Anyone?

A	I'm A. Actually, surprisingly, the patients that I see, they have a very positive (out)look. *[M2 clarifies, "Survivors?"]* For the survivors, yes. So, I think they are going through a lot of counselling and all, and so they are very positive - the patients that I have encountered so far with survivors.
M2	Okay. Anyone agree?
C	… I'm C. The other thing I found in one of the breast cancer survivors that I've seen is she has a heightened sense of the symptoms, you know, from then on, like a little bit of cold, little bit of rash on the body and especially on the breast, she comes to me. I've seen her two to three times a week.
M2	Okay, so did you actively refer out to other people to further work out, or do you do more of TLC (tender loving care) help for the patient?
C	She is under active follow-up. She just got the clearance after chemo(therapy) that there is no metastasis, so for my part here, I'll look at all the medical problems that she brought to me. Those problems so far, to me, (are) pretty simple. There's not a sign of cancer-related or chemotherapy-related (issue), for example, the rash that she came here for, it was just eczema that she has had since her younger days. For the simple cough and cold, she was worried that it might be something to do with the cancer of the throat – there are times when she came (for those fears).
M2	So, it looks like people who come for the chronic illnesses follow-up, they are aware of their issues, they are not too nervous and they are comfortable talking about their problems with you guys? You think that's the case? Okay! So, what I want to move on from here is, the rest of our conversation today will be based on what we call the "shared care model". So, you know, we have this piece of paper here and you flip to the back page, so this is something that we are planning to propose and see how we can further increase the number of cancer survivors being seen at the community by, I guess in this case, polyclinic doctors. So, the shared care model, if you look at this paper, there are four, so to speak, domains that we will discuss. So, "time of transition": when are we going to refer people. We are planning to refer to people three to six months after they have completed active treatment, and what happens is that because it is a shared care model that we are talking about, there will be alternating sort of visits between the oncologists AND the primary care physicians – so, in that case, that will be you. So, who are we targeting? We are sort of looking at breast cancer survivors, because that is the largest bulk of survivors who have completed, what we call, "adjuvant chemo(therapy)" – so, in other words, they've gone through surgery, cut off the lump and then, followed by chemotherapy. They COULD have been on hormonal therapy, including things like Tamoxifin, Amiridex, the aromatase inhibitors. But what we know is that there are LOW RISKS for complications of recurrence of cancer. So, this group of people, it's likely that their cancers are "acute". I just wanted to use that word here. "Responsibility", if you can see, (it's a) very distinct sort of role. So, the oncologist would be more in-charge of screening for secondary

	recurrence of cancers, secondary cancers and then, cardiotoxicity management, mainly because we believe it's a lot more complicated when it comes to patients who've got, let's say, Anthracyclines and Herceptin (Trastuzumab) and they have cardiotoxicities. So, let's say, ejection fraction drop(s) by twenty percent (and they have) symptoms, we feel that it is important for us to have closer follow-ups. BUT in a PCP (primary care physicians) setting, we hope that we'll be more focused on managing physical and psychosocial issues. So, (for) physical, we're talking about toxicities, we're talking about, let's say, they have numbness and tingling after their chemo(therapy); we're talking about their fatigue, let's say a lot of them, some may even have minor cognitive changes, "chemotherapy brain" - I'm sure some of you might have heard of. And then, also health promotion as well – so, looking into how to help promote, let's say, if they have bad behaviours from smoking or drinking. AND of course, the big part which many of you have been managing, the management of comorbidities. And then, lastly, the "process": so, the provision of survivorship care plan upon transition. So, there will be electronic communication. And I think there is a coordinator also, so it's going to be a lot easier for communication to happen between you guys with the oncologists. And to ensure that everyone is on the same page about how to manage, there will be didactic education workshop(s), clinic sessions, we're talking about maybe even thinking about putting pathways together, so that it'll be easier for you and your colleagues to know how to manage side effects or problems associated. So, let's say "refer a patient", you know how to refer and you know who to refer – "standardized care", if that's the way I would put it. And you can tell me your views, whether you like this, or you don't like this at all, but essentially, this is the model that we are thinking to put here. So, let's move on to the next question for the interview. I guess now that I've told you what we have in mind, because you know, everyone is trying to push patients out of the hospitals, because you know, again, if these are survivors and they are already done with their treatment, there is no reason why these people need to come back to the cancer centre. Now, I've done a lot of work talking to survivors about their perspectives. I can share with you what they think, but we want to hear what YOU think. Maybe we'll start from this way. Do you see any perceived barriers with this model of what we have talked about? We can talk by different perspectives – you as the doctor, the healthcare system, what do you think? Does this sound too good to be true, or you think that's just too idealistic or do you think that this is the best that can happen?
D	I'm D. So, I think it's a good idea to have the shared care model, because parents walk in to our clinic(s), they always find coming here easier, whereas in NCC (National Cancer Centre), they have to book appointments and the clinics would be three weeks later. So, for (with regards to) barriers, I'll be thinking, "What is expected of me? Like, what type of care should I give?". So, I think it's good if we have a pathway.
M2	Okay, okay. Anyone else? E?

E	I agree with this is the way we view (it). As what D has said, because when patient want(s) to go back to cancer centre to see a specialist to see, it require(s) appointment, but at the same time, I would suggest that *[laughs lightly]* it may be better to have a more standardized pathway to guide us more on what to do, because we are, in primary care, we are, (after all), not quite trained in cancer-related (interventions), so we may not be able to know how to, for example, do psychosocial issue. Also, we don't know if we can support. The queue, because… our patient slot is actually (one patient in) every five minutes. *[M2 probes, "So, you think time constraint IS a concern?"]* Time constraint is a BIG concern.
M2	Okay, would you all agree with that? *[A few participants reply, "YES!"]* Okay, so, yeah *[trails off]*. Go ahead.
A	A. I guess, in general clinic, there is a time constraint, like I think we can overcome this barrier, because it looks quite ideal. With special training, we have some family physician clinic where we get more time to talk to the patient, so if we can overcome the time constraint if we have a special clinic for that, special allocated slots for that, so I guess that's still doable. (But) for general clinic, yes, I agree with E that, we have to admit there is a -
M2	*[Crosstalks]* – it's not possible? Any other views?
F	F. So, yah, I agree with whatever my previous colleagues have said. So, yes, time constraint is a BIG, BIG barrier I think *[laughs lightly]*. We'll be able to see (the patients), but it'll be a very cursory king of thing, and if I'm the patient, I won't feel secure or comfortable, I won't feel well coming out of the consult. I would just (be) like, "Okay, I just get help for my cough and cold, but I don't feel *[trails off]*". I don't feel that I am treating the patient very constantly, especially if he was coming SPECIFICALLY for this model that you mentioned earlier.
M2	How about the healthcare-system (factors)? Do you think the healthcare system is ready for this? Like, do you think the patients are ready to vacate (the) cancer centre and come to polyclinic to get survivorship follow-up? I mean, I think we all know how our patients are like, right? Do you think they are ready for this?
A few participants	Not ready yet.
C	Like, the patient I just told you about, who just completed chemotherapy, I would say she has anxiety issues or heightened sense when, if you ask her to come here and have a breast cancer follow-up thing, even alternate, I don't think that she would be confident about us looking after her.
M2	What's the confidence issue that you think is a concern?
C	Well, to a person, cancer is a BIG thing. So far, the mentality is, you know, in the general population, I'm not sure whether they feel that General Practitioner or family physician is good enough, (in terms of) knowledge (and) experience, to

	actually treat them and follow up (with) their care SO SOON three to six months after post-chemo(therapy). So, it's a lot of education and change, from the specialists' part, that they need to educate the patient. And also, it takes time for patients who (have) also come to the polyclinic, that you manage to divert, just to feedback to future patients. I don't think that at this stage, it can be so easily done.
M2	Have you seen successful models out there, like, you know, we know that diabetic care is always being pushed out of acute care hospitals too to be seen as APN (Advanced Practice Nurse) in polyclinics or whatever. Those seen in polyclinics, do you think we can learn from those for survivorship?
C	It takes a while for us to actually decant some of our patient care to the nurse. We started our model like a few years ago. Initially, when we were trying to introduce patients to see our nurse three months later - just follow up for those very stable patients - they were quite reluctant initially. When it comes to the appointment time to see the nurse, they just don't want and they want to see the doctor. So, it's a lot of wasted slot(s) with the nurses, and eventually, they still come back to us. So, it takes a couple of years (of) training, and maybe feedback from patients themselves, that finally now we can put out a few patients.
M2	That's a very interesting point.
B	I'm B. I also *[trails off]*. With regards to barriers seen in patients in the shared care model, I find that with regards to the psychosocial issues, since we don't have access to a psychologist in primary care, this is going to be a disadvantage, IF a patient, for instance, who's a cancer survivor, has depression. It's supposed not to be a very complicated case, I mean, PROBABLY we could manage, but because of the lack of access to the psychologist, it becomes a bit more challenging.
M2	How about other allied health professionals? Let's say, physiotherapy -
B	We have a physiotherapist too. *[M2 clarifies, "Yes?"]*
D	D. The MSW (Medical Social Worker), we only have one.
M2	Okay, one per polyclinic?
D	No, shared.
Likely F	She goes to other places as well. She's only here one day a week. Her appointment slots are ONE DAY per week.
M2	I see.
Likely C	MSWs (Medical Social Workers) are very important, because we have a lot of financial issues.
M2	Yes, we identify that as well. Okay, So, the correct MSW Medical Social Worker) will just, based on referral status, if you want to review and refer a patient, the

	person will have to see the MSW (Medical Social Worker), I mean, not even on the same day?
Likely F	Oh, definitely not! Yes, weeks or months away! *[laughs]*
M2	Okay, okay, but let's say, so let's exclude those people…, because we wanted to make sure that they are relatively well when they come to the polyclinic to see you all. Let's say, we're taking out *[trails off]*. I hear from all of you (that) so, we have to take out people with psychological issues, financial problems, perhaps they are very needy, if they have these sorts of issues, they may not be the right candidates to refer. Can I summarize like that?
Likely C	Also, we have heard from Tan Tok Seng (Hospital); we have heard from my colleagues, and A and B, that they always run into patients (whereby) their malignancy is sort of like "by the way" kind of thing that they look after. *[M2 clarifies, "It's in the background?"]* Yes. So, if we have chronic diseases to look after, such as high blood pressure, cholesterol problems or diabetes and all these things, and then, on top of that, we have to go through the malignancy issues, it takes a lot, a lot of time.
M2	I understand. But then, how would you prioritize these problems? You would still think that you would hope to handle the primary care problems first, rather than the cancer-related issues, is that correct?
Likely C	I believe in the beginning it would be that.
M2	Okay. Okay Any other perspectives?
E	I'm E. So, if it's also dependent on when the patient come(s) to see us, where is their complaint, like, what do they want. So, usually… this is what is the patient's presenting complaint (that he) is just talking about, for example, he is more concerned about the URTI (Upper Respiratory Tract Infection), and then the cancer is a "by the way" (issue), just (managed so that it) doesn't cause any trouble, then I think it is appropriate that we just focus on the URTI (Upper Respiratory Tract Infection). But of course, we will also look at the patient and we do an examination for him. Just as mentioned, when the patient come(s) in, he may just be focused on URTI (Upper Respiratory Tract Infection). This is what he is concerned about. But when I look at it and it's definite, and I know he has some carcinoma, then definitely I will question about cancer and all these treatment(s).
M2	So, that means, while you are managing the other issues related to the survivors, the cancer bit will still come back to you, because… again, you will STILL want to make sure that if there are any lingering issues related to you all, you will also look into it as well? Okay, okay. Okay! So, the next part of it is (that) I wanted to find out a little bit more about HOW should we communicate information to all, because I think this is interesting because a lot of providers out of NCC (National Cancer Centre) always feel that they don't get enough information from the

	specialist centre, in this case, NCC (National Cancer Centre). But of course, we're a little bit different because we're polyclinic, so you all have NEHR (National Electronic Health Record), you all can look at all the records. For the handover bit, what do you think is important that we should incorporate? Are there things that you think that are useful and we should also provide? Or do you think whatever that's in our system, like Clin-Doc, you guys think that's a question?
F	I'm F. So, I think there has been a review, if the proposed model, I mean, if we want to have this shared care model, I think it's very important for the hospital to give us a CLEAR plan and have a CLEAR overview of what is expected from the cancer point of view, and, I mean, the management, because when the patients come to us, we actually know what's going on, what we need to do or what. At the moment, sometimes they come with nothing, and then, we just have to -
M2	*[Crosstalks]* – so, what is that "nothing" that you worry about?
F	*[Resumes]* – No plans, no instructions for us to monitor or to follow up, or sometimes, patients lose their forms, lose their memos - *[M2 interjects, "That happens all the time."]* Yah, correct! So, the new Clin-Doc thing, I think most of the memos are inside, PROVIDED the specialists still use memo. If they still use the old one, then if the patient loses it, that's it - we can't see anything anymore. So, only if they use the one that is attached to the Clin-Doc, then we can read (it), so that's very helpful. But - I don't know – maybe (there can be) some big over(view), some big master plan *[laughs lightly; M2 laughs too]* somewhere that we can see what needs to be done and want needs to be followed up with and all that, then we won't be so hesitant, like every time I see a patient and she just finished chemo(therapy) or something, … and then she says, "Oh, numbness here and there.", I'm like, "Yah, I think it's probably due to THAT, I THINK!". I'm PRETTY SURE but I'm not like HUNDRED PERCENT sure. I can't like hundred percent tell the patient, like, "Yah, yah, it's just that and nothing else, don't worry!", you know?
M2	So, what do you worry about this model of alternating appointments, so let's say, you know, do you see the person, and then, next time the oncologist sees, and then the patient comes back to you, so how do we make sure that the information is being communicated appropriately? Because like you said, if you managed somebody's numbers, and then, next time she is seeing the oncologist, and then that person comes back to YOU, let's say three months (later), what *[trails off]*?
C	I think that the oncologist needs to know our limits, like what *[tries to say another participant's name and stops herself; M2 laughs]*. Sorry, I'm C. *[M2 replies, "It's okay."]* Some of the patients come here with side effects of this post-chemotherapy. Oncologists see this kind of side effects day in day out, (so) it's common to them, they are not worried, but to us, we don't see them day in day out. If they don't communicate (this) to us via the letter or what *[trails off]*. It is important also to mention to us that "I've seen this patient with this numbness and so forth, and the plan is this, this, this.", so that when the patient(s) come to

	us and, again, say that they've got this numbness, we know that this is being noted and being told to (the) patient what to do, and so we're not too worried about it. We can reassure the patient as a HELP to the oncologist as well. And also, this (arrangement) teaches us not to be too jittery about what we see post-chemotherapy or to(wards) ... cancer survivor patients as well. So, (we learn) not to brush away those side effects, and (we learn about the) chemotherapy side effects that you see or don't see it here before.
M2	But do you think most of these (are) because you are not comfortable at this point (with) what are you expecting, so let's say if the education bit comes in, and everybody sort of aligns with what you are expecting and what kind of patients do you see, would you be more comfortable in terms of *[trails off]*? You think so?
A	A. So, since you are talking about the proposed shared plan, *[M2 replies, "Yes."]* there (are) some difficulties that my colleagues (have) just let you know about (and it is) that sometimes... there is a gap in the communication. So, (for) some shared programmes that are going to be implemented, they actually have care coordinators, and the care coordinators actually communicate with the primary care physicians about what is happening in the hospital, so -
M2	*[Crosstalks]* – so, you think that there should be another bridge, so let's say, a navigator who is following through (with) these survivors?
A	Yes.
M2	And then, talking to both sides?
A	Yes, so that we don't depend on the patient himself to bring us the memo, and we don't depend on the IT (information-technology) system, whether it's working, whether we can see the documents, whatever the plan is inside, memo or what. And so, we already know that this helps, because before the patient come(s), primary care physician knows, "Okay, this is the patient. He has this particular problem, and then, can control this treatment. He's on this particular medication, which CAN have potential side effects, and this is what we are going to monitor." and all these things. So, rather than just walking to the clinic, there is a plan discharged, where we take over from their side (from) the coordinator, and we think can overcome some barrier(s).
C	C again. The other thing is, breast cancer survivors, after chemotherapy, they don't need to have bone mineral density test regularly, am I right? *[M2 replies, "For some patients."]* So, I notice on (this form), they say that we will continue to monitor for cardiotoxicity. So, will the oncologist expect us to continue two-yearly bone mineral density (test) for them? *[M2 replies, "Yes."]* But we will need access. How do we request for all these -
M2	*[Crosstalks]* – when you say "access", you mean, "how to" (or) like, meaning, "where" is it being done?

C	Yeah, because we don't order bone mineral density for all our patients here.
M2	What about for osteoporosis patients?
E	If we want, we refer to SGH (Singapore General Hospital) at Nuclear Medicine. *[A few participants reply concurrently, "But the report comes to us. We order (any)where. For osteoporosis."]*
M2	Okay. That's interesting, okay, because I would have assumed you have a big population of patients that have osteoporosis, but you just refer out and the report comes back? (How about) mammogram?
Likely F	Mammogram, we have here.
M2	Mammogram is here? And then, how fast is the turnaround?
A few participants	Two weeks.
Likely F	But we don't do diagnostics – just the screening one. The patients themselves get the results posted to their house.
M2	And it doesn't go back to you? Oh! But then, even if you are the primary care provider?
A	Yes, we can order. Sorry I'm A. We can order if we think that there's a reason that we want to, so we can order a DIAGNOSTIC mammogram as well, but the diagnostic mammogram is charged at private rate for the patient. It's not under subsidy.
M2	Not subsidized, unlike the screening?
Likely C	Things like bone mineral density, when we refer, (it is to) Nuclear Med(icine) at SGH (Singapore General Hospital).
M2	Oh! At SGH (Singapore General Hospital)! I see.
Likely C	Because we are referring directly.
M2	I see, I see. So, how would you advise somebody with osteoporosis and needs a follow-up with you guys?
Likely C	So, we'll refer to either the Endocrinologist or whoever first, and from there, they order. If you refer direct to Nuclear Med(icine), we have to counsel the patient for the cost and they have to come back here to see the report.
M2	Sounds like we need some health policy change, right, if we really want to push some of these (through)? So, how about physiotherapy or some other (therapies),

	because you know, a lot of cancer patients will need nutritional support, didactic support and things like that, (so) would it be the same as well?
Likely F	Oh, we have.
M2	Yeah, but in-house dietician? You have an in-house physiotherapist -
Likely F	*[Crosstalks]* – recently. Very recently! *[laughs]*
Likely C	I have to clarify also (that) we have to check with our physiotherapist first on (whether) his team (is) comfortable *[M2 interjects, "With managing cancer survivors?"]* Yah!
M2	Okay. Wow! So, this is why we do qualitative studies, right? Can you imagine if we do surveys, you all will check off, "Yeah! Great idea!", but we'll never, ever figure out all these problems. So, any other barriers that you identify? I know you all only have a few minutes left.
B	I'm B. So, like what C pointed out, the specialists also, sometimes they are not sure about what we can or can't do. For instance, there are some memos to us to trace the histology and follow up (and) I don't think it's very fair to us, for instance, if it's breast cancer survivors and there's some other lump, like, you know it's something dysplastic or *[inaudible; 37:06min]*, it becomes very *[trails off].* The referral has a waiting time, so we've had patients who come up - real cases where they've had a colonoscopy, and we trace their histo(logy) and there's some adenomas (that) went through the next scope, and we may not be the best person to advise. So, I think the institution or NCC (National Cancer Centre), also, they have to know what are possible.
M2	So, that implies, if that's the case, the oncologists would need to have an additional POINT to follow up with this histo(logies), because they can't expect you all to just pick it you? *[murmurs of agreement from the participants]* Okay. Okay, let's move on a bit. You know, I want to talk a little bit about the relationship between the stakeholders, because, you know, who are all the people that YOU THINK THAT we should engage for this model, and to make sure we have seamless coordination, as well as transition of care? I know (it) sounds like you guys are the starting point, because you will be the ones seeing the breast cancer survivors, but who else do you think we need to communicate (with)? Do you think the nurses, do you think the people around us, who do you think are the other stakeholders?
D	D. I think maybe you can have contact from a care coordinator… if you need any clarification from risks.
M2	Okay. So, you think that the care coordinator's work is just (to be) a point of contact? Like, just so that ANYBODY from the polyclinic can clarify about our patients from NCC (National Cancer Centre)?

C	I think that will be a good idea. C. A care coordinator is someone, (to) … email, that we can send to about the patient, and then the care coordinator can look through who is the consultant or specialist looking after the patient, and what are the questions, and then, send (an) email back to us, so that we don't have to go look (for) "Who looks after this patient before? What is the email address?". And then, that takes a lot of time.
M2	I'm sure NCC (National Cancer Centre) feels the same way because there are so many of polyclinic doctors as well. I can imagine that the communication can be quite challenging. Any other stakeholders, you think, beyond care coordinators? Do you think nurses, at this point in the polyclinics, are ready to manage these things? *[Unidentified female replies, "Unless they are trained."]* Unless they are trained?
C	Yes, like I said, it takes a long while for us to actually decant some of our stable patients to them, and NOW they are comfortable with all these patients. But if we were to ADD ON cancer patients when we ourselves are not yet really comfortable with it, I think it's too huge (to deal with).
M2	I can absolutely understand. Other perspectives? So, we talked a lot about resources already, because you are saying you need to refer to other people, and you know, SGH (Singapore General Hospital) Radiology also, and of course, for other issues. How about stakeholders like Singapore Cancer Society, for instance? Do you know what are the things they can offer for our community survivors? Do you know anything about them? Nobody here? *[laughs]* What about NCC (National Cancer Centre) perspective, like support groups and things like that? Do you know anything about it?
Likely F	Yah, support groups, I think, would be helpful. I think support groups -
Likely A	*[Crosstalks]* – already, we have, right? I think, support groups with the cancer survivors, already we have, right? Cancer support groups for all the cancer survivors, where they go to the forum or talk about their experiences (and) all these things? Yah.
M2	But what about community rehab(ilitation)? Do you have any experience(s) or do you know anything about, like, let's say if somebody really needs rehabilitation, where should they go?
Likely F	Community hospitals or day rehab(iltation) facilities?
M2	I see. So, you think at this moment, a network of, so to speak, support, is still quite slim, right, at this point of time, if we really require (them) for survivors?
C	Essentially, – C – we have a lot of other health issues of our patients. Some of them are eldercare, some are them are financial difficulties, some of them are post-stroke patients. These are the things that (are) already giving A LOT OF things to think about, headache, and we are sourcing out community services just to help

	them, peg them, and make sure that they take the medicine correctly, that someone is looking after them, that someone is bringing them to a clinic. So, regarding cancer survivorship and rehab(ilitation) and all these, I think it will be good if someone can come in and just ask, “What else is available for survivors?”.
M2	Should everyone do it, or do you think it should be, like, dedicate by clinic on a weekly basis, or all the cancer survivors be screened to one of two clinics THAT WEEK, or do you think, like, everyone should juts see? Like, you know, you would see somebody with stroke issues, geriatric issues, you know what I mean? And it would make it so clean. Is that a better way to go (about it)?
Likely C	It’s a bit difficult, if they come here with chronic diseases AND cancer. How do we streamline? What if one day, they come in for PHL *[requires verification; 42:48min],* then, the other day *[laughs]*, they come in for their cancer -
Possibly F	*[Crosstalks]* – yah, patient wouldn’t want that.
Likely A	No, but I would say that if you have a special clinic, and you manage this patient, like, we do have family physician clinics and all these things, that particular family care doctor who can take of everything for this patient. But as mentioned, as a general clinic, I don't think it’s possible that a person just walk(s) in and the doctor has to go through everything (starting) from… square one, and then go through that. But if there’s a family care physician who is already known to the patient, and the doctor knows about the patient, it’s easier to access. And then, if a particular appointment needs to be made, it’s like a special clinic for this particular patient -
M2	*[Crosstalks]* – are there models out there right now in your polyclinic?
Likely A	Primarily, we do have, like a family physician clinic where complex cases are managed, and - *[M2 interjects, “What case?”].* Complex chronic medical condition. And we have the MDT (multidisciplinary team) – what is it – *[Unidentified female replies, “Chronic dementia.”]* dementia, we have dementia clinics as well.
Unidentified female	ACP? Advanced Care planning?
Likely A	Advanced Cancer Planning, yah. And then, we have the MDT (multidisciplinary team) where we discuss the complex care, the social, the psychosocial, as well as the chronic problems that we manage. So, currently, we do have all these as well. So, I think we can propose to slot into such a clinic.
M2	I see. But when you are talk about having specific, let’s say, dementia clinic, it’s always run by the same primary care providers or is it like all of you would participate?
Likely A	That’s specific doctors with specific training.

M2	That's interesting, because obviously, there is only one of the disease specialties, because there are SO MANY that want to decant out of hospital, right? You talk to MOH (Ministry of Health) over the chronic illnesses, they are all - *[trails off; a few participants laugh]* So, are we going to have, like, specialty clinic in Osteoporosis, one for Endocrinology, when and how that will ever happen. But are you *[trails off].* Maybe just to conclude because we are running out of time (in) the last few minutes, are you excited and motivated to hear about their shared care model? I mean, tell us if you are not motivated or you are not excited. I think it's important because we need to get your honest feedback. I can start this way. What would be your motivations behind it, if you are motivated?
Likely C	Because I'm already treating a patient that comes with problems, so I'll also treat the patient naturally?
M2	Okay. E?
E	I think it's good for the community… as more and more patients also get cancer. So, if we can play a role to help them, then it will be good, but before we see a good effect there, then we will need to go through a lot of difficul(ties), for example, challenging our knowledge, our comfort level or our counselling (for) patients, our change in... whatever facilities (at) our level, we have to look into all these.
F	*[M2 prompts, "F?"]* I agree. So, I'm also motivated, provided I get more time. Yes! If you give me a lot of time, I can do anything for you! *[M2 laughs; F laughs too]* No problem! Okay - I mean I don't know - my colleagues, there are some in the US (United States) and they are also family physicians and I tell her how many patients I see, and she nearly fell off the chair. She sees only like EIGHT TO FIFTEEN A DAY! A DAY! I'm like, "WHAT! OH MY GOSH! So, then, I can manage the patient holistically, like PERFECT HUNDRED PERCENT! But now, with five minutes, no way, SORRY!" *[laughs]* It's very difficult!
A	*[M2 prompts, "A?"]* Yes, so it's an ideal project, so I think yes, I agree I'm motivated, but yes, I (also) agree with my colleagues that, yes, the resources need to be there, the time needs to be there, more services need to be available, and of course, we need to go through the training as well. It's a good plan because primary care clinic, polyclinic and General Practitioners are easily accessible for the patient and they are within the (region), for example, this resident is around the area to come in and see (us in) the clinic, so it's easy to make that appointment an all these things. I think the waiting time in SOC (Specialist Outpatient Clinic) is much *[trails off]*. Although, I think that they think the waiting time in polyclinic is much longer, but it's not, compared to the SOC (Specialist Outpatient Clinic), so (with regards to) patients, I think it's a good thing, but of course you need to change the system around the clinic as well. You need to get all these in the clinic, before we can proceed.

D	I'm D. I'm enthusiastic about this project and I hope that… for us to roll (this) out, NCC (National Cancer Centre) can train us with regards to the patients.
C	C. I would also like to help, but we need a few things *[laughs lightly]*. We need, at first, when you send the patient over, please don't send us very complex patients first *[laughs lightly]*. Those that are really okay one, and not a lot of other health problems PLUS cancer, for us to look after, because we also need to follow up on our nurses. And also, if we still need to refer the patient back, we have to have a way to do it. *[M2 interjects, "A mechanism?"]* Yes. And also, a care coordinator, like B suggested, that will be very good. Then, there are things that we need to ask oncologist, and we seriously do not have the time to actually look for emails or doctors? So, I would like to help, but help us too *[laughs]*.
M2	Yah, because polyclinic seems to be a natural way of *[trails off]*. You know, it CAN take patients because you are belonging to the same healthcare system. We will also try and explore whether we can decant to also GPs (General Practitioners), in fact. And of course, we have another stew of problems as well when it comes to General Practitioners. So, I think there are problems. We'll just figure out how to overcome it. BUT I want to thank all of you for giving us a lot of very good perspectives today. And M1, do you have anything you want to find out? Anything you want to add? You are okay? Okay. Thank you! Thank you! Yah, really, really! This is a very active discussion group.
M1	I stop the recording?
M2	Yah. Thank you! Thank you everyone!
	[Audio recording ends at 49:37min]
