# Supplementary material for: Implementing a community-based shared care breast cancer survivorship model in Singapore: a qualitative study among primary care practitioners
Source: BMC Prim Care. 2022 Apr 8;23:73. doi: 10.1186/s12875-022-01673-3 (PMC8991467; doi:10.1186/s12875-022-01673-3)
Supplement: Supplementary file 3 — Additional file 3. A compressed folder containing the raw data transcripts and demographics data collection form. [file 12875_2022_1673_MOESM3_ESM.zip › Supplementary Information File 3/FGD (10.20.2018).pdf]

<u>Transcript for FGD 20th October 2018</u>

Key:

Moderator / Interviewer:	M1
Respondent:	Participant A (A)<br>Participant B (B)<br>Participant C (C)<br>Participant D (D)<br>Participant E (E)<br>Participant F (F)
( ):	Paraphrases, additions to or rectification of grammar, vocabulary and/or truncated sentences.
[ ]:	Non-verbal, e.g. *[xx laughs] [pause]*
…:	Removal of false starts, repetitive or ungrammatical long phrases
CAPITAL LETTER:	When there is a louder emphasis or stressing on a particular word or phrase

M1	Everybody, got coffee over there. *[laughs]* You have the topic guide? You don't have, is it? It's for you to *[trails off]*. You can scribble on it, make some notes. We have people who talk halfway and then they say, they don't know what they said *[laughs]*. So, feel free to *[trails off]*. Do you have topic guide? Okay…. So, I'll just go through this one first, because I don't have a computer here, so I'll just use this, just an introductory talk, just talking about what is the discussion today. Oh, we are talking about this group of breast cancer survivors that have completed their treatment and who are now cancer-free, but they may still be on hormonal adjuvant treatment, because nowadays they actually tend to be on it for about seven years. So, the current model survivorship care in Singapore, we think that it's fragmented and the main contact point is at diagnosis, for primary care physicians referring to the cancer centre. And because of this, this has result(ed) in unmet needs for the cancer survivors after they have finished their active treatment. So, this proposed shared care model which we have here on the cancer journey is what we actually thought of before we started this focus group, and we thought that the role of the oncologist (will become) smaller maybe five to ten years after the cancer, (while) the role of primary care physicians will become bigger, and every primary care physician can manage. But from the focus group(s), we realised that, they told us that, it's not for everyone, so we came up with this new model *[laughs]*. So then, because there (are) a lot of barriers, so ultimately, it's where the patients find their medical home, and perhaps, at different parts of their cancer journey. But we do hope that all primary care physicians can provide the health promotion part and to recognise the red flags, and hopefully, after that, to refer on. But (for) the trained family physicians, I think many of them in polyclinics can manage them as a whole – that means, manage the cancer part as well their comorbidities – and perhaps, (to manage) a smaller group for shared care, because shared care is very intensive, so although we keep saying "shared care", from the focus groups, a lot of them tell

	me that shared care is a failure *[laughs lightly]*. So, maybe you all can share whether you have any experience with shared care later… But then, we do know that this group of patients that keep coming back to the hospitals, to the polyclinic, often has a lot of concerns, so maybe the shared care is for this group, where we really need to hold their hands. And why we need community survivorship is (because) based on some papers from overseas, they find that the breast cancer survivors actually have a high risk of chronic diseases, and because they have a poorer compliance with their comorbidities because of their preoccupation with the recurrence – they're always worried about the recurrence and they think that the control of diabetes and all those are not important. But at the same time, when they come back with recurrence and all those, you know, if they have poor diabetic neuropathy, often it limits their choice, that means, the oncologists cannot use all the taxanes, which are actually quite good for breast cancer, so they don't really realise the importance of this. And the cancer recurrence, they noted overseas that it happened in between visits, (and) no matter how often they see the patients, ultimately, they will still go to the primary care with their problems. And oncologists, because they spend so much time treating acute cancers and keeping up with the advances, they really have no time or may not have the interest, so they may not be the best persons to look after the survivors. So, today's objective will be on the perspective of survivors, so we would like to explore acceptance. (For) primary care physicians all the same, when do we actually accept the shared care and when do we not, and we hope to spend a bit more time on the proposed solution(s). Okay? Can ah? So, thank you very much for coming today. So, we'll keep it for sixty minutes or less. So, we'll go one round first to introduce yourselves. We can start recording. Started already? Thank you everyone, for coming to our focus group discussion today. We have a topic guide with the six themes here, but feel free to share your own experiences. In the first theme, it is on the background survey on your current practice and the question is, "Can you share with us some of your experience with cancer survivors?". So, you don't really need to limit it to just breast cancer. You can also share whether you have encountered other types of cancer, like colorectal or prostate cancers. I think we'll go round, starting with A. Would you like to introduce yourself, where you are from, and then, what is your experience with cancer survivors?
A	Hi, I'm A. My name is *[omitted for reasons of confidentiality; unidentified male replies, "No name!"].* Oh! No name? Sorry, rewind. Participant A, yah. Do you need my current place of practice also?
M1	In a public polyclinic setting in the west area.
A	I'm in the public setting, polyclinic (from the) western cluster. So, we don't really see too many patients who are cancer survivors in the polyclinic setting at this time, although there's currently some proposal to get some of these partnerships on board with the restructured hospitals in NUH (National University Hospital) and Ng

	Teng Fong Hospital, so we're starting off with colorectal cancer on a GP (General Practice) partnership.
M1	Okay, thank you A. How about B?
Be	Hi, B is here. I also work in the public outpatient clinic setting. My experience with cancer survival (is that) I believe for our regular patients who come back and see us, there's a small proportion of them that have previous cancer before, and even some of the patients (are) doing regular screening, for example, mammography, they may have previous breast cancer in the other side. We DO see them, (but) most of the time it's not for cancer-related issues, but it's actually more on the acute complaints or the other chronic diseases that they are suffering (from).
M1	Thank you. C?
C	Okay, hi I'm C. I'm also one of the doctors practising in a public institution from one of the polyclinics in the northern side. So, yes, we do see cancer patients, survivors in the polyclinics, but we don't see them often. Perhaps maybe one week, I see about three or five (patients), up to around that range for patients with cancer. Most of the time, with regards to the cancer that they are suffering (from), they are survivors and they are on long-term medicine, they come and see us most of the time for acute conditions. And sometimes because of the volume (in) the polyclinic, you have so little time to check their past medical records in NEHR (National Electronic Health Record), you just assume that they are fine. And some of the patients don't tell you that they are on long-term, chronic chemotherapy or even some cancer medicines which we tend to actually overlook at times. And of course, if they come for acute conditions where, perhaps, it could be a new recurrence or whatever it is, if we don't ask them, they won't tell us with regards to whether they have cancer, so we have to put all this in mind that we have to ask whether there's any history and they have cancer previously. So, yes, we do see, but it's more like for acute conditions. (For) chronic conditions, yes, they come but not for their cancer drugs. And when they come for any acute illness or diagnosis, then we tend to ask if it is related to any recurrence of their cancers.
M1	Thank you. D?
D	I'm D. I'm a doctor in private practice. My clinic is in the western side of Singapore. It's a multi-doctor clinic, which is part of a larger chain, and we cater to both the local and expatriate community. In terms of cancer survivors, we do have a group of cancer survivors which we manage (and) they tend to be those that we diagnosed initially, that means, we pick up the cancer and we refer to the oncologist, and THEREFORE we continue to co-manage, OR (they are) patients who have already been with us for a long time for various chronic conditions and we continue to care for them. Offhand, … cancer survivors that we commonly still interact with include breast cancer, prostate cancer, lung cancer, thyroid cancer and colorectal cancer. And (for) these patients from our clinic, … we tend to co-share more with the private sector, even though we are located equidistance from a public hospital and a

	private hospital. And the private specialists tend to refer back to us to manage certain conditions that are not critical to the actual disease, for instance, not an acute relapse but it is some mild comorbidity, like lymphedema of the arm, following a mastectomy, for routine surveillance. So, that is our experience.
M1	Thank you. So, D, can I clarify that in private practice, the specialist actually refers back to you?
D	Yes, they do. For instance, (for) prostate cancer that has been treated already, often the patients prefer to do the blood test in our setting, so the patients would actually do the blood test, and if the results are abnormal, then they go back. So, in the private sector, we tend to have a little bit more of the kind of interaction where we do part of the surveillance, because it's easier for the patient to go down to our clinic than to go to a (hospital), and there's also the perception that it's more affordable to do the test in OUR clinic than in the private sector in the hospital setting.
M1	Okay. Thank you. How about E?
E	Hi, I'm E. I practice in the polyclinic in the east. With regards to my experience with cancer survivors, my polyclinic do(es) see some patients with concomitant cancers as a comorbidity. However, these numbers are not high. With regards to the cancer that they are suffering from, it is usually breast cancer, prostate cancer and colorectal cancer. Some are in remission stage of monitoring, and some are still on active treatment. Very often, when they come to my clinic, they are usually seeing us for issues or following up of their chronic medical issues, such as diabetes or hypertension. And with regards to their mental state, I would say that quite a few number of them – but I would say it's not a majority – have anxiety about whether their cancer will recur or not, and they have voiced these concerns out during the consultation.
M1	Thank you. That is an interesting input. We also have a similar discussion in previous focus groups about anxiety and mental health issues, so may I ask you, if they do present to you with this anxiety and depression, would you go deeper into it and manage it in the mental health area as well?
E	So, first of all, I would try to allay their concerns, asking (them) why they are particularly concerned about this. It may be because new symptoms have developed, or it may be because they did some blood tests, the CA marker (cancer marker) or the PSA (prostate specific antigen) marker were up-trending. So, I will manage them accordingly, review them and do a physical examination on them to see whether there are any alarm signs that would need a(n) appointment with the oncologist to be brought forward or not. But more often than not, in such patients, we also tend to delve deeper (into)… how they are feeling, how's their sleep, how's their appetite and all that, to make sure that they don't have any concomitant

	depression. So far, all those patients that I have seen have a bit of anxiety regarding their issues, but not amounting to major depression.
M1	Thank you. How about F?
F	Okay, hi I'm F. I'm working in a polyclinic in the east. So, my experience is that, I think, superficially, I think a lot of people perceive that the number of cancer survivors in the polyclinic (is) quite low, but actually I found that it's PROBABLY related to the fact that because now we are using electronic medical records and… when we talk about the relapse survivors, when the cancer has been in remission for more than ten years, it's often not in any records. And I find that patients, once they have passed a certain number of years, they kind of disregard that part of the history and take it as that they have no cancer, so until you intentionally probe, the history doesn't really come out. So, I think the number of patients that we have in the community probably is a lot larger than what we do know about, than what we see. In terms of the type of cancer survivors, where I work, I think the most cancer that I've seen for cancer survivors is colorectal cancer. We do have a few previous patients with thyroid cancer that are still on thyroxine, and I haven't seen as many breast cancer survivors in my line of practice where I work. In terms of the mentality, I think there is a barrier, because I think for patients who are recently diagnosed or recently treated, they prefer to go back to the oncologist for any management and they rather not share with the primary care doctor. And then, as I shared earlier, for patients who have been in remission for very long, they also don't want to share it, because they don't think it's part of their history, they don't regard it as part of their history.
M1	Thank you. Let us go on to the second theme. We will then discuss the perceived barriers of the proposed shared care model. The question is, "What are some of the barriers that you can foresee with this proposed shared care model? You can discuss in term(s) of patient-related, physician-related and healthcare-system-related.". May I invite A? I understand that in the public polyclinic, there are plans to do shared care with the tertiary hospitals and you are saying about colorectal patients. Can you share with us how do you implement this, what are the barriers and how you plan to overcome this?
A	Okay, so, hi I'm A. Currently, we have some oncologists who came over to polyclinic to give us a brief outline of their form of a survivor care plan that they have for the patients that they are handing over to us. So, there is a limit to the number of patients that will come over first and most of these patients must be cancer-free for about five years or more before they can be safely discharged back to the primary care for follow-up. But I think some of the issues that have been highlighted to us, including those that have suggested by the oncologists, would be that with, for example, for patient-related issues, (it) will be that of trust of the family physician. Now, we do not have a *[trails off]*. Although we run a team-let based model, but we still have a lot of rotating doctors through a team-let, therefore it's quite difficult to establish a close rapport with some of these patients who needs a

	little bit more in-depth care, and for the next doctor to actually be familiar with managing the patient's needs. So, I think one of the issues that was highlighted by a patient who want(ed) to be referred back (was that) from his personal experience, … he found that the doctors here are not too familiar with managing their needs, and sometimes we miss the timeframe to actually refer them back appropriately, for example, post-colorectal cancer patients, patients who have survived (and) that (are) require(d) to go back for follow-up for colonoscopy. We DO have handover notes, but sometimes that can be missed as well, or some people clear it accidentally, and that can pose some form of a danger to the patient as well. I think for physician-related, when we survey our own doctors, some of them still express concerns that they are not too familiar with managing them, especially those that require, for example, very specific treatment. For colorectal cancer, one of the things that was highlighted was that they are not comfortable with prescribing three months or six months' worth of Loperamide for patient(s) who still experience diarrhoea following a colorectal cancer or a surgery following that, so they tend to write them a memo back to the oncologist and ask them to top up from THEIR side, while we manage only their hypertension or diabetes. So, that (were) some of the struggles that they will experience. For healthcare-system-related, I think because now there is still a lot of lack of visibility, so partnerships that (are) across systems, for example, ours is in the western cluster, we can't see very well, like, (medical records of) patients who visited Singapore General Hospital or Singhealth clusters, unless they walk in with a physical form. And most of the time, patients do not bring their physical forms over. So, we do not know how real-time or what has transpired between in the few months that they have been seeing us or seeing the oncologists. So, these were some of the barriers that were highlighted.
M1	Thank you, A. So, can I understand how long has this shared care programme been going on?
A	It has just been started for one, two months.
M1	And what is the number of patients they are planning to decant to the polyclinic?
A	It's not much. I don't have the absolute number, but I think probably about five to ten a month.
M1	Five to ten? Okay, so that means you have experienced the shared care programme yourself?
A	No, they have not come up with the actual form with the patient, but it was all in the pipeline. They have given us one or two to try to start this programme.
M1	So, having seen some of this shared care, I mean, do you feel that it's something good to do, to continue or whether it is not very feasible in the polyclinic setting?
A	I think it's definitely something worthwhile pushing forward for the shared care.

M1	Thank you. Can I invite any doctors to share?
B	I'm B here. I echo what A mentioned just now. I believe, for patient-related issues, besides the patients' resistance to return to us because of the concerns about the doctor-patient relationships, there's also concern about the competency of primary care physicians in handling their issue(s), which the perceived issue(s) (will) always be (present in) sub-specialised issues. The other thing (is) I think on the other hand, we do see patients who insisted (on) return(ing) to us after a cancer diagnosis or even after a primary surgery – I do have patients on stoma (bags) and trach (tracheotomy tubes) who return to see me after their surgery and still continue follow-up for cancer and non-cancerous-related issues. A lot of the times, it's about the trust, as what A mentioned, whether the patient trusted us (and) that's probably the main reason patients would or would not return to us. For the physician-related issue, besides the handover instructions, where most of the time, it's lost somewhere… either because of the fragmentation of the system, or as A mentioned, (it) also can happen when the discharge instructions are being done by a more junior staff, or there is no appropriate workflow for the discharge instructions, and therefore a lot of information, important instructions may be lost. On the other hand, the physicians - the primary care physicians I mean - also have this concern (that) with this busy schedule and work environment, how is this being feasible and whether there will be, both the workload kind of overload and burnout *[trails off]*. There will be this connecti(ion) (between) overload and burnout, because it's very hard for everyone to be (kept) updated with so many different cancer conditions, so many different drugs and side effects and interactions, IN ADDITION to whatever acute and chronic illnesses that we are taking care (of). For the healthcare(-related), besides whatever (has been) mentioned by A, I do have this concern about healthcare financing, though we are trying to do our best for a very valued and high-quality healthcare service for outpatient, including (for) the survivor patients, but sometimes it is very hard for us to justify that amount of subvention and money we receive, but the care for the patient is very long(-term).
M1	So, actually just to feedback, this is one of the first few time(s) that a public polyclinic family physician talked about financing, because we noted that financing was not really talked about, because - well, I'm not sure whether it is already told to you that – you're not spending too much time on one patient or not having the workload. Maybe I invite other doctors from the public polyclinic (to share) whether you have a problem with subvention?
C	Okay from *[trails off]*. Okay, I'm C. So, I had some experience in, or rather, I have interacted with the headquarters slightly to actually have a quite lucky – or rather - quite fortunate overlook of the system in primary care in our setting. So, the thing is that the subvention in primary care is actually very – I wouldn't say it's very – complex, but the way how it is being calculated and how the subvention is being channelled to the polyclinics (for) per-head per patient, how much the patient has been subvented et cetera, it's not usually based on how many conditions the patient has. It's not like, you have ten conditions, I pay you "(per) patient head"

	more for their subvention, so it's actually a pro-rated flat fee for everyone, so even if you have one chronic condition or two chronic conditions or even THREE chronic conditions, your subvention for that one head is similar. Of course, moving forward, they have this *[trails off]*. What do you call it? They have this plan for – what do they call it – bundle payment for the condition et cetera, but at this moment, how it's being looked into is (that) actually you see the number of patients and you still have to justify why certain patients *[trails off]*. It's very difficult to justify why you take (such a) long time for certain patients, because we also have our certain number of patients that we (need to) still clock (in) per hour, that we have to see in a certain number of hours to ensure that we do a certain workload. This is ALL related to also the way how subventions are given. So, even if you have cancer plus chronic disease PLUS multiple chronic diseases, multiple (co)morbidities, and you need, like, twenty minutes or even fifteen minutes to actually spend for the patient, it is quite difficult to justify in that sense, from how you are being seen as spending too much time with your patient and et cetera. The other thing is that, even with the implementation of a bundle payment that is in the midst of discussion these days, it's that the bundle payment is mainly for chronic conditions like diabetes, hypertension and hyperlipidaemia and et cetera. I DON'T THINK that the other chronic conditions such as cancer would be actually part of that, taken into consideration for that part, so that is how (it goes). I mean, there is this idea that, for example, like CHAS (Community Health Assist Scheme), that we have for private setting, we have "one-condition CHAS", "two-condition CHAS" and they have these tier(s). But in the polyclinic, as far as I understand, we see the number of patients as what we're actually supposed to see, and it's very difficult to spend time with these patients.
M1	Okay, thank you, C. But with the changes in cancer care now for the longer survival, actually many oncologists think that cancer is going to be a chronic disease, so do you foresee this as happening and whether we can, you know, manage (it) as a chronic disease as well? Anyone?
D	I'm D. On this trend of thought, I hold the viewpoint that cancer is and should be considered a chronic disease. Patients with cancer is no longer a death sentence. They live longer and they have other complications that we have to manage along the way. So, at present, the CHAS (Community Health Assist Scheme) and the Pioneer Generation Scheme DO NOT consider any of the oncological conditions or cancer conditions as chronic diseases. The scope is really limited to the metabolic conditions, (such as) hypertension, diabetes, hyperlipidaemia and the works, so as a result, (for) a lot of our patients who have cancers and come in for their consultation, they cannot utilize the PG (Pioneer Generation Scheme) or the CHAS (Community Health Assist Scheme) to the maximum. In fact, they get very little to no benefit. (With regards to that) mentality, that is actually a barrier for us to have a shared care model, if the PG (Pioneer Generation Scheme) or the CHAS (Community Health Assist Scheme) don't recognise cancers as a chronic condition. In the private sector, our experience is a little bit different. We have two types of

	shared care: one is partnering private oncologists and one is, of course, partnering public sector oncologists. From my experience, with the private sector, it tends to be a bit more (of an) intimate relationship, because it's one oncologist and one family physician, so often, it might just even be a text message or a phone call away, so we tend to be able to react a bit faster as well, whereas we find it a bit more difficult to contact or reach the oncologist in the public sector, often even if we ask for early -
M1	*[Crosstalks]* – even as a subsidized patient?
D	Subsidized or otherwise, even if you try and ask for (an) earlier appointment, it's often a longer waiting time. If you CALL to speak to the oncologist for a quick opinion, it's almost IMPOSSIBLE to reach them, whereas in the private sector, you tend to be able to contact the oncologist much faster, and they are more responsive, so we tend to have a more intimate relationship with the private oncologists, but less so with the public (oncologists), which tends to be a more rigid system. The other thing is that with the private sector oncologists, they tend to *[trails off]*. Because it's more one-to-one, they tend to give us a very discrete plan for THIS particular patient, (for example,) "Repeat the blood test at what interval. And if something happens, do this for the backend, watch out for osteoporosis et cetera and help me manage.", whereas at present, with the public-private partnership, it's NEARLY non-existent, or at least I am not aware of. I often don't see any formal written framework to work on, so there is often not much incentive if I'm a public-paying patient, to enter this sort of shared care, whereas I could just continue the care predominantly in the public sector oncology sector, so those are some of the barriers that I perceive.
M1	Do you have any proposed solutions?
D	At present, I feel that if they want to launch a proposed chat care model, it should be tailored to specific cancers, and the partnership may have to be a bit more intimate – just a few cluster clinics which we equip them with the skills and the technological know-how-to to execute the plan. If you were to roll out a massive partnership where you just give them a letter and bring them to any GP (General Practice) to execute the plan, usually this will be unlikely to succeed, because the receiving family physician or general practitioner may not be comfortable (and) may not have the technical expertise. And even if they want to feedback or raise a question, they tend to be unable to reach back to the public sector oncologist.
M1	Thank you.
E	I'm E. D mentioned that the private oncologists give a framework for the family physicians to follow, and I find that that is a very good thing that we can do, if we were to adopt this kind of model. One thing I want to highlight is that the doctor-patient relationship is very important in (the) management of any chronic illnesses,s and for this to happen, you need to have continuity of care in order for there to be rapport being built over time. So, in some of the polyclinics like the one I work in,

	in the family physician clinics where most of the time, it's a single family physician that is attending to the patient over repeated reviews, and this enabled us to build a good relationship with patients such that they are also able to tell us a lot of their psychosocial issues. However, we must also take note that a lot of these patients come to us with multiple chronic comorbidities, and we have to prioritize what they tell us, in order for us to focus on what is the pressing issue at hand, so time is REQUIRED for this. And in the family physician clinics, patients are given a longer allocated time of about fifteen minutes, which it is definitely better than the walk-in general clinics, so this is also an aspect that we can look into if we were to have such patients being decanted from the tertiary care. And having a framework for the physicians to follow is also very important, because bear(ing) in mind that if the patient goes back to cancer centre for their follow-up of the cancer disease, it is usually that the doctor is very in-tune with that recurring cancer and will give the next review date as appropriate, whereas (for) the patient who comes to the polyclinic, the family physician clinics have to deal with multiple problems, and sometimes detecting the cancer occurrence can be missed in this very busy clinic. So, having the time, let's say, in the family physician clinic, with (an) allocated time of at least fifteen minutes, having (a) continuity of care by the same doctor and having a framework definitely will help the doctor.
M1	Thank you, E. So, I understand that you are proposing that shared care should be located in specific clinics, mainly in the family physician clinics with a protocol. And actually, the reason why partnership with primary care is preferred is because we believe that physicians are really the best people to manage the psychosocial issues. So, in your practice setting, even with the fifteen minutes, do you think that it is possible to address all these issues?
E	Definitely, if the patient has multiple issues, fifteen minutes may not be enough, but having (a) continuity of care is beneficial in the sense that we can prioritize. We give an earlier follow-up review and we can tackle the next issue at hand for the next review and it will be handled by the same doctor, so it is STILL a better solution compared to if the patient was seen in the general clinic.
M1	How about C?
C	Sorry, I'm C. I just want to add something with regards to protocol for managing shared care. I think protocol is definitely a useful thing, but at the end of the day, I still believe that the relationship, as echoed by D, is that (the) relationship between the primary care physicians and the oncologists or the one that's managing (the patient) is very, very important. I think patient(s) will say that the accessibility to enter the hospital is difficult because you need to have appointment before you can see (a doctor) and et cetera, but the truth is that for us doctors to even contact the doctors or even to have the time or communication with the doctors in-charge of patients (in) oncology is also, I think, quite impossible in that sense. If really, I were to have an issue, I have to call the hotline for National Cancer Centre and then I have to wait for who is this patient, who is the one who *[trails off]*. Yeah! I mean,

	there is no direct line and there is no connection for the primary care physicians and the oncologists. Apart from that, of course even in the secondary care, the subsidised patients are seen by different Registrars, ACs (Associate Consultants), and similarly in the primary care, even though we have team-lets these days, the doctors DO change; the doctors do have a bit of change at times. So, from that perspective, protocol(s) will only help to a certain extent, because at the end of the day, I still believe that each patient is still quite individualised by his own treatment (and) it is best for the primary care physician to actually assess the patient. And if the primary care physician is comfortable with the protocol that's in place, then it's fine, but at the same time a protocol will not answer all questions for any of the patients, because sometimes patients come with a little bit of different manifestation (to the) primary care, and even though with protocol, it's very rigid (when) we don't really have the communication with the oncologists to make sure that… the patient is safe. At the end of the day, it's about the safety of the patient, and with the advancement, with all these issues of medical-legal, it's actually sometimes quite frightening to take the whole responsibility on our primary care side.
D	I'm D. I just want to add on to the same line of thought. It isn't just the relationship between the oncologist and the family physician, but also the family physician and the ancillary care. Right? So, in my setting, we work closely with a selected group: we have lymphedema therapist that is in private sector, who actually helps to manage some of our breast cancer survivors who have post-(treatment) axillary clearance with lymphedema; we have a dietician that specialises in post-cancer diet therapies; and we have a psychologist and counsellor that helps, because at the end of the day, if you are managing a busy primary care clinic, who might not have enough time to REALLY teach the patient lymphatic drainage massage or to monitor the patient's diet et cetera. So, you STILL need a network beyond JUST YOU. So, a shared care which involves just the primary care and oncologist is going to be very limited in both scope and ability. So, in the same line of thought, for the proposed shared care model, I feel that we should rope in the ancillary partnership members, such as the dietician, psychologist, social worker and physiotherapist – I think they all play a part and it's all part of this cancer survivorship. Right? Because it's not just about living and make sure the cancer doesn't come back; it's about living well and living healthily. Right? So, in that light, what we experience in the private sector is that we tend to have one or two ancillary (partners) that we work very closely (with), for instance, like knowing that someone is very good with lymphatic drainage massage, I would refer to you, and because it's quite close, we usually get an earlier date if you feel that the swelling is really bad. She needs an earlier treatment and we can get an earlier date as well. So, that's OUR experience. But in the public sector, if you want to refer back to a physiotherapist in the National Cancer Centre or any other public institution, it will be maybe like ten million years *[someone laughs]* – sorry, I correct myself - a very LONG period of time, and possibly

	not even get any (date), so, that I feel, are barriers to a successful proposed shared care model.
Unidentified male	I think, to add on, it's not *[trails off]*. You just have to refer BACK to the oncologist to get to the ancillary (care), EVEN THOUGH it's something that you can fix directly.
Unidentified male	Yah, correct, so that's one of the -
M1	*[Crosstalks]* - F, would you like to -
F	I think my main thing is, I think when we are talking about the shared care, the main thing is actually the patient. So, I think it has been mentioned by a few others that the patients' perceptions of primary care providers in Singapore is probably not very good, in terms of what our skills are, whether we are equipped to manage their problems. So, I think that, right now, even for patients who are referred to primary care, they are only referred after a LONG, LONG period of time after their cancer has been in remission for many years. So, when patients are referred to primary care, I would imagine that if we have this shared care model where patients are told that they are going to be referred to this primary care provider, they might feel very abandoned, like "Is there nobody that is going to take care of my cancer anymore?", or they may actually see it as a go-ahead, like, "Oh! My cancer is well enough that I don't really need to see any specialist.", so then …. because of this perception, they may be more prone to defaulting. And I think right now, at least in the public sector, when - I'm just imagining – the patients are referred to us, how can we actually follow-up (with) defaulters, because in SOCs (Specialist Outpatient Clinics), usually you have a patient list, you know who didn't come, you can review their notes and see whether this patient call(ed) back or not; in polyclinic, unless we have a special clinic whereby we have a follow-up patient list, otherwise in the usual general clinics, nobody has a(n) appointment list. Even if the patients default the appointments for many years, nobody knows. Oh, I think there's the potential of how can we make sure that the patients that we are supposed to see, don't default as well, in terms of their care.
M1	Thank you, F. So, in your setting in public polyclinic, do you have like a team-let model, do you have a family physician or specialised clinic that you can cater this group of patients?
F	I mean, for example, if you only want to refer these patients to family physician clinics, in the FPCs where we have also an appointment list and patients come. The other thing is also that we have special clinics, like our mental health clinic, memory clinic, that also have these appointment lists, but I would imagine that not ALL patients will NEED to be in the special clinics. I think even in the model you presented earlier, there are patients with very low needs that any primary care provider should be able to address bar, and then there are those with higher needs or that may need more intensive follow-up, or quite early on after their remission, they may need a bit more blood tests, they may need more frequent reviews, so

	those may qualify for the special clinics, then I think keeping track of them would be a little bit easier, but I think the main challenge would be, for those patients who are quite well that they only need to be referred back for scope once every two years, who is going to follow up (and ensure) that this two-year thing will happen? And then, I think right now in the primary care landscape in Singapore, patients are often prone to hop between primary care providers, so if your shared care is communicated to one polyclinic, but the patient decides to see his GP (general practitioner) after a few years, then, whose responsibility is it to make sure that the patient is followed up (on)?
M1	Thank you. So, can I invite, are there any doctors here who can share on their experiences with other shared care models, maybe for other conditions, and whether it was done wells?
E	I'm E -
Unidentified male, possibly A	*[Crosstalks]* - just to clarify, "other conditions", meaning non-oncology?
M1	Non-cancer patients.
A	I'm A. So far, the experience that we have is mainly with renal patients and cardiovascular patients where we do have clear instructions from the memo by the renal physicians, like "Do what, what, what at regular intervals. Refer back early if the twenty-four-hour protein urea exceeds a certain amount.", this kind of CLEAR instructions. It also provides the doctor (with) the confidence to manage such patients, so these are the common shared care model(s) that we see so far.
M1	Any other shared care conditions that you have encountered? How about dermatology?
C	So, in a *[trails off]*. We have *[trails off]*. If there's a shared care in the polyclinic, usually there is a doctor champion to actually look into certain cases for dermatology, so that's so far *[trails off]*. Anyway, I'm Participant C, I'm C, so sorry. So, as in the clinic, usually if there is a shared care, there is usually a champion doctor to look at the cases for patients.
F	I'm F. So, I think, like what E has mentioned, I think commonly we do get referrals from renal or from colorectal (specialists) after the patients have done some scope, and then maybe they just need to be referred back for scope in three years. Even for renal, it's to manage, to review the renal panel every six months, that kind of thing. So, the problem is that for patients who don't have a specific primary care provider, then it is contingent on who is the doctor that they see in the polyclinic to actually take note of that in their plan and (do a) follow up, so then, I think it can be missed, even though there is a section where you just put all the things that we are supposed to follow up (on). Sometimes, after a while, it gets messy or the doctor

	doesn't have time to review that, then there is the potential that these things can get lost. The other thing is that, because I also run the mental health clinic in the polyclinic, so the other common (issue) is that we do get patients who are referred out to the SOCs (Specialist Outpatient Clinics) when they are deemed to be quite stable, and maybe they just need long-term SSRIs (Selective Serotonin Reuptake Inhibitors) and things like that. So, my experience is that the patients who come and see us, often, after a few visits, defer, and if they come, it's because they feel that they are well (and) they feel that because they have been decanted to primary care, they are well enough to stop the medications, so I think that's the other problem… when we want to refer cancer patients. How do we make these patients take ownership of their conditions and make them understand that even though they are better now, they still need some follow-up?
M1	I was just wondering whether you agree with this, because in one of the focus group(s), a senior family physician say(s) that we can upscale our family physicians, we can change the structure, but in the end, (the barrier is with) the patients. If we don't change the patients' perceptions or the education, then all these shared care may not work. So, can invite you all to (share) any perspectives on how else we can help with the shared care? And you can also refer to this survivorship care plan. This is proposed by the American Society of Clinical Oncology. I know that it is a very LONG document; if we "protocol-ized" this into the polyclinics, how well will it work? Is it more of a hindrance or a help?
A	So, I'm A. Also, just to mention that I work in the polyclinic in the western side of Singapore. So, I think, just to mention a few points: the first part, as what E and F have mentioned, those are what we call, more "episodic" kind of follow-up. Although the patient has a continuous medical condition like chronic kidney disease or chronic heart-related issues, … the follow-up that is given up by the specialists is often episodic, like we monitor urine PCR (protein-creatinine ratio) or to monitor blood pressure and to keep blood pressure at certain targets, and if we fail (to do) so, they will be referred back to the specialists. These are still quite manageable for even family physicians, who are still quite junior, or (for) our medical officers. But what we are talking about now that is a little bit more challenging for us is those who require continuous follow-up. For example, things that lapse across a very long timeframe, for example, repeat colonoscopy after FIVE years because of these certain histology subtypes following the first colonoscopy or after therapy for colorectal cancer, or patients with… conditions that will continue to go downhill, for example, Chronic Obstruction Pulmonary Disease (COPD), so we have a few shared care programmes (and) we have the COPD (Chronic Obstruction Pulmonary Disease) nurse who comes INTO the clinic and they sit in together with us for a shared care for patients, for example, who are still not oxygen-dependent, for them to do some pulmonary rehab(ilitation) education. So, that's something that is working quite well and our patients are quite happy with that. We have an endocrine shared care as well, mostly for diabetic patient(s) who are decanted to us. And those patients often require quite a lot of titration of insulin, for example.

	(For) thyroid cancer patients, they have taken them back to them, so like the endocrine (in the hospital), surprisingly, they decided to take them back and say that they are not too safe for us to manage, because some of them still require annual, like two-yearly, three-yearly kind of ultrasound for thyroid, and they do some other markers as well. So, they took back the patients, and they only give us those that are either completely burnt out, or they are really very old and they are allowed to be decanted back to us. (For) mental health, as what's mentioned by F as well, so there are some shared care programmes that we have. I think some of the issues that I see, alongside with the shared care, the survivorship care plan program that you have mentioned, by the US (United States), is that it's very lengthy. So, under NUH (National University Hospital), they also shared a similar one for colorectal cancer – it's about five pages long – and more often than not, the information is too much. So, this is just only for breast cancer. So, they shared with us that they have one for colorectal cancer, and they have one for every other cancer that they plan to transit back into primary care, so that's all in the pipeline, and we are getting a little bit more frightened about that. I think the first part is that, if this really has to come into the hands of primary care physicians' hands, it's that we need to know the recurrence rates of cancers that have recurred, and whether they want to actually take them back, because it's no longer too safe to sit with us, because sometimes we just miss this group of more fragile patients. Certain medications that have been used by the patient(s) that are no longer effective for the patient(s), or they experience MANY, MANY side effects for long-term suppressive therapy, they should (take them back) as well, so there's no place for it as well - at least currently in the one that we have with NUH (National University Hospital) - so we have actually given them feedback. And as what has been mentioned by some of our doctors here as well, it's that there (are) no direct lines, and more often than not, it's not like in primary care where there are fields to put PCP network, primary care, the oncologists, the surgeons, the radiation oncologists. More often than not, they will just draw a line and say, call the hotline *[a few other participants laugh lightly]*. And they give a general email, like "enquiries@nuh.com.sg", so you have to email them back. Some oncologists are more on the ball, so in all honesty, they do try to give their own direct line, (but) I'm not sure how many oncologists are very comfortable with that. We started off with the mental health (programme), so the psychiatrists are on-call during the time that we are working, so they do give us a direct line with them, so that helps, in case we have some patients that we struggle in managing. So, going forward, maybe that will be something that will be very relevant and important to us.
M1	Thank you for the sharing.
C	This is C. Just to add on, I think, I mean, the time is always a limitation for us, and I agree that the summary and survivorship care plan tend to be very, very daunting for the primary care physicians when they don't have much time. (With regards to the) executing, if we have the luxury of time or we have - I won't say luxury of time (but) if we have – a proper execution from (the) operation(al) point of view, then

	perhaps it IS possible to actually look into these treatment summary and survivorship care plan. My suggestion, or rather, what I thought… would be a suggestion to be considered, is that if indeed there is a shared care, it would be good to actually have a specific day dedicated for that shared care, so that patients, say, for example, for that cancer, come on Monday to see this shared care clinic. Based on experience, currently we have dementia clinic, we have mental health clinic, we have – what else we have – … dementia mental health, and these are a few common ones, and falls clinics et cetera, we have these things. So, we have dedicated days. But also, from (an) operation(al) point of view, looking into these days, even for, say, for example, my mental health clinic, when the patients come to us in mental health (clinic), we tend to fall into the fact that we (are) also starting to segment patients in their care for specific conditions. Patient will have two, three appointments in the polyclinic: today you see (for) your mental health, next two weeks, you see (for) your chronic conditions. I mean, that's something that we actually need to look into. If patient is truly coming in for mental health, then (multiple) appointment(s) for the polyclinic shouldn't be made, but (instead, to) book and actually settle in one (visit). That's something operation(al) that we have to think about, or rather, something that that has to be overcome first, because I think that the good thing about having specific days for certain running of clinics for the shared care is actually very good, because (firstly), it helps with contacting oncologists if there is any issue, because the oncologists at least know that "Today, I will be receiving TEN calls from the primary care physician. I will be ready. I will not be bothered (at) certain times.", and perhaps (it is) even good for the oncologists – I mean, … in the hospital, they can plan (for) that day to be (doing) some paperwork or whatever (and) they are always available for the telephone call, rather than doing ward rounds et cetera (and) it's going to be difficult, (so) that's one good thing for the oncologists itself. And for us, it's good also, because when we do a specific day for a certain specialty, your thoughts tend to think faster also, because you tend to dwell in the same condition, and you tend to think faster that these are the complications that I need to think of. And honestly, sometimes, from my experience, if I run a mental (health) clinic, I think it's better for the patient who's seeing me for mental condition, rather than (another patient) who see(s) me in the general (clinic) pool. (At) the general (clinic) pool *[laughs lightly]*, I'm stressed by my clocking of the number of patients (and) I just find that it's difficult because I have to concentrate on other, other things; there will be pharmacists calling to top up medicine, to extend medicine; there will be nurses calling to (ask), "Doctor, come and do for wound dressing, wound inspection.". So, I feel very overwhelmed by these things, but if I were to do - and I DO do - health mental clinic too and when I do health mental clinic, then I tend to think better. I clerk cases better for the mental health, because I'm not (under the) pressure of time. I ask practically EVERYTHING, in fact, I would say as good as a registrar in IMH (Institute of Mental Health) psychiatry, because we have the time and because it's the same thing (and) it's a routine thing. So, I think if you were to do (this), one of the ways to do (this) is to ensure that there is a specific day for shared care. And of course when you have specific days for shared care, then our mind is in tune with all these summary and

	survivor(ship) (care plan), because the first time you see (this), (it) is daunting, but (for) the second patient you see, the third patient you see, you just flip (the page), "Oh! This one, I know. This is here. This is here." (and) I know where to find (the information). Can you imagine, if the patient just comes in with this shared care plan and he's in the general (clinic) pool, (and it's) my first time in the day seeing this, then my mind will be like, "Where is the allergy? Where is the *[trails off]*?". But if you always see this form, familiarity breeds productivity, right, they say, you just do, do, do the same –
B	*[Crosstalks]* - can I jump in from here? So, I'm B here. I have a concern when I hear all these – the concern is (that) what we are doing right now, though a good way is service redesign (so that) we can deliver all this care efficiently, on the other hand, we are bringing a disease-specific SOC (Specialist Outpatient Clinic) into the community right now, which I'm not sure are we driving the correct path (that) we are doing right now. We think that our SOC (Specialist Outpatient Clinic) is flooded with all the cancer survivors and disease-specific patients, and NOW we just build ANOTHER disease-specific clinic in outpatient setting. Though it's good for the patients because it's nearer to their home, but it's not manned by the specialists, but everything is the SAME, and we are seeing them, just like "I only see you just for mental health today or breast cancer survivor(ship) today. For your top-up of DM (diabetes mellitus) medicine, come next week.", which is very silly. *[Unidentified male interjects, "Very silly, correct."]* And this actually goes back to the money (issue) again that I feel strong(ly) about, because all the backend subvention is actually unfair, because if you do spend time for these patient(s) with both breast cancer survival, colorectal survival, DHL (diabetes, hypertension, hyperlipidemia), osteoporosis, and with fall risks and early dementia, you probably take one and a half hour(s) to do a comprehensive assessment and management, but the subvention back to you will probably be ten minutes only, and (with) what you are doing, you might not help with the organization (and) you never help with everybody. I wish I have a lymphedema physio(therapist) in my clinic. I currently only have a musculoskeletal physio(therapist), but the problem is (that) the lymphedema physio(therapy) is sort of like a sub-specialised physio(therapy) in the cancer centre, because of the service delivery efficiency in the public sector. If I were (to) refer all these patients that I see right now with lymphedema back to the physiotherapist, some of the hospital physiotherapists… are willing to see and they are happy to see (the patients), but the administrative-system-related issue is (that the) backend top-up subsidy for these patients where this patient is paying \$20 out of the \$100 cost, the \$80 bill will go back to the polyclinic, and WE have to pay the \$80 bill for the patients, but not the oncologists, (then) our boss will come back to us (and ask), "Now, \$80, count on my account or count on your account?", which is a very silly answer.
M1	Thank you.
D	I'm D. As I hear all your inputs, I want to raise a point: so, there is "shared care" and there is what I perceive as "transfer of care" or "decanted care". So, with non-

	oncological conditions, as I hear from all of you, it sounds more like less of shared care and more of decanted care. The specialists feel that the condition no longer requires the specialists' care, and THEREFORE TRANSFER the care to the polyclinic, be it psychiatrist or COPD (Chronic Obstruction Pulmonary Disease), and they almost have no more links back to the specialists, whereas in oncology, there's always a susp(icion) of fear and recurrence, so almost always, there will always be a link back to the oncologists. So, the model that we adopt in the private sector is more that you see the oncologist once a year, but during the year, the other three visits are to your primary care physician to do the other tests and the other monitoring. And in fact, often, there is always one visit to the family care physician before the oncologist, because the oncologist will then save the time and just look at the test report and review the patient at the same go. So, what I feel is that (for) the non-oncological models, a lot of them are not really truly shared care; in essence, they are decanted care or they are just transferred-out care, that means my skin centre is too busy (and) I don't want to see any more psoriasis patients, or it's just another eczema, (so) just go to the primary physician and then don't come back ever again kind-of-model, whereas in oncology, because of the endless fear of the relapse, usually it's more shared (care) than true decanted (care). It's very unlikely that they will pump the patient away with their care forever, because they often - a lot of them - require specialized test or titration; they may require a scope; they may require a PET scan; they may require other facilities that, at present, are not readily available in the family physician centre. This leads me to this form that we are looking at. This form is great, but if you look at the first section, "List the primary care provider.", and they list a lot of sub-specialities, like radio(therapists), surgeons, oncologists, medical oncologists, but in reality, there should only be one driver, and in our local context, it's often not the breast surgeon. The breast surgeon does the surgery, and it's often the medical oncologist that will be the lead driver, and then the others (are) co-drivers, so to speak. So, in this context, I feel that the form should be redesigned such that the "who to really contact", … as highlighted, should not be identified as a hotline, so that is one of my feedback on the form. On the other aspect of the form: the form is indeed very, very long, but I think the form has some pros, like they do list out some of the common side effects of certain medications, like I see Tamoxifen, the aromatase inhibitors, which is a great idea, because oncology is rapidly evolving (and) there are endless new medications, new antibodies. Unfortunately, these medications have rare side effects BUT are important to know, that unfortunately the training is not really there in our current medical education. These medications also have considerable drug interactions, of which if you are not familiar with them, we often will miss out and we might mis-prescribe. So, THAT, I think it often falls to the back of our mind. So, this proposed form is fantastic, but the only issue is that it has to be constantly updated, because there are more and more new oncology (drugs) – in fact, oncology is one of the most rapidly developing pharmacological areas that often are new antibodies and new agents, so this sort of information needs to be seen and readily available to the managing family physician. However, this form is static and it cannot evolve with time, which brings to my next point that ultimately good shared care programme

	will have to go digital, and the proposed form will have to be a digitalised form and there can be constant updates, and there could be, in fact, even a two-way communication where what we wrote can be seen by the oncologists and the oncologists' can be seen by us. So, EVEN IF we cannot REACH the oncologist or the specialist on time, there (are) still some ways of communication (and are) more official.
B	B here. To add on to what D mentioned on the digital-izing (of) the form, I feel strong(ly) about this artificial intelligence in really putting all the information in and having a processing kind of brain to really sieve out all the information, what are the important things that would flash up and serve as a reminder to primary care physicians. I feel that there are so many things that we need to cover in primary care setting, and with so many issues, there is no way for someone to remember every single thing, for people to read through, even if I digitalised all these and it's hidden somewhere in the electronic medical record system. If there is no system or logic for all these to be processed, it is a challenge for the primary care physician to handle this kind of patients also, THOUGH we can change our mind on that day, having specialized a clinic on that similar day, but this again, is against my - sort of - belief in family medicine.
C	I'm C. I do agree that if you do a specialized clinic, it will actually be actually different care, as I said earlier, are we going to bring in something from the SOC (Specialist Outpatient Clinic) to a culture in the primary care. The other thing is also (that) by doing it by a clinic-based (system), it is also (that) you don't solve the problems of patients where, say for dementia with cancer, plus COPD (Chronic Obstruction Pulmonary Disease), where and which clinic does the patient belong to? So, that's another *[laughs]* issue that you need to look at. Of course, the other thing, the other argument is to ensure that all family physicians that run any of these clinic(s) are capable to manage any of these. The clinic is just meant for the patient to have a proper time, (an) exclusive time for the patients, for the doctors to manage their condition. I mean, as long as you have dementia, you have to come to dementia clinic. Even though you have your cancer, we also will see (you for) it, but it's just a way of ensuring that the time spent for the patient is adequate, and that funding, payment will be reasonably compensated, because you are doing special clinic; you are not doing general pool clinic; or you are not even doing the normal comorbid(ities), DM (diabetes mellitus) and (hyper)lipidemia; you are doing cases of patients with dementia and plus comorbidit(ties) plus whatever it is. I was thinking that it is not more about the separating the condition, but more of stratifying the type of complex patients that are coming, so that when you plan your time, you have excuse – I would say, not excuse, but – you have reasons to say that "This clinic will take a longer time. I'm not expected to see six (patients) an hour, or even five (patients) an hour, but expected it is what the SOCs (Specialist Outpatient Clinics) are seeing perhaps – I don't know for first-time cases - I think for first-time cases, now *[M1 interjects, "Half an hour?"]*, half an hour, so one hour, two patients.", something like that, I mean, just for stratification, not to actually

	specially, just because the patient (is) having all these, (hence) just to segregate patients.
M1	Thank you. F, maybe I can invite you to share? In that case, will it be feasible for family physicians to divide themselves into family physicians, specialists *[laughs lightly]*, family physician generalists and for them to look after complex care?
F	I think at the end of the day, the idea of family medicine is really to see the patients as a whole and not as segregated parts, So, like in a patient with dementia and who also has cancer, then if there is a problem with cancer recurrence, then what are the considerations when you weigh whether the patient needs to go back, whether they want further treatment. I think that's the benefit of family medicine - that we can see the patient and we help them to kind of weigh the decision for themselves. So, the problem, the danger with segregating into different clinics is that you kind of lose that as well. But on the other hand, my concern is really about how do you keep up-to-date with the knowledge then? I think as D mentioned earlier, there's a lot of new drugs all the time, new treatment, there's new evidence (about) when patients are in remission, how long they should be on follow-up , the modality of follow-up, so if let's say you have this shared care plan , and the patient has already been decanted back to primary care, then if later on we get new information that changes the way that they are managed, … then… for these kinds of patients who are more in the primary care rather back in the oncology, how are we going to disseminate this information back to their primary care providers when they don't have a fixed primary care provider, for example? So, I think that is going to be a potential problem that I would foresee.
M1	Thank you.
E	I'm E. I think that for this model to work, patient selection is actually very important, so definitely you will want to see patients who have low-risk of recurrence, who have very clear instructions what we are supposed to monitor for, let's say they are on this drug, look for this side effect as stated here. And the things like psychosocial issues, this is part and parcel of what a family physician should do already. And bear in mind that sometimes the oncologists need to rely on certain tests, like tumour markers to be done and monitored routinely, and many of the tests in the public polyclinics are actually non-standard tests, if you are talking about tumour markers - not easily available - so that has to be kept in mind if we were to accept such patients.
M1	Thank you. Any other input from any participants?
A	Just a point *[trails off]*. I'm A. So, just a point on the digitalization: so, there are plans in the future when we integrate for the non-Singhealth partners, and potentially, the National Health Group as well as the NUH (National University Hospital) Group to move into the new-generation EMR (Electronic Medical Records). They're thinking of using prompts to highlight to the doctor what are the due test(s) to be done, for example, colonoscopy or tumour markers or BMD (bone mineral density

	test), so this will decrease the need for the doctor to remember what to put. And if you put too many handover notes, it turns out that you can no longer remember, because like what most of the doctors here have mentioned, as family physicians, we no longer just treat just one single condition; they come and see us for almost every other thing. So, it's very artificial to say that today we run oncology clinic and therefore, I will only ask you to come back for a colonoscopy, because you are due for that. But let's see if you are due for your coronary risk screen, "Let me give you another two months' appointment to come back to see your regular ischaemic heart doctor or to see your regular hypertensive heart doctor for them to ORDER in your coronary health screen.", so that will be a bit too artificial. So hopefully, with the digitalisation, maybe it will be more useful, but there will also be an issue with the care plan, the survivorship care plan, because it needs to be constantly updated, and when there's new treatment that's being introduced to the patient or when the patient goes back to see the oncologist, it has to be something that we can see upfront and seeing that "Oh! These are the changes that have been made already.". On paper form, it might be very difficult, especially every time a patient comes in, there's something that's updated, then they give you another new form and then you have to feedback, right, then it's five forms again, five forms to go and see, "Oh! This (is) the treatment that was done and it no longer worked, so the cancer might have some form of relapse, or the patient has too much side effects, so this is another new form for this patient again.", and then you need to read through, so that might be some of the issues that we see as well, that we foresee if we don't have something to help the doctor to manage.
M1	Thank you. So maybe as a conclusion, I will invite everyone maybe just to share, what do you think about this thing about shared care of cancer survivors, and cancer increasing its surveillance? Do you think that it's something exciting, it's something feasible, or do you think it's really not worthwhile going into it? Just a short brief summary, starting with A?
A	Okay, hi I'm A. I think it's a good plan to have, going forward, to have some form of survivorship care plan. Nevertheless, because we do know that for Singaporeans, we are living longer with more comorbidities, with cancer, with chronic diseases, so I do think that this is excellent moving forward, but it's just that there (are) definitely teething issues that will come up, because now with specific care plans for specific oncological conditions that, as what E has mentioned, there are sub selected group of patients that are really well and they have maybe an early-stage cancer, they have survived for a good five years before they come over and see us. I think family physicians are in a good position to co-manage together with the oncologists, but these are really for a well-defined sub-group of patients and it cannot be for patients who have multiple oncological issues together with multiple psychosocial issues that require tertiary care, and which requires multiple disease (referrals) back to the hospital as well. I think THAT is still very important to understand that there might be some teething issues, but I think going forward, in summary, this will be a good thing to have.

M1	Thank you. B?
B	I probably summarize as one of the cancer markers; we call it "PSA" – so, "P" stands for "Purpose", so why we are doing this? It's very hard for primary care physicians or the hospitalists to understand how the survivorship handover of shared care is to be, if let's say we are not sure about what is the purpose. Are we changing the care delivery model for better patient care, or are we just shifting the location of SOC (Specialist Outpatient Clinic) to the community, or the hospitalists are trying to push (out) the stable patients so that their KPI (Key Performance Indicator) for their new appointments are a bit better? So, all these are questionable issues. Second will be "S", "Stakeholders engagement", really finding out what are the important things to them here, and feed everybody with the correct meaning of why we do all these. I, as a primary care physician, I will strongly push for this for the benefit of my patients living in my neighbourhood, if I feel strong(ly) about them having this cancer survivorship engagement follow-up in my community, rather than keep on going back to a secondary hospital or tertiary hospital, which is far from their home. Last one would be, "A", "Artificial intelligence", which I think is something really upcoming and that we probably need to explore. With increasing complexities and different cancer survivors, and our patients (are) living longer, and the new medicine is coming in, day in day out, and (the) doubling of the medical knowledge, it is very hard for any one of us to be able to catch up with the updates and make a fair judgement and management for our patients. With artificial intelligence, potentially, it can help to complement and make sure all this information is being processed appropriately for the care of patients within the neighbourhood itself.
M1	Thank you. C?
C	I think that it adds value as an FP (family physician) when you actually be part of the management for patients who have cancer. I echo what A says. He said that as a family physician, you are there for the patients, and of course, you take ownership of your patients, and in fact by caring for patients who have cancer and et cetera, you also add variety to your job and also satisfaction to your job as a family physician. Apart from just taking care of patients, there is also improvement in your clinical skills and et cetera. But of course, the issues that (have) to be answered is how to actually execute this shared care. That is something that we have to bear in mind: funding, time constraint and also the coordination of how we share notes and et cetera. I think it's a lot of operations issues, rather than clinical issue(s). I think (with regards to) clinical issues, a lot of FPs, or rather family physicians, definitely will be more than willing, in that sense, to actually, if they were given the correct condition(s) and environment, I'm sure they will actually manage, because to us, "I can't say I don't want to manage, because that is not part of who we are.". I mean, if you are a family physician, all condition(s) are, of course, of your interest, so I would think that if I were to be given the opportunity, I would definitely want to manage this type of patients, but of course, with the proper support and environment that I would expect.

M1	Thank you. D?
D	I'm supportive of the model or the concept of the model of having shared care programme, but I think, at present, we will have to work on funding, So, as we've highlighted previously, it'll be great that the Pioneer Generation (Scheme), the Merdeka Generation (Scheme) and any other generation (schemes) and the CHAS (Community Health Assist Scheme) can consider cancers as chronic disease(s) and provide some form of funding support. The other thing is the training of our doctors in terms of upscaling their skills, and the postgraduate teaching in particular, as well as improve communication links with the oncologists, so that everybody buy(s) in to the model. And I definitely see value in instituting a shared care programme for breast cancer survivors, and in fact, or any cancer survivors.
M1	Thank you. E?
E	So, I see that there are benefits in this shared care model, so I suggest that we pilot it and we look at the process and we refine the process along the way. Now, as Ministry is trying to move from the healthcare to a value-based healthcare, we also need to look at the cost-effectiveness of this care and determine that if this kind of care allows the patient to be managed in the community setting that is not worse off in the tertiary setting, then definitely, it is something to be considered seriously.
M1	Thank you. F?
F	I think my main thoughts are, I think when B mentioned that what exactly are we trying to do with the shared care model and who are we trying to help, so we have to think about, in the community, who are our cancer survivors and what are their characteristics. So, like for example, if I'm a breast cancer survivor on Tamoxifen and I go to the polyclinic to just monitor and repeat my Tamoxifen instead of NCC (National Cancer Centre), then what are we trying to do? Are we trying to bring in the SOC (Specialist Outpatient Clinic) and adding a nearer clinic to their house, or how are we going to add benefit in terms of how the patients are managed? So, for example, in terms of the mental health clinic, it works because there are many patients with quite straightforward anxiety and mood disorders that, as primary care providers, we should know and manage. So, similarly, I think in the community, the cancer survivors that I see are the ones that are very well, are in remission for ten years, but there may be unaddressed issues like osteoporosis, or they missed their scope because they have defaulted their appointments because they feel well, so as family care physicians, at the basic, I think we should know what to pick up and what to follow up (on) and have that knowledge too. So, I think when we talk about the shared care model, we have to see where, which part of the care that we want to pick up. And personally, I feel that with our current system and with these patient characteristics, maybe it might be better to focus on educating ALL primary care physicians about late cancer survivorship, what to look out for as they grow older, because these are the patients that we are really going to see, and I think it's the largest number of patients that we can make (feel) a little better later on.

M1	Thank you. Any last words? Thank you all for your valuable and practical input today. We'll stop recording now.
	[Audio recording ends at 1:17:33min]
