# Supplementary material for: Implementing a community-based shared care breast cancer survivorship model in Singapore: a qualitative study among primary care practitioners
Source: BMC Prim Care. 2022 Apr 8;23:73. doi: 10.1186/s12875-022-01673-3 (PMC8991467; doi:10.1186/s12875-022-01673-3)
Supplement: Supplementary file 3 — Additional file 3. A compressed folder containing the raw data transcripts and demographics data collection form. [file 12875_2022_1673_MOESM3_ESM.zip › Supplementary Information File 3/IDI (09.14.2018).pdf]

Transcript for IDI 14th September 2018

Key:

Moderator / Interviewer:	M1
Respondent:	A
( ):	Paraphrases, additions to or rectification of grammar, vocabulary and/or truncated sentences.
[ ]:	Non-verbal, e.g. *[xx laughs] [pause]*
…:	Removal of false starts, repetitive or ungrammatical long phrases
CAPITAL LETTER:	When there is a louder emphasis or stressing on a particular word or phrase

M1	Okay. Thank you, doctor, for taking this invitation to our interview today. We'll like to discuss the six themes. Can we start with the first (theme): would you like to share on your background and survey on your current practice? Do you see cancer survivors?
A	Yes, we do, but currently, we don't really focus *[trails off].* Usually, most of our consults are not really focused specifically on cancer survivorship issues, so it could be, but rarely – rarely, I would say. So, I mean, from the stories we hear, from the other doctors talking about it, some of them could be very specific, like it could be lymphedema, but that forms a very small part of the consult task that we do with the patients. So, most of the time, we KNOW that they had cancer, they are on treatment, we usually just try to make sure that they are still on follow-up, and then we stop thinking about it *[laughs; M1 laughs]*. We DO *[trails off]*. I think what are the things that we usually do would be *[trails off]*. Okay, I mean, because we see the preventive and so-called, the "preventive health", (so) if you see that as part of our task, yes, we do that part, but we don't really link it to any particular cancer. Although sometimes we DO. So, for example, sometimes we tell them, as we're talking about exercise, or we're saying, giving them motivations to do exercise, then we'll bring in (points like), "Do you know that it does show that exercise reduces (the risk of contracting) some other cancers.". Of course, but not so much breast cancer but yah, the linking part, we hardly do too much of these links back to the topic of the cancer, because we are already doing it pretty much run-of-the-mill with their chronic disease. So, that part, we seldom do the linkage so strongly.
M1	Thank you. So, based on what you said about the "we don't really do the linkage", but you know, going forward with family medicine trying to provide holistic care, and with the government's perception of having "one patient, one family doctor", do you think that this care is ideal at the moment or whether there is something more that we can do for the patient(s)?
A	Ideally, yes. So, I do agree with the vision that it should each patient should have a fairly regular provider, but of course, operationally, there's a whole host of other issues. So, the more complex the patient('s) (condition) is, it could be possible that

	we can't use for this model for EVERY SINGLE SINGAPOREAN because it might be just challenging, but I suppose (for) a particular group of patients with a lot more conditions, I think that relationship should be stronger. So, whether or not they are *[trails off]*. So, it shouldn't be, say, some need GP (General Practitioner), some need primary care, (but) I would feel that even preventive (care) should STILL be (the) bread-and-butter of polyclinic's practice. But I don't see why not, because you are still treating the patient, right? So, you should actually be able to handle the whole spectrum, and it will work well for the patient as well, because many of the things we talk about, whether it's preventive, whether it's chronic disease related or any health issues, SHOULD USUALLY be discussed in the context of your understanding of the patient's psychosocial preferences and the context. So, being the regular provider, then that will make sense. So, it is just that, well, it's not just so much a cue to get us to link it back to their cancer *[laughs]*. So, we're REALLY just like, "Okay! You are following up with the oncologist. Okay!" *[laughs]*, so we will be very happy, and then we just move on to (something else), because the honest thing is that, there are too many tasks IN that short consult, right? So, it is NOT so easy. But of course, (for) the cancer one(s), I would say, the period that we pay the most attention to them is actually the period post-diagnosis, because (for) that part, we know that, "This one, we send there. Just got cancer.", and we make a conscious effort to check in with them to see how they are doing, if they are coping, but once it becomes stabilized *[laughs lightly]*, then we just *[trails off]*. Because, there could really be other priorities, so once they are stable and are doing well, then we don't really spend so much time on exploring the issues.
M1	Thank you. This is really in resonance with many of the feedback from the (other) focus group(s). There are some of them who shared with us that if they are the ones to pick up the breast cancer and to refer to the cancer institution, they really want to be updated in terms of the care, because ultimately, it's their patient, and they feel that there is a little gap in the communication. Do you think polyclinic should be involved at the BEGINNING or at the END of the treatment, when the patient is actually well and discharged from the institution?
A	Well, I suppose if you involve the physician earlier, then naturally, the understanding of the context will improve, and that may help bring in some of the issues at the consult; because it is not happening now, we don't really talk (about it); because it is a black hole, we don't know what is happening there, so we don't talk about it, because we don't really know what's happening there. But I suppose in reality, then it's again, the prioritisation when it comes to the consult. So, sometimes as primary care physicians, we struggle a lot and I think it's all across a lot of situations that, because it's the holistic person, there is so much information, so how are we ever going to get past this volume. And I don't see an easy solution to this, because there are really so many things talk about... But I suppose if it's something that's bothering the patient at that point in time, and we have information about the treatment and the decisions that are made based on whatever reasons, then we could help to at least address some of the patients'

	initial issues and not say, just brush it aside. So, that would certainly help in bridging the gap in terms of the involvement.
M1	Thank you. So, I guess there are two aspects: one is the assessment of symptoms, like for example, if a breast cancer patient comes in with a cough, then I guess it'll be good for the primary care physician to know how to evaluate the symptom. And I guess the other thing will be the side effects of the medication, because many of them, like the aromatase inhibitors, they do affect the lipids. It's whether that you see primary care physicians to play a role, or you expect the oncologist to manage the side effect(s).
A	No, so what we are saying is that we know that primary care has that usual chronic disease (in which) we have our strengths, so all we need is just that "yes" to bridge that gap, to say that "Okay, this is what she's on. And these are the potential problems, then we will know what to do naturally." – it's just sometimes, let's say we're not familiar with the newer drugs, even the names are difficult for us (and) we don't know what that means, but as long as you just tell us succinctly that "Okay, (for) this one, they will have a problem with lipids.", then you can leave the rest to us. It's just that initial cue that IT IS going to be a problem.
M	So, it'll be good for the oncologist to communicate regarding this?
A	Yah.
M	Because many of them think very highly of primary care physicians to manage lipids, so they think they should have no problem(s), and then, they will still manage the osteoporosis part, so you see (that) many oncologists… will still support them with biphosphates, but in terms of the lipids, they know that the primary care physicians are the specialists in it, so they actually leave it to the primary care physicians to manage.
A	Okay. So, I mean, we CAN define these things. (For) osteoporosis, we are also managing, so there is no *[trails off]*. The demarcation to us is quite clear when it comes to medications used for treatment specifically, so if you talk about the side effects, things like osteoporosis (and) lipids actually can be managed by primary care as well, but what we need is just the cue to say that "Okay, this is the drug I'm using. This is going to cause these problems.", and then, we will be able to then handle the monitoring and the treatment even, but the cue now… is missing, then we won't even think about it. We may talk about it, but (it's) from another perspective that's not so much related to the cancer drug treatment. And so, the other (thing) that you mentioned was about the acute symptoms. So, I think (for) the acute symptoms, as primary care physicians, we are quite confident. We usually write that they are cancer survivors, so when we see them with back pain and cough, headache (et cetera), we should have, we HAVE confidence that they don't just brush it aside (and that) they will do closer follow-up and make sure that this is not a (metastasis)-related issue.

M1	Thank you. So, as a senior physician in the management, I'd like your perspectives of the organisation. Do you have the resources to look after the cancer survivors?
A	*[laughs]* Okay, so, again, I refrain from saying "cancer survivors", but I would say that, yes, this group definitely will be a group of patients with more needs, so I think we (can) see it generically. So, we ARE struggling - there isn't much support on the ground because maybe (of) funding – I mean, of course it's funding - and the perspective of what primary care can do, and what primary care consultations look like. So, we may not have progressed that far from primary care if they give us five to ten minutes. What can we do in five to ten minutes? Even so-called the Family Physician Clinic is fifteen (minutes) and we are already struggling with fifteen (minutes), and more so when you have a whole host of problems. So, it is (about) how the funders and whoever out there is visualising our role. So, there is one (role), one that is with patient at that point in time (whereby) you need to engage… the biopsychosocial and that's like three components. And when I say "complex", (it) means (that) MANY of the domains are affected, then you have to *[trails off]*. You really have a lot to (handle). But of course, now it's a little better, because we have a little bit of a multidisciplinary-team approach, so we break up the tasks into, say, "Okay, so the nurse will help me with this and some of the tasks.", which is the next part I'm going to talk about. It's the task about planning the care, you know, asynchronous staff that (is) not dealing with the patient, but you are actually clarifying the care plan; you are actually making care plans, or you are trying to link up, care-coordinate, or you are trying to do care integration. So, actually, people don't realise this part takes up time, and it is NOT (done) during the consultation, because at the consultation, you want to focus on the patients. It is between the consults (that) you actually do the other things, like care coordination. So, you have mentioned about the oncologist sharing with us care plans, (but) WHEN that doesn't happen? If you show it to me IN consult, I'll be like, I can't. You know, I'll be struggling already. I'll be like, "Okay, what do I do now?". But in a way, if it happens along the way, so that when we next see the patient, the information kind of like weaves in bit by bit, so it's not like everything happens (in) five to ten minutes. So, I think some people don't understand that these things (are) difficult if you want us to do it. The five, ten minutes (are) so precious (so) you want to deliver your discussions with your patients already. You don't want to be (like), "But now, what's happening?" in the five, ten minutes now, you are LOOKING at NEHR (National Electronic Health Record), looking at SCM (Sunrise Clinical Manager) *[reference to computer programme for electronic medical records]*, looking at everywhere, and if you wire our brains, you will see our brains, like, flying all over the place and there's a flurry of action trying to link everything together, so that's really the tricky part. So, if we need to change the model, then we need to figure out HOW to CARVE out some time for care organisation and care coordination purposes. Even (when it comes to) sending out an email to somebody, it doesn't happen in the consult! It happens at the END of the consult and many doctors WILL NOT do it, because if they finish and they want to go home, they may not have time to sit down there and say, "Okay, so, for which patient? What should I do now between now and the

	next visit for the patient?", but I think if we can do that, I think the care will be more effective, because you are planning ahead, you are looking, you know, at your options, you are not just relying on that five, ten minutes, which is a pitfall for primary care consultations now, because it's just not enough time.
M1	Oh, do you have other suggestions how we may go forward?
A	I mean, the first one will be your patient – okay, for those with complex needs, right? Identifying the family physician will be important, so then at least you know if you want to communicate, you will know who it is. So, the same, likewise for the oncologist. Sometimes when we look at the patients, we actually don't know who the oncologist is (and) we just guess from the SCM (Sunrise Clinical Manager) *[reference to computer programme for electronic medical records]*, whoever is prescribing the medicine, but we don't know if they are residents or they are the primary provider, so figuring (out) that part will be important. So, the other thing *[trails off]*. Some of these things *[trails off]*. So, it really depends. The other thing is also whether preventive health and health promotion… could also be delivered in the community space. I mean, in the primary care space, it's more of making plans, but how do we support the patient in their *[trails off]*. So, it's also (about) empowering the patient, because if you are talking about (how) systems are being stretched, then how can we tap on the patients' motivation in the community as well? So, that's one part that we are quite interested to be working on as well.
M1	You mean, for the patients to access the resources themselves, like vaccinations, they may not really need to come back to the polyclinic to do?
A	No, that means, what I mean is the polyclinic is still the provider of these services, right? But sometimes, what I said initially is that, there is not enough time to go through what you have, what you don't have, what you need (et cetera) sometimes in the five to ten minutes, because you are already delivering the other parts of the care. So, what we are saying is perhaps that we can also empower the patients, so that means, each person kind of like have age-appropriate recommendations and guidelines and it's how to make this information packaged and compartmentalised in a way that is easy for the patient to understand, so that they can also play a small role in self-seeking to fill the gaps, because (for) the filling of gaps, there can be many options. They can go to *[trails off]*. I mean, it really depends - some of them can go to company doctors and some of them have other things. So, it is just to put some responsibility back to the patient. I think, that one, we are trying to do in primary care, so we are trying to see HOW to do that, because we realise that by the time we talk to the patient and at the end of (the) ten minutes, we only talked to the patients on diabetes, high blood pressure and all the stuff, (but) the preventive care really happens (as) "If I have time, I tell you, but if I don't have time, I don't.". But we try our very best to do that and it's improving, but if the patients can even take control of some of these, it's even better. Then, they can call the centre to come and do the vaccinations, rather than us having to plan it.

M1	That's right. I can see it's really challenging for the primary care physician(s) to manage so many aspect(s). So, can I invite you to look at the survivorship care plan? So, if a patient comes in with this document - this one is actually adapted from the American Society of Clinical Oncology, because for them, it's a requirement to have a care plan when (they) look after the cancer survivors - would it be very stressful when the patient comes in with so much information? Would it be too much information? When do they bring in this sheet (and) will it be useful as a means of communication?
A	Yah, it IS a lot.
M1	*[laughs]* So, there are two parts to it - first of all, it's a treatment summary, is it useful for you to know whether the patient has gone through which type of surgery, and radiotherapy, what type of chemotherapy, in terms of side effects?
A	I mean, yes, it would be (useful), as in, for us, we will need some orientation to these plans, so that we know that this is basically what the patient has done, and we will just run through it really quickly, and then, the treatment ongoing is where we need to look at, which (are) the side effects that (are) going to affect that bit.
M1	So, the other second part is that of the follow-up care plan, that means the oncologist will try to put in what is the frequency whereby they are expected to do their screening. And there's the other area where we really think that primary care physicians are really good at, in terms of managing psychosocial issues. We'll just like to invite your perspectives, what do you think of this area, in terms of fatigue, anxiety, depression, stress, weight management (et cetera)? Will that be good areas for primary care physicians to be involved (in)?
A	Yeah, I mean, that one, I would think so, because whether you have this plan or not, they will already present like that *[laughs]*, so we still have to deal with it, right? Okay, I'm just thinking, because this form is actually a lot of words and a lot of things, so like you said, I think there is really a lot of information, so how do we *[trails off]*. We might want to go through some reiterations of how this care plan looks like, because even what's on page one matters, because by the time you give us, you throw us a lot of things, then (when we) come to the last page of the problem and (read that) you have problem, I (am) already very stressed, so I may not look at it it's whether we want to see how to surface out the issues early, and then, it is like what is the most important (thing) that you want to catch their attention (for), and of course, it's just a matter of just re-ordering the sequence, or putting what the patient *[trails off]*. Is this what's shared by the patient? It's not, right?
M1	I mean, it's given to the patient, that means they are supposed to know what they have gone through, but many of the primary care physicians feedbacked that this is a good area, but it's also difficult to manage so many areas.

A	So, could it be just like, okay, you have "patient-concerns-related"? Actually, I'm not too sure … how do I fit this in, because to the patient(s), they also don't compartmentalise their problems, (like), "Oh, this one is related to my cancer.", or (for) anything, they will really just (say), "I'm feeling like THIS. You figure out what's happening.", so that's the part that maybe a little bit tricky, right? So, when –
M1	So, I guess, ongoing concerns may be difficult, but if they have something that is completed, like a past history, do you think that may be useful?
A	Past history, yah! But then you see, here in this thing, you will have, like "continuing care", "bone density test two-yearly", then you have this one, the "mammogram" and these things *[trails off]*. Oh! This is a continuation from previous page!
M1	*[laughs]* It's okay, because the form is so long!
A	That's right! So, that's what I meant! That's what I meant! You just need to give us the *[trails off]*. So, that's why it's to go through some iterations of… what is it that the patient needs, so that it is very clear to us what it is. But of course, this is in paper, and you have to transcribe it to SCM (Sunrise Clinical Manager) *[reference to computer programme for electronic medical records]* or (something) electronic.
M1	Would it be useful in the SCM (Sunrise Clinical Manager) *[reference to computer programme for electronic medical records]*? Like, I understand that you have a template for the various conditions, like for example if they have cancer, whether there is a column, and then, the mammogram is in it, so you know whether it's done or not?
A	That's right. So, this is the architecture of a very complex system. Obviously, it will really help us, because… that's where we struggle also, because different hospitals that we work with will have different care plans, different things, but when it comes to primary care, everything has to fall in one place, which is the great challenge. So, unless we have a sense, a feel of what are the common things, even so, designing a template, like some co-person thing in the SCM (Sunrise Clinical Manager) *[reference to computer programme for electronic medical records]*, detaining the BDS (bone mineral density scan), preventive care kind of content, and wherever this person is,… because some of them could be also seeing O&G (Obstetrics and Gynaecology), then they already have the Pap smear done, so if there is this template in SCM (Sunrise Clinical Manager) *[reference to computer programme for electronic medical records]*, and wherever possible, this information is plotted in, that will be wonderful. But to design such a system, you will need a really dedicated group JUST to integrate care. That will be very useful, you know, because for us, it's all blank now, (on the screen), I see "private (doctor)", then I see this, I see that, so it's a struggle just to bring the information together, because sometimes we don't have the time to do that, then we are stuck, then we will *[trails off]*. Some people don't do (it), because "I don't know you did or not.". If it is done within Singapore, then it's easy because it's already consolidated, but if it is done elsewhere, then we will somehow have to bring the information in. So, if let's say there is a way to bring

	this into SCM (Sunrise Clinical Manager) *[reference to computer programme for electronic medical records]*, that means even the cancer specialists can write (to say) they need these things, how does MY template pull in the information, that will also be (system) architecture, which is possible, but we will need a lot of refining and work on that, but it's definitely useful in that sense.
M1	Because, actually at the National (Cancer) Centre itself, actually the next phase will be building up an electronic survivorship care plan and we really want to know how to reiterate it. I mean, because now with the Clin-Doc *[reference to online system for documentation]*, we can actually see each other's notes, but there are so many.
A	BUT, exactly! Therefore, when the specialists are designing the system, then it may be helpful to imagine how it fails into place for one person, because it is easy when you guys are just doing THAT part, so whether (the) early engagement of primary care provider will be helpful, so that as you are designing (it), then it will also benefit the ideal state where the primary care can get to the information early. That means, what I mean is that, let's say you are already clicking now on the notes, right, the next time I open this body, this template, it, kind of like, updates or - *[M1 interjects with a laugh].* It can! It can be done! It can be done!
M1	Err, we have -
A	*[Crosstalks]* – yeah, it can be done, but it's just (that) you need that specific group to really define which fields to update and things like that.
M1	Is it currently being done at the polyclinic -
A	*[Crosstalks]* – so, for example, we have, but I don't know what works backend. For example, now we have a page with clinical summary, so we have patients', so (it's) very good. So, we have injections, influenza, pneumonia (et cetera). Last time, we used to have to look for the notes, find out what this person did and it's so painful, right? Now, you just click that tab, and then, in that same field, you already have (information on) the influenza is done when, and then, IMMEDIATELY you know when is the next thing due. So, actually *[trails off].* So, this information, I don't know where it comes from, but it is probably met by something else, so that it is very helpful. So, for primary care provider, we need these kinds of dashboards and it needs to be in that "one-person view"; it could be many sources, but it all comes back to that one person, because there are only these number of things you need to screen as well. You need to be, like, for example, Hep B (Hepatitis B), you need to remind (yourself) when to do the ultrasound, when you do the Hep B (Hepatitis B) screening, right? So, again, it's also in that tab now, then you can see when the last ultrasound was done, when was the last blood test taken, so that's very helpful.
M1	Sorry, is that in your intranet?

A	No, so it's in SCM (Sunrise Clinical Manager) *[reference to computer programme for electronic medical records]*. So, if we look at "clinical summary", this is built for poly(clinic).
M1	Built for polyclinic? So, can we view it? Can the National -
A	*[Crosstalks]* - I'm not sure. You can try? There's a dropdown. You can go and try it and see if it's there? It's a "clinical summary" for SHP (SingHealth Polyclinic). So, if we *[trails off]*. And I think there are preventive fields, so as to see how we to weave in some more, and so if it's stated that this was last done *[trails off]*. But actually what it doesn't have now is, it only has "what was last done" (but) it doesn't have "when is next (done)" *[both A and M1 laugh]*, assuming that, because the stuff there are all general knowledge, right, so you kind of like now when it's next due, but let's say if you have a specific care plan, then you have to figure out the field that says when it's next due, THEN you will know, because if not, you see already when it's done, you also don't know when it's next due, right? So, that might be helpful.
M1	I guess when the patient is still undergoing active hormonal treatment, probably it's still good maybe to see both sides and both sides can keep track. But then, we are thinking that when the patient has really completed treatment after ten years, there is very little the institution can do, so we take over the care, I guess that can just be a transfer of care, (that is) doable in the polyclinic when they are stable, their cancer.
A	Yah, it's doable when there's clear plans, that means like, what you need us to continue surveillance (for), and if you have specific care standards to be done, then we are quite - *[M1 interjects inaudibly.]* Mammogram, the usual.
M1	That's right. But the thing is that, why we pick breast cancer is because they are known to have long relapses, even patients being well for seventeen years, they can still come back with a cough, and often, they go to the polyclinic, so it's just that whether we are building up on confidence in training and education, it is still something that the polyclinic can do.
A	That means, to detect?
M1	To KNOW what the possible long-term side effects (are). I mean, not the very esoteric ones, but whether it's common for everyone. And then, in terms of psychosocial, we know that primary care is very good in psychosocial, which the oncologists are not very confident handling. So, in terms of mental health, do you think support in allied health services itself?
A	Okay, we don't have the psychologist per se on site, so (for) that part, we have the specialised clinics in the bigger polyclinics, like Health Wellness Clinics where they deal with the simpler ones - they don't deal with the suicidal ones and the depressed patients; that one will still be dealt with at the psychiatry level. So, having that setup of that clinic just give some respite to the physicians, that means when

	we addressed the issues, some of the more confident providers… actually deal with it ourselves, so we can just see them SPECIFICALLY just to deal with THAT SPECIFC (issue), that means, we just schedule, we pick it up and we can start treatment and discussion, and we get them to see us earlier, like within the next week, a SPECIFIC consult just to deal with that. Then, for the other physicians who are not too keen to do that, then they have the option of Health Wellness Clinic(s). Then again, the concept is just the same - the visit is just to deal with the anxiety issues, because you can't… but you want to dedicate more time to that.
M1	It's very good that they actually offer more services in the polyclinic. So, just regarding the fact (that) in the hospital, like in Orthopaedic, they are very sub-specialised, like, for example, you have a specialist for back pain, for knee pain. So, will the polyclinic envisage going into that particular area, for example, you say that if you have got mental health problems, then you go to a mental health clinic, then you have a diabetes clinic, and then you have a generalized clinic? *[laughs]*
A	Okay, no. So, I think that's not the mental model. The mental model is that we are all still holistic practice, but what I'm just saying is that, we have so many tasks to do, so what we are saying is that we might actually transfer certain in-depth tasks to a resource that allows (for) more in-depth tracking of that, that means, for example, I've got memory loss, right? We have a clinic that does cognition, so in that clinic, we do all the scales, all the things which none of us will do, but the output of it is that "Okay, I've screened and there's nothing.", so that means it's just a shoot-off to say, to go in-depth into certain complaints, but the output should go back to the person and the next provider who sees for the (condition) and that particular provider will just get a sense of "Okay, so how are things?". We are using the resources as a way to go in-depth to certain parts, whether it's the family physician clinic or whether it's just the current memory (clinic) and mental health (clinic). But the mental health one *[trails off]*. So, it's just to buy time, to sit down and work in-depth towards the same problem, but when we next see the person, (it's not like) we don't really care what's happening there – it's not that – so we will still see what's happened and do the continuity. But because in THAT visit, you might have to deal with the other things, the diabetes, the other things, right? So, you know that you cannot deal with mood as much, and this person has ANOTHER clinic to deal with it in-depth. So, that is like the balancing model that we have. It's not *[trails off]*. We don't foresee ourselves branching off like that, because in primary care, it's almost impossible; if not, your patient will be seeing psycho-clinics, so that's probably NOT where we want to go. It's just that, at any point in time, if we find that there is a particular care need that needs a little bit more attention, then we need to carve it out to say, "Okay, how do I address that part?". And if we have specialised clinics that does all these scoring and everything, then we will make use of that resource to help us out with that, because the nurses are trained to do certain cognition tests and scales and things like that.
M1	So, I must say that this is a very good vision of the polyclinic to bring back the patient as the whole, and then, to coordinate care, because in our experience in the

	hospital, patients are actually lost, because that there are so many sub-specialties and they don't communicate even in the same discipline, and they are saying that, "This is not my problem. You go and see the other doctor for your other problems." *[laughs]*.
A	So, you are right, but some of them are even *[trails off]*. This is the problem, right? We also have a problem with THAT problem, because they refuse to *[trails off]*. Okay, so (firstly), I think that may change if we are serious about (letting) every patient (have) some primary care provider, THEN maybe there is some hope for these people floating around in SOCs (Specialist Outpatient Clinics), right? But the fact is that the people in SOCs (Specialist Outpatient Clinics) may not be helping us, because sometimes if you noticed, they also give all the diabetic medicine -
M1	*[Crosstalks]* – everything that that patient asked in the outpatient -
A	*[Resumes]* - therefore, it has to be a shared vision; it cannot be a one-sided vision, because then, we are SO UPSET with the specialists when they are prescribing all the medicine, and they refuse to come back and we can't do anything about it. So, it's probably about the whole vision that we have, (like), okay, this one, you can give them a bit (of medicine) to tide over, but they should still have a regular provider for their primary healthcare needs.
M1	So, if I can just delve a little bit into this area in terms of ownership, so, if the institution helps the patient by providing a little bit of medication, I mean, what is the… maximum duration (and) do you want them to titrate the medication, or it should still go back to you?
A	I think this is all contextualized, I mean, if there is an urgency, why is *[trails off and laughs]*. In fact, we do a lot for the specialists, some patients call and say, "Something has to be done.", and then we have to go inside and look, but we try to contact the providers and see, to confirm if that's the case. So, I think it's contextualised, but the whole idea is just not to *[trails off]*. I don't know. It's like, the intention is clear, that means, they are not really seeing yourself as an ongoing provider for, say, chronic disease management, then don't give so much (medicine), maybe like up to a month or two weeks - usually two weeks to a month - just enough to make (it to) the next appointment to see (us). I mean, we are highly accessible. We will see ANYBODY that comes in throughout the hours, so there is actually no huge accessibility issue, REALLY, in polyclinics.
M1	That is the really good part, the accessibility and the relationship, because they see polyclinic much more often than they see the specialist provider.
A	Correct, so I don't see any issue with that.
M1	Okay, that's very good. Is there any other input that you would like to give?
A	I mean, if NCC (National Cancer Centre) is thinking about *[trails off]*. I mean, we are also thinking about some of this "whole person care" kind of model. In fact, part of

	us is also thinking, like, if you have, say, an index case of a cancer in a certain family, actually some of the better primary care physicians will actually make it a point remind themselves also advise on, say, screening for their family members, so it could be useful also to think of it that way, (like,) how do we remind the primary care physicians that with the index case, they can be even more proactive in the management of their family. So, like colorectal (cancer), I think the surgeons just cut and they forget about everything else, right?
M1	They know family physicians are very good. After that, they can - *[trails off and laughs]*.
A	But no, even family physicians have to remember that, "Eh!", sometimes when they see the person, they will remember to ask, "Oh! How old is your kid?", like, you know, are they in the age group that you start screening, and then you DELIVER that advice. It's not like something so haphazard, like you remember, you do, you don't remember, you don't do. It can be tighter, because these things are already known, right? You have an index case, so how does it impact the individual and family. If we get that sorted out, the whole thing is (about) how do we present that as cues to say that this is something that you need to do as well, (that) you CAN do, you can OFFER to do as well.
M1	Actually, we did discuss cancer genetics with one of the groups, but they feel that it's a bit (too) specialised. So, this is still an early area. I think for many Singaporeans, they are not ready to accept it; they rather wait for things to happen; they say that, "I don't want to know whether I'm BRCA1 *[reference to a gene associated with higher risks of breast cancer]*, and I'll just do my screening.". Just to share a story that I shared with the group yesterday – it's like, I saw this breast cancer patient at forty years old. She was being followed up you know, every year meticulously by the cancer centre, every year for twenty years, and at sixty years old, she developed ovarian cancer. It was then the oncologist said, "Okay, let's do a gene screening.", and she turned out to be BRCA1 *[reference to a gene associated with higher risks of breast cancer]*. I mean, we actually missed (it) and she had stage IV cancer, so it's just that if someone had picked up, it may not *[trails off]*. I mean, I would say that it's not fair for the primary physician, but it's just that ANY doctor, and even the oncologists they themselves can miss such cases, so it's just with more awareness, like, can we upscale our population's knowledge as well, for them to do preventive care, rather than wait for the cancer to develop and there is not very much that we can do; because there (are) quite a lot of patients out there, five to ten percent, who actually have hereditary cancers.
A	That's right, and it may impact their family members. I mean, it may be something good for the family, in that sense. But it can be stratified, I mean some of it is (due to) genetics, right? But some of them is also be (due to), for example, other things that come along, like, smoking, or maybe you have *[trails off]*. Yah, it's just (that) we don't want to miss the *[trails off]*. But I guess some of the very *[trails off]*. What you call that? We probably need to think more in-depth, but I think from our more

	generalist perspective, it's just to say that, "Well, if I have cancers in the family, how do we proactively provide information actively to say that, to link it up and say that "This is what's relevant for you.", right? So, then your family member's cord blood may not be good for you, so we'll try something else already. So, for breast (cancer), (and) similarly for some of the more common cancers, we can have that awareness, then it may help you to give that preventive advice.
M1	Do you have any other perspective(s)? I think you have already shared many valuable points. It's just that, going forward, do you see any picture, because we know that this care is not going to change over the next few years, and it may be a long-term plan?
A	I think now, the immediate thing is like, sometimes the access to the hospital *[trails off]*. Let's say, if the vision is that hospitals want primary care to play a bigger role, then the support that the specialists can render will be something that is very important, (and) how do we get access, be it (for) queries, clarifying things (et cetera), these channels should also be well-established as you roll out the plan for empowering primary care.
M1	Actually, in terms of the institution level, they did think about having open lines, phone calls to the oncologists, but to the oncologists, it may also be a duty of care, it could be that "It's not my patient.", or "I can't remember when was the last time (I saw the patient)", so they may not able to pick up the phone and answer straightaway, which some of the groups feel that they want the information quite soon when the patient is there. So – *[trails off]*?
A	Personally, I mean, many of times, it's not immediate "immediate", I would say. I think within the next one, two days, it should be fine, but it's what kind of channels that we envision, and that is comfortable. So, now what we try to do is that we email, but sometimes (for) the emails, some specialists, maybe four out of five will reply, (but) one out of five wouldn't, then we are stuck, and then we don't know what to do next. *[laughs lightly]* Then, so these are channels should be *[trails off]*. So, that means, so then we feel that it's a SHARED partnership, and we feel that it is not just decanting or -
M1	*[Crosstalks]* - yah, because so many departments actually want to decant to the polyclinic –
A	[Crosstalk] – yah, so you know, for example, there's this case of lymphoedema, so we know that, physio(therapy) or I'm (actually) not too sure what is the up-to-date treatment, but we are trying to get her to go for it, but then our physio(therapy) in the polyclinic doesn't do this kind of specialised thing, and then, we're trying to get her back into the hospital, so there were some coordination issues, so we might need some proper channel. It may be that some of these queries could be directed. This one (is) probably not relevant for oncologists, but (it means) that the channels should also involve some sort of navigators or something, so that people who are very familiar with the resources, can support the patients. So, sometimes (for) some

	of the medical ones, they can go to the doctor but for some of the not-so-medical ones, we can also direct to whoever you tell us to, then we will do, so that will be helpful, because (currently) "We don't know what to do, refer to where, so then how ah?", because (it's) very funny, right, "Lymphedema. So, go back to where?", so we don't know, so that was a bit challenging.
M1	So, I guess if we have a shared care, we must include a coordinator?
A	Yes.
M1	So, whether it is to the doctor or it's a referral for certain tests or to support services?
A	Yah, I guess you could say that. And so, a contact point would be helpful. And then, basically, if we are in trouble, HOW can we navigate, so that their constant engagement would be useful, because then we can target, "Okay, so what are the challenges?", and then, we continue to refine the shared care model, then that will be good.
M1	Okay, thank you very much. Thanks for your time. I'll just stop recording.
	[Audio recording ends at 43:49min]
