# Supplementary material for: Implementing a community-based shared care breast cancer survivorship model in Singapore: a qualitative study among primary care practitioners
Source: BMC Prim Care. 2022 Apr 8;23:73. doi: 10.1186/s12875-022-01673-3 (PMC8991467; doi:10.1186/s12875-022-01673-3)
Supplement: Supplementary file 3 — Additional file 3. A compressed folder containing the raw data transcripts and demographics data collection form. [file 12875_2022_1673_MOESM3_ESM.zip › Supplementary Information File 3/FGD (09.13.2018).pdf]

Transcript for FGD 13th September 2018

Key:

Moderator / Interviewer:	M1
Respondent:	Participant A (A)<br>Participant B (B)<br>Participant C (C)<br>Participant D (D)<br>Participant E (E)
( ):	Paraphrases, additions to or rectification of grammar, vocabulary and/or truncated sentences.
[ ]:	Non-verbal, e.g. *[xx laughs] [pause]*
…:	Removal of false starts, repetitive or ungrammatical long phrases
CAPITAL LETTER:	When there is a louder emphasis or stressing on a particular word or phrase

M1	*[takes time to settle down; 0:00 – 0:12 min]* Good afternoon. Thank you for coming to our focus group discussion today. Our topic today is on community management of low-risk breast cancer survivors by primary care physicians. So, we have six themes. We'll start with the first. We'll go round. Maybe you'll like to introduce yourself as, like, A, and share with us some of your experience with cancer survivors in your practice. A?
A	A from the polyclinic. For the cancer survivors that I see in the polyclinic, generally it's mostly from their previous history and records, and most of the time, not much is actually brought up with regards to their cancer condition, as some of them may still be on follow-up with their oncologists or with their surgeons, but we do come across, once in a while, some complications related to their cancer treatment, like, lymphedema definitely for breast (cancer) patients, but by and large, not much troubleshooting (is) done. Generally, we'll still more treating their chronic conditions, like their diabetes and high-blood pressure.
M1	Okay. B?
B	Hi, I'm B from polyclinic as well. So, my interactions with patients are mainly like what A mentioned. So, they will let us know that they have a past history of cancer, but we do not actively do any follow-up, unless there is any memo regarding their care from a specialist. But otherwise, we generally just treat their chronic conditions, but we do keep a lookout regarding any cancer occurrences as well.
M1	So, C, maybe you'll like to share with us also your experience, how many patients (there are)? Is it a lot or not so many?
C	Okay, I'm C. I'm also working in the polyclinic. I think, in my years working here, I do encounter quite a lot of patients with cancer diagnosis, but they are usually part

	of their background history, and they are usually here for, as mentioned, their chronic diseases. Then, we may try and ask whether they are still on follow-up, and if they establish that they are on follow-up with someone, then we don't ask so much about cancer-related issues. We do keep a lookout if they develop some acute complaints, for example, chronic cough or back pain, we will be a bit more proactive in doing further investigations to rule out any cancer recurrence.
M1	D?
D	Hi, I'm D from a polyclinic. So, as my colleagues have shared, most of the patients whom we see, who DO have a cancer background, either they will volunteer that information to us or we see it from our electronic records, we actually trace back, or when we are prescribing certain medications, we happen to see that they are on cancer treatment, for example, like hormonal therapy for breast cancer, we will then question them about whether they are still on follow up with somebody, whether they are still on the medication or (whether there's) potentially any side effects from the medication. In certain cases, for example, if I happen to be running my family physician clinic, and I do have a little bit more time and I do know that the patient has cancer, I will actually then explore a little bit more on some of the issues that they may have faced during their cancer, whether they have had a follow-up, for example, for the bone mineral density scan as a result of … complications from cancer treatment. Because we have this new clinic that looks into advanced care needs as well, there've been some patients who may have been referred to us actually from NCC (National Cancer Centre) for discussion of their Advanced Care Plans, so that allows for interactions at a deeper level about their cancer treatment.
M1	E?
E	Hi, I'm E from polyclinic as well. I think I don't have much to add after all that my colleagues have shared. (With regards to) the number of patients per month, probably, I mean, I've had QUITE a number - I cannot remember the exact number – but in terms of how we can contribute to the care, I suppose sometimes they may have questions regarding their treatment, particularly those who have just recently been referred to us and are new, and they may ask us about some symptoms that they have experienced, whether it may be related to medication. So, I think what would be useful for us to manage these patients could be better awareness, in terms of knowledge, I mean, we can always look it up. We are aware of all the issues that we may encounter, then we can look it up. And I suppose some collaboration with NCC (National Cancer Centre) may be useful as well. I think a good place to start may be just, maybe, a teaching session with CME (Continuing Medical Education) to raise the awareness of what are the possible things that can go wrong in this group of patients, these low-risk survivors.
M1	Okay, so the reason why we pick breast cancer is because they are very long survivors, and some of them are actually cured, and because of the long duration

	of the hormonal therapy, which has many side effects, so maybe, as senior doctors here, you have a lot of experience actually encountering these patients. Would you be able to share instances or stories in which the patient is actually well-managed IN the polyclinic? Or can you actually share with us stories whereby you feel that the care is just NOT IDEAL, (whereby) you only know one part, the treatment part of their condition, and then, the other part is actually another area, and whether it (requires), like, special service, special areas or extra time, you know, that would be good to provide holistic care for these patients? So, we invite any doctor to share? Maybe you can identify yourself before you speak?
E	I'm E. I think, MAINLY, I still go back to the theme about the awareness, (on) what are the possible things that can happen in these patients. Just like (for) low bone mineral density, the fact (is) that that you need to screen these patients. A lot of these patients were not actively informed about *[trails off]*. If they feel that it's a cancer-related thing, they may not raise the issue when they come and see us, because most of the time, they see us for their chronic conditions and they may think that, "Oh, I can wait till my NCC (National Cancer Centre) and tell my cancer doctor rather than tell the family physician.". And so, in the area of preventive care, at the end of the day when we recommend screening and preventive measures, it's all about knowing this patients' risk factors, so I think some awareness of what are the specific risk factors that are unique to cancer survivors, that would be useful to know. So, when you (ask) which areas are lacking, I suppose if the patients do have the risk factors and it's not known to them, there is one gap already.
M1	Thank you, E. So, can I ask that, are you quite happy with the care of the cancer patients when you encounter them?
E	Honestly, in the consults, I think there is very little focus. The focus is *[trails off]*. A lot of the focus tends to be on their chronic medical condition (and) on their vaccinations. And I would say, (for things) specific to the cancer, unique to these cancer survivors, then (nothing) doesn't really come to mind. So, that itself might not be such a good thing *[laughs]* because of the awareness. Their awareness is quite low.
M1	So, the other group did share about the time factor and the load, … it's really difficult to manage so many things. But it's just that, as we are promoting family medicine to look after patients (with) patient-centric, holistic care, how do we see these cancer survivors, because it is going to be that cancer is the most important leading cause of death and one in three has cancer, we may counter it maybe in our friends or own family members, or even in some of them ourselves, so it's just that, how do we, as family physicians, promote care for these patients? In the polyclinic, they have the structure, the teaching, you know, the system. How do we enhance care for them in the polyclinic setting? D?
D	Hi, I'm D. I think we do need to have some handles on what is appropriate to screen for these cancer survivors. For example, it would be good to give a certain time

	frames, for example, in the first one or two years, what to look out for, then in the next two to three years, what to look out for, so at least we are more aware that if this patient was recently diagnosed with breast cancer and has undergone treatment, what are some of the side effects or complications of treatment that they have actually experienced, or what are some other areas of care that we can look out in a post-menopausal woman, for example, or in a young female with breast cancer. For example, also, whether the psycho-emotional parts have been addressed, especially for a woman's sexuality when she loses her breast, whether there is even the need to extend breast screening to her family members and if there is a genetic component that play(s) a role in bringing the person to intervene. And then, in the long-term care, I think it's mostly the ones, who have had cancer, for example, for ten, twenty years ago and are actually very well that … it just becomes a medical history on their chart and is actually not very much related to (the cancer) and we don't really look back at the history. And some of them are actually already discharged after bilateral mastectomy, so we don't really ask them any more whether they have any issues with their history of cancer. But I think it'll be good, from the oncologist's point of view, whether they could share with us about the different immediate care, intermediate and long-term care that we can provide for the patients and being inclusive for them.
M1	Thank you, D. So, you are saying that it's good for them to communicate from the special(ty) to the primary are setting. Do you think that they should involve the primary care physician at the BEGINNING or at the END, say, five years after when the treatment is finished, and then they give you a discharge letter, would you want to be involved at this stage?
D	I think the preference is that we are involved at an earlier stage, especially if they already know that these patients are already seeing us for the diabetes, hypertension, or chronic disease, then all the more we would want to participate in this patient's care and understand that this patient is going through this series of treatment. We can also play the role of looking out for whether this patient is experiencing any potential mood-related disorders, or anxiety-related problems as a result of the treatment, or the disease that is labelled on them.
M1	Can I ask A, because we have one feedback from previous group, from a male participant (and) he is saying that in terms of breast cancer care, it is something very personal and … if you really want to… manage the cancer aspect, you need to do a physical examination. Do you think, as a male physician, (that) these are the patients you are comfortable with, and which part and in which capacity (do you think) you can contribute to the care of the cancer survivors?
A	A. So, to the question, I feel that if there is already a relationship built between the patient and the doctor, there shouldn't be any *[trails off]*. I think there will be less of a barrier in terms of the doctor examining the female patient, and by and large, we also have a chaperone every time when we do examination on sensitive areas.

	So, in that respect, (there is) probably not (any issue), if there is already a relationship built -
M1	*[Crosstalks]* - that means, it is okay? You are comfortable?
A	Yah, it is okay. But of course, if you were to only see the patient episodically and it's a different doctor each time, then I suspect the patient herself would also feel quite uncomfortable, even if the doctor is agreeable to do the examination. So, that's what I think.
M1	So, the context whereby this was brought up is that because they were saying previously there were a lot of antenatal shared care or even postnatal care, but the trend seems to be (that) the patient is going straight to the specialist. So, can we persuade this group of breast cancer survivors to actually come out and do shared care in the community (and) whether there are other barriers which are something that we can't really control? Yes, C?
C	I'm C. I think it depends on what is the patient's confidence level with cancer survivor care by the primary physicians. So, they may be so used to being cared for by the oncologist, which probably would be the same oncologist who has been seeing them for so many years, that they feel that when they are transferred to the primary care physician, especially a new one (whom they have) no prior relationship (with), it can be harder to establish that. And they may have this concept that the oncologist, being a specialist, if there (are) any issues encountered at the primary (care) physician side, they STILL have to go back to see the oncologist, so why waste the time going to and fro to different institutions or different places. If I just STAY with my oncologist, and (if there were) any issues, he or she will settle it immediately because they might have more resources. So, that might be one of the (barriers). And for primary care, I guess most of us are… pretty willing or not reluctant to be involved in this, (but it's) just that I think we do need more training (and) more belt confidence as well, because the less we see this kind of cases, actually the less confident we are. And for me, there's a certain workflow, flowchart or something black and white that guide us as we see these patients, to remind us what are the things to look for, and whether it is actually mid or late (stage) survival, then that will be very helpful. And we might want to consider a dedicated clinic whereby there's more time given to these cancer survivor patients when they come in for not just cancer, but maybe their cancer-related issues PLUS chronic conditions. We need more time, because we don't usually have a lot of time to sit there, and if you want to talk about psycho-emotional counselling, we REALLY need a lot of time.
M1	Thank you. D, maybe I can invite you to share on the barriers, because we think that the relationship is really the important thing that is between the primary care physicians and patients, because they come often, as opposed to when they go to the specialist centre once a year? So, do you think that this relationship will be an important solution to providing this shared care, especially if you are the primary

	care physician who actually picked up the breast lump and you sent the patient away? Would you be able to continue the care of this patient?
D	Yah, of course, and I think I speak for all of us that we want to follow up on these patients, because we know that most of our patients are generally not in the family physician clinic. So, we do see quite a big pool of such patients in general clinic where the (nature of the) follow-up is different, so ideally, we would also like to encourage, say, (when) the specialists, oncologists have discharged the patients, maybe we can register them in for Family Physician Clinic. The other thing about the barriers is really the mindset of the patients. Like what C mentioned, they would be happier to just stay on long-term follow-up with the specialist and be in touch with the specialist, so maybe for the oncology side, they could help by reassuring the patient that all is well and they can discharge to primary care, and that there will be no problems to refer back to the specialist oncologist or whoever is following up with them. So, probably with that kind of assurance, maybe the patients will be more willing to be followed up at our side with as well. And for our side, maybe we could have a list of who we can contact, by email or by a text or other ways with the specialists if we do need to clarify anything with them. I think that would help as well.
M1	Oh, C, sorry D, just now you mentioned (about) a special programme, Advanced Care Planning programme. Do you think that breast cancer patients can be seen in this clinic? Would it be appropriate?
D	Yes, I would think that it's appropriate, unless of course *[laughs]* the cancer centre offers it to them during their time of diagnosis, because what we understand is (that) USUALLY Advanced Care Planning is best discussed at a time when they are comfortable, when they are not newly-dealt with a new diagnosis that is potentially life-threatening or life-changing, and the opportunity is that they have had a critical experience in their life, they can then plan for some of the things that they value, lay some foundation for their family will be able to support them WHEN one day they cannot voice out for themselves anymore. So, I think it's indeed a good consideration that because we ALSO do not want to roll out Advanced Care Planning just for cancer survivors, but (for) anyone who wants to consider about their health in the future.
M1	Thank you. So, maybe we can go on to this care plan which we have here. So, this is a care plan which the American Society of Clinical Oncology use, so they mandate – it was actually a law passed down – that all those who look after cancer survivors must give the cancer survivor a care plan. So, we'll say that probably we'll give the cancer survivors a care plan at the END of active treatment maybe after six months or one year. So, there are two main components in this care plan: first, is the treatment summary, what they have gone through, whether it is the surgery, chemo(therapy) or radiotherapy, and the second part is actually a follow-up care plan in which the recommended screening modalities, as well as the frequency are listed out here. So, we'll like to invite you to give feedback on this care plan, (on)

	whether there is too much information, too technical, or are there any other areas in which there's information that is missing? *[pause; 19:45 – 19:54min]* So, maybe I can bring you all through: in terms of treatment part itself, do you think all this information helps you, whether it is estrogen-positive, progesterone-positive, whether the patient has gone through surgery, (removal of) lymph nodes or (there has been) systemic therapy? C, you agree?
C	I agree that it is useful.
M1	Is it too much information, because some of the groups are saying it's too much, so just tell us whether it's surgery or whether (the patient is) taking medication now?
E	Maybe – *[M1 interjects, "E?"]* okay I'm E - the information is definitely useful, but I think we need to know what to do with the information. So, if there's surgery (done) to this particular area, or there's this lymph-node that is removed, be it sentinel versus axillary, what does it mean, what do we need to look out for and how do we manage (et cetera), so I think the information would be useful.
M1	So, there is a knowledge gap?
E	Yes, there is a knowledge gap, so… if you don't put it in this form and it's delivered to us in a separate set of notes, it's hard to look after the patient, you see. So, I think that's important, otherwise -
M1	*[Crosstalks]* – that means, a link to the further information?
E	Yah, that's right, a link or something. So, I mean, so some of the treatment is listed here and I suppose it is to look for side effects. And the other thing that would be useful would be things like medications, drug interactions, because these patients are often on many medications, so, common things like antibiotics to prescribe, does it affect the treatment or if it is a younger patient and they get pregnant, then what? Can it be continued or WHAT? So, I mean, for these things. And I think the other thing that is important is (that) if they are to be managed at the primary care level, probably it will be good if these patients are managed in a specific clinic. Currently, what happens is that they may come to our clinic for follow-up for their chronic conditions, for minor ailments, but if it is not a dedicated clinic for following up for patients with breast cancer – I'm not talking about those who have had it diagnosed for more than ten years and earlier, but maybe those who are still within the five years of follow-up where there is still a potential of recurrence - then if you have to examine them and maybe do physical surveillance for recurrence, then what are the things that we need to be looking out for? Because often times, the cancer may take a back seat when they come with a chronic condition, for example, their diabetes is not well-controlled or they have some acute problem, then that tend(s) to be the focus. I mean, they run out of clinic consultation time, (so) the cancer will take a backseat, and we'll be quite sad if no one is following up and then we miss (it), or they don't tell us what are their symptoms because there is not time, (and) we may miss a lymph node or something. So, if they have to see us, I think

	probably (it has) to be a dedicated clinic, and then, maybe (we) need to extend the consultation time – it can't be seen as a "by-the-way", as part of a usual consult. I don't feel safe in that manner.
M1	Thank you, E. So, I guess the role that we are trying to advocate is that the primary care physician is not responsible for cancer recurrence. I mean, they are mostly responsible for health promotion, but perhaps it will be good if they can pick up the red flag, because a lot of *[trails off]*. We interviewed some previous groups, even the GPs (General Practitioners) at a previous study, (and) they say that, "What happens if I miss the cancer?", and we are telling them in the first place that it's just to divide the roles and they are not responsible, don't have to worry, you know. So long as they pick it up, if the patient tells you about the lymph node; because we do have stories whereby the patient tells us that she has gone to GPs (General Practitioners) with skin nodules over the surgical scar, and it was seen multiple times and given creams, so, I mean, that is what we are thinking (about), that it is just the picking up. I mean, if there's recurrence, then it's the oncologists' role, and it is not the primary care physicians'. So, we are just thinking that in terms of this list, primary care physicians are very good at the psychosocial picking-up of the anxiety, depression, fatigue and sexual functioning, so can we have some feedback (on) what is the role of the primary care physician? Yes?
A	A here. I think most of us would even fear to venture into this area *[laughs and a few others laugh along]* because it's like Pandora's box *[others laugh again]* and you have a crying patient in front of you, that would last for at least twenty minutes, so probably (they are best seen) in one of the other FPCs (Fam Physician Clinic). I think you probably heard this many, many times, so it is the same here and it is the same with the senior doctors. No matter how efficient we are, psychosocial is always pretty tough to handle. So, as mentioned by the earlier participants,… we really need a dedicated clinic, more time, and of course, that patient-doctor relationship, the same doctor each time and then, overtime, you may get bits of information with repeated visits, and then you have a clearer (clinical) picture of the patient, then it will be much simpler to tackle such things, I find. And especially with sexual dysfunction, I think it is a bit of a difficult topic to bring up, especially if they are seeing the doctor for the very first time.
M1	Thank you, A. I must share with you it's the same feeling from an oncologist's point of view - they just don't want to talk about sexual functioning *[A laughs],* because they don't want to open up the Pandora's box. So, I mean, if the patient really has this problem, who do they talk to?
A	They talk to TCM? (Traditional Chinese Medicine). *[laughs and everyone laughs too]*.
M1	B, do you have any suggestions who should the cancer survivors go to or talk to when they have such issues?

B	I guess, also, mainly, it's who they are comfortable with, so hopefully if they are following up with us in our dedicated clinic, we kind of have a relationship with them, then they might be able to open up. Like what we mentioned, maybe the fifteen minutes talk in the clinic is also too short, so we might need to look into (having) a bit more time for this group of patients.
M1	So, we also have the experience whereby we have cancer survivors with maybe nasopharyngeal cancer, who come back smoking away, so it's just that the oncologists also have problem(s) talking about the prevention aspect, so may we invite *[trails off]*. What do you all think? In this group of patients, do you think that primary care can play an important role?
C	I agree. I'm C. If, for some reason, we managed to pick up that this patient is still smoking, maybe during one of our consults, I think (in) our role, we have to advocate smoking cessation, whether it's through counselling, non-pharm(acological) or pharm(acological) management, because, I mean, we have to educate the patient, and also to explore the reasons why they never stopped smoking or they picked up smoking. There might be other psychological, socio-economic factors, stress (et cetera). So, in that way, I think as a primary care physician, we can actually help to manage in that manner.
M1	Thank you, C. So, so that's an important area. How about lifestyle modification, diet, exercise maybe?
C	So, I think that as primary care physicians, if we happen to be managing them for their chronic disease, we probably do have more touch points than the oncologists with this patient, and it's usually really on the basis of relationship that we can advocate for the patient. If there is no relationship, it's very difficult to tweak through certain perceived concepts or barriers as to why they have chosen to adopt certain lifestyle(s) or to consider re-smoking again. And I think this is all part of, well, they could be stressed, could be emotionally down or they could find that, "Since I've had cancer, anyway, I'm not sure how much longer I can live anymore beyond this, so why not just continue with something that I like? It's my habit.". So, I think that as a family physician, we do have that touch point to be able to educate and give gentle council, but we are also not the ONLY person(s) who can do that. So, if we could engage the family, that would be one good thing. IF the oncologist can also step in to reinforce, I think more mouths speak better than one *[laughs lightly]*, and that will help the patient perhaps to reconsider their own behaviours and decide to change for something better.
M1	So, the other area is also in terms of the family, because we know that in a primary care setting, most of them come with families, you know, whether the knowledge of the cancer or especially there's any suggestion of genetic cancers, whether that would help the primary care physician to be able to influence behaviour? So, for example, I share with you a case of a youngest cancer patient diagnosed in (her) forties, and then just goes on with annual surveillance and was well, and then, when

	she was sixty years old, she just came down with ovarian cancer. So, the oncologist, after twenty years, then he said, “Okay, let’s do a genetic screening.”, and the patient actually has a BRCA1 *[gene associated with higher risks of contracting breast cancer and secondary cancers]*. So, we are just saying that, in terms of such patients, if the knowledge is with the primary care physician, would they be in a good position ALSO to encourage family members to come, to be able to either advice on the lifestyle modification or even for early screening in terms of the risk factors?
C	I’m C. In the polyclinics, it’s a little bit different, because we don’t always get to see the whole family – probably seeing GP (General Practitioners) would be good, because from the young to the old, they have the relationship. Here, we RARELY actually manage that. I mean the most is probably husband-wife couple, so it has to be THROUGH the patient, for example, you tell them, “Yah, you have colon cancer. MAYBE you should ask your siblings to for screening.”, that kind of thing, but not directly (tell) the rest (of the family), UNLESS it happens, or the family member is together with the patient when they come to see us, or if we happen to be managing the same family member. So, there’s some kind of limitation in that aspect. And then *[trails off]*. But it’’ not impossible. As for genetic counselling, that part, I think we are a bit lacking, so I don't think we should be the one to – *[M1 interjects, “Counselling is a very specialist area?”]*. Yah, correct. But just looking, screening (et cetera), then it depends. Sometimes it’s if you are NOT seeing the rest of family members, family members are not with the patient, then it is really THROUGH the patient (that) we encourage their family to come and do screening. And if the patient doesn’t, then it’s quite hard for us to follow up.
M1	So, can I just ask that in terms of primary care, (with regards to) the success of a screening, is it accepted by a lot of patients? What is the success rate of them coming for all their routine screenings? That means, even for the normal patients?
D	D. I don't think we actually keep tabs on how many *[laughs lightly]*, the denominator of those we screen, and then, those who actually TURNED UP for screening, and those who test positive or negative. I mean, we DO have a results-and-acknowledgement workflow, but we don't really trace back whether the person has been encouraged by the doctor to go for screening or the person was actually (making an) independent choice (and is) aware of the risks that are involved and had gone for the screening alone, for example, to have called in directly for a Pap smear and called in the Singapore breast screen hotline for a mammogram. So, I think it’s fairly difficult to trace. Some will come with the letters sent by the government to tell them that they are due for the screening, and then from there, we then send them for the relevant screening. Some are very opportunistic - they have come to see us for something else and they fit into that age range and we send them, but we don’t actually trace specifically back whether this person had gone (for the mammogram) and what is the result of that, because of the very same-day-queue kind of patients that we have. So, unless they are our family physician clinic

	patients, we may then spend more time to look back at the results, because they are tagged to us for their chronic disease.
M1	So, in the family physician clinic, do you also make sure that they go for their recommended screening?
D	Ah, yes, and also for the vaccinations. And we have a little bit more time there too, and because of the longitudinal relationship, we more or less know what they have gone for and what they have not gone for, it is easier therefore to then flag up to the patient.
M1	Thank you. So, maybe because of time limitation, let's finish up with the last two issues. Do you know, are you aware of community resources for cancer survivors, and who do you think should be the stakeholders, besides primary care physicians and oncologists? Who else should be in the team to look after cancer survivors? *[pause; 33:41 – 33:49min]* Do you know of community resources? Because a lot of the other groups are not aware *[laughs]*, but I'm sure you all know, started giggling about it?
Unidentified male, likely E	I'm sure that some cancer society, you know, some support groups and all that. But I was just thinking of a rather frivolous suggestion: in our setting, maybe the TCM (traditional Chinese medicine) practitioners – *[M1 interjects, "Should be part of team, is it?"]*. I'm sure they are aware, and a lot of our patients DO go to see them. I think they should set the limits of what they can manage (and) what they should not be managing. So, when you think of an example, some good feedback, some observations that I have seen that made me think that certain TCM (traditional Chinese medicine) practitioners are good, we have seen patients who have had falls and had gone to these TCM (traditional Chinese medicine) practitioners, and some of these practitioners, if they suspect you have got a fracture, they will actually not touch the patient and they will say, "Go see the doctor. You have a fracture. You may need an X-ray.", so we know there ARE some practitioners who are pretty good and you know, for the very fact that they are popular among our patients. And they should be kept in the loop. We can't ignore them, because our patients ARE going to see them.
M1	So, they are popular?
Unidentified male, likely E	They are popular. They are popular, yah. But patients can't tell the difference between a good and a bad one *[laughs and others laugh too]*. So, the information should go out, I mean, not just to them, but to patients as well. And going back to that example that you illustrated earlier about the lady who grew (skin) nodules over her scar, so the very fact that she was seen by fellow GPs (General Practitioners), I think, and also, you mentioned that there should be a division of responsibilities, right? So, the healthcare provider knows that, but I think the patient also needs to know that. You know, some of them may think, "Oh! I'm seeing my GP (General Practitioner) and he didn't say anything, so I must be fine! I'm not going to… call earlier to go back to see my NCC (National Cancer Centre)

	doctor, because of that, because my GP (General Practitioner) just saw it and it's fine.". It could be a false -
M1	*[Crosstalks]* - they trust the GP (General Practitioner) more?
Unidentified male, likely E	Yah, that's right, but we may be missing something, you know?
M1	Okay. Do you have any last things to share?
Unidentified female, likely D	I know the really long-term cancer survivors themselves can also form their own counsel or support groups for patients who are undergoing treatment for cancer in the earlier years of cancer survivorship, when they still have follow-up very regularly. I think that experience would help patient normalize their feeling(s), their grief, their loss, (and they know that) there are programmes in life, and (that) they can still find meaning in life, so I think this is one group (who) will be the strongest advocates, probably even more reasonable voices than us.
M1	Thank you. Any last words? If not, thank you very much.
All participants	Thank you.
M1	So, we will email the details to you for the reimbursement. I think they will either GIRO or -
	[Audio recording ends at 37:02min.]
