# Supplementary material for: Implementing a community-based shared care breast cancer survivorship model in Singapore: a qualitative study among primary care practitioners
Source: BMC Prim Care. 2022 Apr 8;23:73. doi: 10.1186/s12875-022-01673-3 (PMC8991467; doi:10.1186/s12875-022-01673-3)
Supplement: Supplementary file 3 — Additional file 3. A compressed folder containing the raw data transcripts and demographics data collection form. [file 12875_2022_1673_MOESM3_ESM.zip › Supplementary Information File 3/FGD Senior (08.31.2018).pdf]

Transcript for Focus Group Interview Senior 31st August 2018

Key:

Moderator / Interviewer:	M2
Respondent:	Participant A (A)<br>Participant B (B)<br>Participant C (C)<br>Participant D (D)<br>Participant E (E)
( ):	Paraphrases, additions to or rectification of grammar, vocabulary and/or truncated sentences.
[ ]:	Non-verbal, e.g. *[xx laughs] [pause]*
…:	Removal of false starts, repetitive or ungrammatical long phrases
CAPITAL LETTER:	When there is a louder emphasis or stressing on a particular word or phrase

M2	Okay, let me introduce myself, so at least I get to know all of you. My name is M2. I am an associate professor with *[name of institution; omitted for reasons of confidentiality]* and *[name of institution; omitted for reasons of confidentiality]*. I'm actually a pharmacist, but I have a huge interest in health services research, and mainly in toxicity management in cancer patients. So, I also have an appointment in *[name of institution; omitted for reasons of confidentiality].* But I'm part of the core group where we're looking at how to discharge patients from the specialized centre, which is NCC (National Cancer Centre), to the community. So, we've been doing projects to figure out how we can do that. I mean, just like all the disease states like diabetes, dementia and what-not, our goal is really to decant. When you guys hear the word "decant", it's like, "Oh! All come in to you all *[laughs]*.", but I think before we even think about how to make it happen, I think we need to ensure that we understand what the barriers (are), in the session, for survival care for these cancer patients. As you know, cancer is one of the main disease state(s) out there among our Singaporean population. The challenge that we're facing is that, of course, the numbers are going up *[trails off; unidentified female arrives and asks, "Are you waiting for someone?"]* Hello! We've already started. So, we have a lot of new cancer diagnosis, particularly with breast cancer, colorectal (cancer) and also like some of the other solid tumours, like prostate cancers and so on. And many of these people get well-treated and they become survivors. So, I guess the problem here that we're facing at NCC (National Cancer Centre) is that we're not able to manage the load of the survivors, so we are trying to think of creating models and things that we are able to get patients to be seen in the community. So, this is why we think it would be important to talk to you guys. So, in this qualitative focus group discussion, your identity will be anonymous, so which is why you've been given a label here. A, and you're B and you want to be C or D? *[everyone laughs]* Up to you. Okay, D! D sounds bad, that's all *[laughs]*. You know, it's like we all don't want to be "D doctors" right? *[laughs]*. So, anyway, but your views will be highly valued,

	because we want to ensure that we get an understanding of what is happening on the ground, so which is why we are taking this qualitative type of approach. Okay, so, in the next half an hour to forty minutes, I'll be going through some items. You can discuss whatever you want, and if you agree or disagree, please voice out. But every time when you start talking, make sure that you mention you are Dr So-and-so, A, B or D, just so that we know who you are. Okay, so I'm going to jump straight in. The first question that I have for you is something is very straightforward: did you see any cancer survivors in your current practice, and if you have, what kind of experiences do you have with these survivors? We can start with anybody.
A	Okay, A. On and off, yes, they do actually come and see us for other problems. They can be coming for like acute problems, like URTI (upper respiratory tract infection), or back pain that kind of thing, but not really cancer-related or cancer medicine related.
M2	Sure, okay. But if you want to quantify like how frequent(ly) you see these survivors, how would you say? Let's say, in the clinic every day, how many of these people would have cancer history?
A	Maybe just a handful in a week?
M2	In a week? Okay. Do you all agree, disagree? *[A few participants reply, "Yes."]* Agree? Agree? Okay. Besides seeing these people with their acute problems, like you mentioned, maybe (for) URTI or whatever it is, what else have you seen among these people? Have you encountered people who've got problems with their cancer treatment, physical or psychosocial issues that you have observed?
B	Yes, I'm *[trails off; M2 prompts, "B."]* So, once in a while, I do see patient(s) who come for, like, newly-started on medicine by cancer doctors, oncologists, be it for their cancer or their cancer-related side effect(s), (as in,) the medicine are for their cancer-related side effect(s). So, sometime(s) they do come to us for like (reasons such as), "Oh, this medicine was increased recently or was started recently, and I have developed these (side effects). So, do you think that the side effects are actually related to the medicine itself?". Or I recently saw one who is a cancer survivor and who (came) mainly for back pain but of course, it may, CAN BE related to the underlying cancer itself. Of course, I don't know … what kind of cancer status is this patient at, at that point of time. So, these are some other less commonly seen (cases), but I've seen so far for cancer-related (cases) for cancer survivor patients.
M2	Okay. Do they have a lot of questions, do you think, or do they mostly kind of know that these are issues that they need to go back to cancer centre or NCC (National Cancer Centre) or -
B	*[Crosstalks]* – most of the time, they are actually quite anxious, although they know they need to wait for their appointment to NCC (National Cancer Centre) before they can clarify all these. But a lot of time(s), when they come for some other things,

	they will "by the way, I will ask you about this", see whether the doctor know(s) and can explain to them.
M2	I see. Any experience coming from you, like people you've met, who are cancer survivors?
D	I'm D. So, so far, I haven't really experience much problems. I do know that there is a same-day walk-in clinic in NCC (National Cancer Centre), (so) whenever they develop any side effects of drugs. I think my only experience… with oncology patients, (is) actually maybe one with multiple myeloma and was started on some drug. So, she actually came to the polyclinic for rash. In the end, I just called the specialist directly, and gave an early "TCU", so that's my approach, if I were to see and encounter any problems.
M2	Okay, okay, okay. So, to summarize, you probably didn't see a lot in your practice, maybe a handful that you see every now and then, but mostly because they come in for acute issues that are not related to the cancer. If they have any other problems, they might highlight, but you may just refer them back to the cancer centre. And that's okay! I mean, I think based on what we've heard so far from other focus groups, this is kind of similar in terms of what we're hearing. So, what we are currently working on, proposing, is to figure out whether we can do something called the "shared care model". The shared care survivorship care model is quite well-used in the United States and in Australia as well. The way it works is that the oncologist will still be seeing the cancer survivors, but of course because the frequency will decrease over time, because they are quite well, so what happens is that they only come back maybe every six months or every couple of months or so, but the appointments would alternate between the primary care provider AND the oncologist. So, obviously, you can already tell *[trails off to greet a participant who just arrived]* Hey hello! You can already tell that the issues that you may encounter with this sort of model, because… we need to ensure that there might be some of communication bridge, just so that everyone is kind of in line, knowing, understanding what is going on with the survivorship care. So, which is why you all will see the survivorship care plan. This is actually coming from the American Society of Clinical Oncology. And this is a care plan that kind of list(s) out all the different kinds of information that will be brought upon to the visits in primary care, so (it's a) treatment summary of what happened to the patient's cancer history, what exactly are the follow-up plans, so, for instance, like, do they need mammogram at what time point, do they need an MRI, do they need a pelvic exam or PAP smear because they are on Tamoxifen or something, or do they need a colonoscopy and then, you know, what are the other symptoms that they have, and how should we manage these side effects. So, obviously it will be an electronic version at that point if we're really implementing something, but this is kind of a draft of how the survivorship care plan will be like. I guess the *[trails off]*. Hello! I guess the next phase *[trails off]*. C, we're going to *[trails off]*. So, we're having this focus group and we'll address each other as A, B, C or D every time, so that your identity stays anonymous. But then, when you express your opinion, you've got to

	say, "I am Dr Whatever.", and then you just tell me your responses. So, I guess this is kind of a care plan that we are thinking (of putting) together, but electronically, it will be delivered. I don't know whether it will be through NEHR (National Electronic Health Record) or other means or so. What are your thoughts about doing a shared care sort-of-model to see cancer survivors with the oncologists? What are the first things that pop up in your mind?
A	Okay, I'm A. So basically, in primary care, especially in polyclinic, we don't have much exposure to the cancer survivor patients and their related complications, be it with the cancer itself or … from the cancer medicine. So, firstly, I think we need education. So, educate our doctors (on) what are the signs and symptoms that we need to look out for, the cancer recurrence, as well as the side effect(s) of all the medicine, be it acute side effects or long-term side effects. So, education. Secondly, (it) will be the time and resources, like, I think in the questionnaire, we actually all answered (how), usually, we have about five to ten minutes per patient *[M2 and A laugh]*, and that's the time that we have only. So, cancer patient(s) may come back with some psychosocial issues, which I don't think five, even ten minutes of consult is enough. So, we actually really need a lot of *[trails off]*. If (we're) really going to start this shared care model, probably (what) we need is some sort of specialized clinic in the primary care itself *[M2 interjects, "Ah, okay! Interesting perspective."]*, like maybe just (in) one hour, just see one to four patient(s), or three to four patients(s), just to cater to this.
M2	I think the vision is (that) very likely, those people who get discharged from NCC (National Cancer Centre) and to be seen in the community are those who have low risk for complications. So, if they are very high-risk, meaning that, let's say they get chemotherapy and they have heart failure or something like that, these people are likely to be kept at the NCC (National Cancer Centre) setting. But for those who (are) multiple years out (of treatment), maybe they are just on Tamoxifen – I mean, I'm talking about breast cancer here – then those are pretty low-risk patients, if they don't have a lot of side effects. But, you brought up some very interesting point(s). For instance, you mentioned education. What are some education that you think is important to provide to family medicine doctors or polyclinic doctors?
A	Like the medicine cancer drugs, what are the side effects that they need to look out for and what are the surveillance that we need to do in the primary care setting.
M2	Okay, is there anything else? Others?
C	I'm C. I think medication interactions are also useful to know. We are seeing a lot of patients who have chronic disease(s), who are also cancer survivors. So, when you talk about following up for cancer, it's not only coming for surveillance for their follow-up. Along with that, a lot of them will bring along all their other chronic disease(s) that we are following up (on), so we definitely will need more time for them, as well as knowledge about how the medication interacts with the other chronic disease medications.

M2	So, medication-related. Anything else that you think that are important? Do you think the training should be just didactic, (or) do you think you need clinical attachments and things like that?
Possibly A	Clinic attachment will be good, like to see how the oncologists actually manage these cancer survivor patient(s) at their end, because it's shared care, so they alternate between the primary care and the oncologists. And also, another way would be GOOD support from the tertiary centre, like (at) any point of time when we are seeing this group of patient(s), if we have any question, there must be… an accessible way for us to have a consult or phone call or something like that.
M2	So, at the polyclinic level, do you think it is good to sort of do psychosocial sort of education, or even like, let's say, counsel, because you know, a lot of our cancer survivors may have lingering anxiety about recurrence. Or from our studies, we know that side effects from the chemo(therapy) give them a lot of anxiety. Do you think that can be managed at the polyclinic level in the shared care?
B	I'm B. As I mentioned just now, *[laughs lightly]*, if it's just our normal general clinic patients, I don't think we can do that in such a short consult time, unless there is a special clinic to actually deal with this type of patient(s), and we (are) given more time, and of course, with proper training, then I think that's not a big issue. The time is actually a big issue to us. *[M2 interjects, "So, time is a big factor?"]* Yes.
M2	Okay, okay. What are your thoughts?
D	I'm D. I'm looking at the checklist and I think it's a very holistic way to look at (and) address patients' concerns. But the truth, the reality is, you won't even have time to do THIS among our patients. *[M2 interjects, "Yeah, so for instance –"]* So, that makes it very difficult to do that -
M2	*[Crosstalks]* – yah, so that's something we've been wondering about and I do agree with you. So, for instance, like, because one of the main things for these LOW-RISK cancer survivors is that you want to prevent them from going back to bad habits, because I think one of the main things for - at least for addressing in survivorship - is (that) you want to, for instance, promote tobacco use cessation, you want to promote, like, maybe, healthy lifestyles. Do you do that on (a) regular basis to your patients? I mean, you can be very honest, because (your identity) is all based on alphabets here. But especially for cancer survivors, it's even more important. So, do you think that it should be something that should be done at the polyclinics?
Possibly A	We do promote healthy lifestyle in almost all our patient(s), who are on chronic follow-ups, yes -
M2	*[Crosstalks]* - but this group -
Possibly A	*[Resumes]* – I mean, not only (for) cancer survivor patient(s) (but for) everybody, especially for those patients with diabetes or just high blood pressure, chronic diseases. We DO promote healthy lifestyle, but having said that, with the five to ten

	minutes consult – sometimes (even) less than ten minutes – some of them have some other medical problems, let's say, for example, diabetes (glucose level) can be very high, so we might be spending more time on titrating the medicine, asking around. So, rather than *[trails off]*. So, we don't have much time in every visit to promote healthy lifestyle, like it's not every visit that we can actually ask them, "Have you quit smoking already? Have you cut down already?", or "How is your diet"?" and all that. So, (it all) go(es) back to this time factor. We don't really have much time because of the heavy load.
M2	I see. Do you guys, on routine, refer your patients to nurses or anyone for counselling for, let's say, smoking cessation or (such) things, which I PERCEIVE is important for cancer survivors? Do you think there can be resources devoted to survivors that way, in the polyclinics, like, besides yourself, but *[trails off]*?
Possibly B	If it's a general lifestyle-modification kind of counselling, I think our nurses… are quite good at it. Unless you're talking about specific thing(s), for example, (like) smoking cessation, I think they DO get SOME training. But over the years, no one has been referring and they have not been conducting those training, so those specific (issues), for example, smoking cessation, they might not be used to it anymore. So then, it depends on what kind of cancer survivor(s) you are talking about. So, let's say, the cancer is related to lifestyle modification which are related to diabetes and all these, yes, I'm sure our nurses will be able to do well. But if you are talking about smoking cessation, I think as of now, (they are) not very good (at it) *[laughs lightly]. [M2 replies, "Okay, okay."]* Then, the other thing is that whether the patient is confident enough *[laughs lightly]* in being managed by primary care doctor(s) as compared to their oncologists. I guess when you talk about cancer oncologist compared to a primary care doctor, of course they will trust their oncologist more than a primary care physician. So, also, (a longer) time (is) taken to build rapport before they actually trust our word for it *[laughs]*.
M2	So, I think which is why, in a shared care model, I'm one of these people who believe in a few things: (firstly), I believe that this should be introduced very early on in the patient journey. It cannot be something like, you've seen the oncologist throughout, and then, at the end, "sayonara" *[Japanese, translated to mean "good bye"]*. And then, the next appointment is going to happen at *[name of polyclinic; omitted for reasons of confidentiality]*. You know? Patients are not going to come. So, we probably need to have navigators in the very beginning, and then, introduce them (to) ideas about the fact that this is YOUR patient journey, and you know, YES, at this point you are getting treated at the cancer centre, but then we'll have trained healthcare professionals that will take care of the survivorship care. If we don't implement it this way as a MODEL, then patients will not be confident also that this is the way how they are going to be managed. But of course, it needs to be tried out. I mean, it's easy for me to say. In some previous focus group(s), we get some very similar ideas as well, that they also feel that the family medicine (physicians) or GPs (General Practitioners) may not have a lot of experience of managing cancer survivors. But what are (the) things that you think that you can do at the cancer

	centre level to overcome that? And for instance, other than education, what else do you think that WE can assure that our survivors… are getting quality care from you guys?
Possibly A	Adequate support *[laughs]*. *[M2 clarifies, "Support? Okay."]* Yeah *[laughs]*. So, not only like whenever we have any query about maybe a certain drug, side effect or symptom, there must be somebody that we can actually reach out (to), we can just pick up phone and call immediately, with the patient sitting right in front of us.
M2	At this stage, do you think that this is possible, like let's say you want to get in touch with the oncologist or just to communicate about care plans, like let's say you want to clarify about something, is that possible?
Possibly A	Currently, yes, sometimes we do (it) on and off, but we have to look at… what's the name of the consultant, and then we have to call the hospital, and then after that, get transferred, a call to the doctor, and so on and all that, so it actually take(s) up some time.
M2	So, seemingly, time is a big lingering issue, isn't it? *[a few participants laugh]* Any other suggestions, things that can instill more confidence in our survivors to JOIN the shared care model?
Possibly B	Patient education? *[laughs; M2 laughs too]* Yah. Probably, I think that's very important as well, to educate the patient (on) what are the things we can do and what are the things that the patient actually requires the oncologists' assessment, rather than *[trails off]*. I don't know whether it's just culture or what, (but) I realize that more and more patients prefer to see specialist for simple things that actually primary care doctors can manage very well. But a lot of time(s), they'll just say, "It's okay. Let me just see the specialist and see what they say", although the specialist may say the same thing as what we said. So, those are like minor things, like osteoarthritis, (therefore) not to mention that it is a cancer, I guess most of the people would actually want to see the oncologists more than us, if the patient education is lacking.
M2	But I think people also need to understand that they are cured. So, you are E now. So, every time you speak, you just let me know that you are E, because it's anonymous. I think the issue here is that a lot of survivors… don't acknowledge the fact that they are cured from the treatment, even from cancer, even though they have gone through all the treatment, they are being told that their cancer is already gone, but they still have this lingering feeling that they are still having that cancer and they wanted to go back to the oncologist. But I mean, are there anything different or things that they can offer? Which is probably not the case. There probably isn't much that they can offer. But can I ask for everyone's opinion – if we have survivors really coming to you, and let's say, you need to do referrals and things like that, what are other stakeholders that you think should be involved in this shared care model? Like you mentioned that oncologists definitely need to be communicated (with), but what are other components that you think that are

	LACKING within the polyclinic, that you FEEL that there is a point that we should engage in the process?
C	I'm C. I think you may need a patient care coordinator, and probably a social worker who's -
M2	*[Crosstalks]* – but there are social workers in the polyclinic?
C	*[Resumes]* – we have one social worker who's here only twice a week, and we see six hundred patients a day. So, I think if you are talking about cancer centre, that you're going to send more here, you may need to get a dedicated social worker, because like you said, they need a lot of psychosocial support, so I don't think our current social worker may be able to cope with this.
M2	How about rehab(ilitation)? Like, do you think that we have enough rehab(iltation) support within polyclinics to -
C	*[Crosstalks]* -Rehab support? I don't know about that *[laughs lightly]*.
M2	I'm talking about, maybe to encourage more exercise or *[trails off]*. Because some of them may have certain symptoms after their cancer treatment, like a lot of them have neuropathy, right? So, how do we ensure that these people are safe to go for exercise and things like that?
E	I'm E. We only have physiotherapists at certain polyclinics, such as *[names of three polyclinics, omitted for reasons of confidentiality]*, so it's not (in) all polyclinics. And if I'm not wrong, these guys are also quite overloaded, so they can only do three sessions per patients, and that's it already. I'm not sure if it's the best -
M2	*[Crosstalks]* – do they actually run these sessions at the polyclinic?
E	At the polyclinics which have physio(therapy).
M2	Like *[name of polyclinic; omitted for reasons of confidentiality]*, you were saying?
E	Yah, it's at *[name of poly; omitted for reasons of confidentiality]* itself, the physiotherapy class, but it's only for three sessions, and then the patients (are) told to leave already.
M2	Okay. So, what type of patients do you refer (for) this physio(therapy) nowadays, let's say you are at *[name of polyclinic; omitted for reasons of confidentiality]*?
A few participants	Neck pain… I think muscular skeletal kind of problems.
M2	Oh okay! Which our cancer survivors have a lot of these symptoms, as you all probably know, a lot of it.

Possibly B	But do they usually need special equipment for their rehabilitation or physio(therapy)?
M2	Depends. So, if their symptom complex is… very severe, or there are a lot of lingering symptoms that they can't manage on their own, then probably, yah. So, we also use *[name of hospital; omitted for reasons of confidentiality]*. Do you guys ever refer patients to *[name of hospital; omitted for reasons of confidentiality]* for physio(therapy)? No, you don't do that?
Possibly A or B	No, we can't.
M2	Oh, you can't. You can't! How about patients with osteoporosis? That's another one big one. You know, we have *[trails off]*. You guys manage a lot of those people, right? *[laughter from a few participants; unidentified female replies, "We can't!"]* You can't?
A and B	There's no BMD (bone mineral density test) that you can order. *[M2 probes, "So, what happens if you have to -?"]* We refer to the hospital? The specialist? *[laughs]*
M2	I see, I see. But then, isn't that a private rate?
Possibly B	If they go and see a specialist, they have subsidized rates in a subsidized clinic, then the BMD (bone mineral density test) (is) ordered by the specialist.
M2	Not by you? But can you guys do more? So, let's say you have somebody start off on Fosamex, can you guys monitor? Because a lot of our breast cancer survivors… have bone loss, so would you be able to monitor?
Possibly A or B	*[Crosstalks]* – we used to monitor BMD (bone mineral density test) -
C	*[Crosstalks]* – we have the KNOWLEDGE to monitor. *[a few participants laugh]*
M2	*[laughs]* – we ALL have the knowledge to monitor!
C	We have the medications, but as of current (practice), we do not have access to BMD (bone mineral density test) direct ordering. So, that is one roadblock. So, that is the roadblock, but otherwise, we are actually capable of monitoring. It's just more of the access to the services *[M2 interjects, "The logistics?"]* Yah. So, perhaps like what you mentioned earlier about social support and all, if we can actually tap (on) the NCC (National Cancer Centre) or the hospitals' support services, which is actually larger and probably (there is) more staff and support *[M2 laughs]*, so perhaps that can still be considered -
M2	*[Crosstalks]* – actually, WE are also sorting our in-house *[laughs lightly],* because we *[trails off].* I don't know if you guys know, but within NCC (National Cancer Centre), we DON'T have our in-house dietician, physio(therapy), none of that. A lot of people

	assume that we do, but actually we have none *[laughs lightly]*. We only have social workers. We have a HUGE psychosocial oncology department, but actually they are mostly MSWs (Medical Social Workers) who go for means testing, you know, making sure that our patients get their drugs, that kind of thing *[laughs]*.
C	I guess there's just not enough allied health support.
M2	Seeming(ly), seemingly. Do you guys also know that Singapore Cancer Society has resources for cancer survival? Do you know of any, like what services do they provide?
A few participants	No.
M2	No. They actually have a community survivorship rehab(ilitation) clinic, and they have gyms. They have a gym over at "JEM" *[reference to a shopping centre]*, which is over at Jurong. It's kind of far but… they have in-house physiotherapists, dieticians, and people who are *[trails off]*. I feel like this is an educational session now *[laughs]* rather than a focus group! *[a few participants laugh]* But yeah, they have those, and I think -
Possibly A or B	*[Crosstalks]* – there's one centre for that entire -
M2	*[Crosstalks]* – so far, only one centre, yah, but it was opened, like, two years ago? But they have a very low referral rate.
Possibly A or B	Okay! Who can refer? Anybody?
M2	Anybody.
Possibly A or B	Self-referral by patient?
M2	Yeah! So, you see *[trails off]*. I mean, as long as you are a healthcare provider. Actually, you can go to their website. They have referral forms that you can *[trails off]*. I THINK SO. I might be wrong, but -
Possibly A or B	But must be referred by a doctor?
M2	Yes, that you think that this person is in need. But I think in the process, I think we'll also need to engage the SCS (Singapore Cancer Society) as well, because they also provide a lot of philanthropy and resources as well. What else? What other resources do you think that we should also look into for our stakeholders? Do you think the nurses here can see the cancer survivors? *[inaudible murmurs from participants]* I see the reaction. *[laughs lightly]*

Possibly A or B	They are quite overloaded. *[laughs]*
C	*[laughs lightly]* They are also overloaded.
Possibly A or B	Yah, very overloaded.
M2	They are overloaded?
Possibly A or B	Yeah. With our chronic patients *[laughs]*.
M2	Okay, do you see any fairly successful programmes, that are so-called "decanting programmes" from hospitals also, that you feel that cancer survivorship (programmes) can mirror?
C	Currently, we only have - in our *[name of polyclinic; omitted for reasons of confidentiality]* - dementia clinic. But (for) the dementia clinic, we just started the decanting.
Possibly A	Yah, so dementia programme is a programme which was started few years ago already. Yah. But if you (ask if) it is successful, I think it's along the way, because we have the support from the neurologist that actually comes down to the clinic almost every session. *[M2 clarifies, "Oh really?"]* Yah. And then, we are still training the nurses for this occupational therapy as well, for the dementia patients.
M2	Oh! So, the nurses deliver the care?
Possibly A	In the pipeline, yeah. They are undergoing training at this moment, so currently we only have clinics, but no therapy or anything else yet. But in the future, the nurses are going to do it.
M2	I see. But these are clinics that are run by you guys?
Possibly A	Yes, yes.
M2	Wow! Oh, okay, okay. And how long is the clinic session? I know you guys are talking about (how) time is precious.
Possibly A	Correct. So, it's a special clinic, so we only have three sessions a month and we see about five to six patients per morning. That's all. *[M2 clarifies, "Oh, so not too many?"]* Correct, so about forty-five minutes to an hour per patient.
M2	What were the motivations for those patients to be followed up in polyclinic? Why do they come all the way to see you?

Possibly A	They are actually our *[trails off]*. Most of them are our chronic patients here. So, they are concerned about memory, so they get referred to be see at the clinic. And then, we can start medicine; we can follow up with their care.
M2	So, the touchpoint is actually from you guys? *[A replies, "Yah."]* You identify patients who are in need of dementia help? *[A agrees, "Mmm."]* Okay. Do you think this model is successful?
Possibly A	In a way it's actually *[hesitates; M2 interjects and laughs, "You can be honest! You can be honest!"]* along the way to success? Yes, yah, yah, I can say that it's on the way of BEING successful, yes, because… we are starting to see patients who are actually stable, (have) dementia and on medicine already from the neurologists, and they actually come back to us. And then, we are seeing them in our own clinic, in this dementia clinic, like, for follow-up four to six monthly kind of thing.
M2	Is this a shared care model as well, that they also go back to the neurologist?
Possibly A	Errr, now, because at the beginning, they started with (it) being a shared care kind of thing, that means that (every) six months, they come and see us, (and every) one year, the neurologist will see, so the neurologist will see (them) once a year. But now, the neurologists have *[trails off]*. Because they actually have one neurologist who actually come(s) here as well, so that neurologist actually oversees the two clinics that they are running, and basically the patients are stable, (so) they don't need to go back and see the specialists anymore.
M2	But the specialist is at the polyclinic?
Possibly A	Correct, (but) not (for) every session, but about seventy to eighty percent of the time, he will be around, and we can actually call him for anything.
M2	I see. And this person is from NNI (National Neuroscience Institute)?
Possibly A	NNI (National Neuroscience Institute). *[M2 replies, "Okay, wow!"]* So, there is actually the time *[laughs lightly]*, PROTECTED, AND there is the SUPPORT. And there is education as well.
M2	But the load is very different when it comes to survivorship, because, I mean, if we're just talking about breast cancer, of course it's (just) one group, but if you talk about different types of cancer, it's quite A LOT actually, I mean, in terms of how we are going to be able to have dedicated time for these survivors to be seen – quite challenging. So, one other question that I wanted to find out is, … would you be interested to see survivors in the polyclinic environment? I mean, you can be honest.
Possibly B	If we're given protected time AND *[laughs]*, and then, proper support, education, as a primary care doctor, I think, yes, we really should see (them) as well, but with the adequate time and resources, yes.

M2	What are some motivations for you to see cancer survivors if you feel that *[trails off]*?
D	Yes, yes.
M2	You do? You do? But what are the motivations to see cancer survivors, if any?
D	Because family medicine prides itself in being holistic, so I think we should also extend to the patients with cancer, especially (for) the ones that are stable. I think family physicians can be trained to manage them.
M2	Okay, okay. So, primarily, you think training, as well as making sure there's protected time… are the most important concerns? What would be the biggest barrier, you think? I mean, aside from time, aside from *[trails off].* This is one of our last questions. Aside from time, aside from these sorts of resources, what are some of barriers, biggest barrier(s), for cancer survivors to be seen in the polyclinic environment?
C	You mentioned "barriers", (it's) for the patients or for the doctors? *[M2 clarifies, "For the patients."]* For patients?
M2	I think for doctors, you all have already said it's time and resources, but for the patients, anything else?
C	Maybe like what B said earlier about the patients' lack of trust or thinking that the specialist centre is always better. So, that may be one of the main barriers that we will need to go manage, because or else, the patients wouldn't want to come anyway. And in the end, they will just come back to us to ask for referral letter back to NCC (National Cancer Centre).
M2	So, how come the dementia model sort of worked? Like how come patients are -
C	*[Crosstalks]* – because they came from us in the first place, so they already touched base with us. But if you talk about cancer survivors, unless perhaps we are the ones who pick up a breast lump, we refer them, or maybe if it's one of our chronic patients, she's known to us, and then we somehow picked up a breast lump, we refer, and then, we continue follow-up for her chronic condition, and then perhaps maybe these types of patients will be the better ones to decant back, because the trust is already built, and they are familiar with us.
M2	But you notice from your practice that you don't see a lot of these people, right? Do you -
C	*[Crosstalks]* – actually, we see a lot of patients who are cancer (patients), who are also on follow-up with *[name of hospital; omitted for reasons of confidentiality]*. They follow up for chronic problems. So, we do see them, (but) it's just that we are not involved when you talk about their cancer.

M2	Like, in the beginning when they were first diagnosed? *[C replies, "Yah."]* VERY SELECTED group *[laughs lightly]*.
C	Quite selected. *[M2 interjects, "Yah, quite selected."]* But the other option is whether you refer them back to the main primary doctor. But the problem with our polyclinic is (that) they do rotate around the doctors, and if we are going into the team-let where we are trying to put them into certain groups of same doctors every time, so perhaps that would be a better way of achieving that rapport.
M2	So, you are saying that, I mean, if somebody is being referred back, they would automatically be slotted back to whoever they have been seeing before? Or you are saying that *[trails off]*. Because not everyone is being assigned to a specific family medicine doctor, right?
C	So, at this point, we still don't see that.
M2	I think that is another major challenge, because they will end up seeing *[trails off; C interjects, "Random (doctor)."]* Yeah. And that may not serve the purpose of building rapport. Okay. Anything else you want to add? I don't want to hold you guys up for too long for too long. I know it's more than two o'clock. Anything else you feel that it's important that we need to address for if we're really running shared care on survivorship? E?
E	I mean, I guess it'll be good to have a set of guidelines from NCC (National Cancer Centre) if they want us to follow up on it. And then, they should just tell us what exactly they want us to follow up (on) and when to refer back -
M2	*[Crosstalks]* – so, you prefer to have SOPs (Standards of Practice) and algorithms and -
E	*[Resumes]* – yah, yah, yah, so that we know when to refer back and what to do if this happens. It'll be good to have the specialist input.
M2	Okay, okay, so, but more so for family medicine doctors just to follow, to make sure that these things are being done at different time points, is that what u are referring to?
E	Yah, something like that.
M2	Okay, okay. But then, I think most of these will be addressed in the educational sessions or so, that will be provided, so part of these would also include some kind of GP General Practitioner) education. So, NCC (National Cancer Centre) DOES have a branch where they look into cancer education, and we do teach GPs (General Practitioners) and family medicine doctors about how to manage some of the cancers. But I think a more formalized programme needs to be done, and I think at this moment, we don't have something like this to offer. So, M1 was talking about, we were talking about whether we can work with Duke (University) or whatever

	and come up with some kind of programme. But at the moment, there isn't anything specific in cancer.
D	Because (in) polyclinics… quite a significant (amount) of the load is run by MOs (Medical Officers) that rotate every six months. *[M2 interjects, "Yeah, the residency one, right?"]* No, not residency, the non-resident(s). *[M2 clarifies, "Oh, the non-residents?"]* So, they may not be familiar with how to manage, and even if they have gone through the training, six months later, you are going to have another batch of MOs (Medical Officers), so they may not be aware of what happened the last time.
E	Yah, that's true. Actually -
M2	*[Crosstalks]* - thanks for mentioning that. I didn't even know. You were saying something?
E	Another concern is that if you want to have like a clinic to follow up with the survivors, it needs to be a special "protected time" kind of clinic, because we can't really squeeze these kinds of patients into our general clinic when there are so many patients, but yet if we pull doctors aside to run the special clinics, that will be a FURTHER burden on our manpower, which right now is currently running very low, so that's a concern also.
M2	Thanks for the honesty *[laughs]*. I think if we are really doing this in the beginning, we will definitely need to run it as a resource model, and as a research programme too, even to understand why do patients like to come here for follow-up. And the second thing (is) of course, how feasible and how effective (is it) in terms of management as well. So, I think we're far from that, but at least coming up with the model is the first step that we need to have here. Okay. Thank you everyone for your time. Can you help me fill up these forms, SPECIFICALLY with the consent as well? *[E clarifies, "This whole thing, one set?"]* Yeah, one set! There is also a demographics survey. *[E replies, "I see. Done already."]* Yes, yes, I'll collect it. Thank you, everyone! Thank you. Thank you.
Possibly A	I feel about the education for doctors, I thought real exposure, that means sitting in with the specialists and see how they manage patients, personally I feel that will be more useful, rather than didactic and you are trying to teach GP (General Practitioner) what are the things to know, because each patient has different needs and present differently. So, for me, I would think a lot of exposure by seeing these kinds of patients even before they decant to us, it will definitely be very useful, like, for example, they actually have this group of patients that can be decant to the polyclinic, they group them in one clinic for that day, then you send some FP (family physician) to go over and see how the specialists actually manage the patients, and SUBSEQUENTLY, to decant to our polyclinic. If in the end this is to work *[laughs]* -
	[Concurrent talking from other participants in the background is not transcribed and deemed irrelevant; 38:49min till end of audio recording]

M2	*[Crosstalks]* – we're actually thinking of something like that -
Possibly A	*[Crosstalks]* – because there is really no point for us to sit in and see those patients who have very, very severe (symptoms) or require the oncologist's attention, that kind of patients. If you can also group that group of patients in THAT ONE CLINIC and then we will go and attach and see how they manage before they decant to us, then it will be useful also. I think it will be FAR more useful than didactic *[laughs]*.
M2	Yah I agree. So, (for) the dementia programme, how was the education done?
Possibly A	The specialist is here - I think for the few months - I think the specialist is here ALL THE TIME at EVERY clinic.
M2	So, there was a specialist?
Possibly B or A	Yeah, and just for about one, two sessions, we went to the hospitals and sit in.
M2	At NNI (National Neuroscience Institute)? *[Unidentified female B or A replies, "Yes, NNI."]* Who is it by?
Unidentified female	*[names colleague; omitted for reasons of confidentiality]*.
M2	Oh My god! Mcare? *[a few participants laugh]* Yah, he's too kind!
Possibly B	And in the initial period, he is here for ALL the session(s), right? *[mumurs of agreement from other participants]*. And then, the senior consultant was here.
Possibly A	So, they sit in for all the sessions. And then, they will SEE the patient with you *[laughs]*. That's really a lot of *[laughs]* attention!
M2	Attention and devotion! *[laughs]*
C	But I think by taking out a specialist here, you probably can see more in your own clinic.
Possibly A	That's right!
C	And for that one day. *[A laughs in response].*
E	Sorry, is the employee number like compulsory?
M2	Ah, no, no, no, no! I think eventually, it will be. Well, actually it will be useful, because I think M1 said that it will be "giro-ed" *[reference to bank transfer]*, you see, so if you don't have your employee number, I wouldn't know if they know how to -
Possibly A	*[Crosstalks]* – they "giro" the money to you?

M2	But you have IC (Identification number)? You have IC right? They have your phone number, right? If anything, they'll call. Don't worry!
C	I think I will just *[M2 interjects, "They have your phone number, right?"]* Yah, yah. I just didn't bring my card.
M2	No worries, no worries. It's okay. Thank you.
Possibly A	No, I think it's theirs.
C	Thank you. *[M1 replies, "Just make sure I have everything."]* This is for us, is it?
M2	Yes, there is one additional copy of consent form.
Possibly A or B	Actually, this whole programme is started mainly because there are too many patients generally in the clinic, is it? The low-risk patients?
M2	Yeah, because that takes the time away from the very acute patients. Then, those survivors over there, they are been seen for three minutes, five minutes, and they go, and we realize that many of them actually have chronic illnesses. So, M1 is actually seeing patients at NCC (National Cancer Centre). She's functioning like -
Possibly A or B	*[Crosstalks]* - actually, I'm thinking, instead of decanting patients to polyclinic where we have a lot of our own issue(s), like seeing different doctors, probably seeing a doctor(s) that are not familiar with cancer survivorship, isn't it better to actually have to have, to actually employ an RP (resident physician) or a FP (family physician) to be there to see JUST purely this group of patients?
M2	That is what M1 is thinking. The cancer centre doesn't see that as a priority. *[speaks to other participants]* Thank you! Thank you! *[resumes]* I know what you mean though, I know what you mean.
Possibly A or B	Because at least they are more accessible to the specialist -
	[Audio recording ends at 42:15min]
