# Supplementary material for: Implementing a community-based shared care breast cancer survivorship model in Singapore: a qualitative study among primary care practitioners
Source: BMC Prim Care. 2022 Apr 8;23:73. doi: 10.1186/s12875-022-01673-3 (PMC8991467; doi:10.1186/s12875-022-01673-3)
Supplement: Supplementary file 3 — Additional file 3. A compressed folder containing the raw data transcripts and demographics data collection form. [file 12875_2022_1673_MOESM3_ESM.zip › Supplementary Information File 3/FGD (08.04.2018).pdf]

Transcript for Focus Group Interview 4th August 2018

Key:

Moderator / Interviewer:	M1, M2
Respondent:	Participant A (A)<br>Participant B (B)<br>Participant C (C)<br>Participant D (D)<br>Participant E (E)<br>Participant F (F)<br>Participant G (G)<br>Participant H (H)<br>Participant I (I)<br>Participant J (J)
( ):	Paraphrases, additions to or rectification of grammar, vocabulary and/or truncated sentences.
[ ]:	Non-verbal, e.g. *[xx laughs] [pause]*
…:	Removal of false starts, repetitive or ungrammatical long phrases
CAPITAL LETTER:	When there is a louder emphasis or stressing on a particular word or phrase

M1	Okay, start recording. Thank you everyone for coming to our focus group discussion today. We will cover six topics and we'll start with the first topic, number one: background survey on current practice. So, the question is, "Can you share with us some of your experiences with cancer survivors?". We shall start with A.
A	I'm A. I work in the central part of Singapore as a GP (General Practitioner). *[M1 probes, "What is your experience with cancer survivors?"]* We do see them on and off when they come for chronic disease management.
M1	Thank you, A. How about B?
B	I'm B. I'm working in the north part of Singapore in the polyclinic. We often see cancer survivor patients as a part of the chronic disease management as well. And sometimes, we also manage their acute conditions.
M1	C?
C	Okay I'm C. I'm a private GP (General Practitioner) in the west part of Singapore, and… in the GP (General Practice) setting, I've seen only very few cancer survivors.
D	D. I'm working in the northern part of Singapore in a polyclinic. We do encounter a lot of patients in our routine chronic care follow-up. If the patients have got any acute complaints, we do manage their conditions in the polyclinic.

M1	E?
E	So, I'm E. I also work in the polyclinic in the northern part of Singapore. We do see a few - not many - cancer patients, mostly for chronic disease follow-up.
M1	F?
F	Hello, I'm F. I work in a polyclinic in the northern part of Singapore. We do have a fair amount of cancer survivors whom we see. We manage their acute and chronic care. Thank you.
G	I'm G. I'm working in a central polyclinic and we do see quite a few cancer survivor(s), usually for follow-up for chronic (diseases) and also see for acute problems.
H	Hi I'm H. I work in a polyclinic in the west of Singapore. In our clinic, we do see quite a few cancer survivors. We are usually seeing for follow-up of their chronic diseases, as well as miscellaneous acute conditions.
I	Hi, I'm I, and I'm working in a polyclinic in the central part of Singapore. The cancer survivors that we see in the polyclinic, usually we follow up with them for chronic care and acute issues.
J	Hi I'm J. I'm practising in the east side of Singapore in one of the polyclinics. I do see a number of cancer survivors. They come from acute care and also chronic care. From experience, they tend to have concerns about their cancer, and whether they are actually still healthy, whether there's a chance of relapse, and I do feel that I may not be that adequate in managing some of their concerns.
M1	Thank you, doctors. Let's go on to the second topic. The second theme is on "Discuss the perceived barriers of the proposed shared care model.". The question is, "What are some of the barriers that you can foresee with this shared care model? You can discuss them in terms of patient-related, physician-related and healthcare-related issues.". We can open to the floor. Anyone would like to contribute?
A	I'm A. So, for healthcare-related issues, for example, if, like, a breast cancer patient has a limb swelling and we'll like to refer them back for imaging, that might pose logistic(al) issue in being able to order them in a primary care setting, and also, the speed (at which) we can order them might be delayed. Thank you!
M1	*[pause; 5:06 – 5:15min]* H?
H	I'm H. So, regarding the physician-related issues, I think that most of us do not have very much training in cancer disease, so I think there would be some knowledge gaps for the average primary care practitioner. And also, the way that our clinics function is that it's a very fast-paced, high volume load, so I think that they may be some time constraint in managing some of these potentially more complex conditions. Thank you.

M1	Thank you.
J	I'm J. Adding on to (what) A and H have shared earlier, from the patient's perspective, I think I'm afraid that there may be some lack of trust (as to) whether primary care physician has the right skills set to help with their complaints and their concerns. And in terms of the healthcare system (barriers), there may need to be improvement in the infrastructure, whereby we can communicate and refer patients to and fro the hospitals. *[M1 probes, "J, can I ask, what do you mean by 'improvement in the infrastructure'?"]* So, J again. In terms of the infrastructure, it's probably in terms of the resources, and also, how fast we can communicate with and to the hospital to get the right treatment advice, the right investigation advice.
F	Hello, I'm F. In addition to… (what)… J (shared), I would actually like to comment on a healthcare-related reason. I feel that there's still much to be improved within the bridges between primary and secondary care. One comment I'll probably like to say is the lack of information that we have on the outpatient consultations that happened in tertiary care. For example, all that we are able to see are the results of the investigations done. We do not know what is the… management plan of the tertiary doctor, and this would serve to be a limitation in our management of this group of patients in primary care setting. Thank you.
M1	Thank you. Anyone else see any perceived barriers? Okay, if not, let us go on to the third topic together to gather feedback on the survivorship care plan to facilitate communications-planning. So, the question is "What information should be included in survivorship care plan?". Okay, let us look through the survivorship care plan together. So, the first part has the information on the patient, as well as the healthcare provider, in terms of the primary care, surgeon, radiation, the oncologist. So, there are two components to this survivorship care plan. First(ly), is the treatment summary, and secondly, is the follow-up care plan. So, in the "treatment summary", it will talk about the type of cancer history, and what was the treatment done, and in the "treatment ongoing", it will be the type of medications, as well as other concerns. And the follow-up care plan will be what are the things which need to be looked at after the treatment. We can invite comments on how do you feel, how useful is this care plan, is there too many details, or is there not enough information? *[pause; 9:33 – 9:51min]*
D	Hello, I'm D. I feel the treatment summary details are quite extensive. Just… "what is the diagnosis" and "what stage" and "what treatment they are undergoing", and "what we need to follow up (on)" should be fine. *[M1 probes, "Is it too much information?"]* Yah, too much information regarding the treatment summary, yah. *[M1 clarifies, "So, it's not really that relevant?"]* Yah.
M1	Okay, thank you. How about other doctors? What do you think?
F	Hello, I'm F. So, I'll just like to comment that I think what would be useful in primary care is perhaps a couple of lines or a segment on what discussions were had with the patient and the family, and which is probably something more subjective rather

	than objective, so that we will have a better understanding of how to continue with their care. Thank you. *[M1 probes, "Are you referring to the care plans, F? What conversations are you referring to?"]* Conversations, meaning subjective, so how did the patient feel on discharge, whether they are coping well with the diagnosis, so that we are able to manage them holistically, including the psychological aspects. Thank you.
M1	Thank you. So, coming to this point about the psychological issues, do you think as primary care physicians, you are in a better position to manage and are you able to cope with it?
F	Hello, I'm F. Yes. Thank you.
G	I'm G. I think the information given is quite extensive, but they are important. The worry is that with the amount of information, for example in the last page, they ask about… whether the survivors may experience any issues with the areas stated below, (what if) there are quite a lot of issues, let's say, if the patient ticked a few and input, for example, "fatigue", "depression", or "anxiety", "memory problems" and "weight changes", each of them… are big topics for even a family physician to test, so they go through a lot, and that would eventually become at least five or four chronic consultations, that will easily take away one hour of our time, (and) which is limited, and we have our own responsibilities to chronic patient(s) and acute patient(s). The second (thing) to add on would be that it would be good if we have a direct phone number with the consultants or the specialists in-charge that we can directly call, and they will answer the phone call, and they know that they are responsible for the day, that means they are responsible for the patient, rather than (when) we call, - even if we have the number and we call them - they seem to be not sure what are they doing, which is happening for some of the projects that we went through with the tertiary centres.
M1	Maybe can you elaborate on the projects have encountered?
G	We went through a few. I mean, for example, (for) one of the projects, we collaborate with one of the hospital specialt(ies), their daily specialists involved are responsible for phone conversation(s) with the polyclinic doctors, and what happens is that sometimes when we call them, they never pick up the phone. Sometimes we call, they pick up, and they do not know that they are responsible for that case, for that project. So, it become(s) a problem. And sometimes, when we call them, they KNOW that they are in-charge (of) that project for that day, but they just make it very hard for us to communicate, for example, to try to like call them.
M1	Yes, thank you. Anybody else?
B	*[pause; 14:29 – 13:35min]* Hi, I'm B. So, this treatment summary and the survivorship care plan for breast cancer (survivors) is a bit extensive. And I would be happy if it's going to be summarized in the form of the stage of the disease, the

	specific treatment that is given to the patient, and what symptoms and the side effects the patient had, (like), so far, what are the side effects, the primary complaints and the secondary complaints, or the primary issues and the secondary issues, which of the issues we, as a primary care physician, have to address, and so far, which are the issues that have been settled and the patient is coping well. If this is given in a summarized way for us to manage, it will be better.
J	Hi, I'm J here. Can I just clarify, this survivor(ship) care plan is actually a copy of cancer treatment summary and follow-up care plan kept by the patient, and the patient is supposed to bring (it) to the primary care provider, every time they hand this to the clinic?
M1	Yah, what we propose is for this care plan to be given to the patient at the end of the acute treatment, when there is a plan to share the care with the primary care provider, so the patient (will bring this) as a physical paper to the primary care provider for the further continuation of treatment.
J	And for ALL breast cancer survivors, when they are discharged – not even discharged, but - on regular follow-up in the tertiary centre, are they actually given a copy of the last page of this care plan, whereby they are supposed to tick off the different symptoms that they may have to discuss with their doctors?
M1	So, currently, in our practice setting, we have not started this care plan, so we actually want to invite your comments and feedback from the primary care, as to which areas are useful.
J	Because if I'm a patient, from a patient's perspective, these things will be quite overwhelming for me to actually sit down and think whether I'm facing all these things and these things should be discussed with my doctor. Perhaps a better way of doing is if the primary care providers could be provided a template of what are the things that we should look out for, for this breast cancer survivor patient, as a form of template in our clinical records, so as to guide us what to ask the patients when they come back, whether is it related to osteoporosis or depression or something like that, rather than having the patient to SIT DOWN and look through what are the things that they actually are facing and I should discuss, because RIGHT NOW this is NOT even done with the current patients in the tertiary centre, (hence) it may be overwhelming for patients to bring back to the primary care providers.
M1	Thank you. Anyone else?
A	So, I'm actually A, a GP (General Practitioner), a private provider, so, a few things on the last page like "financial advice on assistance", actually I find it hard to help the patient, because I don't have access to a medical social worker. In the end, we will have to refer them back to the hospital to get referral to MSW (medical social worker), so maybe there are certain limitations as to *[trails off]*. Maybe polyclinic may have access to MSW (medical social worker), but in the private provider, there

	are things that we don't have access to, that they might need the care of for such patients.
H	Hi, I'm H. I just want to comment that this checklist given on the last page is actually very, very generic, and looking through it, it actually is something that we will go through with patient with any sort of chronic disease, not just cancer survivors. So, I think that if the form is more specific towards things that are specific to cancer survivors and their condition(s), then that will be more useful for us, because these are things that, I would say, most family physicians are *[trails off]*. I mean, it's within our area (of expertise), things that we know and things that we do… So, something more specific towards the cancer disease itself will be more useful for us.
M2	*[pause; 19:09 – 19:21min]* Is there any other input for Question Number Three, on what information should be included in the cancer survivorship care plan?
A	Hi, I'm A. I think that the resources you may be interested in can include, like, some breast cancer support group hotline or ways to reach them. I think, mainly those in the primary care setting, we might not be exposed to such support group(s), so we might not know about such support group(s) existing, so if the information… is available for the patient, at least we can read more on it and offer them such resources.
M2	Any others would like to add on or else, we'll go on to the next question? (Are) there any others you would like to add? Okay, so next question, (we'll) explore some of the motivations to participate in this shared care model. Anyone like to share about this?
F	Hello, I'm F. *[laughs]* I guess one motivation would be maybe a bit more (of) altruism. We sort of *[trails off]*. Being in primary care, … patients have better access to us compared to tertiary care physicians. And if we are managing other aspects of their chronic care, then probably taking on this group of patients with this shared care model would be good, in my opinion.
J	Hi, I'm J here. In terms of motivation, I think the first step is probably knowing the fact that we can actually do more for our chronic care patients, because if it is a patient whom I see often, with chronic conditions, and now I am also managing her breast cancer, breast condition, then, that itself, I can probably form a rapport with the patient; we have a… good working relationship, that itself is a good motivation for me to actually help them with their survivor years after they are diagnosed and treated for breast cancer. But from (a) practical aspect, some form of financial help would ALSO be useful, because we do also understand that it will take time off from our clinic session(s), and some of the issues that we discuss would be time-consuming, resource-constrained as well, so having some form of reimbursement or financial help would be useful.

M2	(Are) there any other points that is important, so that we can make it more practical especially for you to be involved in this care? *[pause; 22:39 – 22:47min]*
D	Ah yah, I agree. I'm D. I agree with J. In spite of time constraints in this shared care plan, we… can build a good rapport with the patients, and the patients also become more compliant to treatment and follow-up. And that improves the patient care, you know… bring the patient care to a higher level. Yah.
M2	Anyone else likes to share? Those are very good points made by J and D. Any others?
Possibly E	Can I just get you to clarify what do you mean by "motivations"?
M2	I mean, what is, in MY view, … "motivation" in this case, is that what (are) the higher goals to help the patient. I mean, "motivations", of course there could be (reasons such as) to do better for your patients in terms of holistic care. But of course, there is also (the) monetary (aspect). We won't delude ourselves about that. But I mean, what are your PERSONAL motivations as a doctor YOURSELF, (on) why you want to help your patient? I think everyone has their own reason, but personally for yourself, why YOU want to help? What's your(s)? Do you have any? *[pause; 24:16 – 24:23min]* E? *[everyone laughs]* I think you look as if you want to say something probably.
E	But I think if you define "motivation" that way, then, I mean, so our motivation would ALL be the same – we want to provide a more holistic care for our patients, in terms of managing ALL their diseases. And also, because they have more access to us, if they have any psychological or emotional needs, they can always come to see us, instead of having to call up their specialist every time. So, that's one thing.
M2	Ok, we'll go to the next question?
M1	So, one of the extra points we would like to explore is that while this shared care model is good, what are the proposed solutions whereby you think will be useful, such that this shared care model will be a viable programme, whether (it is) in terms of, like, say, financial, or whether (it's) in education, in training, or in other aspects? *[pause; 25:44 – 25:53min]*
B	I'm B. I would like to have some *[trails off]*. I think it'll be helpful if (there is) education given in the form of training, in the form of forum(s) where the primary care physicians can come in and hear or discuss with the specialists regarding the patients, clear their doubts regarding the treatment and management aspects. So, training programmes to update our knowledge… for whatever management that is going on hospitals, it'll be fine.
J	Hi, J here. So, is it possible if there is a platform, like a forum, whereby perhaps it is hosted by a specialist, an oncologist, whereby we can discuss cases that we may have or have any queries as well on this platform? And what would also be useful will probably be regular updates from the oncologists, whereby there are any new updates in terms of the treatment or care regarding post-breast-cancer survival and

	disseminate(d) from the tertiary centre to the primary care providers on a regular basis, that would be useful as well.
M1	Thank you, J. Are you referring to, like, Continuing Medical Education programmes at a postgraduate level, or do you think it's important to incorporate in undergraduate or even in other training programmes?
J	Perhaps not at an UNDERGRADUATE level. It'll probably be at the postgraduate level. We talk about CME (Continuing Medical Education), Continuing Medical Education and all, those are very sketchy workshops that we hold. But I'm talking about maybe a less formal or informal platform whereby it is adhoc, (whereby) updates from the oncologist's side can be more regular, rather than waiting for a six-monthly, four-monthly type of scheduled lecture. So, to make it more regular on an adhoc basis.
F	I'm F. I just also think that maybe adding some numbers that we could call, maybe, like a breast cancer nurse, who is usually fairly familiar with the patient load, (would be useful). I do not imagine that we would need to call her all that often, however if there's an instance where we do need to clarify something which would make a difference to the way we manage our patient and we needed to know the information fairly soon, then I would find that quite useful. Thank you.
M2	Is there any other point to add regarding education and training that would be useful for yourself in managing the patients?
G	I think it would be good that the tertiary centre would have a conversation with our management team that allow(s) us to go for regular attachment that is part of our working hours with the cancer centre, so that we KNOW what is the normal practice that is happening in the cancer centre, rather than just give us pure technical talk or discussion. We might not *[trails off]*. Sometimes, from the GP (General Practitioner) point of view, we might have overdone it *[laughs]* or underdo certain things. So, that would be very, very helpful if …. we could use our working hours as our training hours, rather than our private time as training hours. Thank you.
M2	Are you referring to solo GPs (General Practitioners), because they can't pay for that? For locums? *[someone laughs]* Which level of practice (are you referring to)?
G	Sorry, I think for solo GP (General Practitioner) or polyclinic, I think (for) both (settings, it) would be very helpful, but I think it would be quite hard for solo GPs (General Practitioners), because it is their financial *[trails off]*. I mean, they need to get locum(s) to help with their GP (General Practitioner), so they may have more (issues to overcome), rather than (just) financial. But for polyclinic, it would be easier, because (the) polyclinic would have enough manpower to cover each other, so we can send our doctors, who have interest in the topic, to be there in the tertiary centre for training. Thank you.

M1	Thank you, G. That is a very interesting proposal. Maybe we'll like to go deeper into that. So, if we do have a primary care doctor who's interested in attachments in the cancer centre, how about many sessions or over what period do you think it'll be good to have, such that the doctor is comfortable and feels competent in managing cancer survivors?
G	I think that would be quite subjective. What we can do is we make it a bit more objective. For example, the tertiary centre should have screened through the patient, have a proper slot, like half-a-day session or one full-day session, (so) for example if you have a criteria of patients that you would like to decant to the primary care centre, so (for) that group of patient(s), the tertiary centre should screen through and put it under a formal slot, for example, like Tuesday whole day. Then, the whole day (of clinic) will be run by a GP (General Practitioner), with the support from the specialist for a few session(s), then we can test-run in (the) polyclinic. Then, we can schedule for another formal training session maybe three months down the road for that kind of practice again, so that they can even out the technique and the knowledge. But that have to be *[trails off]*. We have to go through it first, before we can tell objectively how many times (it should be), because (for) some of the things, we might not be able to see it while we (are) discuss(ing) it, but we only notice it when we test-run the problem, the care.
M2	Thank you, G. Just a question: if you say the polyclinics, (how do) you anticipate how many doctors need to be trained, because they can't be seeing you ONLY?
G	If that is the case, then what we can do is, it depends on… what kind of patients the tertiary centre is going to decant. Let's say, they decant Stage One patient(s) who have (been) cleared from the cancer for five years, very stable patients, then we can easily train all our doctors to do it. That would be easier for the doctors to gain that kind of knowledge, and easier to care (for) this group of patients in (the) polyclinic setting. You are right (in that) we are not be able to run the full-day clinic for that few doctors to see that pool of patients. So, it would be the same as minor surgical procedures… For example, it would the same as seeing depression patient(s). We train all our doctors to be able to see the depression patient(s), the low-risk depression patient(s), and we can start medication, and when we think that it is not within our ability, we can refer to the tertiary centre, so that kind of practice would be easier for us to cope and to train our doctors. Thank you.
M2	Thank you, G. How about D?
D	Hi, I'm D. Instead of focusing to do it on "one particular day a week" that kind of thing to follow up on these patients, now we are doing the team-let care for the patients in polyclinics, (and we can) focus on education (and) training (for) those doctors in the team-let who handle the chronic patients, who would also be in the position to handle the step-down care of these cancer patients as well. Yah.
H	Hi, I'm H. So, just to add on, actually (in) the polyclinic, we do have these, what we call, "champions", whereby one or two senior doctors are actually trained in a

	particular area, for example, dermatology or cardiology, so having these so-called "champions" who are chosen just to go for this training, they may be able to just provide more experience for the whole clinic, especially when dealing with our more junior MOs (Medical Officers) who are in clinic side by side. Just for maybe more complex cases, they can be consulted on the floor directly to get some advice on the management of these patients.
M2	Thank you. (Are) there any other points you would like to add in terms of education and training? I mean, besides polyclinics, in the General Practice, anything, considering that (are) challenges there, especially in getting locums? Would you want it to be a particular time where it's more feasible in terms of training, besides weekdays *[laughs lightly]*? Weekends? Saturday afternoons or mornings? I heard that Saturday mornings are very busy, not the best time, but is there anything (else) in terms of the family physicians on the ground especially? *[pause; 35:49 – 36:00min]*
A	I'm A. So, actually I think what G says is good *[everyone laughs in response]* because we have family and weekends are quite precious. In terms of, I think, encourag(ing) the private sector (to take) up shared care for cancer survivor patients, it has to come from the monetary value unfortunately, because similar to the PCN (primary care network), the companies are actually given a certain sum if they are able to fill up their… CDMP (Chronic Disease Management Programme). So, with that, actually a lot of – at least I know of some – companies…have done their best to set up their PCN (primary care network) just for the monetary value from the government, which is actually quite substantial. So, if the government is able to put aside a certain amount, I'm sure the private company will send their doctors for training during office hour(s), in order to be able to train their doctors to see this set of patients with the government support.
M1	Thank you, A. I would like to invite your opinions on whether it is useful, important, or not relevant, either to have, like, a short course on cancer survivorship education, or even to have another graduate diploma on cancer survivorship or oncology, would it be useful?
E	I'm E. *[laughs]* Sorry, I keep forgetting. So, I feel that when we see these cancer survivors, it's always as part of a chronic disease management or when they come in for any acute problems. So, one way to do it (is that) instead of a specialized diploma in cancer survivor, you can integrate it into the existing programmes we already have, which is like GDFM (Graduate Diploma in Family Medicine) or the MMed (Masters in Medicine) level *[laughs]*. *[unidentified female mumbles, "Is it like Palliative Medicine?"]* Yah, a diploma, but palliative? *[unidentified female mumbles, "To integrate it?"]* Yah. A diploma in Palliative Medicine is something *[trails off]*. I mean, integrate it into something we already have, because we don't see them just as cancer survivors – they come with other things like, we want to monitor their

	general heath, we want to also prevent the same cardiovascular diseases and all these things.
M2	Thank you, E. Is there anything *[trails off].* So, with regards to diplomas and extra training, it's not going to be necessary? Is that the view or (are) there any contrary views to that? Or it should be in pre-existing training programmes where it should be enhanced? Because cancer patients are a sizeable proportion of our population.
J	So, J here. When we are looking at this shared care plan, I will be looking at NOT a big proportion of cancer survivors because we are only looking at the early-stages ones. So, having just a postgraduate training on this topic itself may be an overkill, but I agree with E it could be integrated into a part of the postgraduate training. On top of that, perhaps if there were two-day workshops or some adhoc training possibly, it may be useful as well, but not to the extent of having a structured programme, postgraduate programme.
M2	So, would you consider college skills course a particular useful way of doing it?
J	So, that would be a way to do it.
M2	Anyone else would like to add (on)? I would say I agree with, D, is it? J and D. J and D agree. Anyone else?
G	I'm G. I think if we can do it in our working time, I agree with that. *[everyone laughs]*
M1	*[laughs lightly]* Thank you, G. Just now we did say that there is a lack of patients' confidence in primary care, so do you think having, like, a certification, would help to increase the confidence?
F	I'm F. I do think things like patients' attitudes towards physicians (et cetera), trying to change that would take time. It also depends on whether the patient who (has) survived cancer has an existing relationship with the primary care provider. The vast majority of patients who come to polyclinic are so used to seeing a different practitioner each time, so I *[hesitate, laughs and trails off]*. *[M2 probes, "Yes, doctor, you have any concerns? You have the team-lets, I understand?"]* Yah, there are the team-lets, but even within the team-lets, you get one of three (doctors). Yes, you MAY get to see the same doctor each time, but it's not confirmed. And if we were then to going into the private sector, patients actually can pick any doctor they (want to) go to.
J	So, J here. So, I think to answer the question on whether having a certification will help raise confidence in the primary providers, personally, I DON'T think having a certification will put us slightly higher in terms of patients' confidence level, because (for) our local population, I don't think they really look at the doctor's certification. In fact, they may not REALLY understand what the difference (is) between the primary care and also the specialist care plan, but they put their trust mainly more in the institution, rather than in the individual doctors. And as what F

	has said, as long as you have a relationship with your patient, actually they DO trust you. So, the certification may not help.
F	F. I agree with J. I just forgot the question halfway *[laughs; everyone laughs too]*. But I do agree that it's not just (about) the certification, but it's more (about) the relationship and the care you have provided thus far, and the trust the patient has in you.
B	I'm B, hi. I think certification is not going to help to build the relationship between the patient and the primary care physician anyway. But what I observe in the polyclinic is that (for) most of the patients who come, they have trust in their primary care physician; they ask so many questions regarding their conditions, which they wouldn't have asked the specialist. They'll say that tertiary care, they don't explain more, so they come to the primary care, they ask for explanations for everything, (like,) "Why this? Why that?" and so many things, so I think we need frequent CMEs (Continuing Medical Education), so that we can upgrade our knowledge, so that we can explain to the patients in a nicer way. So, that part (helps) - the confidence and to increase the rapport between the patient and the primary care physician.
M2	Thank you, B. (Are) there any other points that you would like to add? Okay, the next question: why do you think are or should be stakeholders in this shared care model, and the possible barriers that affect the communication and seamless coordination AND transition of care? *[pause; 44:28 – 44:36min]*
M1	Ok, I'm the facilitator here. Maybe I can elaborate on this. So, we are thinking that, you know, should cancer patients be mainly managed in the tertiary centre by oncologists, will there be other people who would be able to contribute more to survivorship to have holistic management of the survivors themselves? Besides primary care physicians, oncologists, who else do you think can contributes to their care?
A	Patients with past experience - *[M2 interjects, "A."]* Oh sorry, I'm A. Patients with past survival experience and who have gone through similar disease, for example, breast cancer support group, they actually bring hope to the current patients and can give good advice, so I think that is a good resource.
B	Hi, I'm B. Apart from primary care physicians and the oncologists, I think caregiver education is really important, so caregiver(s) should be educated on how to handle their minor complaints, and then, they should take (them) to the primary care physicians if it's needed. And it depends upon the type of the patients, whether it's stable patients or it's like Stage One and Two, or Stage Three or Four patients with other complications. So, if it's going to be Stage Three or Four patients with more complications, maybe home care nurse can also address the patients' problems as well.

D	Hi, I'm D. I notice we have the Advanced Practice Nurse handling the chronic care of the patients with diabetes, hypertension and other things, (therefore) the Advanced Practice Nurse can maybe (be) involved in the cancer survivor patients for the follow-up. *[M2 clarifies, "When would you expect the APNs (Advanced Practice Nurses) to be? In the -"]* In the follow-up of the – *[M2 interjects, "Sorry, in the practice or in your polyclinic or where would that be?"]* In the polyclinic, as well as in the tertiary centre.
H	I'm H. I think another area that can be expanded is also our allied health providers, so physiotherapist, dietician, psychologist, (et cetera), I think they all can play a bigger role as well in the management of this kind of cancer survivors, in terms of nutrition, in terms of mental health, and then, in terms of also the cancer relapse as well, and so, physiotherapists will be important in that area. So, there are a lot of these allied health workers that have important function(s) that just the doctors themselves are not able to provide.
M2	Thank you for the feedback. But can I also ask that, in terms of these resources, are they available in your settings, either in the GP (General Practice) or in the polyclinic settings?
H	So, there is allied health in the polyclinic setting – most polyclinic do have allied health, if not onsite, then at least in the neighbouring clinics. I think in the private sector, it's a bit harder to get access to this, but at the same time, I am not very sure regarding (their) capabilities as well, whether they are trained, whether these *[trails off]*. Because the physio(therapists), for example, in the primary care, … have different expertise and different facility compared to the tertiary centre, so I'm not sure if they are actually equipped or trained to deal with this particular kind of rehab(iltation).
M2	(Are) there any other points? Thank you so much, H. G?
G	Yes, I'm G. From my observation (of) those patients that I have seen for chronic disease(s) (and) at the same time they are cancer survivors, I notice that those people who have religion(s), deep trust (in) the religion, they seem to be coping well. So, if you really want to involve some other organizations…, I think religion can be considered there. Thank you.
M2	So, I think there are two parts to the question: what affects communication, seamless organization and transition of care? I think that was touched on, as you've said, you would like to have more feedback. Is there anything else that could affect communication, seamless coordination and transition of care?
I	Hi, I'm I. So, in terms of the transition of care, sometimes the patient may present acutely, and is still concurrently seeing the specialist as well, but we are unable to see the details of the clinic notes that the specialists have input inside, and we don't know what (are) the plans or their impression of the case, so we can only just make an informed GUESS to figure out what exactly are the specialists trying to do to

	manage the patient. So, then we just do our best for the acute component, and we can just follow up with a memo back to the specialist. So, especially if let's say, it's in different clusters… right now, you can see, in terms of like, if you are in one cluster, you can see what medicines have been ordered by the specialists, what investigations have been ordered by the specialists, but if you are cross-cluster, you really can't get this information, so therein lies the barrier in the transition of care.
M2	Thank you. Is that a problem that you think would be same problem for everyone else? Is that a universal problem?
A few participants	Yes, yes.
M2	Everyone said 'yes'? *[everyone laughs]* ok, yah. So, of course if there is the National Health Group NEHR (National Electronic Health Record), of course you can't see all the doctors' notes and everything, it's like the results and the radiological investigations, is that possibly, at the moment, useful, good enough or not good enough?
F	Hi, it's F. I really don't think it's good enough. Investigations, yes, you don't know why they did the investigations; you don't know HOW the findings and the investigations were managed; we don't know when they went for the last outpatient appointment, what transpired there, was the patient feeling okay and what was the plan that they doctor had for the patient. So, I think NEHR (National Electronic Health Record) probably still have some way to go. Thank you.
M2	Thank you. All right, the community resources: who are the community resources available and WHOM we can engage, refer for effective shared care? So, I assume, in this case, the community resources available would be, for example, community health centres, for example CDC (Communicable Disease Centres) or FSC (family service centre), cancer societies also, or befrienders or any of the support groups, for example?
M1	In this topic, we'll really like to know the awareness among primary care physicians, who are the community resources that you are aware of, and whether they play a useful part in the shared care. Or you are not aware, it's also okay. *[everyone laughs lightly]*
D	Not aware! I'm D. We're not aware of the community resources to engage them *[laughs].*
M2	How many are not aware? *[everyone laughs]* Oh, for the record, everyone is not aware? *[everyone laughs]* What are the community resources, if you HAD a wish list, would you love to have, if you had one? If you wish you could have?
J	So, J here. So, I think we have mentioned earlier, support group(s) or hotlines whereby the patients themselves can call, if they have some anxiety about their condition, some queries, or even just a good respite where they can go to often to

	share their concerns with, (where) they journey alongside with people who have gone through similar conditions (et cetera), those would be useful.
M2	Any other points? Thank you. *[murmurs from other participants, "No."; pause 53:37 – 53:43min]* Can we just ask a question: do you think this programme, this cancer survivorship, is a FEASIBLE programme in your view, based on the current state?
M1	It's exactly the same question I want to ask as the facilitator. We just want to invite the floor to comment *[M2 interjects, "Free for all."]*, is it unrealistic, is it too idealistic or is it reasonable and it should be expected of every family physician?
A	*[sighs softly]* A. I think it's feasible if there's sufficient support, like the PCNs (primary care networks), government is willing to put money in it, so it's feasible. But if it's a lot of altruistic volunteerism, it might be difficult.
M2	Thank you, A.
F	I'm F. So, I guess it comes down to (how) it's very easy for the specialist to fill up this form, give it to the patient, and then when the patient comes and see the primary care doctor to give the form to them. So, in that sense of things, I think actually this is completely feasible. But whether or not the outcome of the interaction is … exactly how we wanted it to be, that would be like what A said, perhaps there needs to be the financial incentive and things like that, and more support. Thanks.
M2	Can I ask, what is the – how to say it – sufficient remuneration *[a few participants laugh]* that we consider as FEASIBLE, because we are mindful that otherwise, they will be hiring more oncologists to fill up the gap if the cost is too high. What would you say would be a REASONABLE level, so that we will consider (and) so that government would consider?
A	A. Well, it could be on par with chronic disease management, like for every diabetic patient or hypertensive patient, the government gives hundred dollars per patient. Cancer care is similar to chronic disease management, (therefore) I think if it's included, it shouldn't be a problem.
J	Hi, J here. I think to answer that question, there (are) a lot of assumptions that have been made, because in the first place, we're not even sure what will constitute a shared care plan, because for diabetes, we know every year you must fulfil this, this, this every year. So, for cancer per se, we need to be very specific but we're not sure, like maybe a mammogram or something, so things like that, that's the first thing (we need to know). So, to answer whether it's feasible, whether it can be done, or (whether) it's totally not feasible, I think the question here is more of probably something that we all, as family physicians, SHOULD be expected to do in the first place, because if this is MY chronic care patient dealing with post-breast-cancer condition, it's actually part of the package. So, it's not so much to think about whether it's feasible, but rather, how can we have better support, whether it is

	supported in this journey in cancer. I'm not sure, because personally, (I think) if it's a very early-stage breast cancer, (for) most patients, after five years, they are actually discharged from the tertiary centre, they are not even discharged to SOMEONE, (but) they are just discharged. So, I think having such a shared care plan will help to improve patient care, in the sense that at least the patient knows if something happens eight years down (her) post-discharge, (she) can go back to someone and talk to someone about it, and start, perhaps the process of doing investigations, or just an early diagnosis. Basically, someone is in-charge of it. So, it's probably having a handle tool for the primary care physicians to have A BIT MORE knowledge on the topic, and (we) hope to better our care to our chronic care patients. Thank you.
M2	So, you won't increase your charges, doctor? *[everyone laughs]*
J	I'm J. I work in an institution *[laughs lightly]*, so that's REALLY by institution, so I can't speak for my GP (General Practitioner) colleagues, but I can really see the challenge. I can really see the challenge. But I do feel (that) it's part of our care to the patients. We can't compartmentalize them, that "You have this Disease, A, B, C", so care is really quite fragmented, so it's good that at least we are providing holistic care to the patient. But monetarily, you really need to see what is expected of us for that individual patient, if not, it's very difficult to charge.
M2	Thank you. (Are) there any points to make on how we can do, I mean, whether this is feasible and what is?
H	I'm H. So, actually, I don't think it's feasible. Personally, I think that I just want to take a step back and it doesn't have to be like such a formal survivor care plan. I mean, if you want to treat cancer as just a chronic disease, there is no, like, diabetic care plan or post-stroke care plan and we just treat the patient as it is. So, I mean, there are certain nuances with cancers which I guess most of us are not so familiar with, but I think with some shared education, and overtime, with greater practice, then this will just come naturally, like how we treat our usual chronic diseases, then all this extra care plan and checklists, checkboxes (et cetera) may be excessive and create more confusion, and more "checklist-ing" during consultations, which I feel is a bit too much doing all these.
M2	How about the CPG (Clinical Practice Guidelines) with FM (Family Medicine) doctors involved? And just like (for) the diabetes, asthma, all the "CHAS (Community Health Assist Scheme) Twenty" whatever conditions, and we just add on cancer survivorship, not necessarily breast cancer, but it could be breast, colorectal or whatever, but for breast cancers in this case, what are your thoughts?
H	H. Yah, actually I do agree with that. Some sort of *[trails off]*. Just some sort of guidelines that we can take, not strictly as a rule but as some guidelines, that we can incorporate into our care of patients, that would be very, very useful. I think cancer is very, very important, so I mean, now we are talking about breast cancer, but every cancer has their own similarities, so to just have a general understanding

	of the disease, and in terms of long-term care, what we need to know is like chemo(therapy), post-radiation, what are the post-treatment complications that we should be looking out for, these are the things that we need to be aware of (,but) not so much of the cancer treatment itself. I think that's way beyond our level of practice.
J	J here. So, adding on to H, I agree that in terms of feasibility, we need to consider exactly what are we looking out for in these post-cancer survivors. We are probably going to deal more with their complications of treatment, and these complications, we are talking about long-term complications. And we do know that there are a lot of trials of chemotherapy drugs out there, so for every chemotherapy drug, we also do know that there are specific long-term side complications and I personally wouldn't be able to deal with all this information, because (for) different patients, we have different treatments, and different treatments entail different medications as well, So, there lies in how are we going to treat the complications to the point where we have either a guideline or a list of medications with possible long-term complications, and from THAT, we CAN help the patients better. So, I'm not sure how feasible this actually is.
M2	The CPG (Clinical Practice Guidelines), with the regular updates and family physicians on-board, would that be helpful?
G	I'm G. I think that would be very, very useful. At least we have something to base on, rather than experience *[trails off]*. Let's say if the patient (is) taking certain medication and do not know whether that is a common side effect or that is even a side effect that is associated (with the medication), at least we have guidelines to base on, and that would help, that would be very useful. Thank you.
F	F here. So, I actually want to add to what J mentioned. Yes, a CPG (Clinical Practice Guidelines) would be useful. The other thing that is useful would be that perhaps the oncology team would write some special notes which is actually very personalized to that patient, so (things like) what should we be looking out for, for this patient. The other thing (that) I want to just make a general comment (about) is that actually this piece of paper would really empower the patient. So, wherever the patient goes, whichever the doctor he sees, he knows that whatever that was done pertaining to his cancer is all there and he is able to show that to the doctor whom he's seeing. Thank you.
Possibly D	The CPG (Clinical Practice Guidelines), apart from the general framework, you can consider more on the common cancers, like colon and the breast cancers… the more common cancers that are being followed up.
C	I'm C. Actually, in the private sector, and actually I thought for the private sector, pertaining to feasibility, there's more time to engage the patient. Actually, I'm being a graduate of the DipDerm (Diploma in Dermatology) programme and they are also right-siting patients, and they will ask us to take on the right-sited patients, and they have actually come down to the clinic to speak to me with regards to right-siting

	their patients, and they have forms, and they have plans and everything, but I think there is difficulty because until now, I hardly see any right-sited patients *[everyone laughs]*. They DID come down once, and then many months down the road, they came down AGAIN and then said, "Okay, we are going to start.", and then we said, "Okay, we are going to start.", but I haven't seen it REALLY START *[everyone laughs]*. And the thing I heard so far *[trails off]*. Oh, then, they had ANOTHER conference on right-siting and ANOTHER meeting at the NSC (National Skin Centre), which I didn't attend already, the second time round *[someone laughs]*. But from what I heard so far, the difficulties they face (are) actually that the patients just get the medications cheaper from the tertiary hospitals, and in a private GP (General Practice) setting… I would think there are a few cases (whereby)… there are some GPs (General Practitioners) who WOULD WANT these patients - especially new clinics or whatever - they would want the business, so they would WANT to take it on and they might even go for training hours OUTSIDE their after-hours, just to be qualified to get this patient load…. So, I'm thinking that there might actually be some people who would WANT to do it, if not for altruistic reasons. But then, from the patients' point of view, it's that there are only some GP (General Practice) clinics that only run CHAS (Community Health Assist Scheme) and not for the Medisave programme also, so the patients cannot claim Medisave, and therefore they would still have to foot the bill from the ones that are only available for the CHAS (Community Health Assist Scheme). I mean, even if you put cancer as a chronic disease, so they are unable to deduct from the Medisave, so they (still) have to pay upfront. And therefore, patients don't like it because if they are in the tertiary hospital, they feel safe, their drugs are very cheap and they get all the drugs that they want that the private GP (General Practice) clinic may not stock (up on), like if they want to, like, for example, pick up their Tamoxifen or something like that, then they would rather be on follow-up with the tertiary hospital.
M2	Thank you, C. Thank you, all, for this very fruitful session. Thank you for your time for coming this afternoon. The session ends. Thank you.
	[Audio recording ends at 1:05:42min]
