# Supplementary material for: Implementing a community-based shared care breast cancer survivorship model in Singapore: a qualitative study among primary care practitioners
Source: BMC Prim Care. 2022 Apr 8;23:73. doi: 10.1186/s12875-022-01673-3 (PMC8991467; doi:10.1186/s12875-022-01673-3)
Supplement: Supplementary file 3 — Additional file 3. A compressed folder containing the raw data transcripts and demographics data collection form. [file 12875_2022_1673_MOESM3_ESM.zip › Supplementary Information File 3/FGD (08.25.2018).pdf]

<u>Transcript for Focus Group Interview 25th August 2018</u>

Key:

Moderator / Interviewer:	M1
Respondent:	Participant A (A)<br>Participant B (B)<br>Participant C (C)<br>Participant D (D)<br>Participant E (E)
( ):	Paraphrases, additions to or rectification of grammar, vocabulary and/or truncated sentences.
[ ]:	Non-verbal, e.g. *[xx laughs] [pause]*
…:	Removal of false starts, repetitive or ungrammatical long phrases
CAPITAL LETTER:	When there is a louder emphasis or stressing on a particular word or phrase

M1	Good morning everyone. Thank you for coming to the focus group. We have six guiding questions here, but feel free to bring up any new points. First of all, we'll go round and introduce yourself. Tell us about your practice and your experience with cancer survivors. Maybe we start with E?
E	Hi I'm E. I've been working in primary care for the last ten years or so, and I think my main contact with cancer survivors are mainly those with concurrent chronic disease, and with a clear treatment plan, and from simple things like, for example, breast cancer survivors may only have their blood pressure taken from one side or there's chronic pain issues, or there's some problems with medication or interaction with their medications between the cancer drugs and their chronic disease drugs. So, I've not met many with symptoms or specific concerns about their cancers itself. So, that's seems to be my current exposure so far.
M1	Thank you, E. D?
D	I'm D. So, I'm currently in private practice (for the) last four years. So far, with cancer survivors, like E has mentioned, most of the time they come in for chronic or acute complaints, which is, to me, reason for visit. Along the way, they MAY have things like a bit of Tamoxifen or chemo(therapy), so usually I see them, I just tend to look out for possible interactions and any side effects of the drugs, but they really seldom come in for cancer-related symptoms. Those (who do) usually are not the survivors; those usually are the palliative patients that come in with the cancer-related symptoms. As to (my) experience, most of them already have their own plan from the oncologists, (such as) when they are doing their screening, or for those who are in total remission, actually they may need even need much care for the cancers, so these are what we see in the clinics, so there isn't really any shared care so far.

M1	Okay. C?
C	Hi, I'm C. I'm a family physician who is in practice for about ten to eleven years. Same as the previous doctors, my experience with cancer survivors are basically either for episodic care, when they come in for some flu type symptoms, or secondly, they are actually breast cancer survivors with chronic conditions such as diabetes or hypertension. Rarely, we see some breast cancer survivors who come here and we pick up (that) perhaps (their) mood is a bit low, we pick up some depression or anxiety disorders. Most of these patients are actually on follow-up with the oncologists or specialists and will actually go back to these doctors for their breast cancer issues.
M1	Okay, C, you mentioned that (for) some of (the patients), you may pick up some low mood, in that case, in your setting, would you go on to find out more about their low mood?
C	Yes, in our setting, definitely, as family physicians, we need to be holistic, so we manage the psychology part as well. So, once you pick up (that) perhaps this patient has some low mood, we'll go on to assess for depression and treat accordingly, or perhaps contact the specialist, write a memo to say that we've picked this up in the patients.
M1	Thank you. B?
B	All right, I'm B, family physician in the government practice. I've been practising for about ten years as well. So, I think, quite similar to previous doctors, the main experience with cancer survivors are those patients who have been seeing us for chronic disease management, and on and off, these patients will actually come and see us for acute consults. Majority of the cases that I see actually (are) more of the colorectal cancer survivors, prostate cancer survivors, as well as, of course, breast cancer survivors as well. Intermittently, I would see patients who are actually having a chemotherapy at this time, but I guess, while they may come to us for certain side effects of the medications, such as oral ulcers, they don't want to keep going back to their specialists, so they come to their nearest family physician for a review. So, I think basically that's my main experience with seeing these cancer survivors. I just want to ensure that their chronic disease is well-controlled and they actually follow up with their appointments in the hospital.
M1	A?
A	I'm A. I'm also a family physician working in government practice and practising in family care for about eleven years now. So, I run my own family physician clinic, so I do have the privilege of seeing a few of my own chronic patients, so most of the patients I'm seeing are for chronic diseases. So, I think my experience with cancer survivors ranges from those patients that I've picked up to have cancers, to those who are currently on follow-up with the oncologists and I will co-see for their chronic conditions, as well as those that have been discharged from cancer follow-

	up, then I follow up on them. So, I think some of the experience, I think, (are) MY patients that I've picked up for cancer, that I've subsequently referred and their family members come to see me, because they know me from their family members, and the family members would have cancers themselves, (so) I'm seeing them. So, I think conditions would include colorectal cancers, as well as breast cancers. I think (for the) patients that are being shared-follow-up, I think I do see some patients who are on certain chemotherapy (and) they do experience worsening of their chronic conditions because of complications, which can cause their blood pressure to go up, or their sugar levels to go up. And subsequently, those patients would have been discharged. And I think like E mentioned, patients with mastectomies done, then we have the same issue of whether we can do their blood pressures, and those with bilateral mastectomies, we don't even measure the blood pressure from the arms.
M1	Thank you, A. So, it looks like our senior group here has a lot of experiences with the cancer survivors. So, just to elaborate on this point, so since you all have seen patients with a lump and referred them, do you think that (you are) happy with the amount of information you get from the tertiary centre? Do you feel like the care is very disjointed after the initial referral? Do you hear from the tertiary centre after that?
E	Sorry, E here. I think some of the specialists do reply promptly after the diagnosis is confirmed or the treatment plan is also confirmed, but more often than not, there is no… early correspondence. Only when… patient is treated to a certain extent and the doctor, the specialist thinks that now the bulk of care is (to be) handled by primary care. Otherwise, a lot of the information that we get before the memo comes, is either from the patient's own history, or we have to go on to the electronic, nation-wide records to try to find and sieve out as much details as we can. So, I think communication could be improved. And I think it's a matter of probably prioritizing resources and seeing how that goes. I also understand that very often communication is a two-way process, and from our side, I think more probably could be done, especially where there ARE cancer-related concerns or other things that sometimes the patient doesn't have the opportunity to share with their specialist, either due to a long wait time or it's maybe (due to) the perception that the primary care providers are better able to handle these emotional issues. I'm not saying that it's a common occurrence, I must say, but I think communication ON A WHOLE could be improved.
M1	The reason why I asked this is that from the previous groups, some of them feel that especially when they are private GPs (General Practitioners) and they refer the patient as a subsidized case, because they go through the polyclinic, and in the end, if the same patient comes back to the polyclinic, the GP (General Practice) clinic, they find that there is no information about what actually happened to this patient. And even worse, after the patient is shifted to another place, some of these private GPs (General Practitioners) feel that the care is really not ideal, there is really no

	communication, and (their) patient, in the end, is given to other people. So, how about private family physicians, do you all have any thoughts on this?
D	I'm D. So, I would think that it's more of where's the best place to take care of the patients in the interim. … If you ask me, I would say that a team-based approach is best, so if the patient ends up with the government sector, for example, the oncologist or the polyclinics where they have a better infrastructure to take care of cancer patients for that period of time, I don't think it is an issue for me that my referred patients end up there for a certain period of time. But in terms of communication, of course I think, truthfully, if I'm the specialist that sees so many patients a day, it's very hard for me to keep writing memos back to the GPs (General Practitioners), to update him what's happening to the patient. I think the current state where if they are planning to discharge or hand over the case, the patient, back to the GP (General Practitioner) and they are given a memo, it may be sufficient. In the interim. Same as E, I would just look up the NEHR (National Electronic Health Record) and speak to the patients to get some info(rmation) from them (on) what has happened and all that. But so far, I don't really foresee any issues if the patients end up in the government hospital in the interim. If the patient is very keen to come back and see me again, I'll be happy to have the patient back again, but as a solo practice, it's very hard to take care psychologically. If I need to refer for psychologists, you have to go to another place, (I) refer to some other areas, and then they have to go and see him. So, I think that the infrastructure in the government is still better, until they really clear off everything, and they are very, very stable, then of course the solo practice can just take them back.
M1	Okay, so thank you. So, we'll go on to the second theme, so we're talking about barriers now, having discussed your experience. So, what do you think are the barriers that you'll foresee, if we DO have this shared care model between oncologists and primary care? You can divide them into either patient-related, physician-related or healthcare-system-related (barriers), and then, at the same time, you can give us some proposed solutions?
C	I'm C. So, if you talk about physician-related factors, if we go on to talk about primary-care-related practices, some physicians in primary care may not be that comfortable attending to patients with oncological complaints. Basically, perhaps their training may not be that adequate to deal with this group of patients. Secondly, for physician-related factors, as you mentioned just now, it's more of the unfamiliarity and we also may need to improve the communication with the primary care physician as well, according to the care plan, for this particular group of patients. For patient-related factors, most of the patients, I guess, after surviving cancer, they may be more comfortable to go back to their own specialist - I mean, a certain group of patients to go back to their own specialist - to continue with treatment, instead of going back to a regular GP (General Practitioner) for assessment. Healthcare-related wise, there may be some operational issues as to what's really the best care model for them. That's my contribution mostly.

M1	Okay, C, I noticed that you said that there's inadequate training. Can you elaborate on that?
C	Let's say for family medicine training, I mean, we do have MBBS (Bachelor of Medicine and Bachelor of Surgery), GDFM (Graduate Diploma for Family Medicine), MMed (Masters in Medicine). At GDFM (Graduate Diploma for Family Medicine) level, even for onco(logy) modules, even though there's a set of notes, it's just that and we can only sort of recognize the symptoms and recognize red flags, but there's not much emphasis on post-cancer survivorship and what should we do for this group of patients if they present with certain symptoms. Similarly, for MMed (Masters in Medicine), I think it's really not emphasized in the curriculum for this group of patients.
M1	Thank you, C. How about A? I understand in the public sector, you do have care plans, practice guidelines (et cetera). Will that be adequate training?
A	So, I do agree with C in that I think a lot of our guidelines in the public sector are more tailored perhaps towards the more common chronic conditions and all that. I think when it comes to screening for cancer, I think (it's) not so much of an issue, because I think a lot of family physicians are quite sure of what to do, what to look out for. Perhaps (for) some aspects of screening for recurrence or screening for complications of the TREATMENT of the disease, the cancer and all that, I think that may be the thing that we're not so well-covered. But I think training notwithstanding, I think if there's proper communication between the specialists and primary care providers, I think that can help quite a bit. But unfortunately, I think, like E mentioned, sometimes a lot of communication doesn't quite happen, and a lot of what we find out about treatment to the patients actually comes from patients themselves. So, for the well-educated patients, I think (it's) not so much of an issue, but for those who have no idea what's happening to them, we can only rely on NEHR (National Electronic Health Record) or the electronic records to give us an idea. And (for) a lot of issues with the medications that the patients may be taking themselves, we probably have to end up looking at our own resources, whether it's subsidy or MIMS *[online drug reference directory]*, (in order) to look out for common side effects of some of these medications, and then to advise our patients accordingly. Of course, if the oncologist would communicate that a little bit better, especially if he know(s) that the patient has a primary care provider and the patient is being followed up for chronic disease, and he knows that some of the medications may cause a rise in blood pressure or rise in sugars, he should write a little bit on that, on something to look out for, for the primary care physician to monitor.
M1	Thank you. So, do you think it's expected or reasonable to expect the primary care provider to know the side effects of the medication?
A	I think it's a bit tough, because especially with the advances in cancer mediation, I think you guys are really looking quite far ahead in terms of the different kinds of

	medications available. I think, in terms of big groups, I think most doctors would probably be aware of some of the common medications, but I think for the very, very new ones, I think (for) the biologics and all that, I think it will be challenging to expect the primary care physicians to know.
M1	So, for the big groups, the basic medication, so it WOULD be reasonable to expect the primary care physicians to know?
A	Perhaps a little bit of refreshers would be useful for the primary care physicians? Yah.
M1	*[laughs lightly]* Okay, E, I understand that in your practice, you do have (and) you write guidelines about screening. So, will there be any plans to write guidelines for post-cancer, for survivorship cancer, or even to know the side effects of common medications?
E	I don't think we've gotten to that stage yet of writing guidelines for ALL physicians in government practice, but we do know that for practical resource-planning purposes, some of our oncology colleagues are already trying to give talks and trying to share resources on a very small-scale basis to try to educate us on the common diseases. And precisely, (for) the more common cancer with high survivorship, like breast, colorectal (cancers), those who are in earlier stages (are) supposed to be cleared of tumour. There's an initiative that they want to train us to be able to take care these (patients), but as you have outlined, I think there ARE quite a few barriers still. So, we haven't got(ten) to the stage where there's going to be an organized guideline yet on the survivorship care, but I do appreciate (that) there are a lot of issues in the three big areas you have outlined. (With regards to) patient (factors), just offhand, I'm sure if the patient and specialist have a good relationship, and already patient feels that the specialist literally saved (his) life, there's probably more trust to go back to the specialist, even for something simpler, other simpler -
M1	*[Crosstalks]* – that is the problem. I totally agree with E, because the patient is so grateful that the doctor has saved his life, so definitely he will want that doctor to follow up with them for their whole life(time).
E	Correct. Precisely. It's always this concern that WHAT IF, WHAT IF, the cancer recurs, and the first person to turn to out of convenience is the specialist, and they can at least work out whether there is another recurrence that needs to be treated promptly, rather than going through the process of primary care, primary care does some initial assessment, then referring back to the specialist. So, that's one psychological issue that many, I think, patients grapple with. So, that's one (factor). (With regards to) physician-related (factors), also, like what you've been saying, the amount of exposure and training and all that, at the primary care level, it's still quite limited. And I think even if we just deal with two cancer groups and just (be) familiar with common drugs, it's already a challenge, because you need to be familiar with the patient, you have to go back and make sure that the patient comes back and

	see you on a regular basis. And for some of our colleagues, that is a concern because sometimes that does not always happen, right? So, that's the thing. And as for systems issues, I think it's like drug availability, you know, like, cancer drugs, are they available? If the cancer *[trails off]*. If the patient has to go back to the hospital to get those drugs, why would they bother running to two places? (And) likewise, some tests that may not be done in primary care, so what, then? And then, there may also be certain allied health, psychological support that PERHAPS tertiary centres are most experienced with and resources are there. So, some of these things (are what) we currently don't have in primary care. So, again, the patient may be forced to run to several places to take care of ALL their concerns, so sometimes, given the choice, they rather stay in a specialist centre.
M1	Thank you, E.
A	Actually, I have a different thought about that. A here. I mean, I think the majority of patients are probably like what E has described, but I think the thing that's lacking there is the "one family physician, one patient" kind of thing, And I suppose the rest of the senior doctors here would probably agree that, I think, for some of the patients that we've been following up with ourselves, I think while we didn't SAVE the lives of these patients in terms of operating on them, we are the ones that made the diagnosis - *[M1 interjects, "That's right! It's actually YOUR patients, right? And you picked them up?"]* Yah, and the patient THEN comes back to us, asking us whether this chemotherapy drug is suitable for them, and then WE would be the ones advising them based on whatever knowledge *[laughs lightly]* we have, that "Yes, it's good. Yes, you should continue. Yes, perhaps you should have that discussion also with your oncologist. Or perhaps I can write you a memo to your oncologist to highlight some of your concerns as well.". So, I think for these group of patients who have already established that kind of relationship with their family physicians, I think it could go the other way round.
M1	Thank you. How about B? Do you think that in terms of training and care guidelines, is it very important? We know that there's time limitation in each consultation, and there's a template in which you need to complete, so if we put down all the side effects of the medication there and include the care and care guideline(s) into the template, would it be useful? Would it be practical?
B	I think to me, having all these guidelines will definitely be useful. It's just that, well, I mean, all these guidelines, we can read these for the first time and be very familiar, but overtime, when you see not many of these patients and there's no refresher, all these guidelines are lost and then, we'll forget about it. So, I think pertaining… to the particular question about the perceived barriers, from my experience with other specialties wanting to do shared care, there's probably a few things to (consider). It's like patients themselves… don't want to travel (to) too many places, and if their chronic disease is well-controlled and (they are) on simple medications, one of the few things that they always tell us is whether the specialist can just see, because they can do the usual tests and all those things. So, these (are) some things

	that we also grapple with. Although we tell the patients that "You can actually see us and let the specialist handle your main situation.", … we also don't want them to travel all over the place, and then THAT might actually lead to compliance issues. And (secondly), some of the specialists prefer to do shared care if they are able to view notes. Similarly, for us, I think it's very useful because we are actually able to view, from the electronic records, some of the notes that the specialists are actually writing down. So, in between, when the patients actually come back to us and see us, we can actually see what has been happening. And these patients sometimes will actually ask us, "Oh, actually we don't really know what's going on.", and so we try to interpret from the notes and we explain it to them, so I think this is sort of like a systems-related barriers, that, I mean, now that we're moving into this new generation of electronic medical records, if that can actually be solved, that would be useful. So, yah, but back to your question on whether these guidelines are important, yes, I think it's very important, because (of) all these medications, as what A has mentioned and everyone else (too). So, all these side effects, new things that come up, we need to be updated.
M1	Thank you. E, because regarding what you were saying about the non-availability of certain drugs like Tamoxifen, what if there is a way we can deliver the medicine to the patient's home? Would it still be feasible for the patient to come back and see the primary care physician?
E	Yes, that would be ONE solution. That model depends on cost, convenience, because the manner of which the things are delivered, what if the person is working and there's nobody at home to collect, is there some issue there? So, these are the logistics and practical issues to do with home delivery. And also, I think it's good … (to know) who's the one to write the prescription. If there is any need to change the drug dose or the type of medication and all that, and has bearings on other chronic disease treatment as well, then we have to make sure that, if it's the oncologist writing the prescription, is there enough communication to know that the prescription is the most appropriate at that time? So, I think that's one part of it, so, who writes the prescription, and what is supposed to finetune the practical issues and make sure of that.
M1	Thank you. So, in terms of that, can it be co-ownership in the writing of the prescription? That means, usually in the tertiary centre, the prescription is written usually for one year, so the patient actually comes back to the centre once every few months to pick up the medication. Would it be reasonable for the centre to ask the patient to follow up with the primary care for any side effects? I mean, as a primary care physician, do you think you'll be confident enough to manage the side effects of Tamoxifen, which includes, like, thrombosis or endometrial cancer?
E	So, depending on the more common side effects, so things like endometrial cancer, I think it needs to be worked out at the hospital, cancer centre, rather than primary care. So, if there is some kind of fast-track service, for example, for the common,

	expected side effects, and that access is easy, it'll be a much easier process for us, rather than for us to grapple with trying to get that organized on our own.
M1	Thank you. Any other thoughts?
C	I guess it's *[trails off]*. I'm C. I guess there must be a platform for easy access, as what (has been) mentioned by the other doctors just now. So, in this area of telemedicine technology, whether there (are) any thoughts of creating this communication platform via tele(communication) with the specialist AND primary care physician. That's something to consider.
A	Yah, A here. I was also thinking about the *[trails off].* I mean, using breast cancer and Tamoxifen as an example on the screening for complications, whether, again, the guidelines, I think, may be useful then. On hindsight now, when, say, I was doing my family medicine training in the GP (General Practice) department, I remember seeing patients on Tamoxifen and we would see them once a year, I think, for ultrasounds and PAP smears, looking out for endometrial cells on PAP smears. But now, thinking back on whether it's really evidence-based and whether it's actually really good screening, I doubt that was, so if the guidelines can come and help to guide physicians along in terms of what IS the best thing, and I think it's something that doesn't require so many resources, then I think it should be doable as long as there is a proper follow-up on the patient.
M1	Just to share, in terms of the tertiary care guidelines for breast cancer review, it's just a history(-taking) – usually we just ask them whether there's any leg swelling and whether (there is) any vagina bleeding. And then, usually, for the first five years, we don't even refer to the gynae(cologist) and it's just an annual mammogram. I mean, that is the only requirement. It's just that, as a primary care physician, do you feel safe enough to manage all these patients, if it's clinical examination and the history?
D	D. I think right now, the issue I have when I think of this is that when they are segregated, when they go to see the oncologist, it's all oncologist-related; it may be once a year screening and once a year kind of physical examination. But when they come into primary care, then it may be a bit tough, in the sense that how do we decide when you want to do a calf diameter. If they come every three months for diabetes, do you do it every three months, because you are the family physician and you are supposed to take care of everything, or there's a specific appointment so-called for this cancer screening once a year? So, it turns out to be a bit grey. The guidelines can be aonce a year mammogram, very easy to follow, once a year and you just make sure there is no abnormality, but actually they see us more than that, then how do we *[trails off].* Are we safe enough to do it once a year, or actually we are becoming more and more liable, and when we see them, we are actually taking care of their diabetes, and along the way we must make sure there is no DVT (deep vein thrombosis) developing because of the Tamoxifen? So, these are all the things I would think about, whenever we see patients with this supposed shared care. It's

	very easy to follow guidelines, but the moment they are out, they actually see you more than a year. When they see the oncologist, it's very clear-cut (that) "I'm just going there to see (the oncologist) because of Tamoxifen, 'KIV (keep in view)' side effects, therefore, (I do) the mammogram yearly.". That's one of the things. The other one (thing) I just wanted to say is, I think the greatest barrier is still the patient(s), because where do they decide which is their medical home, and do they think of a medical home on their part or they think that (they) are seeing the oncologist and the GP (General Practitioner) for different things that they are good at? So, to me, that is the biggest barrier. Physicians, we can train; we can change a bit of mindset; healthcare system, we can change (but the greatest barrier is with) the patients themselves. It's like A has mentioned, he saw the patient, he referred (and) they come back to him, it's very logical; they will say, "I want to come back and see A again.". But what if it's in a mixed (institution) and they see the polyclinic, (there are) different doctors and because there are many different stages, you have to do X-ray, give them the results, then they see different doctors (they are) referred (to), then when the oncologist wants to discharge them, where do they go to? And will they be happy to go to some place where the oncologist decided (for them) VERSUS their own image of a medical home? So, I think, to me, the patient may be the biggest barrier. In terms of medicines, like when you talk about Tamoxifen, I think there's one system the psychiatrist department (does). They actually deliver medicines to the GPs (General Practitioners), who take care of the discharged psychiatric patients. Then, if that's the case, they don't need to do home delivery. They just come down and see the GP (General Practitioner), GP (General Practitioner) says "This is correct. We'll continue with medicine.", then they will send down the medicine to the clinic, the patient at the clinic will collect it. That's one (idea) if you are thinking about medicine deliver. We can try something like that. But again, that's only one aspect of the very big thing you are talking about, this shared care.
M1	Thank you. So, the other issue which previous group(s) brought up is that of mammogram, because when the tertiary centre discharges the patient, and then they say, "Go to the polyclinic or go to the GP (General Practitioner) to do the mammogram.", often, they come back and tell us that the polyclinic does not do mammograms for cancer patients. Can you share with us does that happen in the institution that you are working at or in the GP (General Practice) setting?
Unidentified male, possibly E	I guess sometimes there's confusion over the guidelines in Singapore. The general, first guideline they gave is that if there was any history of breast pathology, we should not be doing screening mammogram, but there's also a little footnote that says that if it has been cleared for five years, they are deemed to be of almost normal risk, therefore they CAN go back. But the second condition is probably not so well-known and there's also some dispute, so sometimes patients may call up the breast screen(ing) hotline to arrange for an appointment, and the person picking up the phone may be another receptionist that may just apple the first guideline and reject the patients from doing so. So, there's been a few cases of

	confusion, as such. So, I guess it's also (about) maybe just reviewing that as a national screening process, is there education for ALL levels of healthcare professionals, so that information is currently given and the right guidelines are applied. So, well, we do see that once in a while.
M1	So, am I right to say it can be done in the polyclinic?
Unidentified male, possibly E	Yes, technically it can be done, but we need that clear documentation from the specialist that this must have this, so once that's done, that's fine. But it's usually those patients who call up and fix their own appointment, then there's a bit more (of a) problem because there's no doctor to screen through their past history.
M1	Thank you. Maybe we'll go on to the survivorship care plan. We've been talking a lot about communication. So, if we do give the cancer patient a documentation for you to keep, will this information be useful or is it be too much, or are there areas which are lacking? Okay, first of all, about (the care plan), so this in two portions. One (portion) is a treatment summary. Do you think the details are too technical? Are they useful to you, whether the breast cancer is estrogen-positive, whether it is HER2-Positive, the stage and what are the treatment that has gone through? Then, the second portion is on the ongoing treatment, that means, whether the patient is on Tamoxifen or on aromatase inhibitors, and there are some side effect listed there. Is the information adequate? There's also another portion which the previous focus group did feedback to us - they said that for certain areas like fatigue, it's very important to pick up, but there's no recommendation what to do with them in terms of numbness and pain. Are there more information that's needed and whether – the second section is on follow-up care – is this information enough to know when is that mammogram due and when is the next follow-up?
A	A here. So, I think having a comprehensive summary is useful, but in MY context, I'll probably keep it for record in case I need to refer to it ever again, but it's not really for management, follow-up with the patient. I think the comment about the symptoms, like fatigue or numbness, I think it's useful STILL to have, even though there may not be any recommendations on them, and we also know that sometimes we MAY NOT be able to do anything about it. If anything, it's more so that we know what patients come back to us with, so that if they do develop new symptoms, we know whether anyone has heard of that before, and perhaps we can reassure patients that "Oh, (that's) whatever you have.", and we are not too concerned, as compared to something which may happen, new, and it wasn't present on the discharge summary. And then, the comment on the follow-up care plan yearly, because, say, the primary care physician should be the one for them to follow up with, this would be useful if it was a separate section to identify, because at the end of the day, I want a quick summary about what I need to do, and what's my role then, and I don't want to have to look HARD to find it. So, if it was to be on a separate page or with a very clear header, rather than a sort of continuation of some other documentation, that would be useful.

M1	So, thank you, A. So, these symptoms, if you look at the part about follow-up care plan, actually they are very non-specific. It's anything that represents brand-new symptoms or are persistent or that you are worried about, then this should be brought up to your provider. So, if your patient comes to you in a primary care setting, is this information adequate for you? *[pause; 35:35 – 35:43min]* One of them was saying that this one is a hundred-dollar consultation; one GP (General Practitioner) was saying that it's too much information to provide *[laughs]*, you know, in a clinic setting.
D	D. I'm just worried about the presentation that the patient may come in with, (that) they may think of many things related to cancer recurrence and things like that, and we end up having to handle, allay their fears, which may be time-consuming. But of course, on the other side, we can say that these are things that will help us to pick up the recurrence, so it's just how many symptoms they come in with, versus how many visits, versus how many (are) true cancer symptoms, or things like that. I'm not sure if having this, maybe I feel it will be a bit (too) general, in terms of the three statements (and) the patient can get very worried reading all these (and think that) anything new may be related to cancer. That's my (thought).
M1	I agree with D that it can be quite challenging in the primary care, because a cough is actually important on a recurrence, especially if they keep coughing for a month. So, will it be difficult to assess all these symptoms in the primary care setting?
C	I mean, C here. So, at primary care level, I guess we get a lot of red flags, let's say, they come for a cough, but it's very challenging to say for sure whether this cough is related to his or her cancer. But back to this form, there's some issues also about the follow-up care plan, about later parts, (that) I guess a doctor receiving the care (plan) should be backed up by a multidisciplinary team, because there are some ticks, like, for insurance, and perhaps advice for financial assistance, which perhaps a solo practitioner may not be able to give all the counselling for. Yah, here's some thoughts about this.
B	Yah, so I agree with B, sorry, C. I'm B. So - I don't know - in a way, it's sort of like a screening sort of test for patients when they come and see us. I mean, it sort of reminds me of things like asthma control test (where we) have some idea of patient control. So, maybe the intent of this care plan may be, I would say, maybe… just to be clear, actually the intent is for this patient, to make sure that their chronic disease is well-controlled, to make sure that they understand that these are some of the things, the recurrences and the red flags, as mentioned, that has to be addressed. So, a lot of things like insurance, school and work, parenting and all that, even on a normal consult, we also don't know, as family physicians, how much we can actually help them with. But very urgent things that they may need to always remember when they come and see us, I think that probably is of more value in the care plan, and also of more value to us. So, I mean, of course, this mental health (and) all that is important, because we CAN manage. So, basically, when doing this document, (we also need to) come from (the) aspect of a family physician (on what)

	do we normally see these patients for and what we can handle. I mean, it'll be difficult to talk to them about insurance, for example. Then, (for) counselling, I guess actually some of us have some expertise in it *[someone laughs]*. No, not me *[a few participants laugh]*. But I think there've been some recent *[trails off]*. So, I think (we need to) really look at it from the aspect of the family physician, what we can deal with WELL, and then, what is important for them, and then I think it would be useful (for) the rest of the doctors.
M1	Okay, thank you. We have a question here about what type of multidisciplinary team do you think is required to support in the primary care setting?
B	*[pause; 40:13 – 40:19min]* Probably a psychologist, if probably need (one), in the sense, a financial counsellor, but that's more in the government sector. In the private sector, I'm not sure if it's something difficult.
M1	Do you have the resources in primary care?
B	Yes, we do.
M1	Psychologists as well? *[B interjects, "Yup."]* And what about physiotherapists? Social Worker?
A	So, A here. I think for a few of us from different settings, I think we'll have our own experience. I think some of us in the public settings would probably be a bit strong(er), in that we probably do have the support from the different services, like allied health personnel. Perhaps one of the multid(isciplinary) persons would be the oncologist there, and whether, if not in person, whether that person, he or she can be part of the team virtually, whether through email or whatever means of contact, in case there's an answer we require just to follow up with the patient.
M1	So, I hear that in the public sector, there is adequate support. So, what about in the private sector?
D	D. We CAN refer to psychologist, physiotherapist, then the main concern would be cost, and where do they (go), how many places would they need to go to get it. I guess, in polyclinics, IF we are lucky enough, we'll have a psychologist and physiotherapist IN THE SAME PLACE. It's like going back to the same place, otherwise we have to send them to other places when they come to see me, then maybe to see the oncologist and he's like going to four places, even though it's supposedly (that) one person coordinates care. Cost will be an issue as well. There are pro bono counsellors or psychologists; I don't think there are pro bono physio(therapists). So, if the cost keeps escalating, then they'll probably go back to government sector again *[laughs lightly]*. That's (my) experience.
M1	Thank you. Okay, I think maybe we'll go on to the last few topics, which are quite related. So, as a primary care physician looking after YOUR patient who happens to be a survivor, what do you think will be the motivation for you to want to continue care for this patient? And also, I think (for the) stakeholders, I think we have roughly

	talked about. And then, maybe, if there's… other resources that you are aware of for cancer survivors, and what tools do you think, or what other resources do you think we should provide for patients? *[pause; 42:55 – 43:05min]*
A	A here. So, perhaps, (about) motivations to participate, I think to me, probably the biggest motivation would (lie with) the patient him(self) or herself, and if this is a patient that I know and I've been following up with, I will continue with follow-up. I think the motivation would be to be able to PROVIDE that holistic care to that patient without having to trouble the patient to run back to the hospital. For patient(s) whom I have not quite developed that kind of relationship with, and who's not quite empanelled in my team, you know, that hasn't QUITE had that kind of relationship (with me), then I suppose the motivation is a lot less. So, I think, then it brings up the question of the systems issue, of whether we can truly achieve that "one patient, one family physician" or "one team" kind of model. I think, to the system, I mean, of course helping out my colleagues in the hospital setting would be sort of a small motivation there. I understand that, I think, all the specialists are also getting a little bit crowded, and with chronic diseases, it can only get more and more (crowded), so I think it's also our duty to our colleagues in offsetting some of the load for them. So, I suppose the money will be a small motivation as well, and whether it's from the public or the private setting, I think there can be some kind of adequate renumeration. I mean, obviously, from the private setting, I think (it would be) in terms of the actual money (and) for the public setting, in terms of the recognition of (service to) that particular patient, a more complex patient, that requires a bit more (care). So, now we are trying to look at our own workload in the public setting, and (for) a patient like that, that requires so much care, probably it cannot be seen in the same light, say, like a URTI (Upper Respiratory Tract Infection) patient. So, whatever kind of weightage we are giving for such kind of patients, it must be recognized. And I think Ministry is also talking about paying for performance for certain conditions and all that, (so) if cancer is going to be such a big issue that we need help looking after them, then I think there's must be some recognition from that point.
M1	Thank you, A. So, if there is additional funding for that, will it change our practice? *[pause; 45:05 – 45:12min]*
A	A. I think a little? Maybe more so for D? I don't know *[M1 laughs]*. I believe he can answer a bit more *[someone laughs]*, you know, in public sector, the initial funding doesn't always translate into hits, but I suppose it'll help, better than nothing.
D	Err, D. Again, in terms of funding, I think it's only (that) it may apply to only one specific type of patients where we diagnose only cancer, and the oncologists say (they) can discharge, (they) can go to the GP (General Practitioner) just to continue (with) cancer surveillance and your usual health promotion. But what if it's for a patient that we're seeing for the chronic diseases, then it's very blurred. I mean, how do you decide how much to pay the GP (General Practitioner) to take care of the cancer-related problems? It's very blur. It's going to be very blur, because like I

	say, you don't have a specific appointment, you are just coming in for cancer, and you don't get remuneration for the cancer per se. Tough, if you ask me! *[laughs]* But I would think the best model would be one that the patient benefits (from), IF the remuneration can go to them getting subsidized medicine, getting subsidized physio(therapy), getting subsidized psycho(logical) (services), if they need, they can stay outside longer with the GP (General Practitioner), versus you are just paying the GP (General Practitioner) alone. You pay for a consult like that, the GP (General Practitioner) is happy, but the patient may not stay long, because if I need to refer him or her to some other places for allied health or some other medication that he needs that is cancer-related,, then the cost is going to up and he may not stay with the GP (General Practitioner) – that's for the private sector. This happens to the chronic (disease) patients as well. So, I think if you want to reimburse, reimburse the patient, (then) they can stay longer with GP (General Practitioner). I think it's good enough.
M1	Thank you, D. What if we create a system that allows the patient to still be a registered patient under the institution, that means, it's almost like a shared care, but the GP (General Practitioner) takes care all the time, in case if there's any referral, they can activate the referral in the institution and still be considered as a subsidized patient? Will that be able to work?
D	Referral is easy. It's the medications and what other things they will need when they are discharged from the hospital, so, like I mentioned, allied health, psychologist, the medications part, can they still get subsidized… for allied healthcare? If they can do so, then I think the motivations (are there) for the patients to continue going back to the GPs (General Practitioners) *[laughs]*. I mean, (it) depends on the GP (General Practitioners), (whether) they want to see back the patient again. It may still be higher than just paying the GP (General Practitioner) more money just to see patients.
A	A here. I think that it's probably a bigger system issue that I think D has highlighted as well. I mean, for a pure cancer patient with one diagnosis, if, let's say, he were able to tap on CHAS (Community Health Assist Scheme) for that, I mean, that budget to me, may be or may not be enough. So, if the patient has cancer, diabetes, hypertension, hyperlipidemia and all that, the same CHAS is going to cover all these, then obviously it's not going to be enough. So, maybe there's a system that we also need to look at, rather than giving the same kind of budget to all chronic patients. The budget needs to be adjusted to the number of diagnosis that they have.
M1	So, what if cancer is considered as a chronic disease and is added to one of the CHAS (Community Health Assist Scheme) condition?
A	So again – I'm A – if it's just cancer, it MAY BE enough, but if cancer is added on to your DHLs (Diabetes, Hypertension, dysLipidemia), and he has COPD (Chronic Obstructive Pulmonary Disease) as well, it's definitely not going to be enough, so…

	it may need to be a certain budget PER diagnosis, rather than the current budget we are familiar with.
M1	Any other thoughts?
D	D. *[laughs]* I think at the end of the day, I think it's the vision (why) you want to do shared care. Is it because the oncologist wants to so-called decant patients out to the GPs (General Practitioners)? If that's the case, there may be a bit of issue regarding, (because) first of all, it's the (medical) home - where the patient likes to go to. After that, all the related issues, we can actually solve by policies or solutions - money, medicine or allied health. It's more of whether the patients want to *[trails off].* That's my biggest barrier, because some of them actually come out from Cardio(logist), Psychologist, Psychiatrist, and they may not stay long because they don't feel the sense of belonging to the physician that they are discharged to.
E	There's one other group of stakeholders. E here. There's one other group of stakeholders that maybe we don't talk enough about and they are the caregivers, and how do we *[trails off]*. If it's just one primary care provider, it may be easier to arrange, if it's near the home of the patient, but if more time, and especially, let's say, a colleague in private practice, primary provider has referred the patient to another location for allied health support, then who brings this patient? Not ALL patients, survivors need to be trailed, but many of them may be. So, how do we then factor in the concerns of the caregivers in this respect? And then, maybe there has to be more dialogue with such caregivers. Primarily, a lot of time, it's the issue of time if they are working, and then, (the) transport costs and all these kinds of things, or even opportunity costs. So, it's something we should look into as well. But I also think that it is also good for selected cancers to be reviewed as chronic conditions, right? (It's) not a death sentence anymore, and that would help, … then resource-planning would be seen in a very different light, right? So, just like you say, HIV (Human Immunodefiency Virus) used to be a death sentence (and) it no longer is, then how do we then view these patients and the resources needed for all these patients? So, I think that would be a move to the right direction. So, that's perhaps one other linkage that we can make it all work.
B	B here. So, (I just have) some personal thoughts here and pertaining directly to MY motivation to participate in this care is that – I don't know but for most of us is that – if we have already established the rapport with patients and maybe we are the ones who refer the patients after diagnosis, I think it will be quite, maybe, fruitful for us to see back these patients after they have been treated by the oncologist. In a way, we are not expecting them to be grateful to us, but at least we know that there is THAT level of understanding and support and maybe trust that when these patients come back to us, if let's say we are manging their chronic disease, (we) may have a bit of advantage in swaying them to improve their disease, because we've helped them to tide through a critical period of their lives. So, I don't know whether you want to have that conversation with the GPs (General Practitioners) who have

	actually helped to screen and actually caught these patients at the beginning, and then refer, and these patients are treated well, and then, go back to them. And then, of course, it would be useful to me if there is that continuous conversation with the oncologists themselves who (have) been in-charge of the patient itself, and (for) any issues along the way, I know that, at a phone call, I can catch the oncologist to have a conversation, a talk or even anybody. I don't know. Some specialties have their Advance Practitioner Nurse or whoever. So, I mean, all these resources from the hospitals WILL be useful to the primary care practitioner, knowing that WE also have the support when these patients need it. So, I think this's just my sense.
M1	So, actually in the tertiary centre, they do build up a multid(isciplinary) team survivorship care with the APN (Advanced Practitioner Nurse) ultimately to lead the team. Currently, we don't have it yet, but if we do have this communication channel, would it boost the confidence of primary care physicians that you know you have a number to call if you are not sure when to refer back? Would that be useful?
B	I think, certainly, because we know that it's going to be within the working hours and stuff like that. Yup.
M1	So, maybe as the last bit, maybe (from) each one of you, can we have (some thoughts on) what do you think about these patient(s) having primary care physician(s) looking after the survivors? So, if you do have family member(s) or friend(s) who are cancer survivors, do you think they'll really benefit being looked after by primary care? What is the ultimate long-term vision? Of course, you know, it's not going to work in the meantime, not in the next few years, but would it be a good direction to pursue, or shall we just stop it and let the patients see multiple doctors since they are used to it *[laughs]* and go wherever it's more convenient, it's cheaper? *[laughs]* What should be our vision as a senior family physician?
A	A here. So, I think a good vision is that, I suppose I agree with, perhaps your first slide, where you talked about, perhaps, some, if not most, family physicians probably should go towards that direction, but I don't think we're going to reach a stage where everyone is going to be followed up by family physicians for all cancer survivors, because we also have different kinds of patients as well, and some patients who are already in control and empowered to look after themselves, they are really comfortable about seeing different specialists for their own different conditions, then they are able to have a good grasp of it and they don't mind, so I think we have these different groups of patients definitely. Some patients will be very happy with family physicians. I think they probably trust the family physicians more so than anyone else, and I think this kind of patients will be happy to discharge to the FP (family physician) for follow-up. So, again I think having a bit of a tailored or individualized approach for these groups of patients would then be suitable. And then, we shouldn't go to the direction of saying, "Discharging everyone from

	oncology care to primary care.", because I think some of the patients will not want it.
M1	So, maybe, do I understand that maybe it should be like an option, we should provide many different options for them and let the patients navigate care themselves, and probably guide them along?
A	For those who can, perhaps, but of course the navigation depends on whether the patients are empowered or are ABLE to do that.
M1	Anyone? Maybe each one take(s) a turn?
C	C here. This is definitely the direction, because this is in alignment with family medicine - providing a continuity of care, providing holistic care. I guess one of the things to consider would be where the patient or the doctor wants the care to be sited. Can it be a family physician working in the hospital providing this care, instead of a family physician in the community? I guess I agree with A that ultimately, it's the patient's choice of where they want their care to be. Yah, that's my (two) cents worth.
D	This is D. I think for this to work successfully, we will really, really need the whole system to be primary-care-based first. If patients have their own medical home, like family physician, once you do this, it's very logical that they will say, "Okay, yah, if you want me to go back to FP (family physician), I will go back to FP (family physician).". And the other thing is (about) the diagnosis itself. Interestingly, when we refer to Ortho(pedics) for, like, back pain, some of them come back to me and say, "Aiyah, see them also no use, because they do the same thing.", but never, never, (have) I (seen) an oncologist patient tell me, "I don't think I want to go back to the oncologists because they do the same thing every time.". They want to go back to the oncologist, because it's a very drastic diagnosis, because they fear the recurrence, so again, it's diagnosis-based. This may be tougher, versus back pain *[laughs]* that they go back because (it's) the same thing *[laughs]*. They actually come back to the FP (Family Physician), because it's cheaper and they are more familiar with us. So, I think for onco(logy) patients, especially if there's colorectal (cancer), there (are) so many diagnoses, it's going to be a challenge to try and get the whole system to buy in and have FPs (family physicians) to start seeing onco(logy) patients, because it's quite a drastic diagnosis.
M1	E and B? Any last thoughts?
E	I would echo *[trails off]*. E here. I would echo what's been already said, and I think it's in the ethos of family medicine to give as much shared care (as possible). And currently, while we say, ideally, it should be in the hands of patients to decide, but sometimes it's not possible because of cost, because of expertise, because of the way the primary care OR the tertiary care is set up at this moment. And a lot also has to do with some of these (issues) that we have to deal with in the patient(s). So, yah, maybe it's an ultimate goal, and we need to take some steps otherwise we'll

	never get there, but I think even just talking about it or thinking about it is a good first step.
M1	Thank you. Last words from B?
B	Err, I don't know what's the vision, but I think right now, I think our climate might be moving from healthcare to health. So, generally, if I'm practising as a *[trails off]*. I mean, I AM practising as a GP (General Practitioner), so I would feel more comfortable in that my patient is actually STILL on follow-up with the specialist, whoever they see, because at least I know that the particular critical part is at least being covered, UNLESS it's really at that point in time whereby it's very sure that (for) this patient, (there's) really nothing much that can go to this specialist (for), really they are doing just counselling, nothing really very much, and the patient has been informed by the specialist that they are actually very stable to come back to us, then I think I'm okay to actually see them on a long-term basis. BUT that's really for cancer survivors who are already past a certain stage. So, (it's) a bit difficult to talk about the vision at this point in time, but more of what is safe for the patient. So, we CAN have all the training and learn how to screen, but because we're in general practice, we have to see A LOT OF other things. So, some of the things, if the patient(s) don't mention, we may just forget. So, it would be good to really have a work group, PERHAPS, of the oncologists and family physicians to really sit down and talk about how each practice is like, patients that they see, the load that they see (et cetera) and really work and see how to streamline it and make it intuitive for everybody; maybe run through, like, a patient journey, if a patient comes and see the family physician, what happens at certain (milestones), and see whether it really works before (implementing). Then, … I think that might be useful, before we roll out all these. That's my thoughts.
M1	Do you have any other comments, thoughts? Okay, if not, thank you very much for giving all your perspectives today. We'll stop the recording. *[B replies, "Thank you."]*
	[Audio recording ends at 1:01:11min]
