# Supplementary material for: Implementing a community-based shared care breast cancer survivorship model in Singapore: a qualitative study among primary care practitioners
Source: BMC Prim Care. 2022 Apr 8;23:73. doi: 10.1186/s12875-022-01673-3 (PMC8991467; doi:10.1186/s12875-022-01673-3)
Supplement: Supplementary file 3 — Additional file 3. A compressed folder containing the raw data transcripts and demographics data collection form. [file 12875_2022_1673_MOESM3_ESM.zip › Supplementary Information File 3/FGD (08.31.2018).pdf]

Transcript for Focus Group Interview Junior 31st August 2018

Key:

Moderator / Interviewer:	M1
Respondent:	Participant A (A)
	Participant B (B)
	Participant C (C)
	Participant D (D)
( ):	Paraphrases, additions to or rectification of grammar, vocabulary and/or truncated sentences.
[ ]:	Non-verbal, e.g. *[xx laughs] [pause]*
…:	Removal of false starts, repetitive or ungrammatical long phrases
CAPITAL LETTER:	When there is a louder emphasis or stressing on a particular word or phrase

M1	Good afternoon. Thank you for coming to our focus group today. We have six themes here, so we will start with the first theme. Can you please introduce yourself, like, "I'm a family physician working in which area.". And also, what is your experience with breast cancer survivors, whether you are seeing them and in which context? Can I invite A?
A	Hi, I'm one of the doctor(s) working in (a polyclinic) *[omitted for reasons of confidentiality]*. Regarding my experience with cancer survivors, we do see a few – not a lot of numbers of cancer survivors in our (polyclinic) *[omitted for reasons of confidentiality]* in my short duration of practice here. We don't see that much (on) a daily basis. Sometimes, if the diagnosis was not written in the long-term care concern, and if the patient actually didn't actually address (it), we might not even know that the patient actually had cancer before.
M1	Thank you. How about B?
B	So, you mean, how frequent do we see cancer patients? *[M1 clarifies, "Yes, that's right."]* I think (it's) the same thing (for me), if … let's say a previous colleague put (this information) in the history, then we can tell, or if the patient volunteers, saying that he was previously on chemo(therapy) and if he is still on chemo(therapy). But it can be for other non-relevant issues and he doesn't volunteer, and sometimes there's just a discharge summary or other cancer centre notes, then sometimes we may not be able to know, or it's a bit difficult for us to know also.
M1	How about C?
C	I'm a doctor working at (a polyclinic) *[omitted for reasons of confidentiality]*. I think so far, I've been here a few years, and we do see a fair share of cancer survivors. Most are pretty well, but some may have background of chronic conditions. Usually, I will check through their past history and look through their prescription list,

	because from their chemotherapy drugs, then we may be able to have a sense that they are breast cancer survivors.
M1	Thank you. Let me go on to the second theme. So, we want to discuss the perceived barriers of the proposed shared care model. Would you like to share what are the barriers? We can divide them into patient-related, physician-related and healthcare-related issues. I think for *[trails off]*. Yes, A?
A	Yes, A here. So, I think for patient-related (issues), the barriers that, I think, from the patient's (perspective), is that sometimes because they, especially from patients who have been receiving ACTIVE treatment, some of them… feel that when they do experience some symptoms, they would rather present themselves directly to the Emergency (Department) or the National Cancer Centre, because a lot of the time, they think, based on their experience, especially when they started to have fever, (they) know that sometimes their white cell count will be a bit low, then they might need admissions. So, sometimes they might just present themselves DIRECTLY either to the A&E (Accident & Emergency Department) or National Cancer Centre.
M1	So, in that case, do I understand that they are less likely to come to the primary care?
A	I would think so, because even when… back in my training time when we're running Emergency (Department) and also were running other surgery clinic(s), we always advise the patients and alert (them that) if they are on active chemotherapy or any treatment, (and) if they started to develop any symptoms (and feel) unwell, then usually our advice is to see doctor or either come to the Emergency (Department). But my experience in the Emergency (Department) is that, a lot of the times, they just came directly.
M1	Okay, thank you.
B	I think (one of) the barriers is that *[M1 interjects, "B."]* some of the chemo(therapy) medications that the patient is on - or other drugs - sometimes the side effects (are what) we are not so familiar with. So, when patients presented with possible side effects of medications, we may not be so committed to diagnose it. Maybe a bit more communication -
M1	*[Crosstalks]* – so, B, do I understand that you are saying that one of the perceived barriers is the confidence level of the primary care physician? *[B replies, "Yah, yah."]* Okay. Thank you. How about C? Any barriers that you can share?
C	So, I can possibly see that the patients might be more willing to go back to their oncologist in-charge of their condition, definitely. That would be most likely the case. And if (it is) regarding barriers that are physician-related, I would think if we are not that familiar with the types of chemotherapy drugs, then there would be

	some worries, like, what if this patient is on this medicine, what should be the level of investigations and treatments that I should do.
M1	Thank you, C. So, perhaps, I will also explore what are proposed solutions. So, do you think that *[trails off]*. How do we increase this confidence among the primary care doctors?
C	So, therefore, probably there should be a programme where some training can be provided for a select group of family physicians, where they can then gain confidence and knowledge in the relevant chemotherapy drugs or treatments that are provided to the cancer patients.
M1	Okay, thank you. How about A? Any proposed solutions to overcome the barriers?
A	I think I agree with C. I think getting us some training courses to identify some of the common cancer diseases that we will see in polyclinics, that will step into polyclinic, and also, what are the usual side effects, what are some of the drug interactions that they should avoid for certain chemotherapy drugs, (and) some of the basic training and also the red flags (as to) when can we actually monitor a bit further in polyclinic, and when do we truly have to refer back to cancer centre or A&E (Accident & Emergency) for… admissions.
M1	Thank you. How about B? If you're saying that it's good to have training and education, (and) we understand from previous groups that they will say that there is time limitation of about seven minutes per patient in the polyclinic, (so) would this extra training and information be able to help you manage the patient more holistically, or you think it's unrealistic or it is really difficult to manage in the polyclinic setting?
B	I think it's beneficial, because at least we know better of the medications that the patient is on, and if they developed, presented with certain side effects, we'll know how to deal with them (and) we know better when to refer back or can keep in the polyclinic. *[M1 clarifies, "So, it's good to know?"]* Yah, yah.
M1	Okay. Thank you. Okay, let's us discuss the next theme on the survivorship care plan. So, if you look at the care plan over here, this is like equivalent to a discharge summary, which we hope the cancer centre can give … to every cancer survivor, then, if they bring it to you *[trails off].* Maybe we can have a look at this together. There are two main components – one is the treatment summary, what the patient has gone through, then the second part is follow-up plan. So, maybe you can give us some ideas whether this information is useful or too detailed, or which are the areas that you would like to have more information? So, let's go through the treatment summary. So, do you think that the details here about, you know, whether it is being estrogen-positive, the staging, the surgery or the radiation (et cetera), is this information useful? Is it necessary?

A	*[pause; 8:18 – 8:30min]* I think it would be great – *[M1 interjects, "A."]*. Sorry, A here. I think it would be good if *[trails off]*. I mean, it's good to include the diagnosis year and when is the surgery date, but it is also good, to say, (in) the "systemic therapy" column, whether it is still ongoing or it is past treatment. So, if there's still ongoing… there is an indication, at least we know that the patient is currently still receiving treatment, or whether the patient has already completed treatment for how long. Then, the other (point) would be *[trails off]*. Yah, I think for now, that's it.
M1	Okay, A, would I be correct to say that if the treatment is like many years ago and is no longer giving the problem, this information won't really be useful for you and is this what it details -
A	*[Crosstalks]* – err, I think it is good to say that the patient is previously on Tamoxifen, for example, "Breast cancer, on Tamoxifen, stopped at which year", then we know that at least the patient has been on Tamoxifen for how many years. It will be useful for us to see the stability of the disease, I mean, like how long has the patient been disease-free, and also (for) the subsequent complications, at least we know what's the risk factors.
M1	Thank you. How about other doctors?
B	Erhm *[M1 interjects, "B."],* yah, so I think maybe like the history of surgery and chemotherapy, that (information) is really important, but maybe the (information on) the receptors, (whether the cancer is) estrogen-positive, progesterone-positive, so on and so forth, I think maybe (that's) not so relevant. *[M1 clarifies, "Not so useful, is it?"]* Yah. The stage, I think "plus minus" (useful).
M1	You can also comment on the follow-up care plan about whether this information is useful to you.
Unidentified female	Errr I go toilet first. Cannot concentrate.
M1	Okay *[laughs].* C? Thank you! *[laughs lightly]*
C	I think I like the part where you actually include some side effects of the chemotherapy drugs *[B agrees, "Yah, yah!"]*, because that will actually highlight to us some things to look out for *[M1 interjects, "Okay, sure."]*, so that we will take note of the restriction (on) that. And regarding the familial cancer risk assessment, I think that provides us with the avenue to further suggest to the patient to get their relatives to be screened, so I think that is actually quite beneficial.
M1	Thank you, C. That is a very good point. Am I correct to say that you often see the family members as well in the primary care setting? Do they come with their family members?

C	Yah! Yah! They do.
M1	Okay. So, it would be useful to know more about the family history as well?
C	Yah.
M1	Okay. And how about the follow-up care plan? B, would you like to -
B	*[Crosstalks]* – I would say that I do like this cancer surveillance recommended test, so that we know (we) are sure that this patient needs (an) annual PAP Smear or annual mammogram or whatever.
M1	Okay. So, the guidelines in the care plan is useful?
B	Yup.
M1	*[pause; 11:53 – 11:58min]* What about in terms of the last section (on) the details, you know, like, "psychosocial issues", because we think that psychosocial area is actually the part that primary care physicians can play a big role. So, you know, if they state that they have anxiety and depression, or fatigue, or have problem(s) in their school, in their work, would it be useful to you, and would you be confident to be able to manage their concerns?
A	A here. I think, in this column, because this care plan will be prescribed to the polyclinic setting for us to have a look, to review on, so if they already have screened through some of the possible symptoms… to be highlighted, and for us to take action to it, then it will be easier. For example, some of the psycholog(ical) symptoms like, for example, anxiety and depression, … sometimes with the short consults, if the patients actually don't voice up, we may not be able to pick up as well. But if it's from the National *[trails off]*. I mean, if it's from the survivor care plan (and) it has already been screened and noted that there might be signs of these (symptoms), then at least we would take note and highlight, and we might do an appropriate assessment and also an appropriate referral.
B	I think - *[M1 interjects, "B."]*. Yah, B here. So, I think maybe regarding some of the specific issues such as like sexual functioning or fertility issues that are (present) after chemotherapy or radiotherapy, I think maybe we would need more information on that, because sometimes the management can be a bit different from the general populations.
M1	Okay, thank you. Actually, B, you bring up a very good point about sexual functioning, because this is an area in which even the oncologists themselves try not to ask, because they may not have the necessary training or the confidence, so they say that maybe the primary care are better at it? So, what do you think? C?
C	I would think that in the primary care setting, we might not be that confident in dealing with this issue of sexual functioning or fertility, but there can be an avenue that can probably guide the patients maybe to have a further consult with our O&G

	(Obstetrics & Gynaecology) colleagues. Maybe that might be something that can be that and they will be more confident in dealing with the areas of anxiety or depression. Yah, that should be something we can look at.
M1	Okay, so we are looking at the survivorship care plan. So, we'll talk about the treatment summary, which are the areas which are useful, and also about the follow-up care plan, especially talking about this area about whether… this information would be useful for primary care physicians to have when the breast cancer patients come to you. So, will things… like "insurance" or "sunscreen use", or "memory" or "concentration loss", if the patient comes to you with these concerns, do you think the primary care will have the confidence to manage? D?
D	I guess, insurance ah?
M1	Is there something that primary care deals with about insurance or about jobs? Because now, as the patients live longer, probably they want to go back to jobs. There would also be resources that you want to access to help them to look into this area about returning to work?
A	I think a lot of – I'm A here – so, I think a lot of the time, we can basically only ask very brief questions about the occupation part, and then if there is any assistance needed, for example, financial or occupational, usually we will refer to Medical Social Worker, and then *[trails off]*. Yah, usually we will refer to Medical Social Worker.
D	I mean, this is for patients who are *[M1 interjects, "D."]* This is only for those in remi(ssion)?
M1	That means, we are looking at these breast cancer survivors who actually have finished treatment, and maybe they are (in remission) five years or ten years after the cancer treatment, and actually they are cancer-free and they are well, but they still need surveillance. And then, they tend to have a bit more other concerns, like psychosocial, long-term side effects of the treatment. So, if they come to you with this, like, you know, any patients come to you with a discharge summary, do you think the information is useful? Which are the ones that are not very relevant to primary care and can be taken out, or are there some information we have not included? Because this is based on the American Society of (Clinical) Oncology.
D	I think there shouldn't really be a lot of issues *[M1 probes, "With?"]* with, I mean, like helping them in terms of *[trails off]*. Actually, they don't really have a lot of side effects in their treatment and after that. And if they came to work, they are normal also, right? *[M1 replies, "Yes."]* Then, if they have any issues arising from, I mean, if it's like post-RT (post-radiotherapy) and there are stark changes, usually those would come up much later. Then, only then, it has become (such that) any issues can be referred back, perhaps?

M1	Okay, thank you D. Would you like to share with us that in the polyclinic you are working at, have you experienced many cancer patients, and usually, which stage of their treatment are they in, or they are cancer-free or finished treatment long ago?
D	A few, I guess. *[M1 probes, "So, there are some?"]* Yah, some. There are some cancer patients that come to the polyclinic, not many. *[M1 probes, "What do they come for?"]* I think there were a few like post "Stage 1 or (Stage 2) cancer", but very early stage and already post-op(eration). So, she just came for other issues like knee pain or that sort of things. *[M1 clarifies, "Oh I see. So, they come for unrelated issues to their cancer condition?"]* Yah, unrelated issues. *[M1 replies, "Okay."]* The ones who are actively having cancer, it could be (that they are here for) symptoms. If the symptoms are not very suspicious and they may be related to other treatment they have, ongoing, then we refer back.
M1	Ok, sure, thank you. Let us go on to the fourth theme: what are the motivations for participation of this shared care model? So, here, we are asking, as primary care physician(s), should they be involved in the shared care of these cancer patient(s), or should that area be left to the oncologist alone? Any thoughts? *[pause; 19:48 - 19:57min]* Is it too specialized or is it beneficial for the patient, because we are still seeing the same patient, but here in Singapore, the patients here usually see multiple doctors? Am I correct to say that? *[laughs]* B is nodding in agreement. Okay! *[laughs]* And are they used to seeing multiple doctors? Are they quite happy with it?
B	I mean, if the problem gets solved, then yah, they're happy. *[laughs; everyone laughs too]*.
M1	Okay, C, what do you think? I mean, this is what we call "fragmentation of care", right? So, but is it really fragmentation?
C	I would think that actually patients do prefer to see the same doctors, but I guess since they have several medical conditions at the same time, the best we can do for them is probably (to have) the same family physician that they are seeing each time, and then, their oncologist. So, probably the shared care model would prove to be quite useful, because maybe the family physicians will be definitely better in managing their chronic conditions and (will) be able to take care of their psychosocial aspects as well. So, I think that will be a plus, an additional benefit to the patients.
M1	Thank you. So, A, what do you think? Ultimately, is it Singapore's vision about "one patient, one family physician, one family doctor", and the family doctor looks after everything? Do you think it is something which we should aim for, and is it realistic for cancer patients?
A	I think it is still possible, when the cancer patients DON'T just present with cancer disease (and) they present with other diseases, other comorbidities. And, I mean,

	with very good support to the family physicians regarding *[trails off]*. I mean, like for example, some of the training helps us know what are the things that we need to look out for (in) the primary care setting, and when can we refer, what are the things that we can actually handle, say, in a primary care setting, with the supports and also the knowledge and training. I think it will help us to gain more confidence in participating in this shared care, especially in those cancer patients in outpatient setting.
M1	So, do I get the idea that, like, family physicians actually WANT to participate in the holistic care for cancer patients, (and) if they are free of cancer and they have finished treatment, you are quite happy to be able to look after the other aspects of the patients?
A	Yah. Yup.
D	*[Crosstalks]* – but I think the family physician *[M1 interjects, "D."]* need to be educated on the kind of complications that can arise from previous treatment with chemo(therapy) or RT (radiotherapy). I think as long as the family physician is highly skilled in picking up *[trails off]*. Because some of these complications can occur years later after the treatment, I think picking up things like recurrence of cancer is not that difficult. *[M1 interjects, "Yah, most of them would have the training."]* And then, (it's about) having the facilities, like, having access to ultrasound and what-not. So, I remember when I was a student and I was in the clinic before, there was a patient who had RT (radiotherapy) for breast cancer and she was on regular follow-up and then there was recurrence later. There was recurrence (shown) in the ultrasound. If we have access to these (facilities), of course this will help as well -
M1	*[Crosstalks]* – so, the access to the resources is important as well?
D	Yah, then we know when to go back. I think it's better.
M1	Okay, thank you D. So, perhaps, the last two topics are actually quite similar. So, we're asking that, what are the *[trails off]*. Who do you think should be the stakeholders in this shared care? So, we have the oncologists, the family physicians. Do you think that there will be other people who would be important in the care of the cancer survivors? And in your experience, do you know of community resources for the cancer patient(s)?
C	I'm C. So, I'm not sure if it's beneficial if the social worker or psychologist be included in the shared care model as well, because I think – *[M1 interjects, "So, it should be a multidisciplinary team?"]* Yah, a multidisciplinary team, or even a dietician if needed. And a lot of breast cancer survivors… have lymphedema issues, so some of them CAN still have that when they present to us in the polyclinic. So, do we have access to the therapists that can help them with lymphedema.

M1	I see A is nodding. So, if you have a multidisciplinary team, would you be more confident to look after the cancer survivor(s)?
A	I would say, not only… to us, but more confident in taking care of any patient. I think the patients will find it very easy in terms of finding solutions for their other symptoms as well. For example, if they have been seeing a doctor, and also at the same time, they have been seen by the dieticians looking after their diet, and the psychologist for their mood, (that means that) they don't have to see one doctor, and then, we refer to another, and then, from one, (patients are) referred to another (again). Then, you make them have multiple visits, and in the end, the patients will feel a little bit troublesome to have multiple visits as well.
M1	Thank you. So, C, you are nodding. So, is it frustrating to the patients, you know, from your encounter with the patients? Do they find it very difficult having to see multiple doctors and (go for) multiple visits?
B	Yah! C! *[M1 interjects, "C? B?"]* Oh sorry, B. So, I think it's (about) the expectations and the understanding of the system, because some of the problems (are) that we don't have the facilities or resources to solve, but if they EXPECT or if there is some misunderstanding in the communication, then they may be frustrated about it, (like when they) have to be referred back, and it's very troublesome and all that. But sometimes, I think maybe a coordinator would be *[trails off]*. A coordinator who is familiar *[trails off]*. I think the coordinator may be based on *[trails off]*. Err, so, I think (the coordinator can be) based (in) the cancer centre, but (is also) someone (who) is familiar with the polyclinic framework. That is better for the patient.
M1	So, D, what do you think of the stakeholders and the resources?
D	What other stakeholders (are) there? The patient, right? *[laughs; M1 replies and laughs, "Yes. The most important stakeholder is the patient, yes."]* So, patients, their families and the oncologists, I think the nurses? *[M1 replies, "The nurses? Yes."]* The onco(logical) nurse, the patients follow up with them. Community resources? Maybe Singapore Cancer Society does things in the community. *[M1 clarifies, "Are you aware of what they provide?'"]* Not very well. Not very well *[laughs]*.
M1	Okay, so they should raise their awareness, Singapore Cancer Society. Actually, they do have a rehabilitation centre in Jurong. Okay, so, as a conclusion, maybe we just invite each one of you to just have a short word: what do you think is the future of breast cancer survivors? Do you think it is a good thing to explore further training and shared care model, or you think that the care is good enough in the tertiary centre and it should be kept in the tertiary centre? Maybe we'll just go round (to share)? A?
A	I think shared care will be ideal for patients who, especially when they have multiple… conditions that the primary (care) physicians can actually handle. And I think it's a good idea for the shared care.

M1	Thank you. B?
B	I also agree with the shared care model, so that they can *[trails off]*. As in, finishing the treatment, then we can take care of the chronic parts. And with more information that we can have, then the better we can (provide) care.
M1	Thank you. C?
C	I think the shared care model is ideal and probably family physicians can take on more roles and support the patients in the journey after cancer treatment.
M1	Thank you. D?
D	I think it should (help) - the community, I mean. The tertiary side offloads a lot of case(s). *[laughs]*
M1	*[laughs]* Yah, (but) besides the offloading from tertiary care?
D	Family physicians are supposed to be more accessible in that sense. *[M1 interjects, "So, it's good for the patients?"]* Yes.
M1	Okay. Thank you very much for coming for this focus group. I'll stop the recording.
	[Audio recording stops at 30:12min]
