# Supplementary material for: Implementing a community-based shared care breast cancer survivorship model in Singapore: a qualitative study among primary care practitioners
Source: BMC Prim Care. 2022 Apr 8;23:73. doi: 10.1186/s12875-022-01673-3 (PMC8991467; doi:10.1186/s12875-022-01673-3)
Supplement: Supplementary file 3 — Additional file 3. A compressed folder containing the raw data transcripts and demographics data collection form. [file 12875_2022_1673_MOESM3_ESM.zip › Supplementary Information File 3/IDI (11.23.2018).pdf]

Transcript for IDI 23rd November 2018

Key:

Moderator / Interviewer:	M1
Respondent:	A
( ):	Paraphrases, additions to or rectification of grammar, vocabulary and/or truncated sentences.
[ ]:	Non-verbal, e.g. *[xx laughs] [pause]*
…:	Removal of false starts, repetitive or ungrammatical long phrases
CAPITAL LETTER:	When there is a louder emphasis or stressing on a particular word or phrase

M1	Hello, A, thank you for agreeing to come to our in-depth interview today. Can I ask, can I use this topic guide? Feel free to if you want to share from your perspective. Can you share, from your experience, what your experience with cancer survivors?
A	In the *[trails off]*. When I run clinic – I run the family physician clinic – I see mainly patients with chronic diseases, and a number of them are either currently receiving treatment or they have some cancer in the past, but now they are in remission. A number of them are actually still seeking some sort of treatment, but not many.
M1	Not many? Okay, thank you. So, what do you think is the reason why family physicians, like in the polyclinic setting, will be a bit hesitant to look after especially the cancer survivor(s), especially the cancer aspect, since you are already seeing them for your chronic disease?
A	Yah, I think it's always *[trails off]*. One of the reasons… in the polyclinic, especially for doctors running general clinic (is that) some of them are already struggling to handle the increasing number of tasks or consultation(s), (and) of course, attending to the patients' medical needs, administration needs, and now, we have an increased emphasis on preventive care, screening and vaccination. So, for them, it will be another area (in which) they are worried that they will have no time (and) they have to prioritize. So, sometimes that becomes something that is secondary in relating to their cancer symptoms. I think a lot of doctors also feel – myself included feel – that they are still seeing their specialists, especially those who are still on follow-up, they will kind of largely manage those areas. The other reason is, I guess, (the) lack of experience, in terms of having established guidelines to help them manage symptoms that the patients might present with.
M1	Okay, thank you. So, can you share, in the aspect of, like, cancer education, so like I understand, for yourself, you are involved in education for the family physician training, (so) how much of the time is really provided to train your family physicians in cancer care? Do you think it's something which is relevant, which is important, which we need to allocate a bit more time in teaching?

A	I think in this polyclinic institution, there are mainly two types of education, so one is our internal CMEs (Continuing Medical Education), our Continuing Medical Education, that time to time, we will get specialists in different fields to come and deliver talks and CMEs (Continuing Medical Education) on particular topics. So, once in a while, there will be a topic relating to cancer or palliative care, but I would say at the moment, those are really few and far between, because most of it is on chronic disease management, is (they are) on surgical conditions or things like that, so that is one thing. And we encourage the doctor to develop professionally in terms of taking things like post-grad diploma in family medicine, getting the Masters of Medicine and so forth, so that will be largely dependent on the curriculum of these two diplomas, and I think the GDFM (Graduate Diploma in Family Medicine) have a little section on palliative care, and likewise for MMed (Masters of Medicine), there (are) some areas of palliative care, but perhaps this area might need to be looked at to see whether there's any need to enhance, to have more (of). And I know that there's a diploma in palliative care, but I think in the polyclinic, because, largely again, because we don't see, or rather, we DON'T THINK that we see such patients, so I think in our polyclinic, very few of them don't even think about go(ing) to pursue a palliative diploma.
M1	So, from your experience seeing patients and managing polyclinic, do you foresee an increasing need to educate our doctors and to give them the experience to manage cancer survivors?
A	I think definitely, because cancer is on the rise, either detected early or they receive treatment, so we are going to have more and more patients who are going to be cancer survivors or they are in remission, and as the cancer rates go up, as treatment gets better, they would inevitably come into the system, at least in the polyclinic setting where they either have some chronic conditions, or they would come to us episodically for their acute care needs, so I think as family physicians, we always try to find out whether they have other continuing problems, so inevitably, we're going to find out that there might be a cancer patient or maybe (a patient) that is currently seeking some maintenance therapy, and I would say, as part of holistic care, we SHOULD consider these areas, like their cancer histories.
M1	Thank you. So, from our interviews with other polyclinic doctors, one of the things which they shared with us is that if they are worried that if they manage cancer survivors, they are worried (that) they don't have the competency and they would miss recurrences, and that is a great barrier for them. So, what do you think about this?
A	Yes, there is always this fear "Am I ignoring certain symptom presentation or what?", but I think like in all areas of all practice, be it seeing a patient with acute complaints or seeing chronic (complaints), you have to, (firstly), be up-to-date, then, (secondly), when you are seeing the patient, be as thorough as you can, and I think guidelines will help too. I think we don't have guidelines relating to what to look out for in, maybe, certain types of cancer, so I think guidelines will definitely

	help us, in addition to us taking a proactive approach to learn developments in certain area(s). So, right now, I think we don't have. I think in our doctor's guidebook -
M1	*[Crosstalks]* – I think it's only on cancer screening, right?
A	Yah, it's not on managing symptoms per sec, so we could put that in the guidelines, we could have some CMEs (Continuing Medical Education), I think that would be useful as a start.
M1	Okay. So, I see that it is a very positive aspect. Can you share with us, what are the shared care programmes… that the polyclinic has done before and whether they have done well, they have succeeded or they have failed, and what we can learn from them?
A	Shared care programmes?
M1	Yes.
A	Is it relating to cancer or other programmes?
M1	Other programmes. I understand you have mental health, you have dementia?
A	I mean, dementia and mental health (are) more at a primary care level (where) we created the mechanisms to actually do a more thorough screening … prior to referring onwards. That, technically, is not really a shared care. A shared care would be that maybe the patient has already been seen in the hospital, and then discharged back to us, so some of them would be things like the heart failure pilot (programme) that is going on. The barriers would be, I think, to still maintain that two-way traffic, that means, when the patients come back to us, we will still manage, but whenever issues arise, is there a point of contact back at the hospital level that we can quickly ask to decide whether we need to refer quickly back, or is there a fast track back, rather than (have) these people have their next appointment to the hospital one year later, and there is no way to then establish the link back. The other barrier that I think would be also (having a) common care plan, to exactly know what to do and to establish what is the care plan for the patients, but we know that care plans are not static (and) they change, so if it's changed from the primary care, then how would the specialists know? And if it's changed for the specialist, HOW would the primary care know? So, I think this flow of information enabled through IT (information technology) would be also very helpful for us, so maybe I would say that, if shared care (is to happen), once in a while, you (can) get a status (update) of what the specialist is doing for follow-up, and maybe the specialist can also check on how have the patients been in terms of what they are doing during our usual routine follow-up at the primary care in the polyclinic (then) I think that would be useful. Yah, that is what I think so.
M1	Thank you. So, I think the communication is very important – a point which is brought up a lot in the focus groups. So, have there *[trails off]*. From your

	experience, have you all tried ways of communicating on the electronic basis, whether through the Electronic Medical Record(s) (EMR) or through emails? Has that worked?
A	So, okay, I think at least in Singhealth, having the common IT (Information Technology) platform for Electronic Medical Records in the form of the SCM (Sunrise Clinical Manager) *[reference to electronic system for case records]*, it's helpful because at least in some of the visits, we can see what the specialists wrote, and now, (for) the memo, there is also an electronic version, so if the patient forgets to bring the memo, we can still see what's been written. Likewise, the specialists will be able to see the poly(clinic) consults, so that's one area. Across medication changes, we do have a little section in the Sunrise (Clinical) Manager that enables instructions, so, sometimes we know "Stop this. Start this.", (so) that is really helpful. So, for patients in the Singhealth institutions, maybe that is a little bit easier, but once they cross (over), if let's say this patient is seeing, let's say Tan Tock Seng (Hospital), through the NEHR (National Electronic Health Record), (we) will not be able to see what happened in your routine consultations by the specialists. But in terms of email, I think from time to time, we do email maybe directly to the specialists, to the point of contact, and they don't reply, but that's not exactly formalized in a systematic way at the moment, so it's more ad hoc.
M1	More ad hoc basis? Okay, so can I understand, just now you mentioned about a pilot study which you are having with the hospitals on heart failure. What are the push factors to start this programme and how do you think we'll maintain it and make it sustainable?
A	I don't know enough to actually answer that. It's a collaboration between Changi (General Hospital) and Pasir Ris Poly(clinic), but I guess it will be… along the lines of the flow of information both ways and (having) the access both ways. Other than that, they have guided protocols on what to look out for during our routine visits at the poly(clinic) and when to refer upwards, so I think that is useful. That is useful.
M1	So, when we actually shared (about) the survivor care plan, which we sort of adopted from the Americans, a lot of the family physicians actually tell us that although there's a lot of information, they are quite burdened by the length of the document. Do you see problems if this is something that we roll out as a means of communication?
A	Okay, family medicine has a problem, in that we grapple with many different specialties, so a shared care with Cardio(logy), a shared care with Gastro(logy), a shared care with Rheumato(logy), they will have their own nuances in terms of maybe with the discharge template, what is to be look(ed) out for, so in the end, we end up with many, many templates. In fact, we face problems when we want to refer to different specialties, and now they are kind of saying that (they are) mandating certain information, prior to be put in for referral(s), so we grapple with many, many different templates. So, we struggle on our end, especially if the

	patient ends up seeing the general clinic, the doctor only has an "x" amount of time, and if you come with a very lengthy referral letter or even a discharge letter, it takes some time to actually assimilate and see what (can be done). So, we suggest that maybe a standardized template might be useful with certain useful areas, and of course, when there are special areas to be looking out for. So, I mean, I'm looking at the form here and it's rather comprehensive, but just imagine if this patient has this form and then he's got another one, (like) maybe this patient also has renal impairment *[laughs]*, and then it becomes a problem. So, some information is like (the) demographics, it's kind of shared, so it's not a problem, but we have to then decide to prioritize what is the relevant information that is useful. Some of it can be kept by the patient, but some, especially relating to the care plan, then it should be something that we kind of share. But I think, like in every template, it tends to be comprehensive and we grapple with the "How do we then translate this template of three pages into maybe a few summarized sentence(s) about this, on what to look out for". So, imagine if a patient has four follow-up(s) with four specialists, they all have their plans for these four areas. When the patient comes back to the family physician, how is he going to then juggle this, and then fit everything in, keep track and being aware of all these, so there is a challenge. There is a challenge.
M1	How do you foresee that, such patients, if they have greater needs, should be cared for, at least in the family physician clinics? Or do you have any other specialized clinics whereby they can be seen?
A	Currently, we have the general clinic that sees everything that might not be the ideal place. Then, we have the family physician clinic, where… at least the appointment slots are fifteen minutes, so that will really give more time and that is helpful, although I would say that for some patients with multiple issues, sometimes even fifteen minutes is not enough, you know. Ideally, maybe for first visit, or handoff from the hospital, we should at least do thirty minutes to make sure everything gets properly coordinated right from the start, then it's easier backend. So, yes, in some areas, like we have the dementia and wellness clinic that is FOCUSED, but that is from our side, focused on sorting it out first and then deciding whether to refer or not. For these special clinics, we also would think in future as the patients get discharged from the back-down, is this a landing platform or should patient go then to a family physician clinic where everything is kind of stable already, so you just carry on? I very much doubt I would like to create a second-tier clinic JUST to cater to patients just to do palliative care. I don't know. I mean, it would then mean that you are fragmenting primary care already, so it's not ideal as well. RATHER, we should have (the) ability to risk-stratify patients. (For) patients who need more care, maybe we can spend more time. Patients don't need so many things, (then they get) maybe a shorter consultation time. Or maybe we can even get our nurse(s) or APN (Advanced Practitioner Nurse) to see some of these patients, but when patients need the full medical review, then we allocate the time accordingly, then that will be very good, because then we tier the resource according to the care needs of the patient.

M1	Can you share with us what do you mean by this "tiering"?
A	So, right now, we have an IT-enabled system to kind of band patients into bands, from the well-controlled with one or two chronic conditions, to the higher band where they have multiple conditions (and) they have poorly-controlled conditions like diabetes, or they may have condition(s) that affect their care, like dementia and depression, so roughly it's still work in progress, so we kind of cut the patients who are empanelled to our teams in(to) these four bands, so THAT will enable us to say, maybe in the future, these type of patients should be seen in what type of service, but of course at the moment, they all still potentially end up in the general clinic.
M1	*[laughs lightly]* I guess that's the biggest pool of patients?
A	Yah, because the general clinic just sees everyone, although we say we need to increasingly have a mechanism where we can then move some of these higher-needs patients to the family physician clinics. Or maybe even those with social plus medical elements to be under our case management, because we then address not just the MEDICAL problem, but if there are social issues compounding the medical care, we can then touch base with social agencies outside of the clinic to kind of sort those things out. So, we are looking at holistic care for the patients.
M1	Okay, so do you have empanelment?
A	We are starting on that, so our clinics have just started team-lets in our clinics, so patients will increasingly be empanelled to the team-lets. Hopefully, the patient ends up seeing that team, with time, to … enable the team to develop collective ownership of the patient, rather than (having) the patient being potentially moving around the doctors in the clinic.
M1	Yah, I think that's a very useful *[laughs lightly]* direction. Then, can I ask, if you tier a patient with greater needs, requiring more time, in terms of funding, is it sustainable?
A	Not with the current one, because the current funding, in a way, does not really recognise the theory. So, I think MOH (Ministry of Health) is also looking at how to risk-stratify the patients, to say that certain type of patients would need a certain type of care, therefore (to) be funded differently. There's some development, but at the moment, unfortunately, it is not the case. (For) patients seeing the family physician clinics, upfront they have to pay more, so it might already be –
M1	*[Crosstalks]* – but that's only a little bit more for Singaporeans, right?
A	Yah. On our side, it's like double. *[M1 clarifies, "Double?"]* So, to some, that is a financial gradient, but of course we will enlist the necessary financial assistance when they need, so that is one area to circumvent the present gradient.
M1	So, there was one suggestion by some of the doctors. They were saying that, I think for them, they prefer, like, because when you see more of the same type of

	patients, actually you think better and you are able to anticipate, like for example, if one day, you are in the acute clinic and you keep on seeing acute cases, you sort of know what they are, and if another day is (for) chronic (disease patients), then you see chronic, so how about (having) one clinic which is for *[laughs lightly]* cancer survivors, then for that day of the week you see the same, (and) in the end, would you end up with multiple appointments for the same patient *[laughs]*? That is the same problem as what the hospitals are having now.
A	Yah, so we need to control that and make sure that that kind of (arrangement) does not become the eventual model, because like I mention, (in) family medicine you deal with many systems, so if you have a special diabetes clinic, and then you have a renal clinic, and you have dementia clinic, then you have a cancer clinic, then this poor patient will end up (everywhere). So, our model has to be something that is workable. So, currently, I feel that in certain focus areas like maybe dementia and mental health, you could then pull that out as a focused clinic, but in ALL other areas, we have to resist this urge to create a special clinic, a focused clinic for every single area. It is still best to manage the patient as a whole, be it in (the) general clinic, which is not so good, or in (the) family physician clinic, which is a better setup for them. So, I would say that if we were to touch (on) this area of palliative care, maybe even just seeing just cancer patients, they should still be seen in our family physician clinic, where possible, to manage the patient as a whole.
M1	Can you share with us what is this special element of dementia and mental health that warrants a clinic on its own?
A	Well, I think because it's under funding by programme, so that's why we carve it out separately, but if you look at it, dementia IS going to become more common, mental health IS already common, although if you don't find it, you don't find it, right? So, technically, yes, patients with mental conditions and dementia SHOULD also be managed. So, what we have done for the mental wellness clinic is to create a higher tier where in certain conditions, you might need input by the specialists, to then create that higher level, but at the same time, we train our family physicians to run that higher-level clinic. In the future, they then bring it back down, then we (can) say that we don't need that second-tier clinic to just see these patients in the future, so that is the plan. Likewise, for dementia, it is this issue about whether we have access to neuroimaging, access to the use of dementia medication, which is (about) which level should it be (at). Should it be contained at the primary care level or should the patient need to be referred out and then come back? So, these are still work in progress, these models. But I would say, in the true essence of family medicine, the patient should be managed as a whole. As best as possible, you should manage the patient's comorbidities.
M1	Thank you. So, in terms of education and to train up the family physicians, like how you train them up in the mental health and dementia (clinics), what do you think is the best way to train them up?

A	Best way?
M1	Yah, because I understand that in terms of the Graduate Diploma and Master, they are really full – the programme is so full that they cannot fit in any other modules. *[A interjects, "Oh, I see!"]* So, I mean, in terms of practical ways, do you give them –
A	*[Crosstalks]* – I think, well, you could use more innovative ways. They could display some content *[laughs]*, or they could create maybe, like, I think now MOH (Ministry of Health) doesn't really do clinical practice guidelines anymore, but they do focus areas, in the form of "appropriate care guides" - they call (them) "ACGs". So, maybe an "appropriate care guideline" for maybe very common cancers or symptoms to look out for, that might be quite a useful blast to at least primary care. And maybe, through CMEs (Continuing Medical Education). So, in our institution, our internal CMEs (Continuing Medical Education) are "simul-cast", so just one specialist can be delivering a talk to one clinic but "simul-cast" to all the clinics, so that is really useful. So, we should just do more of some of these things and that would help. And then, lastly, in our doctors' guidebook, if there's an area on that, that would be useful. Yah, I think.
M1	Yah, that sounds good. So, can I ask, in terms of shared care, I mean, we know that in health promotion and disease prevention, primary care physicians are really the expert(s), so how about in the other aspects of cancer care, because patients nowadays are also on five to ten years of hormonal treatment, and with that, we do have a lot of problems – we have osteoporosis, we have lipids problems, premature menopause, (so) you think family physicians are able to manage that as well?
A	I don't think that should be a problem. Again, guidelines would help. Guidelines and Continuing Medical Education would help, just like I remember EARLIER ON, we were not very comfortable to manage patients who are on insulin in the earlier years, but with time, we are now fairly confident to manage most patients on insulin, and therefore, we hardly refer diabetic patients to the hospitals, because we are able to manage them using insulin. So, it's a matter of whether you have enough push to (go) towards a certain direction.
M1	I also agree with you because I feel that like last time, ten years ago, diabetic patients are mainly managed in the hospital, but now, you don't expect them to go to the hospital. So, how does this change in the mindset (occur)? Is it funding? Is it the availability of the insulin, education (et cetera)? What has changed?
A	I think it's multi-factor(ial). So, sometimes it's the extent of the problem that creates the need for the systems to adapt to contain it. So, for diabetes, I think it's because more and more people have diabetes and you can't afford to send everyone to the endocrinologist. So, there might come a time when there are many patients with cancers or post-cancers, then they also inadvertently have other conditions and they receive care, so you can't NOT look at that. You might need to look at that. So,

	it is a matter of the demand, and the system will have to react to it, And how you react to it? We can get people who are willing to share care with us, willing to show us and teach us, THEN with guidelines, with that support from maybe specialists colleagues, then we feel more confident to handle some of these things, and then, (have) a very structured-phase approach to then share the care down – not say, "down", but rather, to share the care with primary care – (and) define the areas that primary care can safely manage and those that SHOULD be continued to have a higher follow-up with the specialist. I think that will help, because (we shouldn't) open everything down, but if it's (done in a) controlled fashion, I think it would be useful, helpful.
	Yah, because I guess with improved screening, we are getting a lot of early cancers that are really (of) quite low risk, so maybe there's a big group of these patients that can be managed. And increasingly, the patients are also asking, "Do I really need to come back to the hospital?", for those who are educated, but at the same time, we are also wondering, "Is the primary care ready?", so I guess that's the main conflict.
A	If you ask me, the only things that I would need (are, firstly), a guide, (secondly), a plan for the patient, and (thirdly), a way that I can check with the specialist if I need to. I think with these three (things) kind of there, it will give (us) the confidence to manage these patients.
M1	That's very good. And how about the patient?
A	Well, the patients of course have to accept that - *[M1 laughs and interjects, "Yah, do you have the confidence or not? Because for so many years, they have been saying that."]* Yah, some patients still need to continue with specialist follow-up, because they are worried, especially if it's cancer, right? So, I think a lot of it would be also (on) the patient education, and the specialist must sing the (same tune) that, you know, "You will be seen at the Poly(clinic), but if anything were to require escalation, you can have an easy access, a ready access out.", (so) that is helpful. And I think with time, patients have also come to realise that the polyclinic doctors, or rather, primary care doctors are not just "cough and cold doctors". *[M1 laughs and interjects, "Yes!"]* They are doing much, much more. And so, that mindset will change.
M1	That's right, that's why many of them are saying that "Why don't I just go back to my primary care doctor? It's just a one-stop.", especially when they have been out of cancer for so many years, so I guess that is the group whereby we can really look on. And just to go into a further step, would the primary care be comfortable with prescribing, continuing Tamoxifen and aromatase inhibitors?
A	Okay, so it is, again, boiling down to the guide for these things, what to look out for and educating, teaching and … being supported. So, case in point: we are now looking towards our doctors being able to switch patients from Warfarin to NOAC (novel oral anticoagulant). So, in the past, it's like the territory of the cardiologists,

	but now, we have guidelines to delineate what is safe to do. So, for the patients on Warfarin, now if we follow some guide to say they don't have mechanical heart valve and they can change to NOAC (novel oral anticoagulant), then you don't have to refer to the cardiologist just to do this, so this is a step. And of course, this is also then endorsed by the specialist giving us their support and *[trails off]*. Yah, more of the support.
M1	So yah, I also hear this from other focus groups, which they shared with (us). And some of them are so burdened because NOW they have SO MANY patients who want to do shared care, or whom the specialists want, so how do you choose which patients and how do you support the family physicians? It is really almost like multiple specialists, right?
A	So, the field of family medicine is both challenging and exciting, depending on *[laughs]* how you see it! Because it's really going to get more complex.
M1	Yah, I mean, there's no end. You can do a lot -
A	*[Crosstalks]* - so, it will be what *[trails off]*. At the end of the day, it'll be prioritisation and seeing where is the big need, but of course if you say it's a big need (for) everybody, then we have an issue. I think the big areas of cardiovascular, cancer, ageing, (you) probably can't escape (from) it. You can't escape (from) it. Yet, on our other spectrum, we are also looking at enhanced child care, post-natal and things like that, so that's another big area *[laughs and M1 laughs too]*.
M1	So, it's really challenging to allocate the resources?
A	Yes.
M1	But how do you view having specialists to come down to polyclinics to run clinics?
A	They will be SOCs (Specialist Outpatient Clinics) minors?
M1	What do you think of that model? Will it work?
A	I think other places have tried this. Again, the specialists will bring a very silo mindset – they are just dealing with that thing; so you are just removing the need for the patient to go all the way to the hospital.
M1	Yah, you are bringing the SOC (Specialist Outpatient Clinics) to the community.
A	You might bring the person down, but you might not bring the SUITE of services down to support this thing, so it might not be totally workable for some of these things. For us, I think it might also be things like accessibility to certain investigations, which is currently not available to us, for example, doing CT scan. If that can be worked out with some protocol, then you don't have to be referred out just to do this. So again, a lot of these things is (about) how you work through the

	steps required to achieve the final outcome. I don't (see) brain specialists come down to the polyclinic. (At) our primary care level, we'll solve the problem.
M1	It's a different mindset, right, for specialists?
A	Yes.
M1	That's right, because from what I have gathered from the interviews, that family physicians are very special, in the sense that they value the patient and they are willing to have this holistic care, because in special(ty) care, a lot of them are just saying "This is not my area of expertise, and that's where I draw the line.". I find the change in mindset *[trails off]*. You know, can you share what is the difference between a special(ty) and a family physician, how we see the patient and how we deliver care?
A	Perhaps family medicine, by the sheer training, focus on the whole-person approach, dealing with not just the bio, but the psycho and the social elements to help. And as family physicians, we also try to *[trails off]*. One of the connoisseurs of continuing care (is that) we want to make sure that his care is well. Even if we kind of know he is still on active follow-up with the specialist, we just need to find out this fact, because we just want to make sure the patient's care coordination is intact. Specialist(s), because they are so focused in that area, the systems, they really also don't have the bandwidth to go and look at the other parts, although I know some specialists, they kind of –
M1	*[Crosstalks]* - yah, I guess there are also many good specialists who manage the whole aspect. Yah, that's true. And one of the areas which they feel family physicians value-add is the psychosocial aspect, so they would want to send patients with a lot of psychosocial concerns to the polyclinic(s), but I know that some of the junior polyclinics doctors are really appalled by the fact, because they say that they only have a psychologist (who) only come(s) once a month, *[A interjects, "We don't have a psychologist."]* and they don't have the time, the psychologist. So, how do we provide that psychosocial aspect?
A	You try to manage. If not, then the medical social worker IS supposed to handle some of these things, otherwise we have to refer out to community resources like some patients in our health wellness clinics are referred O'Joy (Care) Services for counselling.
M1	That's the *[inaudible; 35:19min]* programme. Oh okay.
A	But currently, we don't have enough psychologists to make use of as a service.
M1	So, is it difficult to get a psychologist in, or is it there's no demand or –?
A	I'm sure there is demand. It's (that) they are not funded at the moment. So, how are you going to -

M1	*[Crosstalks]* – okay, you can't employ them?
A	You can employ them, but you are employing them at own *[trails off]*. You are using your own funds and definitely, if you are charging and you are going to do cost recovery, that becomes a financial impact issue. We haven't really worked on this area yet.
M1	Yah, but it's just that you are seeing mental health patients, so we're just wondering how you manage -
A	*[Crosstalks]* - now the patients are referred out -
M1	*[Crosstalks]* – if they need psychologists?
A	Correct.
M1	So, I guess there are community resources that you can utilize -
A	*[Crosstalks]* – yah, we do tie-ups. We have two tie-ups: (for) Changi (General Hospital) that side, they have health wellness programmes; then from the central part, it's the VWO (Voluntary Welfare Organization), O'Joy (Care) Services, that we refer patients to.
M1	That is very exciting. Thank you very much for your sharing. Is there any other area you would like to share with us, whether this is something, you know, that is in the right direction?
A	I think palliative care would eventually form a greater part, or rather, have more prominence at least in the care of the polyclinic, so (I'm) looking forward to the developments that will come up.
M1	Thank you so much. Thank you, thank you.
A	I hope this is what you need? *[laughs]*
M1	*[laughs]* Yes! Yes! Definitely! *[laugh]*
	[Audio recording ends at 37:00min]
