# Supplementary material for: Implementing a community-based shared care breast cancer survivorship model in Singapore: a qualitative study among primary care practitioners
Source: BMC Prim Care. 2022 Apr 8;23:73. doi: 10.1186/s12875-022-01673-3 (PMC8991467; doi:10.1186/s12875-022-01673-3)
Supplement: Supplementary file 3 — Additional file 3. A compressed folder containing the raw data transcripts and demographics data collection form. [file 12875_2022_1673_MOESM3_ESM.zip › Supplementary Information File 3/IDI (09.24.2018).pdf]

Transcript for IDI 24th September 2018

Key:

Moderator / Interviewer:	M1
Respondent:	A
( ):	Paraphrases, additions to or rectification of grammar, vocabulary and/or truncated sentences.
[ ]:	Non-verbal, e.g. *[xx laughs] [pause]*
…:	Removal of false starts, repetitive or ungrammatical long phrases
CAPITAL LETTER:	When there is a louder emphasis or stressing on a particular word or phrase

M1	Hello, Doctor, thank you for accepting the invitation. Today, I'd like to invite your perspective regarding community cancer survivorship for breast cancer survivors who are low-risk, who actually have completed their treatment and are well now, (and) currently they are still being looked after by the institution, but many of them have gone on to long(term) survival, like five years, ten years, fifteen years. So, we are thinking that this group is suitable for community care, because of the holistic nature of primary care. Can you invite you (to share) on the first theme, which is, "What is your experience seeing cancer survivors in the polyclinic setting?" Yes?
A	(We play a) role in the primary care, because there is frequent contact with the patient, either for the episodic reason(s) or for the care of chronic conditions. So, if a patient has a history of cancer, it's quite common. (However), the thing is whether they bring the issue related to cancer to us or not. It is much less common. This is specific to breast cancer. There isn't any arrangement of what aspect of care is requested of or required of primary care, either by referral from the hospital, or the patients volunteering concerns or physical symptoms or anything. There isn't much that I can think of with respect to cancer care or breast cancer survival care.
M1	So, can I ask that, I understand from previous focus group(s) that they actually do encounter cancer survivors in the polyclinic, but it's just that they do not manage the cancer aspect. So, am I correct to say that polyclinic doctors DO encounter them?
A	Yah, we encounter cancer survivors frequently, because the patient should have a record of past history of cancer, main mode of treatment and roughly when (he or she had cancer). I think for every patient, (this information) should be there for every cancer.
M1	So, can I invite your perspective regarding this patient-centric care, which we are advocating for every patient, which is "one family doctor for every patient"? Do you think that it's possible for the polyclinic doctor to look after this group of patient(s)?

A	Okay, (with regards to) the term "patient-centric", I do not know the context for use in this particular case. The meaning of "patient-centric" (has) got different meaning(s) when it is used in, such as, (in) United States. So, to me, I think I interpret (it) to mean that you focus on the person or the patient, the personal aspects, the WHOLE needs of many areas of life and aspects of that particular individual. Right? So, if you call that as "person-(centric care)" or "patient-centric care", I just take that (as) what you mean, okay? So, "patient-centric care" should be in both the hospital and primary care. So, it is not *[trails off]*. There is no need to talk about "patient-centric" here only (as being applicable in) family medicine, so to me, it is a non-issue. So, that is the first part of what you (asked). The second part, you (asked) something else. What is it?
M1	I mean, like, "one family doctor for every patient".
A	Oh! That is definitely helpful towards continuity. It's mainly about continuity and hav(ing) some form of relationship in the long run, so that there is clarity in mutual expectation(s), and there is continuity, and it encourage(s) satisfying experience, as well as some elements of TRUST. So, definitely "one patient, one family physician" as an IDEAL is good. That is my comment.
M1	So, while this is ideal, we know that polyclinic doctors are very stretched for time, in the sense that they only have five to ten minutes per patient. So, do you think it's possible for polyclinic doctors to manage them in the polyclinic setting?
A	To manage who?
M1	The cancer aspect as well - that means, they are well and in terms of cancer care, it's mainly like health promotion and disease prevention, but also looking for long-term side effects of the medication.
A	Yes, for cancer care, of course, it's managed in the earlier stage(s) in prevention, detection, … screening and all that…. It is a very well-established primary care role or scope, as long as patient(s) were diagnosed with cancer, and (are) on long follow-up. What area of their needs (that should be) taken care of in the primary care (setting) is (however) not clear at all, other than wound-dressing – (for example,) some wound(s) for breast cancer, that is not a problem (for us) at all.
M1	Yah, actually in terms of the breast cancer, the only thing which they require is actually a mammogram in terms of the cancer recurrence, but we notice that sometimes they can also have long-term side effects, like even secondary cancers from their chemotherapy, or even peripheral neuropathy from their taxanes which they have been treated (for). So, for these cancers, do you think that primary care physicians are equipped to look after long-term side effects as well?
A	So, what sort of secondary cancers from treatment and chemotherapy?
M1	Because we have encountered cases who have developed secondary leukaemia, even myelodysplastic syndrome maybe ten years down the road when they are

	really quite well and have gone out to the community. Do you think the polyclinic side is equipped to do that?
A	Whether it's skills or knowledge for detecting the complication(s) such as these… well-defined type(s) (of issues), to be able to follow up on this, equipping by way of knowledge and skills doesn't seem to be a problem.
M1	So, this can be taught to them, and like communications, protocols, would they be able -
A	*[Crosstalks]* – yah, if you want to manage this group of patients, it has to be by protocol. That will depend on the crafting out of programmes and all that, trainings (et cetera).
M1	Trainings? Yes, thank you. Okay, so I understand from some senior doctors whom we have spoken to, they think that if these patients are to be managed, they should be managed in specialized clinics, similar to what is being done for dementia and mental health programme now. So, can I understand, how do you select which group of conditions to run specialized clinics (for)?
A	When we have some specialized clinic(s) right now, some of them (are) related to dementia, some of them (are) related to mild psychiatric condition(s), even orthopaedic and so on, how do we select them to be in a more (specialized clinics) rather than… general clinics? *[M1 probes, "Yes, is it by funding-"]* Clinics called by the name of, let's say… one type of service, if you (term) that as "specialized clinic", your question is, how do you select (patients) to go on this kind of programme(s), right? *[M1 replies, "That's right."]* I call this a "programme". So, of course it is delivered through a clinic, and the programme evaluation…. to decide to put (up) a SERVICE as a programme to run through specialized clinics, in my opinion, (is) kind of like (an) investment exercise. Let's say, you need to have *[trails off].* From the beginning, there has to be a clear value that is the first ideal. This is followed up by looking at our resource(s) and delivering what kind of benefit(s). And then, look at the cost, and of course along the way, draw up a clear protocol, identify staff, the workflow, (such as,) from identify(ing) patient(s) to carry(ing) through from appointment(s) to follow-up(s). Then, the SIZE of the programme: economy of scale and things like that. So, if you talk about specialized clinic(s), that's what I think of, and it requires discipline (and) it includes evaluation of the programme, whether the programme is worth going on (for) or (are) there lessons learnt to be used on tweaking or changing the way the programme is done. So, when you talk about specialized programme, all these come to my mind and it is quite often that this kind of activities are not carried out with enough discipline. So, theoretically, yes, specialized programmes, we do have, and that's the best way to do it, (but) there (are) also many not-so-good ways to do it.
M1	*[laughs]* Can you share what are the "not-so-good ways to do it"?

A	(For) example, (there is) no clarity of directive (when) it doesn't come with a clear statement or a few statement(s); not clearly described (to) stakeholders (on) whether it is participation from hospitalists or primary care physicians (and) the roles might not be clear; the scope of the diseases, for example, (in) psychiatry, (there is) "mild", (which) means "how mild (the symptoms are)" and all that; the commitment of the resources (may be missing) and that can include money, because some need money and some even need enhancement to (their) medical records system, EMR (Electronic Medical Record), and it is not cheap – actually not cheap. Many of the programmes, even now (as) SHP (Singhealth Polyclinics) do (it), the EMR (Electronic Medical Records) is not yet done up to fully support the documentation or workflow. They can even use shortcuts like "clinic expansion not so good", and that is (what I mean by) the commitment of resource(s). And about this, measuring the benefit and cost is (also) one (issue), and finally looking at the value it create(s) and then deciding whether it is worth going on, (it) is a separate thing and exercise. So, when I say "not-so-good", it refers to many of these aspects from (the) beginning to the end of creating and running and evaluating the programme not (being) clearly done. If it's clearly done, then there will be reports to (the) correct stakeholder(s) in the right meeting or forum, so that either if there are tweaking, enhancement or even stopping of the programme, it is (being) effected. So, there are many "not-so-good ways" and then, of course, the "best way"!
M1	So, it's very interesting that you talked about "value", because Ministry is really talking about "the three beyonds" to value *[reference to the Ministry of Health's initiative to transform Singapore's healthcare system with three shifts, namely, "beyond hospital to community", "beyond healthcare to health"; and "beyond quality to value".]* So, can you give an example of what you think is a programme that is of value in a polyclinic setting?
A	Right now, there are *[trails off]*. If you said "programmes", then it is not a clinic.
M1	Okay, what is the difference between a "programme" and a "clinic"?
A	A "clinic" is a way of delivering care; a "programme" can be the delivery of services that is not based on clinic. A "service" *[trails off]*. A "clinic" generally (consists of) a doctor, APN (Advanced Practitioner Nurse), then we call it a "clinic". Even (for) nurse-clinician service(s), we don't call it a "clinic", but that's a "programme". Nurse-clinician(s) have touch points with patients, such as through review visit(s) in between doctors' visit, or even palliative care. So, this type (of programme) can generate some value. So, if there is a very good way, is it enough to identify suitable patient(s) and you have a way to deliver a programme to this group of patient(s) in such a manner (that) they benefit the patient(s)? And then you have a way to calculate the cost, and the programme is in a steady state, then you know what value it deliver(s). And this should be evaluated periodically on a regular basis.

M1	So, currently are there any particular programmes that you know SHP (Singhealth Polyclinics) is embarking on?
A	Embarking on? There are always plenty of programmes of different qualities. Some programmes are very small; some programmes are bigger; some are very entrenched, for example, our screening… service is a programme. It's a very, very well-entrenched scope of primary care work, but whether our programme is done in a way that generate(s) a lot of value, that is not the same answer to say that (we) have or have not (any programmes). To evaluate or not in SHP (Singhealth Polyclinics) today, I don't think we have enough evaluation (done).
M1	So, do you think that, in terms of value, screening is really one of the signature programmes of polyclinic?
A	Yah, it is. Screening of diseases is a hallmark of primary care.
M1	And what is success of screening in terms of -
A	*[Crosstalks]* – screening for what?
M1	Screening, I guess, for *[trails off]*. I mean, I'm just trying to make a comparison as to how you evaluate success and value. So, screening will be of chronic diseases and of cancer conditions according to risk stratification, because we know that there is a very established programme of cancer screening in polyclinic, but there (are) very little guidelines on the care of the survivors after they complete treatment and are well and return back to the community. So, do you think that survivorship care -
A	*[Crosstalks]* – yes, later on, it's got very, very little (guidelines).
M1	So, do you think survivorship care is also appropriately, should be, sited in the community, because at the moment, they utilize a lot of resources at the tertiary centre, and the oncologists don't really have the skill sets of health promotion, disease prevention, and *[trails off]*, you know?
A	The hospitals are mainly acute hospitals *[M1 interjects, "Yes that's right."]* and they are less suitable to optimize on the processes involved in, like, screening for complication(s) as a follow-up. So, that is all that I can comment (on). Of course, to use the hospital service is more costly. It costs more. Let's say, this activity is shifted to the polyclinic, then the balance of (the) use of resources in the polyclinic will be called into action and therefore (come under) scrutiny. If we want to do EVERYTHING that is available to do, then practically nothing will be done well or done right, or (is) unsustainable, not because by nature of the work, it's too difficult or what, but by lack of organization, as well as focus. So, if you want to start on a programme in primary care, you need an initial phase of focus until it's so focused that it becomes, not a pilot, but a consolidated programme, then there is ongoing evaluation, and then this becomes a value that the organization maintain(s), including what it cost(s) to maintain, then only (then) it can (be) sustain(ed). So, when hospital looks at things that (are) happening for a LONG time, … they failed

	to be sustainable in the same way the primary care needs to see what is sustainable. So, the leadership and management of primary care can clearly include this in their work – the whole landscape work can be anything – but there (should be) a focus.
M1	So, maybe you can share with us, what is, in terms of (the perspective from the) senior management, what is the vision of primary care towards the next few years? Will it be towards more complex care, integration of patient care, or is it a shared care with the hospitals?
A	Actually, (with regards to) shared care in the hospitals, TOO MUCH has been going on right now, and I do not see enough clarity on HOW they are doing it, or the "HOW part" of it is not shared adequately with clarity. Besides just saying that hospital is too crowded and therefore push more (patients) to primary care, there is not a lot of success in knowing how we assign resource(s). From the very murky, muddled state, we just (say), "Do your best. Fight your fire for the day and get over with each day..", to the other extreme where we can plan ALL your activities and then do them and finally measure the impact, or effect and benefit, and divide it by the cost – we do this very poorly now. It's almost like just trying to go and do the right thing, (but) with not enough consideration of (whether we have) clearly… allocated the right resource(s) among the resources, and it in a manner that is not just for one or two years, but (in a way that is) continued and focused. So, initiation needs focus, but sustenance also need(s) these things to be put in the right place. Whatever I say is still general comments, because we have not done well, not done clearly.
M1	So, have there been any successful shared care models in the polyclinics?
A	With the hospital or with who?
M1	With the hospital.
A	Shared care with hospital? *[M1 interjects, "Because we -"]* Very long ago, one of the MOST successful shared care (example), classically, is antenatal care. NOTHING has come close to that. And antenatal care, of course, as you know, is already completely out of (the) window for primary care. *[M1 agrees, "Yah, that's right. Most of them go straight (to the hospital), right?"]* And there is near-complete loss of skill(s) in primary care for antenatal (care). So, if you ask me about shared care with the hospital, today if you talk about any shared care that I can think of, I can't think of a STRONG example. Even (for) OGD (oesophago-gastroduodenoscopy), where you ask hospital to do the scope where (there is) direct access, you can call that a form of shared care, but the protocol and way to do it to be safe and effective, efficient for the patient, is (still) not catching on (for) the buy-in from the practitioner. You mentioned any other example(s)? I can comment on (them) if you can think of any?
M1	Because we have one sharing from any other cluster polyclinic and they also shared that shared care programmes are not really doing well, and like they have some dermatology programmes with the (National) Skin Centre, they also find that (at

	first) it starts well, but along the way, there is very little utilization and patients still prefer to go straight back -
A	*[Crosstalks]* - yes, for dermatology. We also have orthopaedic(s), where we have mental health, we have even geri(atrics), dementia (et cetera), we have a variety of these, (but) it depends on how it started. For example, if you say this is what this hospital department would like to do, then it's done. Then after a while, the persons involved may NO LONGER be in-charge, then it becomes unsustainable, so this one (has) got element of personality (issues involved). Some say there is the funding and the funding is for two years, and then after two years, what (happens then)? These are the practical issues, right? Then, in some (other programmes), the idea is not bad, but there (are) a lot of practical issues, like OGD (oesophago-gastroduodenoscopy) is one. The idea itself is not bad, but when you really want to operationalize, and the patient (is) to go to (the) hospital from OGD (oesophago-gastroduodenoscopy) and then come out, there is an issue about what happens when the patient arrives at the hospital and whether or not (he is) prepared. (It is also about the) suitability and knowledge of the person performing the OGD (oesophago-gastroduodenoscopy) - basically whether, between the patient and the surgeon, while (the) investigative, somewhat invasive procedure is being carried out (in) the first context, is it a right or good thing. SOME of the surgeons don't think so. And the preparation of the patients to go for the procedure is more than just saying, "You go to this place at certain time. What level of the floor, which door.". You psychologically got to prepare them. The patient must have the time to ASK what to expect, because procedure (induces) anxiety, it's anxiety-causing, and therefore the preparation itself may not be that straightforward. It is not like, (it's) OGD (oesophago-gastroduodenoscopy), just fasting, (but) this requires some preparation, (and is about) a lot more than that. So, for a variety of reason(s), including the follow-up, the follow-up may not be simple, depending on what the surgeon find, such as in OGD (oesophago-gastroduodenoscopy), whether there's a need to use a certain treatment in certain circumstances, whether the drug is available in the polyclinic, there (are) several issues. So, I do not see any particular great example of shared care right now.
M1	So, do you think that this is because of the way our funding goes and the education of the patients?
A	Let's say, just take these two statements, when they say "funding", it's talking about resource. If the resource is skewed to one side without clearly (defining) what it result(s) in benefitting (for) either side(s), then it will fail. (Should) just one side… not feel the benefit, then it will fail already. It takes two sides to meet in the middle, and each side must say, "What's in it for me?", so that is about funding of resource. MOH (Ministry of Health) can say "This is good. Go and do.", but "This is good." is not enough. It has to be (good) for the people directly involved. And if it is monetary, then it should also be directly (beneficial) to the people involved. There's no point saying, "I save money for the world, whereas I bear the burden of the

	cost.". So, that is about funding. (And the other thing) you mentioned is what? Training ah? I don't know what you said.
M1	Training, education, or is it patient's perception, whether they -
A	*[Crosstalks]* – it is patient expectation, like if a patient goes for a procedure, they expect proper briefing, explaining, allaying of fears and safe preparation. Are you sure the primary care doctors in a high-volume situation can focus on delivering that before the so-called procedure? So, in hospitals, is it (that) doctors are left to their own means or is it a clear list, (an) organized arrangement for patient(s) to fully voice their concerns BEFORE they get send off and come back the next time, (already) lying down there to have a scope? So, THAT is actually a GAP that is not closed with our routine processes. It's a very focused one for such patients, if you want them to go (to the) hospital and do procedure. Other than that, (there is the issue of the) follow-up: again, patients must know what to expect after the procedure. Because there is no other service by the hospital, other than the scope itself, (that is why) I'm just thinking this is one example. So, THAT is also a programme that is not successful with us. Even in NHGP (National Healthcare Group Polyclinics), even if we have more numbers, it may or may not be sustainable.
M1	Okay, so finally, maybe we can invite you to share your vision of the future primary care for polyclinic, for example, if you were to have cancer survivors, either (from) colorectal or breast (cancer and) who are WELL, should they navigate the resources themselves? Should they plan for their own future? Who should be responsible? Who are the stakeholders in it?
A	Let's say, you want to focus your interest just on cancer survivor(s), more specifically, let's say in this case, breast cancer, so obviously, as I said earlier, the clinicians' skills (are) adequate to deal with it in the primary care and it can be satisfying for all parties. But… to MAKE A CASE for it, make it focused in its initiation, and then, sustaining it through clarity of roles and ACCCOUNTING it with resource(s), you (also) need all these to succeed. There's no way to succeed in any care programme without clarity in all these. It's not (to) just say, "The idea is good, so why can't it succeed?". It's not like that. So, whether it's like this example you mentioned, follow-up of breast cancer patient survivors, looking out for either episodic needs which may or may not be related to breast cancer, or aspects of the need that is related to the breast cancer and its treatment complication(s) and so on, it's a good idea to make it an idea that is YIELDING value, that requires a lot of talking by stakeholders, and (their) commitment. All these must be in writing – writing, not because you are (being) legalistic and say(ing), "If you don't this, see, I hold you by your word.", but (rather), writing makes it clear, so that people would bring it along with themselves, such as for communication, aide-mémoire and so on.
M1	So, can I just ask, who should be the stakeholders in this?
A	Stakeholders in this case, you have the hospitalists who treat the acute phase; you have the primary care physician(s). And within the primary care physician(s) - for that matter, of the hospitalists as well - it depends on how the department is

	organized (and) the person who represents the institution to speak, must speak with both vested interests, as well as vested power, meaning (that he) makes the decision to commit, and then, HOW to commit. (With regards to) funding, MOH (Ministry of Health) would like to take cognizance as to IF a special fund is needed. It doesn't help if MOH (Ministry of Health) says, "This is a good idea. Deliver it by (this date).", just like that, (because) it will not work well, even if the idea is right, because different institution(s) may not have the kind of same timing for prioritizing projects. So, they are the stakeholders that I have mentioned already – mainly hospitalists and primary care, and if there is (a) certain direction or funding needed, it may involve MOH (Ministry of Health). But of course, if it's just stakeholders, of course, needless to say, patients (are at) the centre. You want to involve them… in a focus group or user representation *[laughs]*, because they are users, they are the stakeholders also. Then, maybe out of this, (it is) organized in a manner that is able to yield clear information, the information becomes "inside", and this would be able to make people, who do the care delivery, design the programme and communicate it very clearly to ALL users, whether it's care provider or the care receiver. Of course, (another) party is the fund (provider), (because) the fund(s) have to come (from) somewhere. Done?
M1	Okay, thank you very much on your perspective.
	[Audio recording ends at 31:40min]
