# Supplementary material for: Implementing a community-based shared care breast cancer survivorship model in Singapore: a qualitative study among primary care practitioners
Source: BMC Prim Care. 2022 Apr 8;23:73. doi: 10.1186/s12875-022-01673-3 (PMC8991467; doi:10.1186/s12875-022-01673-3)
Supplement: Supplementary file 3 — Additional file 3. A compressed folder containing the raw data transcripts and demographics data collection form. [file 12875_2022_1673_MOESM3_ESM.zip › Supplementary Information File 3/IDI (09.05.2018).pdf]

Transcript for IDI 5th September 2018

Key:

Moderator / Interviewer:	M1
Respondent:	Participant A (A)
( ):	Paraphrases, additions to or rectification of grammar, vocabulary and/or truncated sentences.
[ ]:	Non-verbal, e.g. *[xx laughs] [pause]*
…:	Removal of false starts, repetitive or ungrammatical long phrases
CAPITAL LETTER:	When there is a louder emphasis or stressing on a particular word or phrase

M1	About thirty to forty-five minutes.
A	Sure, okay.
M1	We can start recording now. So, thank you for coming to the focus group today. So, we will have an in-depth interview. So, we will go according to this topic guide for today. The first one will be on experiences. So, A, we'll like to have a background survey of your current practice, whether you encounter cancer survivors in your clinical practice?
A	Okay. So, I think, first of all, the background to my practice: currently, I'm doing quite a bit of management, so even my clinical practice is sort of down to about twenty, thirty percent of my time, and in clinic, I look after mainly chronic patients, so the patients (are) seen in the family physician clinic. So, (they are) diabetic patients, patients with multiple chronic diseases and all that. In terms of cancer survivors, because at the moment, really, there is no existing sort of right-siting or shared care programme between (our) polyclinics and any cancer centres, so if I see any patients that have a history of cancer, it's sort of incidental and it's not like a shared care arrangement whereby I look after these patients in between the visits at cancer centre, or at any cancer institutes or in hospitals. So, in a way, we don't actually play the role of looking after these patients in terms of the cancer care or the survival or the period (of) post-(treatment) (and) screening during the remission. And actually, we also don't *[trails off]*. So, our role is really just to look after the chronic illnesses. So, in a way, there is no HANDING OVER of the care, of the interval between the visits at the cancer centres. So, in a way, we don't look after that part per se, even though patient might have history of breast cancer, and they had a mastectomy done, and concurrently on Tamoxifen and all that. It's "by the way" that they just let us know (that) the patient is still under active follow-up with NCC (National Cancer Centre) or any other department, so we just look after the diabetic part of it. You know? So, in a way, the number is very few, because I only see one-and-a-half days of clinic anyway, so certainly (there's) not so much

	exposure in terms of, I think, actively caring for these patients when it comes to the active care, because currently there is no arrangement.
M1	Thank you. Do you feel that this one is a satisfactory model, because it's the same patient that you are managing for the diabetics, but sometimes, you are not really *[trails off]*. I mean, although you say that they are taken care of by the cancer centre, do you feel satisfied as a family physician to just look after one aspect without really knowing about the other aspect(s)?
A	So, I think… certainly, it would be better if we've been informed, at least, of what to look out for, and more to just what to look out for (only), in terms of looking out for symptoms and signs of recurrence and any specific care while the patient is on chemotherapy or whatever, and the specific side effects, whatever, that we need to be looking out for, because not a lot of us have done oncology in the past, and we may not be familiar with that. So, at the basic level, we should be informed, so, take, at least, for example, a letter or something from the oncologists to say that, "Okay, I'm seeing this patient every six months or two years.", or whatever the follow-up is, and "The patient has this, has this, has done this. And then, anything to look out for." You know? I suppose this is not really shared care, in that sense that this is really just keeping each other informed, and MAYBE because of the cancer care, I'm not sure, in terms of the impact on the chronic illnesses, whether we should be looking out for something, (like) when it comes to glycemic control, the blood pressure control, and the nutrition status of the patient, or is there anything that we need to look out for. So, at the moment, we are totally blind. At the higher level, I suppose that's where your shared care comes in. Then, we will actually be ACTIVELY caring for the patients in terms of their cancer care, IN BETWEEN their visits- so, in between, their cancer centre visits can be stretched out a bit longer, so maybe like every year, and in between they come and see us, and we ACTUALLY prescribe the *[trails off]*. Of course, if they are available and they are not so difficult to prescribe certain chemotherapy or hormonal treatment or whatsoever, and in between, (we can) actually be actively caring for this patient. So, I suppose that will be a higher level. So, that will certainly be the most satisfying, but I think – I'm quite sure we'll talk about this later – that there will be some barriers to get to that level. But I suppose at that very basic level, it'll be better if we're informed, so, through a letter or something like that, or through SCM (Sunrise Clinical Manager) *[reference to computer programme for electronic medical records]* (where) there's actually a template for us to actually look at, to know what to look out for, EVEN as we are just reading (and) mainly focusing on the chronic illnesses, BUT because of the cancer treatment, the impact and what we need to look out for, we are informed of that and it will be better.
M1	Okay. So, in terms of the information, when do you think this particular information should be communicated? Should it be done at the beginning, throughout the cancer treatment, or only at the point of time where there is like a transfer of care?

A	I think, realistically, probably at the transfer of care, and provided that in the intensive treatment period, the patient doesn't come back to primary care (or is) not likely to come back to primary care. So, for a lot of cancer patients, I suppose usually during the initial period post-surgery, chemotherapy, whatever, they are instructed to go back to cancer centre for anything, like you run a fever or whatever, you know, neutropenic and then they go back through A&E (Accident & Emergency Department). So, during this period, I suppose REALISTCALLY, you know, with the current workload that we have, of course, it would be nice for us to be informed. So, I refer the patients for a breast lump, I send to them, then okay (they just) disappear. Yah, I don't know what's happening and all that, but of course it'll be nice for us to be informed, like "Okay, this patient has come. Thank you for referring. This patient has come to me. I'm doing this and this and this, and I'm taking over the care of these patients, and even if they have chronic illnesses, somehow (at) the cancer centre over there, there is someone looking after that part as well, but a professional courtesy, I just let you know this is what is happening to the patient.". I think it'll be nice, but I think, practically, it's NOT SO important, but certainly, when patient goes to a phase where he has to come back to primary care for something, and he would be quite community-based and there would be coughs and colds or whatever so he comes back to primary care, then we would be informed, so that at least – *[M1 interjects, "That is at the end of the active treatment?"]* Yah, yah. Basically, at the point where the patient is likely to visit primary care for anything, so that's when I think we should be informed. We may not be participating in the care yet, the cancer care, but since there is a likelihood that we will see the patient for minor issues or continuation of the chronic illnesses (management), then I think we should be informed. And that's it. Then, from here, the next level will really be shared care. (Regarding) that one, I think it'll probably take a while, because the knowledge base has to be built for the polyclinic doctors, the primary care doctors to be a bit more familiar (on) how to care for these patients and what to look out for and all that, when we actually talk about shared care.
M1	That is a very common feedback we have from the focus group(s) that we really have to build up the knowledge. So, from the management point of view, and looking at the education and clinical services, what are the areas that you think should be built up first before we consider this shared care?
A	Okay. So, first of all, I think, (it's) in terms of education and training of staff. I think - I suppose, I'm not sure because it's so many years ago when we did our family medicine, MMed (Masters in Medicine) or whatever - there is (not) enough (syllables) on oncology, and the *[trails off].* But I suppose you can still build up enough through CMEs (Continuing Medical Education) and things like that, but if you SERIOUSLY go into something like shared care, then you have to be more than that. So, probably most of the time, we're trying to arrange for some clinic attachment. So, for the FPs (family physicians) who are spearheading this, pioneering this, we'll send them in to the oncology clinic to see how patients are

	being cared for and all that. So, at that level, of course, there'll be some level for doctors, a point of care to check on. There should be some online resources also, some basic information, whether it's Powerpoint, document on what to look out for (in) the patients, and on this regime or treatment, what the side effects you are expecting and what to look out for and all that. So, that's the education side of it. The other thing would be, I suppose, in terms of support resources, so to care for these patients, if we need certain scans, or labs and all that, to be done, (so) it has to be put in. So, currently, the lab catalogue that we have, the radiology catalogue that we have (et cetera) may have certain limitations, so you are not be able to do the necessary tests, but I suppose most of them are there, like the blood count and sigmoid X-ray, all the tests are there. But we need to follow up (on) the patients about certain markers, cancer markers. We can order at the moment (too), but the next issue is (the) financial part of it. *[M1 clarifies, "Funding issue?"]* Yah, so we're NOT FUNDED for all those tests, so when we do order these tests nowadays, it's very expensive. So, it's not SENSIBLE for patients to actually have these tests in the community. So, we must look at the funding also, to make sure that these tests become affordable when we do it in the polyclinic. So, support services (are) also (necessary) in terms of when we move to a higher level of shared care, it'll no longer just be doctors and nurses. So, in caring for these patients, do we need the allied health professionals, psychologists and, you know, for patients who are physically-disabled, physiotherapists, occupational therapists and things like that, and the necessary allied health professionals in the primary care to be part of the team that looks after these patients. So, I suppose in terms of the clinic structure, I suppose the service(s), the model as well, with the current (arrangement), (it should) certainly not (be) in the general clinic where we're rushing through patients in five to ten minutes. EVEN (in) FPCs (family physician clinics), fifteen to twenty minutes may not be enough for these patients. So, there must be a model, and this model allows enough time for this patient to be seen. And I'm not sure if the SOC (Specialist Outpatient Clinic) may be just as fast, but in primary care where you have to attend to THIS, plus other chronic issues and all that, then the funding MUST BE adequate to support the time that we never to look after these patients in primary care.
M1	So, from our understanding from the other groups, we understand that the polyclinic has specialized clinic(s), as well as family physician clinics. So, for such patients, can they be managed in these settings?
A	Can! So, for special clinics, the *[trails off]*. Some of the special clinics we are running at the moment are funded by the *[trails off]*. But I suppose the understanding is that this funding is not supposed to be forever, you know? We're supposed to use the initial period of funding to learn about how to do things more efficiently. Ultimately, the funding will be taken away, and then we see how to deliver the service, but at least as a start, there should be funding. So, it CAN have this kind of arrangement where Ministry actually gives us the funding for, like, a few years to pilot this in certain places, and then we have to see how best to do it, because at the moment,

	I mean, we don't know how much time is necessary for each consultation, what are the allied health resources that we need, so ultimately I really think that in caring for these patients, it cannot be the usual primary care subvention. It will be higher. But we also should not be actually doing all the things that SOCs (Specialist Outpatient Clinic) actually do, so it has to be, I suppose, scaled down a little bit to make it relevant to primary care, and based on that, how much time we need to spend, what are the resources we need to use, the allied health, the lab tests, whatever, then from there, we have to come up with some kind of funding model to support this.
M1	Actually, why we pick breast cancer in this study population is (because) breast cancer is, of course, the commonest cancer in women, but also (has) the longest survival, and also, nowadays we use hormonal therapy up to ten years, and usually hormonal therapy actually has quite a lot of problems, especially with the osteoporosis, so this is the reason. But in terms of surveillance, what we find in the cancer centre, the main evidence-based (intervention), is usually a yearly mammogram and a physical examination, so in terms of resources, it's actually quite little. So, the mammogram is important, because I guess at the national centre, the radiologists are quite familiar and they are the ones that advise us whether there's any new changes and whether a biopsy is needed. So, we're just hoping to partner, like, primary care, whether in the area besides recurrence, how about in the other aspects, like psychosocial, bone health and all those? You know, are we able to define roles? Then, we can manage in a more holistic way, perhaps at the beginning of the shared care when they move between the tertiary and the primary care. But further on, once the patient is already cancer-free for ten years, fifteen years, twenty years, actually they are just like any other normal patient, because most of these long-term patients… actually default (consultations) and we don't really know where they went to. And most of them actually forget about the surveillance, and sometimes they do come back with long-term side effects, even with recurrences. So, we're just hoping to see that whether this is possible of shared care for long-term survivors, if it is something feasible?
A	I think certainly, it is feasible, so long as *[trails off].* In fact, the boundary has been moving, things we have not looked after in primary care, are actually being seen in the polyclinic, so I think it's possible. I think in the end, my belief is that the training of FP (family practice) actually is very flexible, and it can stretch further, so long as the care… has been adequately, supposed(ly), taught and trained, when it comes to the providing (of) the care components. So, if it's (a) simple examination, what tests to order and at what intervals and all these, those will be (part of the training). So, in the end, it really comes down to the financial model, whether it can support it. But for the *[trails off]*. Since you are using the example of breast cancers, hormonal treatment, (patients are) fairly stable after that, just looking after (the patients) for the surveillance period, what we need to do, whether it's physical examination or mammogram, all these, … so (there should be) no problem. But then, OTHER effects of hormonal treatment, osteoporosis and all that, so, for them, even for as SIMPLE

	as that, currently, at (our) polyclinic, we do have problem with that because we don't have the access to the BMD (bone mineral density test). *[M1 interjects, "Yeah, that's what we heard and it's at private rate, right?"]* So, actually in the past, we just send (patients) to the hospital for private rate, and the hospital is also facing a bit of heat, because if they charge private rate, they will actually get a lot of complaints from patients, (like) "I come from polyclinic, but how come I have to pay so much?". So, now, in fact, we are sending patients to some of the VWOs (Voluntary Welfare Organizations), private providers, that charge quite reasonable rates EVEN as private rates. So, simple things like that have to be sorted out, and I'm quite sure that (for) many other cancer survivors, all they need could just be like, maybe all the cancer markers monitoring and things like that or simple examination. Again, it's then the financial issue will come up. So, if we have to do this test in the hospital, usually it costs, like, twenty dollars, because in hospitals, the subvention is higher; they come to primary care and they charge you a hundred dollars, that's not acceptable for the patients. So, I think the simple things like that would *[trails off]*. So, I think if I put it sort of simplistically, for the really stabilized patients who have been treated and have lower risk of recurrence, all they need is really to have a fixed schedule (to) do this, do this, every five years, ten years, (then) we can certainly do that, so long as we can sort out the financial part for it.
M1	Yes, that brings a very good point, because, like, for example, BMD (bone mineral density test) and mammograms, so I guess if the shared care provides an access to the hospitals to be able to order the tests at subsidized rates, since they are still patients of the hospital, would that be beneficial and make it a more viable option?
A	Certainly, that would be (possible). But of course, the other thing we have to address is the patients' acceptance. So, for oncology, definitely we've been operating a very specialist-centric (model); patient has been living with this sort of model (as well as their) relatives, family members (et cetera); the general understanding is that if you have cancer, you see the specialist. Right? So, we also have to educate the public, to have, generally, greater confidence and trust in primary care, and for them to ALLOW themselves to be right-sited, discharged and so, it's not so easy.
M1	Yah, I guess that is the very traditional thinking, but we encounter some younger patients who come and tell us that "You're actually not doing a lot for us at the national centre and we spend half the day coming here and waiting.", and these are the patients which dropped out. So, we noted that, with more education, we find that there is this group of patients who are asking, "Why we coming? Why can't we go to our family doctors?", so that's why we start thinking of this shared care model, how can it be done. So, in one of the interviews, one of the participants actually say that perhaps there must be a proper handing over, and then, there must be a programme shown to the patient, and they know that it is a partnership. So, do you think that will be a good thing to do?

A	I think can. I mean, we've been doing right-siting in different hospital departments, (so) I think it certainly can be explored. Of course, (with regards to) the success of it, we really have to plan it properly, but sometimes, we just have to really get our hands dirty and do it, before we know whether there are any teething issues, hiccups, patients' acceptance. So, I think certainly it can be done. We probably have to pick the right group, so I'm not sure about breast cancer, whether there (are) any other cancers we should be looking at. Basically, we should start with those simple ones, where the follow-up care is fairly simple, and (it's) probably good to start with some shared care arrangement, rather than clean discharge, and we also need to work out the return path, so that when patients come out, there must be quite a seamless arrangement for patients to go back to the hospital if something happens. And the patients get the confidence. We may need to have something like that, so that when they go to primary care based on all these red flags, they can be sent back to the centre quite seamlessly. And so, I think (it) certainly can be done. Probably just need to take the *[trails off].* In terms of patients, I suppose at the cancer centre, you know the patients better, (such as), who may be more accepting to this kind of arrangement, and then, basically those who are at low-risk of occurrence and (have) less side effects of the treatment that they are on, and I suppose those patients who have lots of chronic illnesses that require them to come to primary care ANYWAY. So, you know, for patients who don't have *[trails off].* Perhaps in Singapore, the concept of having a family doctor is still fairly weak. So many patients actually do jump around and things like that, so the risk of defaulting is very, very, high. For those patients who have chronic illnesses, they are stuck with the polyclinic, but it will not be the same physician all the time; but they have to be there all the time anyway, so I think since they are there, we can catch them.
M1	So, you think this group with the chronic diseases should be the first one(s) we should approach?
A	Yes.
M1	Yah, I think the cancer centre will also re-stratify the patients, so it's mainly the low-risks patients that we think are more likely to have shared care, so -
A	*[Crosstalks]* – so, low-risk, and I suppose, likely older (patients) who have chronic illnesses, then the risks of losing them is not so high, because they have to come back to the polyclinic.
M1	That's right. The younger ones tend to be more mobile, so I guess with education, they know what and how to fend for themselves. *[laughs]*
A	Yah, that's right. I mean, for those, but not for everyone, I suppose the concept is growing, so we do have some people who (are saying), "Oh, I go and see my family doctor all the time, and I'm supposed to get hand(ed) over.". And of course, another point to make is that, I mean, in the end, (in) the polyclinic, twenty percent are (for) primary care, so for any of these programme(s) to have a bigger buy-in and all that, it's always good to involve polyclinics and the GPs (General Practitioners), so

	therefore (for) this primary care network nowadays, GPs are linked to the clusters. So, some of them are very good, senior family physicians with lots of experience, so they can be engaged to take up some of these patients as well.
M1	Actually, we did a pilot study with the GPs (General Practitioners) before we come to the polyclinics. So, for them, especially the younger GPs (General Practitioners), they tend to have problem, say, with time, because they say cancer patients tend to have a lot of concerns, so it's not so suitable. But I guess some older GPs (General Practitioners), you know, when they have more time and they have more experience, I guess those are the group that may have *[trails off]*. And then, I guess with the change in the funding model, this may actually help. But we do note that, like, in the cancer centre, when we look at our patient cohort, about forty-five to fifty percent of them are subsidized patients and the polyclinics are our biggest partners -
A	*[Crosstalks]* – and I suppose after the cancer treatment, a lot of them may have their finances gone drained. So, they will probably prefer to go polyclinic for their follow-up care. But some may prefer to go see GP (General Practitioner), especially where, I guess, you know, depending on the cancer you are talking about, some of them, especially those who are financially-able, may prefer to go private kind of environment, and so GP (General Practice) may suit them better than polyclinics. So, that's the *[trails off]*. So, certainly, I think it can be explored, so we are having lots of all these discussions with different departments. But, I mean, just to share with other departments, usually the big barriers that sometimes give us a hard stop, would be when the care process to inform all these patients is so tedious, and it take(s) up so much time that it's just not doable in primary care. So, I suppose, we're not thinking about those -
M1	*[Crosstalks]* – can you elaborate on what are you referring to?
A	So, take for example, currently, we're trying to do some fall assessment clinic, and based on the initial appreciation after running from the hospital and all that, the consultation time we are looking at for BOTH the nurse and the doctor is very long. You know, you have to do this and do that, do all the assessment. Even for dementia and all that, after scoring, it's forty minutes already or something. Then, for primary care subvention, unless we get external funding, this may not be doable. So, we're still doing it, because it is important and these are the types of conditions that really should be managed in primary care, but without funding, I think it's going to be difficult if we really do it properly. So, that's one aspect. The other one would be where I THINK we have certain governance issues in terms of treatment that's being delivered to patients, like currently, our memory (clinic), our discussion of pain management, about right-siting some of the patients, many of these patients are on the mild narcotics. So, in primary care, of course in order to control, we have very strict rules and regulations of the prescriptions, the length of it and all that. So, if all these patients come to us and need six months of Tramadol *[opioid / narcotic painkiller that is under drug prescription control]* or something like that, we have a

	problem. And I suppose you can control it by protocol and all these, but the general comfort level will be less. So, and of course the other hard stop would be when there is no access to the necessary tests, the labs, the X-rays and all that, then we are stuck. So, I think these are the few things that really *[trails off]*. Of course, the other thing will be when the type of care we are talking about to be right-sited to us is kind of… quite odd-out when you look at the focus of the polyclinic, so that's why when it comes to doing right-sited and all that, we've been focusing a lot on, say, dementia, mental health, medical issues that we can really manage; we are not like looking at surgical issues from the right-siting. *[M1 interjects, "Like what area?"]* Say, take for example, say, Ophthalmology – *[M1 interjects, "You can't do much?"]*, I'm not sure whether there's suggestions for us to put up for lab or something like that, so we just have to refer a lot of time. BUT can we do more, let's say, dilate eyes, we can actually examine the eye properly, than to refer? The caveat is that it may be difficult to train, and if you don't do it all the time, you lose the skills and these are very SPECIFIC skill(s). And then, if you try to do a lot in terms of investigation, examination and all that, (and) you still don't have the surgical skills, you simply can't do certain things like, (then) you're not going to RISK because you are only covered for primary care work, so you won't go and try to take up some foreign body and *[trails off and laughs]*. Yah! So, you are not medically-protected, and therefore (there is) no point looking into those things. So, when it comes to the eye and ENT (Ear, Nose, Throat), they also complain, so you do referrals and some of these things can be investigated further, but after discussing briefly, we also found that it's very difficult for us to do MORE. So, when it comes to some of the surgical areas, because of the limitation, then it's better to explore other models. So, like SNEC (Singapore National Eye Centre) has set up some other primary care – *M1 interjects, "Yah, so you go out."]*. Yah, so we go out. So, if you ask again, I think that's the *[trails off]*.
M1	Okay. So, can you share with us any successful shared care models?
A	I think heart centre is probably the first one. They started *[trails off]*. It wasn't even very conscious in the beginning that they started giving longer outpatient TCU and the cardiologist started giving letters to be given out to the primary care doctors, (like), "Okay, I'm seeing this patient now. Stabilized, no chest pain now. Stent has been put in. His control is these factors: diabetic control, hypertensive control, the lipids. And these are the targets. Go ahead.". So, it started with just letters like that and – *[M1 interjects, "Was there a formal programme?"]* Not really, but in a way, it's good enough for us, in the sense that "You give me all the targets. That's what I'm looking out for.", and you know that the patient is seeing the heart centre every year. But now… when we talk to a specialist, we try to have a more structured programme – "structured", meaning that, I suppose, they give us information based on a certain template, and so that we can see it in our electronic system, and everyone gets the same information. And then, another aspect would be for patients to be fast-tracked back to hospital if something happens. So, like currently, I mean, we are having this discussion about Ophthalmology and about the patients

	who have being referred there, for gout – simple (condition) - and they are started on Allopurinol, and they are okay now, they are pain-free, uric acid levels are all right, then these patients can be right-sited to us. The target needs to be this, and these are the medications to look out for and then, we look after the patients. And if something happens, the characteristic of the pain changes or whatever, we can fast-track back to (the hospital). So, I think these are something quite doable. The dementia and the mental health that we are doing at the moment, we are still at handholding phase where there are actually specialists (who) come to the clinic to help run the clinic with us. So, the thinking is that after a few years, we should have enough people trained, so that we can take over the specialists, and they don't need to come down any more. So, at the moment, I would say that it's still at the beginning sort of training phase. So, yeah, and *[trails off].*
M1	So, besides funding, like for the rheumatological programme that you are talking about, will there likely be a funding –
A	*[Crosstalks]* – that one, probably not really necessary. I would say, it's just part of the chronic disease management, knowing how to titrate the medications, so it's part of the clinical care to be given. I don't remember *[trails off]*. It's not A LOT MORE that we need to do. It's not like we do another two or three sort of disease severity scales or something like that… and in terms of examination and all that, there's not much more that we need to do. So, the way we see it is that it's part of the chronic care. But of course, (for) slightly more complex (conditions), we do hope to see all these patients in the family physician care (clinic), where there is a bit more time given, and so that their hyperuricemia can be cared for in conjunction with the diabetes, the renal disease and things like that. So, for that kind of thing, we feel that it CAN be done with our current subvention model. The problem is when you have to do falls and dementia, and in order to objectively assess these patients, there's a lot more to be done. So, this kind of care (is what) we THINK we would need funding (for).
M1	Because currently, I mean, although like the breast cancer patients are (being seen) in private setting, they actually do scans and do other tests, so usually we just tell them, (going by) evidence-based (intervention), we just do mammogram and we don't assess symptoms unless they tell us. So, for example, if they have a cough that is more than a month, then we say, "Let's do a chest X-ray again. And then you come back again.", so that is very symptom-based actually, not really a protocol. So, for this group of patients, do you think, you know, that means, they are quite suitable?
A	Can, if they *[trails off]*. Again, it's the training. So, of course, protocol-based, there are scores and scales to be done. The good thing is that there IS a structure, even though it is going to take more time, but if (the) funding is there, then in a way, the comfort level for the primary care is there, (like) "Okay, there are these things I can do to look out for things.". So, when there's no necessity to do all these things but (it's only) based on symptoms the patients present and the clinical reasoning of

	what to do, then what comes with it is at least the perception that we need to be experienced, so that -
M1	*[laughs and crosstalks]* – I guess we've to see what it is like.
A	So, we need to be experienced to then sieve out what is important in investigating, but of course if it's quite a good protocol to follow, cough more than three weeks or whatever, X-ray and what to look out for the skin, what to look out for, okay, recurrence coming and quickly do something (et cetera), so I suppose if people can be trained, it's still doable, but of course there will always be this fear that "Oh, what if I miss this out? Or is this cancer?". So, I think (there are) pros and cons. So, if you have all these scores and scales to be done, I think of one level, you do all these and things are not missing, I should not be missing much. But if it's nothing there, and it's really going by patient's symptoms, and the anxiety is okay, then "At what point do I investigate?" and things like that.
M1	So, if we take the role of the family physician mainly (to be) in health promotion and disease prevention, and then recurrence and long-term side effects should belong to the oncologist. So, would that put a more comfortable level -
A	*[Crosstalks]* – okay, so if you insist the *[trails off]*. I think, in the end, you really have to look at the specific group of patients. I think overall, I definitely agree that it is definitely doable, but we probably have to pick the right time and then we slowly expand. So, (we) pick the time that, in a way, they're already out in the community, then we already do hear about them. It's just that at the moment, we don't actively care for these patients. So, like patients who are on Tamoxifen (for) breast cancer, I mean, they are already floating around. It's just that when they come to us, we don't really provide care on that part. We'll be like, "Oh, okay, you have that now! See NCC (National Cancer Centre), okay fine!", then we look after the diabetes part, so we don't *[trails off]*. But at least we already have a touch and feel of these patients, so that we are not scared in that sense.
M1	So, it's like YOUR patient?
A	Yah, so they are my patients, but I only look after the diabetes part. So, these are patients (whom) I actually DO know, because they are already with us, they are not scared, we are not scared, so they are already familiar with us. It's just about "Okay, in that case, now we move to the next level whereby we also do a bit more about the breast cancer part, you know, like what to look out and what test to do and all that.". I think it becomes a bit more natural, whereas if you go to those patients that NORMALLY don't come to the polyclinic, (or) usually at the moment, (they are) stuck in the cancer centre, then they'll be harder to move. They will feel anxious, and when they come to the polyclinic, (it's like), "Wah! Is this cancer? *[M1 laughs]* What is it?", like, you know, that sort of thing? So, it's a bit HARDER, you know, the *[trails off]*. So, I think we pick the right type (of patients), then we slowly expand. That will be easier too. And then, of course we still need to see (that) it's pilot, (therefore) even for FP (family physician) participating in the pilot, we should really

	get them to sit in our clinic and really see what you guys do and things like that, and then *[trails off].* Yah.
M1	So, I suppose that would require some funding to support the training?
A	So, the training part, it certainly would have taken off time too, so more for the institution point of view that I'm losing doctors, still under training, right? And they will get backfield or something like that. So, USUALLY IF there's ministry funding, usually they'll account for that, some of the field sessions of sit-ins, some CME (Continuing Medical Education) sessions, and we probably need some funding to print out, like, educational materials, and NCC (National Cancer Centre) actually give out the things to look out for, so also to give to the patients. And I don't know about cancer patients, whether do they actually have that phone call hotline to call in. *[M1 replies, "They do, for our own patients, yes."]* So, for NCC (National Cancer Centre), if they have shared care with the polyclinic, how is that being done because polyclinic has office hours, so GENERALLY our physicians are not into the idea of having a hotline for patients. *[M1 agrees, "Yeah that's right."]* So – *[M1 interjects, "Yeah, I guess it's difficult to get them to answer the call straightaway?"]* Yeah, so it's different, so I think we just have to look at some of these things. So, we start with simpler patients, so that the need for allied health and the support, psychosocial support may be not so much. So, certainly, we STILL can provide up to a certain degree AND IF the staff involved will have to be trained, we do have Medical Social Workers who are quite keen to go beyond the financial work, to actually do counselling, so we can actually train these people to do it. But it's just that (for) the manpower, we don't have a big army of them. It's very minimal, just a few per clinic. Other therapies, we only have physio(therapy), we have podiatry, we have dietician(s), but (in) small numbers, so if you need more of them, like speech (therapy) and OT (occupational therapy) and all that, we don't have many of them. So, for cancer survivors who need this kind of services, it may be difficult.
M1	I guess that's why certain groups like nasopharyngeal carcinoma, usually those tend to stay in the institution, because they need a lot of support. So, I guess we are already choosing the cancers that are already quite well. Again, (for) colorectal (cancer), we didn't really think about it YET, because after five years, the incidence of recurrence (is) very low – it's about two percent. But it's just that breast cancer survivors, they can even relapse up to twenty years. So, it's with group, that perhaps with education, it may really up-level. But do you think, do you foresee that it's an important area that should receive priority?
A	I think so. It is the *[trails off]*. I've seen for breast cancers and it's not too difficult to do. And depending on which phrase we are drawn in, but I guess it's the patient acceptance, so when it comes to women's care, our workload has been dropping and dropping, because I think in terms of antenatal care, Obstetrics and all that, people don't look up to us anymore. They – *[M1 interjects, "Yah! Why is that so?"]* It's a very *[trails off]*. It's not just any other care. It's Obstetrics and antenatal care, post-natal care (and) these are very personal, intimate kind of (care) and I think a

	lot of the women actually prefer to have it done with their own gynae(cologist) and see the same doctor all the time. *[M1 interjects, "But you do have women's health, women's–"]* We have women's health clinic, so we have the service, but it's just that people's workload has been capped by the very low level (of patients). I think the family planning practice and service, the nurses are still running it, but I don't think they see a lot of patients. The antenatal care is very minimal at the moment. A lot of patients just come to us and say, "How many weeks pregnant? I need a referral to KK (KK Women's and Children's Hospital) or SGH (Singapore General Hospital).", then we just refer. And after that, they also DON'T come to us actively for post-natal care. Currently, we are trying to set up some post-natal care for some of these (patients), but I think generally, I suppose it's the nature of the care that is rather intimate in nature, and then, really, I think most ladies prefer to have their own gynae(cologist), people that they are familiar with and all that. So, I don't know if when it comes to breast cancer, whether the same issue will be there. So, but certainly, I think in terms of the ability to provide this care, I think we are certainly quite happy to explore, and to see what we can do, at which stage we start for following these patients up. And especially for chronic illnesses, it doesn't make sense, because since they come and see us anyway. And, yah.
M!	So, perhaps would it be better to explore with the older group first?
A	Yah, (with regards to) patient selection, I think we probably, as part of this, another phrase of it, you may want to do a patient focus group or whatever and see how open they are and which group is most open to it. I would say that the older ones may be, because since (it's about) practicality. I think the YOUNG ones may not be so. I mean, I've also got a family member, my sister-in-law, who's got breast cancer at a very young age, and she is off the Tamoxifen already and very well and all these things, even though she lives in Australia, she flies back every – don't know how many months – to see the oncologist. *[M1 laughs and interjects, "Oh okay! That's almost like her family physician!"]* Yah, she doesn't want to be referred to the oncologist in Australia, so I think the attachment is there too.
M1	Yah, I guess it's because of the diagnosis and they saved their live(s), so I think *[trails off]*. Yah.
A	Yah, so the young patients who are more financially-capable, they may not do this.
M1	Yah, but I guess, the shared care is more like an option for them, and it's mainly for this group that has a bit more concerns. So, actually why we feel that family physicians will play a big role is we thought that because family physicians have a relationship with the patient that they are more able to manage the psychosocial aspect(s). Is that true of the support in the polyclinic?
A	So, we do have *[trails off]*. I mean, in the community, I think it's certainly a better place for some of this long-term care to deliver, and (for) psychosocial support, we can provide but I wouldn't say our capacity is very high – you know, it's just a few MSWs (Medical Social Workers) who are capable of doing that. The family

	physicians, even when they run FP (family physician clinic), they tend to rush through and all that, so they may not also have time to sit down and listen to you, but (it's) at least better than the general clinic where we're really rushing through cases (in) five to ten minutes, which is definitely NOT adequate for cancer survivors. So, I would say that there has to be at least some kind of second-tiered family physician clinic or special clinic to do this.
M1	But if they go to the special clinic, will that be long-term or is that just a temporary arrangement?
A	I think it depends on how they do it. If they are really quite *[trails off]*. You have to look at the protocol of the care. So, if the care is actually simple and just like any chronic illnesses, they can go to the general clinic, but I THINK for cancer care, the continuity is important, so I would say, probably best for these patients to be cared for at the family physician clinic at least, so that they have a regular physician and someone who knows them, and then it's also easier to talk about psychosocial issues if the relationship is there.
M1	That's right, I guess we may not explore everything in one visit, but with repeated visits and you tend to see them more in the clinic; because in the national centre, they only see (them) once a year. So, if we want to explore a shared care model, would you be able to share, in your experience, how we should go about it?
A	Usually, what I'll do when the specialist department talk about this (is that), first of all, I think usually what we do is of course to set up a meeting with the lead clinician from the other side, and I'll bring my team along to talk about the clinical protocol, so (aspects about) the selection of patients, what is the criteria, and then, when they come to us, what do we need to do, so (it's with regards to) the follow-up of patients, the tests, the care processes, the tests that we are required to do, the lab, the X-rays and all that. Then after that, usually at first screen, if I pick up some BIG barrier, I have to raise it up, like "Eh, that one, I can't do.". Usually, if it looks quite doable, then usually what will happen is that… I will need to clear that with my clinical governance also, so we have our clinic governance meetings every month, where all these new programmes are being cleared. So, once clinically, everyone agrees, usually the management and all the clinical directors will sit in a meeting and (say) "Okay, fine, I think we can do this.", and then, we will look at the operational aspects of it. So, in providing this care, do we need additional funding? Of course, the easier way to go would be that those that can usually be done in our usual FPC (family physician clinic) framework (where) it's just a matter of training for our people to be a bit more familiar, then we can go (ahead). But once it's something that "Wah! This one requires forty-five minutes just to do a first consult, then this one definitely needs external funding.", and then we start to look for external funding, but that external funding may not come, then that's why we get stuck. But if you think that it is actually quite doable in our usual family physician (clinic), fifteen twenty minutes, then it can be quite easy, because then we just make sure that these patients are being scheduled, are given an appointment to

	this clinic. Then, of course, we work out the charges, make sure all tests, we have, and stuff like that. And the operational aspect, usually it'll take some time for my Ops (Operations) people and the Ops (Operations) people from the SOC (Specialist Outpatient Clinic) to work out the arrangements on how patients are being referred, given appointments, and if patients need to be fast-tracked back, what to do. Then, after that, usually of course when you decide on some of the indicators to track. *[M1 clarifies, "KPIs (Key Performance Indicators)?"]* KPI (Key Performance Indicators). I mean, … I suppose just to know, after a period of time, whether it is working or not, whether, when the patients are given the appointments, never turn up, then go back to cancer centre, (then) it's not working. Some (other) KPI (Key Performance Indicators) (include), patients' satisfaction, staff experience, and workload and how many of them actually have to fast-track back *[laughs; M1 laughs too]*, you know, every time call and fast-track straightaway, so it doesn't work. So, usually this sort of KPIs (Key Performance Indicators) that we agree to monitor, and the oncologists' KPIs (Key Performance Indicators). On both sides, there should be KPIs (Key Performance Indicators). USUALLY, institutions like Singhealth and the National Cancer Centre will also explore is there any research opportunities here, so usually I'll just ask, "Is there something you want to do as part of this programme?". Then, once we have settled all then, we already arrange for CME (Continuing Medical Education) for the lead clinician - usually from the SOC (Specialist Outpatient Clinic) side usually - to give us some CME (Continuing Medical Education) lesson and mainly focusing on how to look after these patients coming out to us. Then, after that, we USUALLY launch it among two clinics, to try (out for) teething issues or whatever. Some get stuck you know, like this heart failure programme that we are doing with *[name of colleague]*, we are quite stuck at the moment, because it doesn't seem careful even with patient selection (because) a lot of them don't seem very stable, like they come to us and they get referred back to SOCs (Specialist Outpatient Clinics) and A&E (Accident & Emergency) – *[M1 interjects, "So the referral rates are quite high?"]* They get decompensated quite frequently, so I don't know whether it's the patient selection, or whether it's actually the protocol itself that is not tight enough and they are not well enough and not well-controlled, and they get sent back very frequently. So, at the moment, we're still scratching our head(s) whether it's working or not. So, then (we have) the CME (Continuing Medical Education), pilot two clinics, then we just decide, "Okay, let's look at the data again in three months.". If it's working, then usually we need to ask management, "Are you guys comfortable that it goes to ALL CLINICS?". Then, usually that's how we do all these programmes.
M1	So, can I ask, do we need to get the fund, or do we need to get the grant before we consider starting?
A	I think it's always good to, even before we start talking, whether it's (about) possibilities, in a way, whether we need *[trails off]*. I'm quite sure additional grant is always needed, so even when we see these patients, is there a necessity to have some new templates in the EMR (Electronic Medical Record), or whether there's

	some data extraction, sometimes (for) the ideas, the charges and all that. So, it's always one part, but this may be very minimal, but so long as I think these are quite minimal, if really, the management for both parties are quite keen to do this, a few thousand dollars out, never mind it's okay. But what is the big hard stop barrier will be where we need additional money to even fund the doctors and the nurses to do (it), because it is currently not sustainable to do this in the general clinic and the family physician clinic, that it takes a lot more time than that, then the operating costs of having the nurses and doctors will have to be separately funded, then it gets stuck. Unless we know that there's some foundation that wants to fund this. But if we look at the care processes as needed, and (if) actually it's very good, it's just part of our usual FPC, the family physician clinic, (and it takes only) fifteen minutes, (then there is) no problem (and) we can actually do this as part of care. And then, without the need for the funding for manpower, I think it makes it a lot easier, because once you need money, (to get it from the) ministry side, it's going to be very hard, unless they have a burning issue - but I'm not sure, because they don't talk about oncology; they only talk about those SOCs (Specialist Outpatient Clinics) with long waiting time, so (for) cardio(logy), Gastro(ology), they are keen to do something to try to help. So, if not the ministry, then I suppose we are looking at those big foundations. So, (it depends on) whether they are keen to support this. So, I think it'll be good upfront to see whether there's any possibility, but if (there's) really not possibility, then we just have to really start discussing and look at the care path, and so that we get an appreciation (of) can I actually see this as a normal patient under the family physician clinic. If I can do that, then (there's) no problem, because the family physician clinic is, itself, in a way, sustainable, even with the fifteen, twenty minutes intervention, (there's) no problem. *[M1 clarifies, "So, there's additional subvention for –?"]* It's a bit higher for patients registered to our general clinic. It's a bit higher, because this are for complex, chronic patients focusing on diabetics. So, if we can actually put the patients in that clinic, then it's actually doable with the time that's accorded, then I suppose there's no problem.
M1	That means there's no particular requirement to go to the family physician (and) it's not to say that you must have diabetes or hypertension?
A	We still focus on mainly diabetic patients, but in a way, we make it flexible to also open it up to the multiple chronic illnesses, so I DO have a lot of patients who are seeing us in the FPC (family physician clinic) and who have heart disease, stroke and all these. So, we're not so particularly only about diabetics, but I would say that MOST of the patients in FPC (family physician clinic) are diabetic patients, but we also open up to see others if we can.
M1	Okay, thank you so much for your time. *[A replies and laughs, "Sorry!"]* I'm going to stop the recording now.
	[Audio recording ends at 53:29min]
