# Supplementary material for: Implementing a community-based shared care breast cancer survivorship model in Singapore: a qualitative study among primary care practitioners
Source: BMC Prim Care. 2022 Apr 8;23:73. doi: 10.1186/s12875-022-01673-3 (PMC8991467; doi:10.1186/s12875-022-01673-3)
Supplement: Supplementary file 3 — Additional file 3. A compressed folder containing the raw data transcripts and demographics data collection form. [file 12875_2022_1673_MOESM3_ESM.zip › Supplementary Information File 3/FGD (07.07.2018).pdf]

Transcript for Focus Group Interview 7th July 2018

Key:

Moderator / Interviewer:	M1, M2
Respondent:	Participant A (A)<br>Participant B (B)<br>Participant C (C)
( ):	Paraphrases, additions to or rectification of grammar, vocabulary and/or truncated sentences.
[ ]:	Non-verbal, e.g. *[xx laughs] [pause]*
…:	Removal of false starts, repetitive or ungrammatical long phrases
CAPITAL LETTER:	When there is a louder emphasis or stressing on a particular word or phrase

M1	*[Taking time to settle down; 0:00 – 0:10min]* Okay, if we're ready, one, two, three, start.
M2	Yes.
M1	Thank you everyone. Welcome to our focus group discussion. We have six topics for discussions today. We'll go on to the first topic straightaway. Maybe I will invite each doctor to introduce yourself, and at the same time, to talk about the background of your current practice. So, the question is, "Can you share with us some of your experience with cancer survivors?" We shall start with A?
A	Okay, hello, I'm Dr *[states name; omitted for reasons of confidentiality]*. *[M2 laughs; M1 replies, "Don't mention name. Dr A."]* Oh! A. Ops! *[M2 jokes, "Delete!"; pauses from 0:57 – 1:07min]*
M1	Okay, can start again.
M2	Start again *[laughs]*. Identify yourself as A, B, C. I think you can just *[trails off]*.
M1	Okay, from the beginning. *[unidentified female replies, "So, you start first."]* Okay, I start first. And when we refer to each other, we'll say that, "As what A says. As what C says.".
A	Hello, I'm A. Over the years in our practice, we have had encounters with various cancer survivors, and really, one of the things that we find that we can be of a bit of benefit to patients is really to be of a(n) emotional support and a support from (the) primary care point of view, so whether it is to manage their immediate medical problem or some of their chronic diseases, because many of them actually WILL have chronic diseases, especially those who are older. But it (also) goes further than that, because we're in the primary care sector, and we have, very often, to manage challenges that they will have when it comes to their families, with their children, … and even sometimes, if they are younger, for their parents, if they are the carers.

	So, these are aspects of care for cancer survivors that we actually have to be very cognizant of, that we actually have to manage when we do see them.
M1	Thank you, A. B?
B	I'm B. I have been in the practice and have encountered a number of cancer survivors, and I find that the main issues we have with them is during the initial stage when they are newly-diagnosed, when they come and see us for non-related problems, but we can understand that their anxiety level may be much higher. And I find that they really need a lot of reassurance, the emotional support, because (for) any minor illness, they always have this concern whether it will aggravate and whether their cancer have grown back. But as the years go by, once every few years when they are seeing their oncologists and they are in remission, usually I find that once they have the self-confidence and the reassurance, then they can be seen like any other patient, like your other patients.
C	I'm C. I work in a town practice with corporate type (of) clients mainly. Yes… I also come across some cancer survivor patients, and some of the concerns, I think, (of) cancer survivors, one of the first concerns is whether he's going to get a recurrence *[laughs lightly]*. So, that's their first concern. And of course, that diminishes with time as they go on. Some of the other concerns are also (that) they are concerned whether their family is going to get it. Some of them know it's related and (there is) familial risk, so (they are concerned) whether their family members might *[trails off]*. They are always interested in screening their family members besides themselves. So, I think those are the main concerns. They need a lot of psychosocial support in the beginning, after they come out of their cancer, and some of them are still going through chemotherapy and the treatment, which is not finished yet. And of course, I encounter side effects, which I have to manage, because we know them quite well from the management of their other problems. I think those are the main things that a family physician can help with cancer survivors.
M1	Okay. Let's go on to the second topic about discussing the perceived barriers of the proposed shared care model. So, the question is, "What are some of the barriers that you can foresee with this shared care model? You can discuss them in terms of patient-related, physician-related, and healthcare-system-related (factors).". *[pause; 5:42 – 5:49min]* Anyone would like to share?
C	C here. Some of the barriers in shared care would be - especially if the shared care is with specialists in the public sector, one (barrier) would be - (the) communication and accessibility to the oncologists who are managing the patients, because we need easy accessibility to have effective goal management, like when we come across problems with the patients that we have. Doctor barriers, of course, are also there – doctors do not have sufficient training or sufficient knowledge in the management of such patients, which CAN BE acquired, right? Then, if they don't have the knowledge, they tend to refer the patients back too easily or too fast back to the tertiary institution and to the oncologists, whereas I think if they had some

	training and some knowledge, they can manage quite a lot of the problems in the community. Patient factors also – a lot of patients need confidence in their family physicians to be able to manage their problems, before they can be effectively managed in the community, otherwise they would want, always, to go back to their specialists. So, these are some of them, maybe?
M1	A?
A	A here. I think that I agree very much with C on his views. I think, further to that, I feel that, very much of importance to many patients, is actually the presence or absence of financial relievers, that actually help to maintain the patient in whichever sector that would be taking care of their problem. So, for example, let's say, we may have some issues that can be easily managed in the primary care sector, but because of the cost that may be involved, maybe to investigate or to have some therapy and so on, it may be, from the fiscal point of view, much more advantageous for the patient to go back to the hospital also. So, there are these financial barriers, which I think… are very much systems-related. Patients basically will have confidence in their GPs (General Practitioners) and family physicians if, (firstly), they have been with the doctor for a long time and they have an implicit trust. But because our system is … (a system in which patients do) not necessarily stay with one doctor, THAT actually EATS into that degree of confidence that you may actually have in your primary care physician. So, for example, if you go to a polyclinic or you go to a government-subsidized Specialist Outpatient Clinic, they don't always see the same doctor. They are always changing, and the reliance is upon the record (in order for them) to have a continuity of care. But we know that human beings are not pieces of paper - they have heart; they have feelings; they have good days and bad days. And if they have primary care physicians or secondary care physicians that (are) constant and they are familiar with them, they are much more likely to share some of their more intimate concerns that they may not share with other people. So, I think that is one of the barriers I find, I think not just here, but in many aspects of medicine. So, to have ONE primary care, one PRIMARY condition to take care of, whether it's in secondary care or in primary care, I think it's actually very important.
M1	B?
B	I'm B here. I agree very much with what C has mentioned. The main thing is the accessibility to the doctor and knowing the chemotherapy that is going on, and also the possible side effects, and the current health status of the patient, in terms of like, I mean sometimes when they may come for *[inaudible; 10:15min]* or vaccinations, or they are still on chemotherapy, we want to know what is their latest blood count. And so, accessibility is very important. And I think it will be good also for the oncologist to give a little memo, so that from the physician's point of view, we know what medication the patient is on and what are the side effects that they

	may expect, so that when (the patients) come to see us for non-related problems, at least we know about any complications and any other issues to look out for.
M1	Okay, let's us go on to the third part, which is actually a piece of information. As we have discussed, they have difficulty in access, so let us look at that piece of information. This is a survivorship care plan, which is developed by the American Society of Clinical Oncology. So, we have done some adaptations to our local population, and we would like to gather your feedback (on) whether it is a usual means of communication between primary and tertiary care, and vice versa, and what information in this care plan is useful, what is missing and what other information that should be included as well. *[pause; 11:36 – 11:45min]* Okay, you can look through this together. So, in the first part, we have the patient information. So, we think it's important to know who is the primary care provider, and then, to go on, (also) the names of the surgeons, the radio(logist), the medical oncologist, as well as the contact information. Then, in the treatment, there are two main components of this care plan: first is that of the "treatment summary", what they have gone through. So, in terms of cancer survivors, this will be patients who have completed their active treatment. So, will this information be adequate? Too much information or anything that is missing? This (part) will tell us whether they are hormone-positive, whether they are HERT2-positive, the stage, what surgery they have gone through, when was it done, whether the lymph nodes were involved, was radiation done, which part was treated and whether they are currently on any chemotherapy or hormonal therapy (et cetera). Then, "ongoing treatment": are they on Tamoxifen *[drug for preventing the relapse of hormone-receptor-positive breast cancer]*. These are some of the side effects that have been listed, which we hope can guide primary care doctors to take note of. Like for example, if they are on Tamoxifen, if they are post-menopausal, and if they do come back with per vaginal bleed, it could mean that there could be a side effect of endometrial cancer. So, that would call for an early referral back. Aromatase inhibitors is another big drug which the patients are on, and currently, many of them are on (them) for ten years. And there (are) also other injections. And ALL of these render them post-menopausal as the tumour(s), most of them, are actually hormone-sensitive. As a result, they may have undergone menopausal symptoms, fatigue, numbness, psychosocial depression. So, the second part of the survivorship care plan is actually the follow-up care. So, this will be in terms of cancer surveillance, (and) also if we can provide the details of the provider, how often he's seen, and what are the tests which are necessary, and then, what are the surveillance which is RECOMMENDED, like if there are aromatase inhibitors, then they will recommend bone (mineral) density test for osteoporosis. And then, as a result of your premature menopause or even in a cancer diagnosis, they may have anxiety, depression, mental health issues. And we also recognize an entity called "chemo brain", in which they may have memory or concentration loss. And because of the premature menopause, many of them have problems with weight control as well, and they find themselves being (over)weight after completing the chemotherapy. And also, the other area about smoking, we know that in many patients, they still continue smoking,

	although the very focus on coming back for the surveillance, mammograms and scans, they have forgotten that they also need to protect other areas. Sexual function is another area which we seldom really talk about. And the lifestyle behaviours like alcohol and diet. So, we'll like your feedback (on) whether you think that this is useful or whether there's too much information to the primary care provider.
C	*[laughs lightly]* Okay, C here. I think it's very good! I think it's very comprehensive. And personally, I like all the information that's listed there, because it's very useful. Maybe sometimes else they could add on (is) red flags, maybe something to list what are the urgent things for which we need to refer back or call the oncologist (for) – that could be on another section. But I think this information pertaining to breast cancer is very good. In one document, I can get all the information I need about the patient and the treatment that was given. It IS very useful for GPs (General Practitioners).
M1	Okay, thank you.
A	A here. I would be in total agreement with C, because I think this sort of document is LONG in the waiting for. If every patient having an oncological problem has this going out for whichever cancer they may have, actually, in one step, they deliver to you good primary care support for your patients in the community, and you would actually have less unnecessary recourse and revisits to the emergency department and things like that. One of the things I find, very often is the case, especially for patients who come from SGH (Singapore General Hospital)) is that, when there is a problem, essentially, the default instruction to them is to "Go the A&E (Accident & Emergency)!", which is GOOD, because A&E actually has got a lot of facilities, but the trouble is that it is A&E and therefore it is different. So, THIS actually will be putting the A&E into the confines, not just within the hospital, but bringing in to the community, especially if they have their own primary care provider, their own family care physician. So, they will be able to perform as the eyes and ears at the co-phase, so that they can actually manage, rather than have to have them run all over the place. So, this is very good. I also AGREE that it is useful to have the red flags put up, because not everyone may be as current on the American (Society of Clinical Oncology) information as the rest, so having a sort of an aide-mémoire of red flags would be very useful.
M1	Okay. B?
B	B. I think this survivorship care plan is very comprehensive and actually very helpful. I think both A and C have already mentioned a lot. But I was just wondering if there is this one little bit we could add in: this is for the slightly more advanced (cancers), if they have had any PET scan done before for metastasis, *[inaudible; 18:16min]*. I think it can be added here also, so that it can help us in our management.
M1	Okay. So, from the feedback from previous focus groups, they find that this is really overwhelming, the amount of information, and to deliver the care, to read through

	(this) in five to ten minutes, as is the (consultation) time for most GP General Practice) clinics, it's really not realistic. So, what do you think about it?
A	I think that that's not true. *[C agrees, "Yah!"]* I think if you don't wish to manage your patients, then this is not useful. *[C agrees, "Yup!"]* If you WANT to manage your patients, then this is EXTREMELY useful. *[C agrees, "Yes."]* In fact, I wouldn't take five minutes! I just click (on) this, and like this, straightaway, by looking in twenty seconds, I would have an idea of what is going on. And I know exactly what to look for - it's just like when you are screening through results and things like that, this is like screening through results. And I totally agree with B with regards to the significance of having a PET scan. A PET scan is INCREDIBLY useful and if we were to know that, then we know that the PET scan is going to light up here and there and everywhere and that actually goes up exponentially. So, I don't think that having this sort of information is a problem. I think that it is a GREAT AID. Even if it's not an aid for the doctors, it's a great aid for the patient because it actually tells the patient what's going on, so you are EMPOWERING the patient by having this. When you empower the patient to have this, you empower the primary care to be able to help manage this at a MUCH HIGHER LEVEL that they would normally do. If I as a primary care provider, I am only interested in coughs, colds and sore throats, then so be it. *[C agrees, "Yup."]* BUT we are family physicians. We take care of the whole (of) patients. We are not "cough cold doctors".
C	C here. Yah, I agree. I don't think this is overwhelming. First of all, you want all the information you want in one sheet. And this document is going to last throughout all your follow-up visits. You do not have the need to go and look for more information, especially on NEHR (National Electronic Health Record), because you have to sieve through more information by looking through. And as I've said, the document will stay with the patients in your notes, so the patient comes for subsequent visits, the information is there. Besides PET scan, this is very good that you can put in all the lab results. All the lab results that were done and (were) relevant. So, (with) PET scans, lab results (et cetera), you get a good snapshot. I don't think it's overwhelming. You can easily go through it. Even though you might not USE all the information that is there in one shot, but at least the base is there. You can use bits and pieces of the information that is relevant to THAT particular visit and management, but like I've said, it can be stretched over many visits.
B	B here. I just have a point of add: … I'm just wondering if it MAY BE useful for the patient to carry a separate card with some of the essential information, like when was the last mammogram done, when was the last time you had any blood test done (et cetera), just to have the basics in hand, (and) the dates, so that it would help us in our review and we don't have to scan through everything. We have this: the patients carry another card saying what has been done recently, just the procedures and the dates.
M1	Okay, those are very good thoughts. And (I want to add) just one last point to the care plan: this is a one-way care plan and it's from the oncologist to the primary

	care physician. Do you think that there's a role for the primary care physician to input into the plan and how do you go about it?
A	I think we might be looking at a plan that is actually done from the onco(logy) side to the primary care physician. This is a very useful piece of information. Of course, like example, we will see patients who have issues happening, and quite often, when we have patients who are seeing some specialists in the hospital, and something happens to them, and what do you do? You talk to the patient, you write down a memo, and the memo goes back to the doctor, saying, "Hey, this is what is happening, and this is what I'm doing. Just to let you know.". So, it's actually communicating, whether it is communicating by electronically or with a bit of paper, I think the MAIN thing is that THAT communication should be facilitated, should be encouraged, so that (the) folks in the tertiary centres would be cognizant of what is happening in the community, because very often, they may not know what is going on out there; they may not know that the family is having a meltdown or something like that, because of some issues that (are) going on at the same time. So, these things are communicated, so that more resources can be activated to actually help to manage the problems, I think that will be very useful.
C	C here. Yah, so I think it's a good idea to make it (a) two-way interaction in a co-management plan. There must be also a way for the GPs (General Practitioners) to also feedback to the oncologists. So, we can actually use a similar form, a template, that the GP (General Practitioner) does fill up, so, things like what investigations the GP (General Practitioner) had done in the interim before following up with the oncologists, any complaints that the patients have brought up to the GP (General Practitioner) and any particular treatment that the GP (General Practitioner) had given to the patient, that would be relevant to the oncologists to know. So, it's also a counterpart of this form that goes back to the oncologists. It can also be in the form of a card that is constantly updated, not just a piece of paper. So, I think that facilitates two-way process and communication. Of course, ideally, it needs to be about the accessibility, because this one facilitates the accessibility.
M1	Thank you. Let's go on to the next topic about motivations.
M2	So, we decided to split this into half, so I'm just doing the second half. So, A, you mentioned that as family physicians, you are more than just "cough and cold doctors". So, I'd just like to explore what are the motivations for you to spend your previous Saturday afternoon here to learn more about oncology and to give feedback? Essentially, what are YOUR motivations to participate in this shared care programme?
A	Actually, I mean, there are many *[laughs]* motivations why we are here. One of the biggest (motivation) is actually (that) your primary investigators are actually doing it. So, but THAT ASIDE, actually for us, when we manage our patients, we manage them not as a disease; we don't manage them as a number; we manage them as a WHOLE person, and we try and do so as holistically as we can. So, when we manage

	the patients holistically, we don't just manage them, (but) we (also) manage their families, we manage the communities. And when they really have a problem like this, it really is something that is earth-shattering for many of them. And very often when we first started practice, we do the best that we can, with the skills and the facilities that we have on hand, and in the earlier days, it was really very, very little. So, as we go along, we discover there are agencies and things we can use; there are methods that we can use to manage them better. So, we go along. So, one of the things that motivates me is actually to be able to have some form of guidance that can be instituted as a national objective, so that you are really able to transfer substantial part of that care for patients who (are) cancer survivors, or even during the time when they are actually going through their cancer treatment, so (it's whether) the primary care community is scaled up enough and have enough community assets to help to manage these patients. So, very often, we in primary care, we do not have that INSIGHT into what's been done or what is available and what the particular plans are and so on. So, once we actually have a way to understand this better and to be EQUIPPED with the TOOLS to manage patients like these in the community, it will transform the way we manage patients with cancer or who have survived cancer in the community. So, that's MY motivation.
B	B. My motivation is very simple *[laughs]* – I'm here to learn and find out more about this shared care programme, so that I can improve and help to make the care of the patients a little bit more holistic, and also to help to contribute to what I have experienced in the management of cancer survivors.
C	I'm C. Well, I'm a strong believer in co-management, so that goes hand-in-hand with family medicine training. The reason (why) we all trained in family medicine is actually to better manage our patients holistically, and there's the end to manage them BETTER in the community, whereas I think many of them are being managed in the specialist setting or the institutional setting. So, to bring them back to the community, GPs (General Practitioners) need to better trained. Right? Once they've had that training, there needs to be co-management relationship with the specialists, two ways – it works both ways. So, having a MODEL like this makes that easier. Right? So, when you facilitate the co-management by having templates, by having information that is EASILY accessible between the two of them, then co-management becomes a lot more easier. So, that's why that's my motivation, because attending this co-management programme enables me to learn more a bit, and then, (to) participate in this programme as well. This applies to all things in medicine, not just (to) cancer survival.
M1	Okay, can I just add on: because from what I gather from other groups, they say that many shared care programmes eventually fail. Have you experienced that and why do you think is so?
C	*[pause; 29:43 -29:48min]* I think, it could be, again – C here – shared care programmes can fail? First(ly), you look at the three factors: doctor factors, patient factors and institution factors. So, everywhere along the way, there are obstacles.

	So, if you don't overcome the obstacles, then the programmes will fail. So, accessibility is one *[laughs]* obstacle. Right? Without the accessibility to the specialist, the programme won't work. You cannot have co-management. And here, I must say there is a difference between the public sector and the private sector. So, private sectors specialists are far more accessible than government sector specialists. So, when you have a patient in front of you in the room, and then you want to contact the specialist, you need to ask some questions, it's very hard to do so, whereas the private sector specialists might just be a phone call away *[laughs]*. That's an example of accessibility. Then, from the doctors' point of view, there's fear and there's a lack of training. If you are ready to co-manage, you will never co-manage, so you have to get yourself ready for that, (so) you need to be trained, or at least you need more information. Training doesn't need to be very deep, like, extensive. It just means that you need the means (to handle) common everyday problems that occur in the context of cancer survivorship. And I think from the system point of view, I think it's just (about) getting it right - the co-management system itself must be right for the various reasons I mentioned earlier. I will be interested to know *[trails off]*. So, if a programme fails, you have to analyse why it failed. You can't just say it failed. You need to know why.
A	A here. Having done a number of shared care programmes, I think… the main thing is, when it fails, it depends on what you mean by "failure". If "failure" is because the patient doesn't want to go to the shared care or the person that is designated, then it's just a patient factor; it's a doctor-patient-relationship factor and so on, and whether the care provided by the primary care provider is AT LEAST of the same quality that they will receive when they are in government hospital. Very often, the barriers from that point of view is purely financial. Purely financial. So, if they have no problems with the financial issues, they will come and see (us), and that has been my experience. I find that a lot of these shared care programmes come down to whether agenda are being fulfilled. So, if the tertiary centre has an agenda to fulfil, has "x" number of people to a primary care sector of a certain type, then it will decant (the patients). Very often, their agenda will change halfway. Maybe they have another primary care facility that they DEEM must be supported (instead), then straightaway, the decanting to the original primary care centre dries up or it's even stopped and that patient is steered to another place. So, you must be very clear that when you have a shared care programme, this sort of considerations will need to cease. Once you establish relationship with your partner in your community, that cannot change just because it makes you more money to send for the other side. And it comes back to what I said from the beginning – it's about financing. It's all about financing. So, the financing part affects the institution; that affects the patients particularly; and then, of course, many of the times, the primary care provider is actually doing a national service. And it's when the other considerations come into being, and the primary care doctor will actually say, "Well, I'm doing this as national service and this is how you still treat me. I'm not going to do any longer.". So, this is how it works. So, ownership of the patients, whether it's ownership by one (party), once you've already been given that care, and then, the

	doctor, the primary care provider must feel that the intense relationship and ownership of that patient that they must take care of in the community. And the institution MUST have ownership of the relationship, like, "Yah, this is my partner. Must take of him for me. And this is my patient. I must take care of him. So, together, we take care of him properly TOGETHER.", then your shared care model will not perish because it's built on ownership; it's built on relationship; it's built on responsibilities between the partners. And at the end of the day, it's that working relationship that is established, that if it has longevity, it will actually fuse much better too. So, in time, you'll find that, you know that people have often said that when you do something often enough, you get good at it. So, if you do something ten thousand hours, so you will do very well. So, just imagine you do the same for this sort of treatment of patients who survived cancer. You get better and better, and before long, it's almost like instinctive. So, this is where I see the partnership can coexist. It's really about relationships.
M2	So, I'm going to move on to the next section, which is on relationship(s) with our stakeholders. So, perhaps I'll start with where C… mentioned earlier that one of the major barriers is that of the communication back with the specialist ESPECIALLY in the government sector, and you find that it's a lot easier to, (like,) if you want to consult back with the private specialist. So, what other potential barriers you can perceive that can affect this sort of like seamless coordination and transition of care between the primary care, as well as the specialist setting?
C	Well, C here. We talked about some of it already. In terms of communication itself, the barrier can be the doctor himself. So, in the government sector, if doctors don't want to *[laughs]* be accessible, they don't want to accessible, to MAKE themselves accessible. I mean, this is as simple as emails and phone numbers and, you know? And the GPs, you can access them directly. If you have to go through the system to access them, then we can never find the doctors, especially in times of need. It takes simply too long. If you want to go through the operator, I think you can forget it. You cannot *[laughs]* find the doctor that you need, whereas for private specialist, (you can) just call (on) the phone. Nowadays you even have "whatsapp" *[laughs].* Right? You can send in pictures, you can send in anything you want, so it's immediate. I mean, (in) this digital age, this shouldn't be problem, isn't it? I am happy to say that there are some government specialists who(m) we have good relations with, but it all boils down to individual doctor and his personality. So, we tend to choose the ones who are easy to communicate with and we refer to them in the end. And also, the department and the institution (are) also important, because they support the doctors, they support communication, (then) it will happen. They can make it available. That's as far as communication is concerned. So, (with regards to) the relationship with the patient and the three-way relationship with the stakeholders, I mean, we're talking about the three main stakeholders, right?
M2	Are there any other stakeholders?

C	Yah, the managed healthcare? *[everyone laughs]* Third-party administrators, that's another problem! If THEY are involved, then it's a BIG problem. Shared care becomes very difficult if you have a third party, simply because they control costs and when they try to control COSTS in the private sector, they curtail or they restrict choice, and when choice is restricted, your avenues of management, communication (et cetera), everything is very difficult. And then, it ruins the patient-doctor relationship. That's one obstacle if you have another third party involved. The government can also be the third-party involved, but I think that's where the financing (comes in, that) we were talking about. If they have a role to play, then there's another stakeholder. But otherwise, it's between the doctor, patient and the specialist. Barriers? I think, training, knowledge? Something we've already talked about. That's my take on it. I think that's important.
M2	Anyone else?
A	A here. I think if we look at patients in the community, they actually also will be often using the services offered by hospice care and palliative care teams, and so on. So, the allied health services are also (involved) and would need to be in the conversation. So, very often, a team-based approach is very useful, because each part of the team will be able to deliver a certain part of the care, because the primary care provider may not be able to manage EVERYTHING and so on. So, unless he or she actually is part of a team that can provide the service, very often, especially for small practices, they may not have that. But they are still very much very important in the formula, because they may be the ones who know the patients. So, if we were able to have a close contact and the use of folks in the allied health sector and palliative care and hospice care and so on, I think that will be of course be ideal. I mean, I've had patients where we have had end-of-life management, where we worked very closely with the hospice care association nurse who goes down and monitor and so on, and essentially, because we are very nearby and we are also very close to the patients and their families, so they utilize US to help to make decisions and go down and help them to manage the patients better. So, it's that kind of activation of community resources that I find very, very helpful, you know, we've found very helpful in the past in cases before, in various types of problems.
B *[inaudible; 41:30 – 41:56min]*	B here. I totally agree with what A has mentioned *[inaudible segment]* especially for those in the advanced stage of cancer that are unable to come to the clinics or hospitals. So, we need *[inaudible segment].*
M1	Would you be able to give us an example of your experience with the nurses and in your own clinic setting?
A	Where they've actually had the pain relief and so on, and of course they are trained and they have a schedule to follow. But sometimes, they have other problems, like maybe patients are not doing so well; maybe they have bed sores, and so they need cleaning and so on. And so, the hospice care doctor will come down and because

	we've been seeing them for a while and have managed them, then they actually will give us maybe a summary of what's been going on for the past couple of days. And then after that, we get to a point where we know each other so well that I would give my number to the nurse – MY NUMBER, my personal number – and she will give me HER phone number, so that we can keep ourselves abreast of what's going on. So, she may tell me, "Oh, this is happening.", and I say, "Okay, I can go and have a look.". So, I go and have a look and she cannot be there, she has to be with somebody else. So, I give her a call, "Eh, Patient A, B, C is having this problem. Blah blah blah. I started her on (some medicine or treatment). Can you do this and that?", and she says, "Yah sure!". And then after that, maybe six to twelve hours later, I give a call and (ask), "How ah?". And she says, "I did this, this, this. And she is much better, and these are the parameters.". So, we're able to, in a sense, tele-medicine and manage them. We may see each other or we may not, but all the time, the patients' care is coming along, and we're able to bring comfort to the patient, give some relief and so on and so forth, in managing the patient at home. So, that's where we found that actually communication is very important. It comes down to communication, whether it's with doctors and nurses, doctors and doctors, doctors and carers, doctors and allied health. Communication is key.
C	C here. I agree. I have similar experiences as A. So, home-based hospice care also has a role to play, even though the hospice has their own hospice doctors to do the visit, but they're quite busy. So, we know the patients very well, so we work very well with the hospice nurses from home-based hospice care. And I think that this is something that the General Practitioners can do, because they know the patients so well. And all of us do home visits, I mean, use house calls or elective home visits. So, this is part and parcel of the care plan that you can give your patients. The other thing about communication, you were mentioning about government sector doctors *[laughs]* being so difficult to communicate (with). What I normally do is, when the patients go and see them at outpatient, I tell them to give me a call using their handphone while they are in the doctor's room, and then pass the phone over to the doctor, then I have a word with him. Then, I catch up on what's going on. *[laughs]* That's one way to short-cut the system.
A	A here. Myself, I find it very useful to make a lot of friends *[C laughs in response]* among my hospital specialist colleagues. So, very often, I'm a creature of habit, so I have a tendency to prefer to go to the same person *[C interjects and agrees, "Same."]*. And once I text them, the reply is very quick. And we've actually had cases where the patients are actually seen in another hospital, and they have not been managing with the decant that they have been giving, and they were desperate. And that's where we've actually been able to activate the help from another specialist colleague in another place, and who may have a more sympathetic ear and they would be more responsive to their requests and their needs. The outcome is three, four hundred percent better, even though it's the same medication. And human beings are very interesting. Once they BELIEVE in something, they get better. Somehow, they get better. So, these are things *[trails off]*. So, it's just like,

	again, I said, it's (about) the communication part. And of course, … I've had the same experience as C, where you actually have a patient who is seeing somebody else, and then it's not somebody that you know and you try and call them up because there's some issue with the drug, and the only response you get from the ward is "Go to A&E (Accident & Emergency).". *[C laughs in response]* And then, the only response you get from the department is "Go to A&E. The doctor cannot be *[trails off]*.". *[C laughs again]* Then, at that point, OKAY, I'm going to call the BOSS, and when I call the boss, and then the boss will call the junior, and then the junior quickly call(s) me (back) and says, "What happened?". *[C laughs]* So, then after that, (he says) "I will see this patient at this time TODAY at this place.". So, there are ways and means of getting there, but you don't really want to go over (to) the head, because after that it's not nice. So, what we try to do is we try and communicate *[laughs]* with the patient directly, so that you can actually manage the patient better.
M2	It's an interesting observation, because in the hospital system, we THINK we pride ourselves in multidisciplinary care and how it's easy to communicate between specialists, and it's easy for me to reply to my oncology colleague and all that, and YET, there seems to be this barrier between primary care and the hospital specialist, whereas in the system itself, actually the complication is -
C	*[Crosstalks]* - so, C here again. This communication care between the specialist and the primary care is actually PARTICULARLY private care. So, you know, the discharge summary always automatically goes to (the) polyclinic, even though the patient has a GP (General Practitioner). I've never seen a discharge summary directed to the GP (General Practitioner) by name, you know. They don't bother to find out for the patient who is (his/her) regular P (General Practitioner). They don't keep the GP (General Practitioner) updated when the patient is admitted to the hospital. You know, that's another problem. It should be automatic. All right? They should find out who is the primary care provider and make an effort to them informed on the phone. It's the GP (General Practitioner) who has to keep calling the system. And so, (upon) discharge, (patient is) discharged back to polyclinic, even though it's my patient. So, these are some of the things that we observe. It's the primary care that has always been shunted out and left out in dealing with the government sector.
A	So, perhaps I can say that one of the barriers potentially is actually in the tertiary centres, the specialists themselves are not even AWARE who the patient is being followed up on.
C	Yes. C here. It should start from A&E (Accident & Emergency)! From A&E (Accident & Emergency), they should identify who is the primary care provider, and it should be written in the case notes (that) that doctor is the primary care provider for this patient, and (in) every step of the way through his process in the hospital until his discharge. The primary care provider should be kept informed. It's a two-way traffic.

	They should also get information from the primary care provider about the patient. They won't -
A	*[Crosstalks]* – yah, I just want to QUOTE something: this is from my friend, who is a specialist – this is A by the way – from one of the restructured hospitals, "I wonder why we don't have more family physicians. I have been so frustrated by the level of GP (General Practice) care of patients from the hospitals, to the point that I actually tell most of my patients, on GP (General Practice) follow-up, to switch to OPS (Outpatient Services) (instead)". Sad face. But I don't think it's just me. I think there will be some black sheep. There always are, just like there are black sheep in hospitals, there are black sheep in specialists, black sheep in nurses. Whatever lah! You know! BUT the prevailing feeling is that the GPs (General Practitioners) out there are just not up to scratch, and I think there may be some basis to it. And the question is, as I've said earlier before, we're not just "cough and cold doctors"; we try and be holistic; we try and be evidence-based; we try to have at least minimum standards that commensurate with managing a patient's problem. Also, I think when it comes down to it, it's really (about) perceptions. I mean, (it's) partly our fault, because maybe in primary care, we don't have those standards. But very often, the system itself, not necessarily the government system, but the system whereby many doctors in the private practice actually work, is actually in the insurance and the third-party administration systems, whereby cost is a factor, and standards don't QUITE get met, because the cost don't allow them to be met. So, that's where you have a problem that many clinicians may be a bit stuck from a fiscal point of view, as to whether they managed a patient properly or not. In the past, we also used to do a fair bit of insurance cases. Then, as we became *[trails off]*. We've done more and more work, and we begin to, as all the guidelines begin to come in and so on, we realize these are bare minimum standards that we at least should have. So, we have this chronic disease management programme, the "CDMP", where we have certain minimum standards. So, we start to do the means test, and you "at least must do this". And then, but when you are actually doing it under a TPA (Third Party Administration) Scheme, it's hard to follow through these numbers – not say, impossible, but you may end up losing money. Okay? So, many of us have actually decided not to continue (with) any of these TPAs (Third Party Administration) because it actually curtail(s) how we *[trails off]*. Or actually, SOMEHOW, we may not be able to do as well as we should. So, you know, as C has mentioned, there are many factors and some of these factors are due to how patients are COVERED with regards to what is covered in their health scheme.
M2	All right, let's go to the last question which is on community resources. So, I think A, you mentioned earlier about your experience with working with the nurse from the palliative care team, and how that has been a useful resource for you in the community in managing the patients, especially in end-of-life care. So, what other resources do you think (are) needed or (are) available in the community, that you can tap into to manage patients' oncological needs?

A	Well, I think the – A here – I think as I mentioned earlier, communication, to me, is of paramount importance. So, my ability or facility to contact the attending oncologist directly or at least the oncological TEAM who's managing the patients DIRECTLY, would be a great help, because once we encounter a problem *[trails off].* And we only call if there's a problem. And then how to go on to further manage that? You actually facilitate a lot of reduction in unnecessary referrals back to the hospital. So, that way, you can actually still continue the management. So, I think that's one of the things on my wish list, that I can communicate directly back to the home institution.
M2	Any other thoughts on this?
B	B here. I think patient support groups are very important for cancer survivors. That's where they can have friends going through a similar experience and having a same problem to share with (one another) and support (one another) emotionally in the support group.
M2	Do you know of any cancer support groups out there in the community?
B	I think the cancer society does provide. *[inaudible; 55:24 – 55:30min]*
A	*[Crosstalks]* – there are a few. There are a few around! (For) breast cancer survivors, yah, there's a society.
C	*[Crosstalks]* C here. You can google *[laughs]* what's out there. I think to expand on that, maybe as far as palliation is concerned for the coordination of resources, then agencies like the AIC (Agency of Integrated Care) or something similar, you need a case managers who can coordinate, because GPs (General Practitioners) need help in the coordination of resources, especially where to go, who to refer to, like, for example, if you want to refer to hospice or palliative care, they would know what's the best. The GPs (General Practitioners) don't always have the time to go and find out for themselves or their nurses might not have time, so you need a number to call. AIC (Agency for Integrated Care) does this service, I think, for placement and the mobilisation of resources, and they can coordinate it for you and keep you in the loop as well. That's one. I think that's community resources that you can engage. The support group is a good idea, but we also need to know where they are and how to get in touch with them and how to refer our patients to them.
M2	So, do you think case managers for this sort of patients would be useful?
C	Yes, definitely, especially if they work with us. And we *[trails off]*. It's always the "who to call" and "how to call" that's the main thing.
M2	But there are case managers to the case?
C	Yes *[laughs]*.

A	Sometimes, certain case managers are way, way better than other case managers. So, this person quits and then – *[C laughs and interjects, “Yes!”],* so you are left with the other type. *[laughs]*
C	Yes. So, yup, so just like in anything, there’s good and bad.
M2	Anyone else? Any other *[trails off]?*
M1	Okay, there was one comment by a doctor from the previous group. They said that in shared care, it is really difficult from the oncology point of view if you want to discharge a patient. Who do you refer to? Do you have a group of partners in different parts of Singapore, do you refer out or do you ask the patient, “Do you have GP (General Practitioner) that you can refer back (to)?”. How do we find the GP (General Practitioner)? So, this senior family doctor was saying that you have to spend the effort, you have to find THAT particular GP (General Practitioner) who is interested, who is motivated, who wants to believe in providing more holistic care. So, how does the tertiary centre find that GP (General Practitioner)?
A	You see, actually, it’s a bit of like cat chasing (its) own tail (when) you are trying to see who’s the GP (General Practitioner) or family physician who is interested or are they equipped. I would say that ultimately, it is the patient who will choose, and the patient chooses who he or she thinks is one of the best to take care of him. He could be the doctor that he sees him when that time, they were small that time or whatever. Or it may be a new doctor that they met but somebody that they really trust. And so, it’s finding who that patient thinks he belongs to, and then, relying on that relationship, … in order to better manage their problem in the future. So, I would say that one of the main problems that we face in Singapore is that NOBODY belongs to anybody, and everybody is fighting for the same pie, when actually you shouldn’t be fighting for the same pie. You know? Patient should choose who he wants to share his pie with, and then, after that, in fact, if there was no pie, but the patient was already having this relationship with the doctor, and the doctor was already committed to taking care of this patient, then subsequently when the problem comes up, it’s like taking care of a relative’s problem and (the doctor) just do(es) accordingly. So, what I feel, very often, especially based on what I’ve just shared with you from my specialist friend is that many hospital clinicians don't have much faith in GPs (General Practitioners) in the community, partly because they… are not governed, like in the polyclinics where you have protocols, and we can scan and look at all these things and see who is out of line and so on, because … it’s like an amorphous mess and nobody know what’s going on last time. But I think there are a CORE group. Definitely, there are a core group of folks that we know personally, from professionally, that we know that are doing very good work. And so, first of all, there is that way of knowing who is good out there. Then, the second thing is, once you know who is good out there, then you, as a first, approach these people whom you think are good out there in the system. And in order to do this, we will give the support to you all. So, that’s how I would do things in the first place. And the second stage of it would actually be to upgrade the level or the status of

	the family physician, so that the family physician can hold his head up high and say, “Look, I am an FP (family physician). This is how I do it. I don't do (it) like the rest. And these are my standards.”. So, once you have THESE standards, and they are verified, they are accredited, then you have confidence that they are going to do what you want them to do or HOPE that they can do properly, so that your patient care will continue to be at the same, good level.
C	C here. So, I think it’s not the first time that this has been done. I think with mental health, I think you trained the doctors and you get them to do a diploma if necessary, but I don't think you always need to have a diploma programme, but that’s the way of finding out who’s interested (from) the doctors who put themselves forward for more training and attending courses. Then, you establish whether they’re interested in managing your cancer patients in the community, and then you form a list, and this will be the people you refer out to. Of course, the list should be a big list *[laughs]*, but (it might) not always so. So, if you are asking who and where to go to, that’s how you start a list. And then, it should be built up overtime. I think mental health is doing that. So, those who are trained is where IMH (Institute of Mental Health) will refer schizophrenic, stable patients to them, because they know that they’ve been trained and they can trust them. I think palliative -
A	*[Crosstalks]* – or we go further, let’s say, for example, like in my practice, both of us are GPs (General Practitioners), but I can see the patients who have mental health issues and claim from CHAS (Community Health Assist Scheme) and so on, whereas my partner cannot, maybe because I’m already (in) MMed (Master in Medicine) and above. So, this may be just a matter of whether or not you have already got(ten) the degree or tertiary training at that level, or whether or not you get there by doing a diploma, and so on and so forth. Or you could just be on the family physician register, and then, accredited, and I think anyone who has actually MADE the effort to be on the family physician list probably already has some training. And if there is some tweaking, they can manage. I always felt that the medical doctors are actually very intelligent, just in order to be able to get into medical school. And if you can train them to the level of at least a junior registrar, a junior registrar and all who holds the basic specialist training type of person, so that they can manage the case, say, as you would manage in the SOC (Specialist Outpatient Clinic) in a public institution. I think they can well-manage the case if needed. So, even for myself, I’ve done rheumatology outpatient clinics; I’ve done diabetic outpatient clinics within a(n) institution, and basically there are just certain things you need to be looking out for and certain things you need to be able to manage. And once you’ve done that a few times, it’s quite doable.
B	I’m B. Can I just add a few words? I think at the end of the day, I personally feel that the patients should be the one to choose who they want to follow up with, because it has to be somebody that they can trust (and) somebody they are comfortable with. And I think we can continue to train the GPs (General Practitioners) and family physicians. And that’s also the important thing with the survivorship plan, because

	once they have this, and if they have the guidelines as to what are the complications and side effects to look out for, I think it'll be great help, and the patients can actually go to anyone, any doctor that they are comfortable (with). And the patient should be one to choose.
M2	All right. Thank you. I'll just call this to close and then, we can stop recording.
	[Audio recording ends at 1:05:08min]
