# Supplementary material for: Implementing a community-based shared care breast cancer survivorship model in Singapore: a qualitative study among primary care practitioners
Source: BMC Prim Care. 2022 Apr 8;23:73. doi: 10.1186/s12875-022-01673-3 (PMC8991467; doi:10.1186/s12875-022-01673-3)
Supplement: Supplementary file 3 — Additional file 3. A compressed folder containing the raw data transcripts and demographics data collection form. [file 12875_2022_1673_MOESM3_ESM.zip › Supplementary Information File 3/IDI (09.03.2018).pdf]

Transcript for IDI 3rd September 2018

Key:

Moderator / Interviewer:	M1
Respondent:	Participant A (A)
( ):	Paraphrases, additions to or rectification of grammar, vocabulary and/or truncated sentences.
[ ]:	Non-verbal, e.g. *[xx laughs] [pause]*
…:	Removal of false starts, repetitive or ungrammatical long phrases
CAPITAL LETTER:	When there is a louder emphasis or stressing on a particular word or phrase

M1	Thank you, A, for, accepting our invitation for this in-depth interview. Today, we have six themes to discuss. Please feel free to interrupt and let us know how you agree, disagree. The information will be deidentified. Your privacy, confidentiality will be ensured. The first theme is that we'll like to know about the background survey on your current practice. So, would you be able to share with us your experience with cancer survivors?
A	So, I usually see them in the context of them visiting for other presentations. Most of them are patients with chronic diseases, but sometimes, they are here for acute presentation of illnesses. They will mention that *[trails off].* Some of them do mention that they have past history of cancer or whatever, but some actually don't, unless you specifically ask for it. So, you realize that when patients present to you in primary care, they may not think that it is relevant or important as part of that history-taking. And the focus of the consultation would be (the) acute presentation, because both the doctors and the patient(s) give the impression that, okay, once the patient is being followed up by the cancer centre, then care for the cancer is generally over there, over in the realm of the expertise over there, although I do ask about some symptoms, because I know that cancer has a very diverse dimension to it, so I do ask "How's treatment so far?". And sometimes, they do tell me their side effects. I ask them about how are they taking it, which means, how are they coping with it, and see whether they may need some support for that. So, it will be on an adhoc basis if I sense that a patient is willing to share more about those things.
M1	Okay, thank you. So how about the perceived barriers of the shared care model that we presented? You know, because we know that cancer is a serious diagnosis, so looking after them in the community may have barriers. Maybe you can share in terms of patient-related, physician-related and healthcare-system(-related) (factors)? Like, what do you foresee are the barriers, and would you be able to share your proposed solutions how this can be overcome?
A	Okay, so I know for patient(s), they may not trust the primary care provider to be significantly involved in the care of cancer for them, because to them, it is such a

	serious problem, "I have so-called a specialist.". So, the perception that the specialist can deal with all the issues, I think, is still that, so that would be one barrier. I think the other patient(-related) barrier, again, I think it's (due to the lack of) patient education that they do not see the primary care provider as… need(ing) to be involved in the care for their cancer. Again, it's the mindset that "Okay, I'm being taken care of by the specialist over there.", so that's (a) the patient(-related) barrier. From the physician point of view, certainly one (barrier) is understanding the follow-up protocol a bit better in terms of what is expected for the follow-up. And also, sometimes (it's about) dealing with what I say as "not easy to resolve" issues. So, for example, I have a patient who is (diagnosed and in remission for) colorectal cancer but (it led to) neuropathy (and) it's symptomatic of Type 1 medicine that is given by everybody. Everybody is stuck, so (they) don't know what to do. You see, there's no solution to the problem, so I can see the specialists also trying their best to give some Gabapentin and all those things, but still it doesn't work, so there are things not solved, so that become a challenge for the provider, if there is no access to second(ary) support. I think the third (barrier) from the physician('s) point of view is (that) currently, there is no proper hand(over) from their hospital to the primary (care) provider. If there's going to be a shared care model, to me, it's quite critical there's a clear care plan (and) the (defined) role(s) of the specialist and the primary care provider, and that's one (barrier). And the other barrier is the uncertainty of being left to (feel) not supported within the system for providing care for such patients. So, it'll be good, for example, if there is (a) resource or somebody to kind of contact in the specialist centre, in case there are issues, so that the primary care provider will not feel, "So, now I'm managing on my own and how do I communicate back?", especially (because) the appointment back to the hospital can be a while.". You know, sometimes they see their patients six-monthly or one-yearly. And then, in between, if I manage, how do I get connected back in case of problem(s)? So, that reassurance is quite important. As far as healthcare system is concerned, … I think the time spent will probably be longer, because sometimes cancer patients need a lot of time explaining, evaluating, and also doing the psychosocial aspect, they all require time. So, the consult time will be longer, but I think the current funding mechanism… doesn't fund that kind of long consult, the long consult that may be necessary for such a care. The other one would be, I suppose, the training of the primary care providers, in terms of (imparting) a bit more knowledge, because if you are involved in those care, then patients do ask questions related to the care given in the hospital. And if it's really a true shared care, it must be enough information from the primary care provider to do something more than just doing a test, (like) what are the drug side effects, the common side effects, having a bit more understanding of the matters that the hospitals are monitoring, biomarkers or whatever, so that in engaging the patient in providing care, the primary care provider(s) can do more than just repeating mammogram, repeating whatever, you see. So, I think that will be some things I can share on this question.

M1	Thank you. So, can I understand, from what you say, you need, like, a coordinator. So, should this coordinator be from the cancer centre, or can it also be from the primary care?
A	So, it depends on what we think the role of this coordinator is. Actually, when I said coordinator, (it's) not really so much a coordinator, but somebody that the primary care provider can look to for information when there's a need. So, for example, patient present(s) with new symptom or whatever, so there has been some progression, so we need to get back to say, "Do I need to refer you back earlier, or is there something we can do on our end at the community level?". So, that kind of communication would be important between the specialist and the primary care provider. The coordinator part, I'm not sure whether they have it in the hospital, in the sense that if patient default(s) follow-up, is there a sort of active process to kind of track them, because technically, if you say that there is a need for such long-term follow-up(s), then patients, being human(s), sometimes they feel well, they may just drop off. Then, if there are situations where clinically or epidemiologically or whatever found, that things can crop up (along) the way. Then, we need to so-called get them back and contact them, whether we see that as an important role in the system to put in or not so-called for the "defaulters". So, (I am talking about) a coordinator more from that angle.
M1	Yes, actually, at the cancer centre, they do have a system of tracking the patients that default, I mean, who actually are supposed to attend the clinic, but then, they usually just recall three times, and then, after which, there is no further recall?
A	Okay. "Recall" means couldn't contact or contacted operating theatre come but refused three times and that's it, (taken as) doesn't want (treatment)?
M1	I think they are contacted either by a phone call or by a letter. I guess, usually, it's a letter sent out, and then, if after the three times, yah, they are not really followed up (on). But most of time, the primary doctor, who actually sees the patient, will also look through, because sometimes they could be admitted; they could have other more serious problems like a stroke, things which render them unable to come. *[A interjects, "Okay, could be other reasons lah?"]* Yah, there could be other reasons. So, there is definitely a natural attrition rate. We are not sure whether it is good for this group of patients to just default. I mean, we hope that they will ultimately be looked after by a doctor who can also look after the cancer as well. So, I mean, in terms of the healthcare-related system, we understand from the previous focus group discussions that the average consultation time is five to ten minutes per patient, and they have to look after chronic diseases, so would there be any plans to have, like, a special programme, like dementia programmes? Some of them actually shared, will cancer be one of these programmes as well?
A	So, I certainly see that it IS going to be done; it has to be done in a special clinic. It will not be possible to do it in a general clinic. And in fact, I'm not sure if we need to create a special clinic, because in primary care, we provide care holistically. So, I

	find that even in my dementia programme, my mental wellness programme, the thinking behind it is meant to be fairly short-term, that means to deal with it until it stabilize(s), then finish and go to the family physician(s), but they manage as a whole, because when you are at the specialized clinic, it's still very disease-focused. *[M1 replies, "Ah ha! I see."]* So, the initial consultation is to deal with it, evaluate it and then, come up with a plan, and once the condition is stabilized and well-managed, it can continue on in a family physician clinic, where they manage NOT JUST the condition, but in the context of the other diseases. Otherwise, there will be multiple specialized clinics and it's not sustainable already, you see. *[M1 agrees, "Yah, that what they become."]* And the whole idea of a family physician is a generalist, so to speak, unless there is specialized care that they will need. And things like dementia, mental health, even cancer follow-up, (are) actually part of care of the person as a whole. So, I would see that we may start off with a specialized clinic, but the other option to consider (is that) if the process of the care is really not too onerous, we'll build it into current family physician clinic.
M1	So, what is the difference between a specialized clinic and a family physician clinic?
A	Okay, So, family physicians also started to deal with complex chronic diseases. Okay? So, the patient will come in and the polyclinic thing will be on diabetes, hypertension, lipids, asthma and CCF (congestive cardiac failure), renal impairment, the whole works, and also the complications. And because most family physician(s) are trained to manage them WELL in the beginning, so we can run a family physician clinic. But when we started dementia and mental health condition, not ALL family physician can manage them well. So, the two programmes started with very strong specialist input to help build up the capability. And so, a group of people have been trained, but we hope that this knowledge can diffuse into the general family physician community in polyclinics, so that all of them can manage, just like they can manage diabetes, hypertension, lipids (et cetera). So, that's current thing is that we use second-tier clinic to kind of accurately… evaluate, assess, to come up with a plan, and once the active issue has been settled, this patient should be referred back to the family physician clinic, and this patient, this person is managed as a whole and not just a disease component only. Because in the secondary clinic, we need to look at specific conditions, right? So, similarly, I think cancer follow-up could use this model. Whether we start out at first, then move it in, I'm not sure. My own take is, if the task or the care path is not too onerous, we can build in straight that way, which means that if I see a patient that has diabetes, lipids, if patient is (also) a breast cancer survivor, for example, and if things are not too onerous, I can ask the relevant questions, do the necessary test, and I can manage this person in the context of a family physician clinic. So, I think we can explore models, so as we talk about details, then we know what is required in the session.
M1	I see. So, can I just have an understanding on the dementia and the mental well(ness) specialized clinics? How were they selected, these two conditions? I mean, why not other conditions?

A	Oh okay! Well, I suspect one of it of course is prevalence, importance, and the other one really is support and funding. So, ministry started the programme to really to fund special clinics, because we do need. So, like I mentioned earlier on, when we first started, although many of the family physicians were trained in Geriatric postings and whatever, but because they have not done it for a while before when they come to the community, so there is a little bit of leaking of knowledge, so you need to really scale up. And so, we need the specialist to come in and provide that guidance. So -
M1	*[Crosstalks]* – is this for a short term that they are coming in to help to run the clinic?
A	Oh, it's been for a few years and they are still around, but they are doing for a different purpose. So, the special clinic also has residents over there as well, so they provide a bit of teaching, so there is a bit of teaching element as well, so they put all these thing(s) together - service and teaching together. So, I would foresee this has been a couple of years, three years or two, three years. Since then, these people, who are becoming new family physicians, will be more equipped. This means that when these people run their general FPC (family physician clinic), they are able to manage this kind of mental health, dementia clinic(s), because they have gone through that posting and that handholding from the residents. So, hopefully, down the years, we build up a pool of people to do that, then there will be more and more people to do that, which means, the specialists may be (more) reliant on them down the road, actually, (when they) build their own internal capacity.
M1	Yah, that is a very good approach on how we can build up the confidence in the community. So, from other focus groups, there was also some input about the family physician having attachments at the cancer centre itself. Would that be useful, feasible or is it more fruitful for the specialists to come over to the primary care setting to run the clinic? Between the two, which model do you think will be more fruitful?
A	My personal preference would be to go to cancer centre. The reason is because it is more concentred (with regards to) the cases. If you go to the community, after ten patients I see, one whole day I only see one (cancer) patient. But really, if the role is for teaching purposes and to equip the family physician to manage that kind of work, then there can be concentration and very intensive exposure to the kind of cases to follow up. So, that will probably be a better model. Of course, the difficulty is time, resources, time resources, on our side, but I think that will be a more meaningful way of learning.
M1	Because we understand from some of the focus group(s) that some senior family physicians… know that they are trained and equipped to manage osteoporosis and also osteopaenia, which is one of the important side effects of the drugs, but often they do not have the resources. They cannot order a bone mineral density test and subsidized, so although they have the equipment, they can't practise. So, after a while, the experience, also, is not there. I mean, so that's why they are saying that

	it is good for them to be able to do also, but in terms of management level, how do you allocate resources? How do you decide to allocate resources to let staff go for training, for attachments, to learn (in) this area?
A	So, every staff have *[trails off].* We do have training plan(s) for our doctors and nurses, and that is usually in line with our work plans. Then, we need to think of ways (of) how do we sustain it. So, we have sent people for HMDP (Health Manpower Development Programme), people for training, so these are all training investment(s). It has to be born out of a need, and then to be able to sustain it – that is one of the basic(s). (For) small numbers, we can do, so I think we have to scope up the resources that we need, and then, to plan for it. It certainly would be useful if there's some form of funding support as a programme, just like I know when they do support GPs (General Practitioners) for programme, when GPs (General Practitioners) are taken out for time to do other things, they actually fund for the time, so this is time that is actually opportunity cost to the GP (General Practitioner). So, similarly, at the polyclinic level, when people are away to do some of these work, it means time (is) taken away, and we need to back (up) with locums and things like that, then that becomes something that we need to see how to staff it.
M1	I understand that in terms of GPs (General Practitioners), like, if they are referring to the primary care network, they actually get funding from the Ministry of Health. But if the programme is run at the polyclinic level, do you know is the funding (obtained) internally, or it's still possible to get funding from the ministry level?
A	So, let's say, if you use a mental wellness and the dementia programme, they recognize the need for separate funding because this is a new programme. *[M1 clarifies, "So, it's separate?"]* It's separate. It's a separate funding to run the two programmes, so… it needs to come from separate resources - different thing we are doing.
M1	I see. Okay. So, that will mainly be in the healthcare-related (aspect). So, in terms of physician training, so do you foresee (that), besides the clinical attachments, which other areas of training would be useful?
A	I think we probably need to define the learning objective first, find out what is the current knowledge gap. And then, like all models of training, usually (it would be) in the context lecture(s), case stud(ies) and hence on, so maybe it's good to have these three areas too, to identify the learning needs first, then we scope up what best are the learning needs.
M1	Okay. One of the feedback from the focus group is that perhaps the main barrier is actually the patient(s) themselves - whether the patient is confident for the primary care physician to handle this patient. So, what do you think for the current level of confidence of patients in primary care? I mean, because I'm sure they are very

	comfortable to manage chronic diseases. How do we build up confidence in the primary care physicians, (such that they) can manage cancer survivors as well?
A	So, I think there is way out of this, you know, because currently, in primary care, we have already built a relationship with the patients. So, the patient(s) do trust us in many sense of that word, but because there's no formal partnership, and the patient(s) don't see it as a formal partnership, then therefore they don't bring up, and they also think that, "Okay, the cancer specialist will be there.". One model that we've considered is to launch, officially, a formal programme with a proper hand(over) of the patients from the hospital to the community. So, for example, it could be when the patient is suitable for follow up at the community, the hospital can come up with the programme, find out who's the primary care physician, where is he among the LIST of the primary care provider(s). I'm sure for polyclinics, this would not be a problem. If you are seeing the polyclinic for a follow-up, but I think those not seeing the family physician clinic, it's definitely not a problem, because they are familiar with the doctors, … then so, (tell them), "Now, we have a programme in the polyclinic. Would you like to be followed up in the commy? And the specialist, you can see - I don't know – once a year or three years.", or whatever that they feel is necessary. So, they tell them that there is a programme, and then, do a proper hand(over), and maybe there's a folder that says, "This is the programme. And then, these are the things that the family physician would do.". And then, IF NEED BE - ideally but it would not be easy to do - (for) the last session, the primary care provider join(s) the consult, so there will be a (face-to-face handover). So, it's like if I see a patient in NCC (National Cancer Centre) and just say, like, the patient recognize(s) me, I will recognize my patient, and say, "Okay, the doctor will follow you at *[name of polyclinic, omitted for reasons of confidentiality]*.", for example, or different doctors at a different polyclinic. So, the patient is assured that there is proper hand(over). So, in that context, the primary care provider needs to be able to get back to the hospital or get back to the specialist doctor in case there are issues or whatever. I think that would be ideal, but (we) need to see, (as) that means that (it) would take a lot of time and a lot of planning! *[laughs]* But that would be ideal, because then the patient will know, "Okay, I know now my care in this area is going to be with another doctor that I will be seeing in the community.", so the reassurance is there.
M1	Yah, because actually at the cancer centre, when we ask the patient whether they would like to be followed up at the polyclinic, at the community, actually they are very happy. But then, the next question is, are (the doctors) willing to do or are they happy to do so, because they already have to manage so many other aspects. So, I guess it really boils down (to) what … the expected role of the primary care (is), because from the list we looked at *[trails off]*. Perhaps you can look at the next list. So, we take it as a care plan, in terms of *[trails off]*. I mean, the recurrence is still something that we should manage at the tertiary centre, but (the) picking up of red flags would be good in the community. But, say, if I can just turn over to the other side (of the page of this list), (and see,) in terms of these areas, are these areas…

	areas that primary care physicians are trained in, in terms of especially "mental health", "anxiety" and "depression". I mean, "fertility", I guess is mainly to pick up if they have any concerns. The other thing is the "cognitive changes" – we noted that patients actually have memory issues, so we understand the polyclinic also has dementia programmes, (so) whether they can benefit from all these specialized programmes as well?
A	Yes, certainly, I think it will be. So, at the primary care level, the first thing we (have to) do will be the identification of issues. So, picking up (of dementia) is not a problem. It's the backend follow-up of the dementia that is needed. Some of them will probably have some *[trails off].* But for some, you'll probably realize that it will be a quite… not easy to manage, like "fatigue", whether you see anybody with fatigue *[laughs]*, so -
M1	*[Crosstalks]* – yah! *[laughs]* But I guess even in the cancer centre, it's also the same -
A	*[Crosstalks]* – yah correct, correct! So… it may be easier if we start with something that is more processed- oriented, and then, we can deal with it, so which means that it may be that when they are handing over… issues (to) the community, the care plan should include (information like) what has been resolved, what is still outstanding. You know, there must be an approach to the outstanding bit. Like I said, for example, my neuropathy patients (are) always "it's just like that". I mean, if they actually go cancer centre, they will tell (them) the same thing also! *[laughs; M1 laughs too and replies, "Yah, correct, correct!"]* So, but then, there will be time taken to talk about these, you know? So, it could possibly be a reservation. So, that may be one way to it. These are things that they have to resolve… before they hand over, so that focus on the primary care is really the follow-up, and not the active issues.
M1	Yes, that's right. I think dividing the role is important, because if the patients go to everybody and complain of the same problem, they won't really benefit from the care from that specialty area, because (for) family practice, the main specialty is mainly in prevention. Yah, because for the specialists to talk about exercise, work stress (et cetera) in the National (Cancer) Centre, it also takes away time for them to look into other areas and (what) is more critical, like recurrence itself. So, do you think the education of the patient is important?
A	So, with this, I'm not sure whether in the hospital setting, they have a – I'm sure they have – the "multi d approach (multidisciplinary approach)", in the sense so that they can deal with active issues, and then, what would move the primary care would be the continuous maintenance issues, rather than unresolved active issues passed out to the community, then the community (team) may have difficulty. And then, I can imagine, coming down to it, the primary care provider(s) would (not know what to) do and they are also kind of stuck (and think,) "Eh, let's get an earlier

	appoint to go back and see them because the problem is not settled.”. So, the understanding must be there.
M1	Okay. Thank you. So, may I ask that, in terms of the selection of the patients for the shared care model, do you think that it should be like the older patients with more comorbidities or it can be anyone with concerns?
A	So, I mean, currently my preference would be first(ly), they should be polyclinic patients first. The shared care programme should be -
M1	*[Crosstalks]* - that means the patients should come from the polyclinic and that means they are already seen by them, right?
A	That’s right, that’s right. To start off, they may want to consider starting a family physician clinic, because these are people who actually build relationship. I think the chances of patients (being) agreeable to do that will probably higher. But again, it depends on the care needs and the maintenance issue, but I don't think *[trails off]*. It can actually be young or old (patients), but if … the active issue is sorted out and the main issue is really monitoring the effects, monitoring the recurrence, monitoring for psychosocial (support) - that means, stable, just monitoring, just checking (only). So, the moment something is triggered, there’s (an) avenue to go back, then it can be anybody. *[M1 clarifies, “The needs?”]* Yah, because I’m thinking, if somebody has breast cancer operated on, completed chemotherapy and now on hormonal therapy, but (has) no other symptoms, then, young or old, because if there are no other psychosocial issues, then no side effects issues, THEN the primary care provider will be just check(ing) for recurrence and check for (side) effects, then that is a very biomedical thing, and therefore there is nothing to talk about. *[M1 interjects, “So, that is very basic, that everybody can -?”]* Yah, very straightforward. So, everybody can do it. Just need to make sure that the protocol is clear, then plenty of people can do that bit. And then, there are those with residual issues, maybe lymphedema or whatever, and actually stabilized. So (for) this one, again, it can be handed over, then the primary care provider know(s) that this is a stable (case), but what to look out for (would be things like) worsening or whatever, how do you manage when things worsen, some of the changes in this state, and when do they need to go back, when we need to have communication to go back, when to go back, is it very bad, what can we do before they go back, what is very bad, at what point is considered “very bad”, what can we do in the community. So, this could be lymphedema, this could be nerve neuropathy or whatever the treatment is being given, (be it) side effects of the medication or side effects of the condition, surgery or complication or whatever, so (when) these things stabilize, but there is actually a plan to manage it, but it’ll always be there. So, the patient knows you are going to be there, and primary care providers will need to know how… to monitor for worsening, what to do with some of the secondary complications and all these things. So, that one would be (important). The third one, which is probably not so ideal, would be the financial issues coming in. Financial issues not resolved, still depressed or whatever, so we hope that these

	things can be sorted at the active phrase by the multidisciplinary team at the acute centre, so that when they come in, it's (for) maintenance. I mean, for it to continue and the MSW (medical social worker) waiver is all done, so, no need to worry, because all the assessments have been done, you see, so it's maintenance, rather than pick up some of these (cases), how come depressed, then have to refer for depression or whatever. Let's say they have depression, you kind of stabilized the medication, then they can actually move in to the system, and we just see this patient and just monitor the mood, see each time for the mood. If there's escalation (and) minor changes, we can adjust the medicine or refer for (something else). But if it's not even sorted out, and (they) come in and we start from scratch and dealing with the issue, then (it) becomes more difficult, because, let's say, we are managing them in the context of the other conditions, there (are) the other area(s) of care we provide for them. So, my general preference would be, let the active issues be settled, and we do the maintenance. That should be the approach.
M1	So, for example, if they have been diagnosed with depression and so, they have been seeing the psychiatrist, and if they are stable -
A	*[Crosstalks]* – correct, then we continue to see.
M1	*[Resumes]* – but would it be still good for them to come back once a year to see the psychiatrist or there is no need at all, unless -
A	*[Crosstalks]* – I think, currently, in the primary care provider (setting), I mean, I have also started treatment and managing patients with depression, and they are well, stable, so there is no need to go back. But there will be, again, in the primary care level (that) we need to define our own limitation(s), like (at) what point do we refer upwards, so (that) it will be okay. Certain point will be okay. Our pulmonary is like that, still the best? *[laughs; M1 laughs too]*, then we've got to set up (the service) already. So, what I mean is the scope of work and at what level. So, we'll have to define that. We'll have to define that.
M1	One of the points which the survivor core group in cancer centre envisioned is that there shouldn't be additional follow-up up for the patient. If they are seeing specialist, and they see primary care in between, and then, still go back to specialist, then it really defeats the purpose because we want to cut down the number of appointments.
A	Agreed! Agreed!
M1	That means, for issues that the patients are stabilized at and they are confident managing, they should just be discharged. But in the (event) they need to be referred back, I guess if there is a pathway to be referred back *[M1 interjects, "Correct! That is key!"]*, or if they have a pathway to be referred to the lymphedema clinic or (for) certain areas, then they don't actually have to come back to see the specialist if it is for specific areas. So, that means the primary care physicians

	actually act as the SPECIALIST IN THE COMMUNITY. And then, would it be good for them to access the resources in the national centre as well?
A	I think if the protocols are written clearly, then certainly, why not? It save(s) unnecessary middleman that they have to go through this, go through this, go through this. So, that would be great if this can be done.
M1	I understand that for certain areas, for cancer treatment, like one of the tumour markers for CEA (carcinoembryonic antigen) is not subsidized at the polyclinic, right? *[A replies, "Correct, correct."]* So, I mean, if this is allowed to be done in the cancer centre, and the result (is) seen by the polyclinic, do you think that will create a problem, as to who is the ownership if the result is abnormal?
A	Yah, so this may be an example of whether the funder must come in. Let's say, the polyclinic is given a list of tests that we order for common conditions in the community, and the funding is given based on that list, so anything outside the list is non-standard test. Technically, we can do the blood test and we can make arrangements for those things, TECHNICALLY, like what you said. But the patients still have to pay a higher price. So, unless there IS a integrated way of doing funding, in terms of ensuring that what you pay at NCC (National Cancer Centre) and what you pay here should be the same, but where do I get the source of funding from? So, whether from another angle, you can say that (regarding the funding at) NCC (National Cancer Centre), since you are not doing to see this patient anyway already, can some of the (money) to fund some of these things can be done as a kind of support for this group of patients.
M1	Yah, because from some of our observations, like, for some of the tests, like full blood count, liver function and renal (function), are already subsidized in the polyclinic, and they cost quite a lot at the cancer centre. So, some specialists actually make the arrangement for them to be done at the polyclinic, but they actually bill the results at the cancer centre itself, but I mean, it really depends on the primary care (itself), because this one can be missed out if there is really any alarming -
A	*[Crosstalks]* – so, actually, this arrangement is not very good. In fact, we should discourage this because there's no formal shared care handover, *[M1 clarifies, "Oh, you are just making use of that service?"],* because the risk is that the result is kind of not trigger(ing) (a referral); because if (the) result is abnormal, it triggers off the system to (a referral), but now (in this case), it triggers to who? *[laughs]* And then, (the receiving physician) says "Eh, I don't know this patient! I don't know why is this test ordered?", so this is the risk of doing it this way. So, if anything, it should be a proper handover, an acknowledged shared care mode, and then, if there are any additional resources needed, we'll carve out what resources are needed. So, for example, you say that, as a non-standard test, CEA (carcinoembryonic antigen) costs fifty dollars. But subsidized (rates at) NCC (National Cancer Centre) cost twenty dollars. So, if the patient comes to polyclinic, CEA (carcinoembryonic

	antigen) will be fifty dollars. NCC (National Cancer Centre) will be twenty dollars, because thirty dollars (is) subsidized by government funding. But (I'm thinking) whether NCC (National Cancer Centre) can pass the thirty dollars to pay for this patient, so that patient just pays for twenty dollars, and yet you can see the result, and now, don't need (to) see doctor (for) consult at the hospital. So, it's (about where) the funding originated from, and then, if care is moved, whether that resource can kind of follow -
M1	*[Crosstalks]* – so, it should follow the patient?
A	Yah, should follow, to support the patient. That's one way to make sure that it's sustainable.
M1	That's good. Okay. So, … maybe (for) the last two (themes), (for) next (theme), we talk about (whether) is there a motivation for primary care to want to participate in this shared care?
A	I think to me is (that) if we put patients first, … and it's something within our ability to do that, then we should. And why do you need the patients to go to so many places if we are trained and equipped to manage them, because you are trained to do that? That, to me, will be key. Which means that, because we are not going to participate only in breast cancer survivor(s), we have other survivor(s) also, and we also have discipline(s) (like) cardio(logy) which is also doing a similar model, you see. So, I reiterate the point that active issue(s) should be resolved, because it's very difficult for us primary care to manage the active issues at one time. (For) minor adjustments, we will make, once there's variation. So, the general principle (here) is minor issue, maintenance issue, and (whether) we can build a relationship, then we can refer back, get back into the system in case there are problems. It can be at two level(s): one is through a backend information-checking, which means (it's) somebody the primary care provider can link up (with), (like,) "Eh, got patient referred here that time and somebody should know.". Sometimes, (they) may not even need a proper referral back, if that side can give some input, like, "Oh, this one! Can give some suggestion.", then it can be done (that way too). Then, (for) what criteria should we refer back and the process (to refer), that's one (aspect). The other (aspect) that we talk about also would be if there is additional test that is non-standard to what primary care… provided, so that the patient (doesn't) fear, "How come pay more here and pay less there?", so that there is a same kind, that in terms of the support, it has to follow along as patients move to a different level of care. Thirdly, (it) will really be the training bit. I think (regarding) the motivation part, I think we are all here to try and do a good job, so if we give the people the resources to do it, they won't mind doing it. It's when you ask them, "Your general clinic still continue to see (these patients)!", then… not possible, you see! Not possible, you see! *[laughs]* If (for) FPC (family physician clinic), we want to accept, it cannot be a lot of active (cases), because it is for normal chronic diseases already. We are (already) seeing that. If (we) still have… other maintenance (issues) and

	other active issues, then (the primary care physicians) will feel overwhelm already, so that will be another thing (we need to reconsider).
M1	I guess, with the increased incidence of cancer survivors, do you think that it is actually a good thing to step forward?
A	It is! It is! Actually, we had some conversation before with *[names a breast surgeon; omitted for reasons of confidentiality]*. I think he left already. *[M1 replies, "Oh yah, yah, yah! He did discuss it? Okay."]* He left already. Two, three years (ago), he did initiate a meeting to talk about it. We met at some meeting for a casual conversation and I think we are happy to do that, because it's really to provide more holistic care, but it didn't kind of kick off into any formal discussion, but we need to look at it as a whole programme. So, maybe that is one programme that they can talk about – a whole programme, not just the training bit, but the information flow, the support, the resourcing, the referring-back mechanism (et cetera), all these have to (be) look(ed) at as a whole package, so that your patients are reassured, your specialists are also reassured that everybody is handled over properly, … and it's a good follow-up programme, and obviously there must be resources and capability to manage that. So, yah.
M1	Okay. So, I mean, would a workgroup to work out the workflow be a good thing to start off?
A	If we want to do this, we need to form a workgroup to look at it.
M1	So, with the clinical (part) as an education as well, or maybe just the clinical -
A	*[Crosstalks]* – I think, maybe the clinical, unless we want to bring (in) the educational component. (Regarding the) educational component, I'm not sure what we have in mind but if it's just to equip the primary care provider, then the clinical people can link up to education, like, "Okay, this is what we need to do.", and really, it's education from the specialists' side (on) what we agreed we need to do, therefore, how do you train the doctors to do what we agreed that we should be doing at the primary care level.
M1	So, probably, it's something workable?
A	I think it's workable.
M1	Because when we look at our own cancer centre statistics, about half of the patients are subsidized patients. So, lot of them are actually from the polyclinics, but it's just that, you know, we just discharge them over, and sometimes we just hope that they end up with the right person.
A	It's hand(over), right? To me, it's hand(over). We need to work out a proper hand(over), the role of both hospital and primary care, we know our role(s)… and when one side hands (over), they know who they are handing to, and that they are confident in that person, and the one that sees (the patient) know(s) what (he)

	needs to do, and has the resource(s) to do what he need(s) to do. So, I think the whole thing has to (be) discussed along that way.
M1	So, the resources, in terms of the nursing, the labs and the imaging should still belong to that particular institution, that means, it should still come from the community, from the polyclinic, like the nurses, I mean -
A	*[Crosstalks]* – if possible… (it) should (be the case), because it's handing (over). So, I don't know, are we talking about shared care, does it mean that the hospital might not need to see them?
M1	Yah, that's why we don't really know, because that (model) in overseas, they are living very far off, they can manage with the primary physician. But some of them actually (go back) once a year. But the primary care physicians actually titrate all the medicine, look after the side effects, because they have their own set of support system. So, whether the shared care really needs the oncology review, whether it can be just hands off or it can be open date *[trails off]*? Because actually we have open date up to two years.
A	Okay, okay, so, like I said, if it's going to be maintenance issue, actually can hand (over) quite easily, because it's maintenance. But if there are active issues, then it is different, because we need to think through how to squeeze out the active issues, so (for) that one, maybe we start off with the maintenance issue first, those who… actually (have) their issues resolved. It may not be all (resolved) yet, not all stable yet, it may not be fully-resolved, but this is an issue. The idea is to keep track and make sure it doesn't progress, the psychosocial bit, may it be the medical bit, may it be clinical bit, or whatever. Like some (issues) could be permanent, like side effects could be permanent. *[M1 interjects, "Yah, I think we just watch it."]* Yah, just watch it so that it's stable. That's easier, you see. So, that may be (something we can to, to) start with the model.
M1	Okay, then I guess I understand that it will only be selected family physicians who will be selected to attend the training -
A	*[Crosstalks]* – actually, if you ask me, if you want, those who are running *[trails off]*. All trained family physicians attend the programme, because if the idea is to *[trails off]*. Okay, again, it depends on the protocol. Is it a complicated protocol to understand? If it's not a complicated protocol to understand, I think anybody, any family physician who's trained can kind of scale up quickly to go and learn the concept to do it. So, just now earlier with the breast cancer (checklist and care plan), basically it's checking for recurrence. This one, I think no problem to train. MMed (Masters in Medicine) can do this. This one, they can pick up, not a problem. So, let's say if it's a stabilized issue, this one is actually just checking on maintenance (and) whether there is any issue, so I don't think we need to (pick) a selected group, but to say, anybody can.

M1	Okay, MMed (Mastesr in Medicine). How about GDFM (graduate Diploma of Family Medicine)?
A	Probably, we start a bit slower, because currently, family physicians run by MMed (Masters in Medicine) first. I think GDFM (graduate Diploma of Family Medicine) runs last, so maybe you can go for those (to) run family physician clinics. I think ideally, next time down the road, we are also hoping the consultants in general clinic will also increase for doctors, so that may be - *[M1 interjects, "Oh! Overtime?"].* Yah, so overtime, there may be more people and more things, but now, (we do) slowly, going that way. So, we start off with the family physician clinics first.
M1	Okay, but will it be more costly to the patients?
A	So, family physician clinic, the fees are higher than the general clinic, but still cheaper than SOCs (Specialist Outpatient Clinics).
M1	But SOC (Specialist Outpatient Clinic), when they subsidize, it's thirty plus (dollars).
A	Ours, twenty something (dollars) only. *[laughs]* Cheaper, you see? So, the difference is still there. So, that means, instead of going to see the oncologist to do their mammogram or to do whatever, it's the same. *[M1 replies, "To reduce visits?"]* Firstly, to reduce views, and also, it's cheaper (in) the community, so the value is there.
M1	So, thank you very much. May I know whether you have any last suggestion for us to bring back?
A	I think we talked quite a bit already, and it's something that we are happy to explore further, to create a programme for the next run. I think the reassurance for the patients is to hand(over). For the primary care provider, it's to be able to get back to the system, the hospital, when the need arises (and) I think, for the specialist to be reassured there's actually a programme for the patient, that also takes care of the patient at least. Whether to see the patient, you may want to consider a staggered or tiered, that means, after first year, (review) once a year, okay then (review) twice a year, because (it's) just to check the thing, and then, by when, free, then they -
M1	*[Crosstalks]* – at least the patients are more assured that they can always come back?
A	Yah, they can at least come back. So, that may be one way to build confidence, and when the whole thing runs smoothly, then we tighten all the loose knots *[sic; probably saying "tie up the loose ends" instead]*, all the teething problem(s), then we are confident, then we can actually move away from all these. Can consider lah! I mean, another thing is that we have IT (information technology) now. So, I'm just thinking how does IT help. So, for example, anybody, every breast cancer patient should have a breast cancer mammogram at least yearly, once every year. IT can flag up. Cancer centre can flag up, because (the system) is also joined. If you are

	(checking) who don't have (it), just flag (it) up. Then, "ding ding" *[reference to alarm alert on system]*, how come these three patients don't have (mammogram)? Who has it been handed to? Then, can flag up to you all also, because with the system, it may be easy to do that.
M1	Because we are actually thinking of a new model whereby stable patients just drop and go with the mammogram. They don't even need to see the doctors unless there are some issues.
A	Agree, agree! That one, it's on your side, nursing side, whether the nurse practitioner can do the next step first. But I'm talking about (how) if you can capture (the information in) the system, if you have very defined process that we can document that care has been given, that means this protocol has been adhered to, like mammogram or whatever, then the system can actually churn (the information) out.
M1	So, (with regards to) the mammogram, is it still better done at the polyclinic or-
A	*[Crosstalks]* – we have mammogram in the polyclinic. I think, still, if it's still going to be there, then it should be done in the polyclinic. It should be done in the polyclinic. *[M1 agrees, "Yah, I think it should."]* Yah, it should one-stop.
M1	Yah. Thank you. We are very encouraged that the polyclinic is very open to cancer centre. And for me as a family physician, I think I believe in the holistic care, and I think we really have the better skills-set *[A and M1 laugh]*, I mean, for welfare, because I feel sometimes over there, I mean, when there is no cancer and they want to talk about issues, they want to talk about what food to eat, and what holidays to go (for) *[laughs]*, which I think is good but I think is a waste of resources.
A	Yah, we can talk about more useful things.
M1	Yah! I mean, for us, because we don't have enough time to talk about smoking, lifestyle, stress, occupation, so I really firmly believe so, that's why I'm trying to push this, and I'm really happy that we can find partners in the community.
A	Yah. So, you are a family physician -
M1	*[Crosstalks]* – okay, I think we better stop here -
	[Audio recording ends at 46:19min]
